# Synthesis, Biophysical and Biological Evaluation of Splice-Switching Oligonucleotides with Multiple LNA-Phosphothiotriester Backbones

**DOI:** 10.1021/jacs.4c11402

**Published:** 2024-10-14

**Authors:** Debashis Dhara, Alyssa C. Hill, Abinaya Ramesh, Matthew J. A. Wood, Afaf H. El-Sagheer, Tom Brown

**Affiliations:** † Department of Chemistry, University of Oxford, Chemistry Research Laboratory, 12 Mansfield Road, Oxford OX1 3TA, U.K.; ‡ Department of Paediatrics, Institute of Developmental and Regenerative Medicine (IDRM), University of Oxford, Oxford OX3 7TY, U.K.; § School of Chemistry, University of Southampton, Highfield, Southampton SO17 1BJ, U.K.

## Abstract

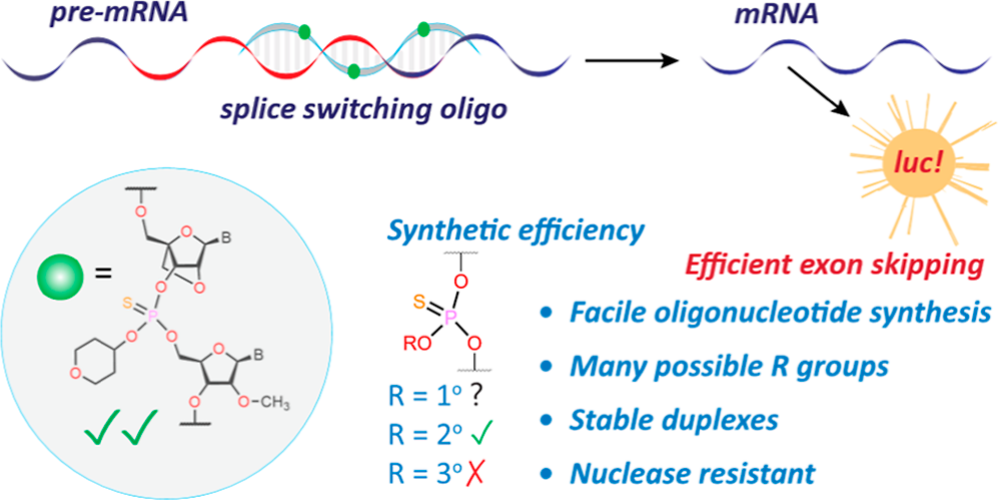

Polyanionic antisense oligonucleotides hold great promise as RNA targeting drugs but issues with bioavailability hinder their development. Uncharged phosphorus-based backbones are promising alternatives but robust methods to produce them are limited. We report the synthesis and properties of oligonucleotides containing charge-neutral LNA alkyl phosphothiotriester backbones combined with 2′-*O*-methyl phosphorothioate nucleotides for therapeutic applications. The nature of the triester alkyl group dictates the success of solid-phase synthesis; tertiary alkyl groups are lost during the P(III) oxidation step, whereas primary alkyl groups are partially cleaved during deprotection. In contrast, oligonucleotides containing secondary phosphothiotriester linkages are stable, and large numbers of triesters can be incorporated. The modified oligonucleotides have excellent duplex stability with complementary RNA and exhibit strong nuclease resistance. To expand synthetic flexibility, oligonucleotides containing multiple internal alkynyl phosphothiotriesters can be conjugated to lipids, carbohydrates, or small molecules through CuAAC click chemistry. Oligonucleotides containing LNA-THP phosphothiotriesters exhibit high levels of pre-mRNA splice switching in eukaryotic cells.

## Introduction

Antisense oligonucleotides are modified short single stranded synthetic nucleic acids that alter gene or protein expression via interactions with cellular RNAs.^1−5^ They function principally through mRNA splicing modulation^6,7^ and RNase H-mediated mRNA degradation.^8^ Double stranded siRNAs are also highly effective in gene silencing.^9,10^ Modified oligonucleotides hold promise for treating cancer,^11^ neuromuscular and genetic disorders,^12^ with the recent clinical approval of Inclisiran (Leqivo)^13^ for the relatively common condition primary hypercholesterolemia sparking intense interest in the field. Advantages over small molecule drugs include simple and logical design, strong and predictable RNA binding, and exquisite target specificity.^14^ Unmodified oligonucleotides are unsuitable for therapeutic purposes as they are rapidly digested by enzymes in cells. Hence, modifications must be introduced to enhance nuclease stability as well as improve pharmacokinetics, cellular uptake and reduce off-target effects.^15^ Sugar-modified nucleic acids including 2′-*O*-Me,^16^ 2′-*O*-(2-methoxyethyl),^17^ LNA,^18^ cEt^19^ and 2′-fluoro^20^ have been developed to provide nuclease resistance. The widely used phosphorothioate (PS) backbone^21^ improves cell uptake and enhances stability to nucleases but slightly reduces RNA target affinity, which can be restored by 2′-sugar substituents.

**Figure 1:**
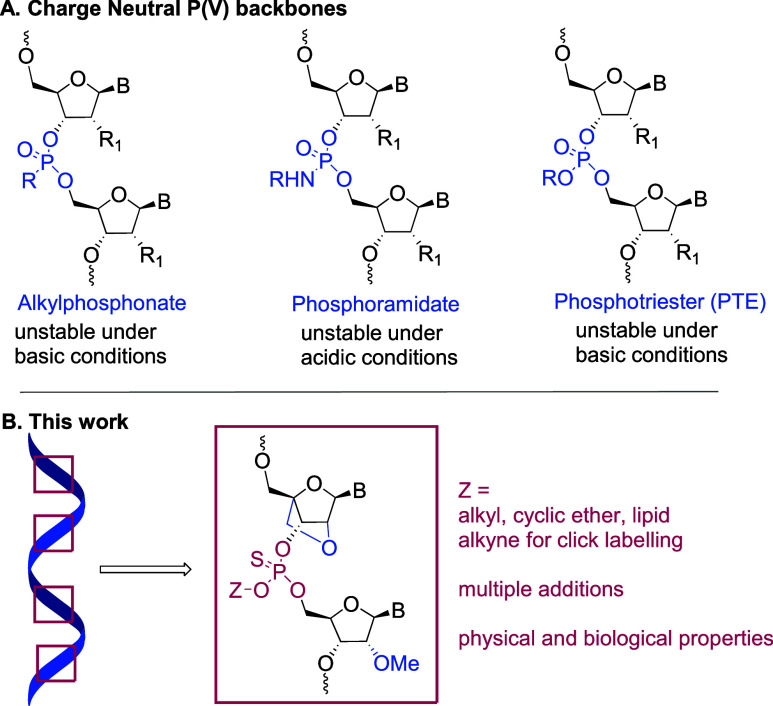
(A) Charge-neutral P-backbones. R, R_1_= various substituents. (B) Current study.

Reducing the net anionic charge of the oligonucleotide backbone has been explored in attempts to increase nuclease resistance, cell uptake and improve pharmacokinetics. This can be achieved by introducing charge-neutral internucleotide linkages.^22−24^ Charge-neutral phosphorodiamidate morpholino oligonucleotides (PMOs)^25^ are used to treat Duchenne Muscular Dystrophy (DMD), a genetic disease affecting 1 in 5000 boys globally, characterized by progressive muscle breakdown and with an average lifespan in the mid–late twenties. No cure exists but several PMO exon skipping oligonucleotide therapies have received FDA approval to treat genetic variants of this disease: Eteplirsen (exon 51),^26,27^ Golodirsen (exon 53),^28^ and Casimersen (exon 45).^29^ This provides the impetus to develop new charge-neutral oligonucleotide backbones, particularly as those currently in the clinic have limited efficacy. Some charge-neutral backbones can be challenging to synthesize; for example if the modified backbone is introduced as a dinucleotide (as is common), 16 dinucleotide phosphoramidites are required to enable synthesis of any required sequence.^30,31^ In contrast, phosphorus-based charge-neutral backbones can be introduced into oligonucleotides as phosphoramidites on solid-phase via just four modified phosphoramidite monomers.^32^ These “P-backbones” are of three main types: alkylphosphonate,^33,34^ phosphoramidate,^35,36^ and phosphotriester^37^ (PTE) (Figure 1A). Despite their favorable physical and biological properties, these backbones are reported to be unstable under the standard acidic or basic conditions used in solid-phase oligonucleotide synthesis and deprotection.^38^

Previous studies on phosphotriester oligonucleotides include their use as biomarkers^39,40^ and as biodegradable pro-drugs.^24,41,42^ Our own interest is based on the ability to vary the alkyl group to improve chemical, physical, and particularly therapeutic properties including cell uptake, and especially exon-skipping activity. Phosphotriester dinucleotides were first synthesized over 50 years ago.^43^ However, due to reported instability, the equivalent oligonucleotides are challenging to synthesize.^44^ This has been addressed by changing protecting group strategies^44−46^ and recently a method was developed for synthesizing hydrophobic and cationic PTE oligonucleotides.^37^ The alkyl-PTE oligonucleotides that have been studied so far include isopropyl,^47^ neo-pentyl,^48^ phenyl,^45^ dodecyl^49^ and cleavable disulfide.^41^ Stearyl,^50^ phenylethyl,^51^ isopropyl and tetrahydrofuranyl PTE oligonucleotides have all shown improved activity in mice.^24^ In addition, short 8-mer oligo dA_8_ and dT_8_ lipophilic phosphothiotriester oligonucleotides have been proposed as cellular delivery agents for PNA and PMO DNA analogues.^52^

## Results and Discussion

**Scheme 1:**
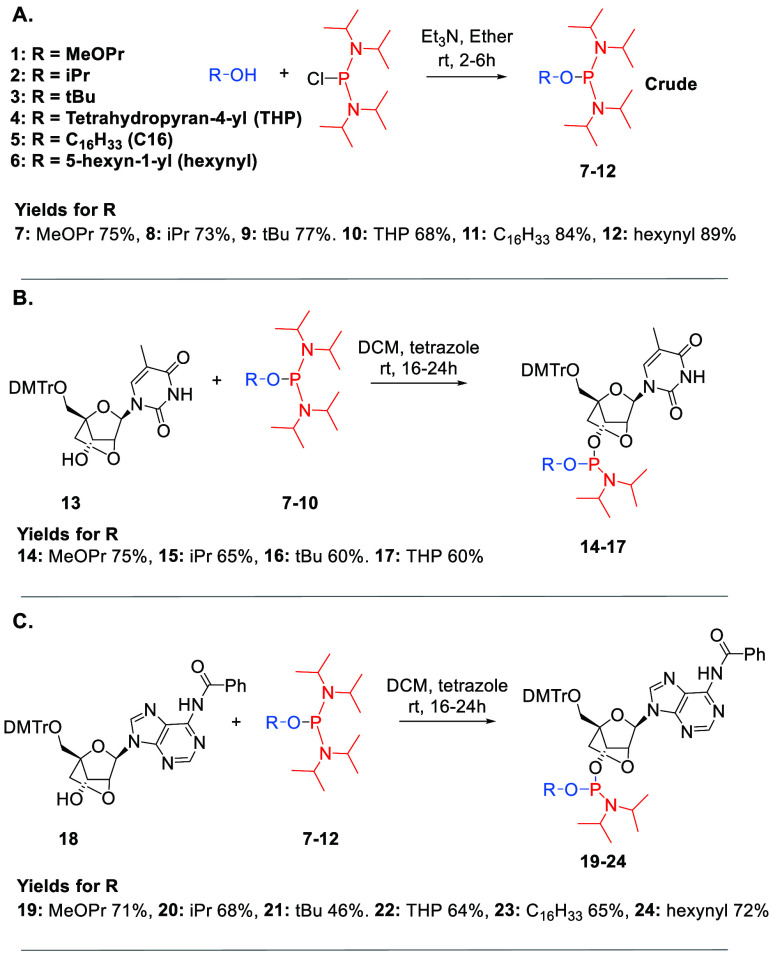
(A) Synthesis of Phosphoramidite Reagent. (B) Synthesis of Modified Thymidine Phosphoramidite Monomers **14–17**. (C) Synthesis of Modified Adenosine Phosphoramidite Monomers **19–24**. DMTr = 4,4′-Dimthoxytrityl

Here, we report the synthesis of mixed-sequence therapeutically relevant oligonucleotides containing charge-neutral phosphothiotriester (PTTE) and phosphotriester (PTE) backbones with locked nucleic acid (LNA) sugars, using monomers that are fully compatible with standard solid-phase assembly. Following our methods, we have introduced more than 50% of charge-neutral PTTE linkages, and we are not limited to this percentage. The synthesis of both PTE and PTTE oligonucleotides requires the preparation of nucleoside 3′-phosphoramidites in which the chosen alkyl group replaces the 2-cyanoethyl moiety of standard phosphoramidites. To prepare the required monomers, alcohols **1**–**6** were reacted with bis(diisopropylamino)chlorophosphine to give the phosphorodiamidite reagents **7**–**12** (Scheme 1).^53−55^ Next, commercially available 5′-O-DMTr-protected locked nucleoside **13** was reacted with **7**–**10** in the presence of tetrazole to afford the thymidine LNA phosphoramidites **14**–**17**. Similarly, locked nucleoside **18** was reacted with **7**–**12** to give LNA A-phosphoroamidites **19**–**24**. Phosphoramidite monomers **14**–**17** and **19**–**24** along with 2′-*O*-methyl derivatives of N6-benzoyl-A, N2-isobutyryl-G, N4-acetyl-C and U (Supporting Information Figure S1) were used for the synthesis of a wide range of oligonucleotides on the 1 μmole scale using EDITH (3-ethoxy-1,2,4-dithiazole-5-one) as sulfurizing reagent (**ON1–ON41**, **ONOX1**, **ONOX4**, Table 1). Coupling efficiencies of the triester monomers measured by liberated DMT cations were high (Supporting Information 7.0). All oligonucleotides were cleaved from the solid support and deprotected with a 1:1 mixture of THF-ethylene diamine (EDA), purified by HPLC and analyzed by UPLC-MS (Supporting Information 2.0). The chosen oligonucleotide sequence is designed to correct an aberrant luciferase mRNA splice site to give a luminescent readout of exon skipping in model HeLa cells (Table 1).^56^

**Table 1:**
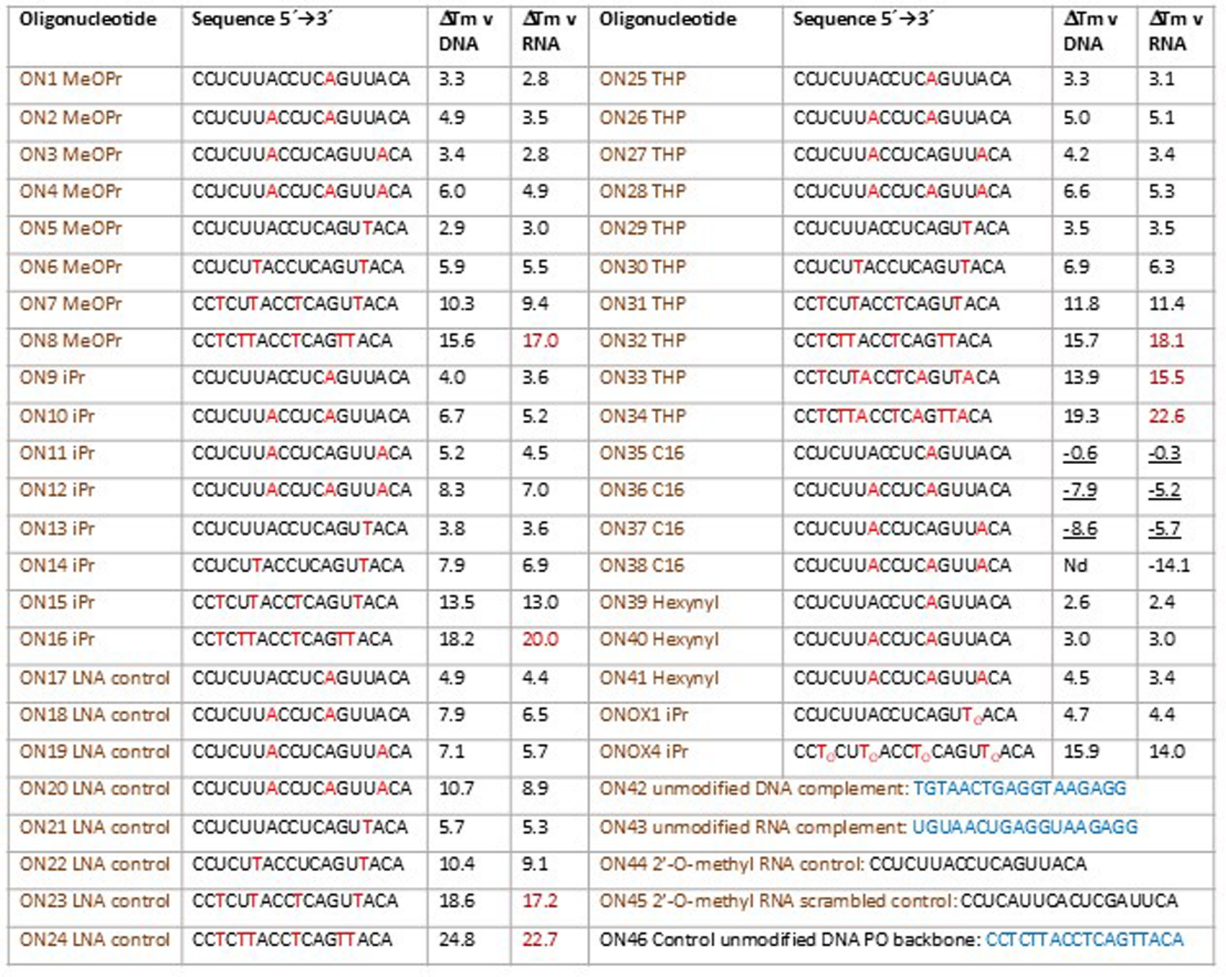
Oligonucleotides Used and Duplex Melting data^a^

a Nucleotides in black have 2′-*O*-Me ribose sugars and phosphorothioate internucleoside linkages. Nucleotides in red are locked nucleic acid phosphothiotriesters (except **ON17–ON24** which have phosphorothioate internucleoside linkages), the red ‘o’ indicates phosphotriester linkage instead of phosphothiotriester. ΔTm = difference in duplex melting temperature of **ON1–ON41** against DNA and RNA compared to control **ON44** (2′-*O*-methyl phosphorothioate). Tm of control vs DNA = 48.5 °C, Tm of control vs RNA = 61.3 °C. Melting temperatures were recorded in 10 mM Na-phosphate buffer, pH = 7.0. The melting buffer for complementary DNA contained additional 100 mM NaCl and the melting buffer for complementary RNA contained additional 25 mm NaCl. Tm values used for the ΔTm calculations are an average of three experiments with an error of ±0.25 °C. Comprehensive melting temperature data is in supporting information 4.0, Tables T6–T17. In some cases, the Tm against complementary RNA was too high to determine so additional Tm data was obtained in 10 mM Na-phosphate buffer, pH = 7.0 with no additional NaCl. These Tm values were adjusted using the following tool: http://biotools.nubic.northwestern.edu/OligoCalc.html. These ΔTm values are in red. See Supporting Information**4.8** for melting curves without additional NaCl.

**Figure 2:**
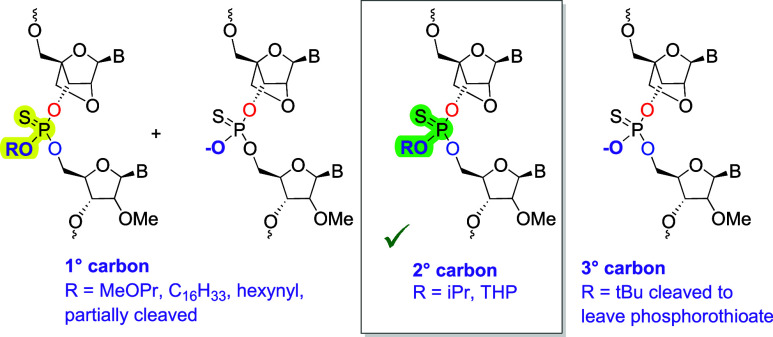
Synthesis of PTTE oligonucleotides using modified locked nucleic acid phosphoramidite monomers. Only secondary alkyl groups give efficient oligonucleotide synthesis. Details of possible side reactions leading to loss of triester alkyl groups are in the Supporting Information Figure S3.

The primary methoxypropyl alkyl groups in **ON1–ON8** were partially cleaved during deprotection with EDA-THF (Figure 2). Approximately 15% of undesired phosphodiester backbone oligonucleotide was obtained in the synthesis of **ON1** and **ON5** which contain a single addition of the MeOPr-A and MeOPr-T monomers respectively (Supporting Information Figures S5 and S21). Oligonucleotides with more than three methoxypropyl modifications (**ON7**, **ON8**) were difficult to purify and lower yields were obtained (Supporting Information Table T2). The same instability problem was encountered with ammonia deprotection of these oligonucleotides, even at room temperature (Supporting Information 2.1, 2.6). Formation of phosphodiester side products was not significant for **ON9–ON16** which contain from one to six isopropyl phosphothiotriesters, and good yields were obtained (Supporting Information Table T2). However, for oligonucleotides **ON17–ON24**, the *t*-butyl groups were cleaved from the triesters during solid-phase synthesis to give phosphorothioates, i.e. no PTTE linkages were found (Figure 2 and Supporting Information Tables T3 and T4). Cleavage of the *t*-butyl group was independent of the capping and detritylation steps and probably occurred during the oxidation or sulfurization step (Table T4 and Figures S66–S69). However, we cannot exclude loss of *t*-butyl during oligonucleotide deprotection. Potential mechanisms explaining the instability of primary and tertiary phosphothiotriester backbones are shown in the Figure S3. In contrast to the instability of 1^o^ and 3° triesters, oligonucleotides **ON25–ON32** containing 2° tetrahydropyranyl phosphothiotriesters (THP) were stable, and yields were similar to those of the isopropyl PTTE oligonucleotides (Supporting Information Table S2). We were able to successfully synthesize **ON33** and **ON34**, containing seven and nine THP triesters, respectively. In the latter case this reduces the negative charge in the oligonucleotide by more than 50%. Subsequently, we synthesized **ON35–ON38** containing one to three additions of the lipophilic C16-alkyl group which could influence cell uptake. As found for other primary alcohols, a mixture of phosphothiodiester and PTTE was obtained. Finally, we synthesized the hexynyl-functionalized PTTE oligonucleotides **ON39–ON41** for subsequent click labeling. In future we plan to use a secondary alcohol/alkyne to facilitate the incorporation of large numbers of alkynes. In summary, the nature of the alkyl group dictates synthesis efficiency of oligonucleotides with LNA-PTTE backbones, secondary alcohols being the best. Moreover, we were able to synthesize the oligonucleotides **ONOX1** and **ONOX4** which have one and four isopropyl phosphotriester linkages respectively (Table T5 and Figures S125–S128). To summarize, the stability of the LNA-secondary alkyl triesters is good in both phosphothiotriester and phosphotriester formats.

Small alkyl groups in the PTE backbone such as methyl, ethyl, isopropyl and tetrahydropyran-4-yl are reported to destabilize duplexes, causing a 1–4 °C reduction in melting temperature (Tm) relative to the unmodified oligonucleotide.^24^ In one case, however, a slight increase in duplex stability has been observed.^45^ Our strategy of combining LNA sugars with triester linkages ensured increased duplex stability against both complementary DNA and RNA in addition to the required chemical stability. A reduction in Tm was only observed for the C16 lipid chain linked oligonucleotides **ON35–ON38**. Stability is highest per modification for **ON17–ON24** but this is due to loss of the alkyl group from the triesters, leaving the LNA-phosphorothioate diester backbone linkages. This fortuitously provided LNA control oligonucleotides for the exon-skipping cell studies described below.

Overall, these results indicate that LNA-phosphothiotriester linkages are less duplex-stabilizing than LNA-phosphodiester linkages (compare **ON17** with **ON1**, **ON20** with **ON4**, **ON24** with **ON8**). The duplex destabilization caused by the alkyl groups on the PTTE backbone follows the order iPr < THP ∼ MeOPr < hexynyl < C16 (Table 1 and Supporting Information 4.0). In summary, adjacent LNA sugars increase the duplex stability of phosphothiotriester oligonucleotides compared to those with deoxyribose sugars, but the stabilizing effect is not quite as extreme as for LNA-phosphorothioate diesters. We also performed UV melting studies of **ON34** and **ON44** with complementary DNA at different salt concentrations. The results indicate that the melting temperature of **ON34** has slightly lower salt dependence compared to the control **ON44** due to the smaller number of negative charges in the backbone (Supporting Information 4.7).

**Scheme 2:**
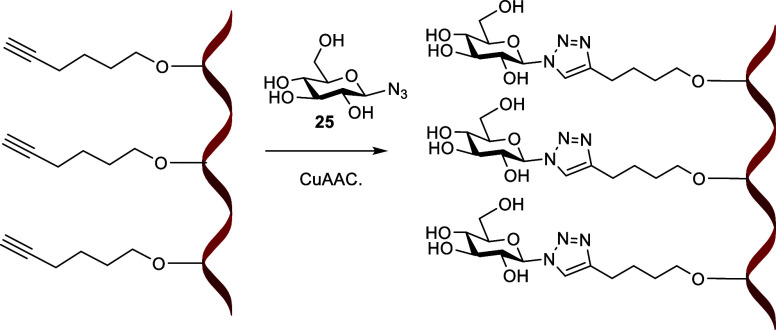
CuAAC Post-Labelling of Hexynyl 18-Mer **ON41** with Glucose Azide (**25**) Conditions: CuSO_4_, sodium ascorbate, H_2_O, DMSO, tris(3-hydroxypropyl-triazolylmethyl)amine (THPTA) 24 h at room temperature.

Hexynyl phosphothiotriester oligonucleotides **ON39–ON41** were functionalized with glucose azide by click chemistry to generate **ON47–ON49** corresponding to mono-, di- and trivalent glucose conjugates respectively (Scheme 2, Supporting Information 3.0). This represents a useful and versatile method of adding multiple internal reporter groups or other labels or cell-targeting moieties into the backbone of oligonucleotides.^57^

**Figure 3:**
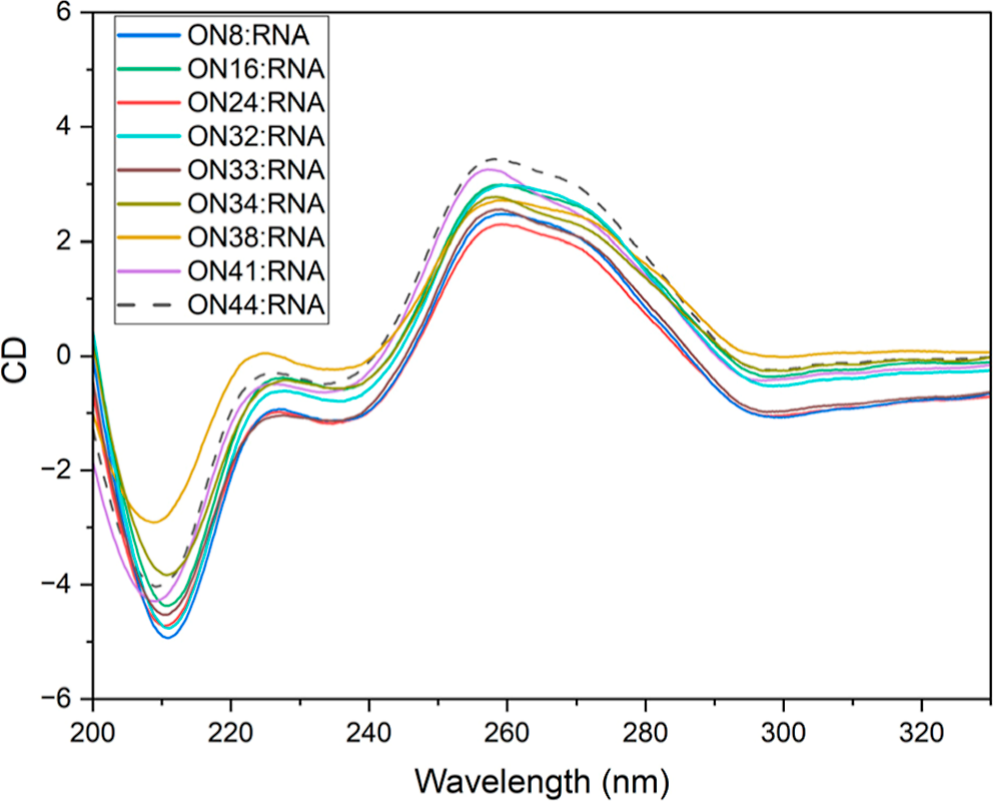
CD spectra of oligonucleotide-RNA duplexes. *Y*-axis is ellipticity θ, (10^–3^ deg.cm^2^/dmol).

Circular dichroism studies show that LNA-phosphothiotriesters have minimal effects on duplex structure, even when oligonucleotides contain large numbers of triesters. The CD spectra of oligonucleotides **ON8** (6 x MeOPr), **ON16** (6 x iPr), **ON32** (6 x THP), **ON33** (7 x THP), **ON34** (9x THP), **ON38** (3 x C16) and **ON41** (3 x hexynyl) hybridized to complementary RNA are almost perfectly aligned with the control **ON44**/RNA duplex (Figure 3). The highest structural deviation is observed for **ON38** which has the three lipophilic C16 alkyl groups. This could be due to intra- or intermolecular hydrophobic interactions between lipids, or changes in hydration of the duplex induced by the lipids. Either or both effects might also explain the lower duplex stability of these oligonucleotides. The PTTE backbones also cause minimal structural deviation of duplexes with complementary DNA (Supporting Information 5.0).

Stability of ASOs against nuclease enzymes in vivo is essential. To evaluate this, oligonucleotides **ON4** (MeOPr), **ON15** (iPr), **ON31** (THP), **ON35** (C16) and **ON41** (hexynyl), representing all alcohol variants studied, were incubated with nuclease S1 from *Aspergillus oryzae*. The unmodified control O**N46** was converted to mononucleotides within 1 h whereas oligonucleotides carrying a hydrophobic PTTE linkage remained fully intact after 2 days (Supporting Information 6.0). Moreover, we synthesized a series of oligonucleotides **ONS5**–**ONS8** based on (dT)_12_ in which one nucleotide is modified with LNA-PTE or LNA-PTTE (Supporting Information 6.1, Supporting Information Table T18 and Supporting Information Figures S177–S185). Enzymatic digestion of these oligonucleotides by exonuclease I (*Escherichia coli*) is blocked by the alkyl LNA PTTE and LNA PTE linkages, while phosphodiester linkages are completely digested by the enzyme within 1 hour. These experiments confirm that LNA-phosphotriester linkages are enzymatically stable, even in the absence of phosphorothioate groups. This offers the possibility of using oligonucleotides with reduced phosphorothioate content in vivo, thus potentially mediating interactions of therapeutic oligonucleotides with serum proteins and paraspeckle proteins such as P54nrb, which are reported to be undesirable.^58^

**Figure 4:**
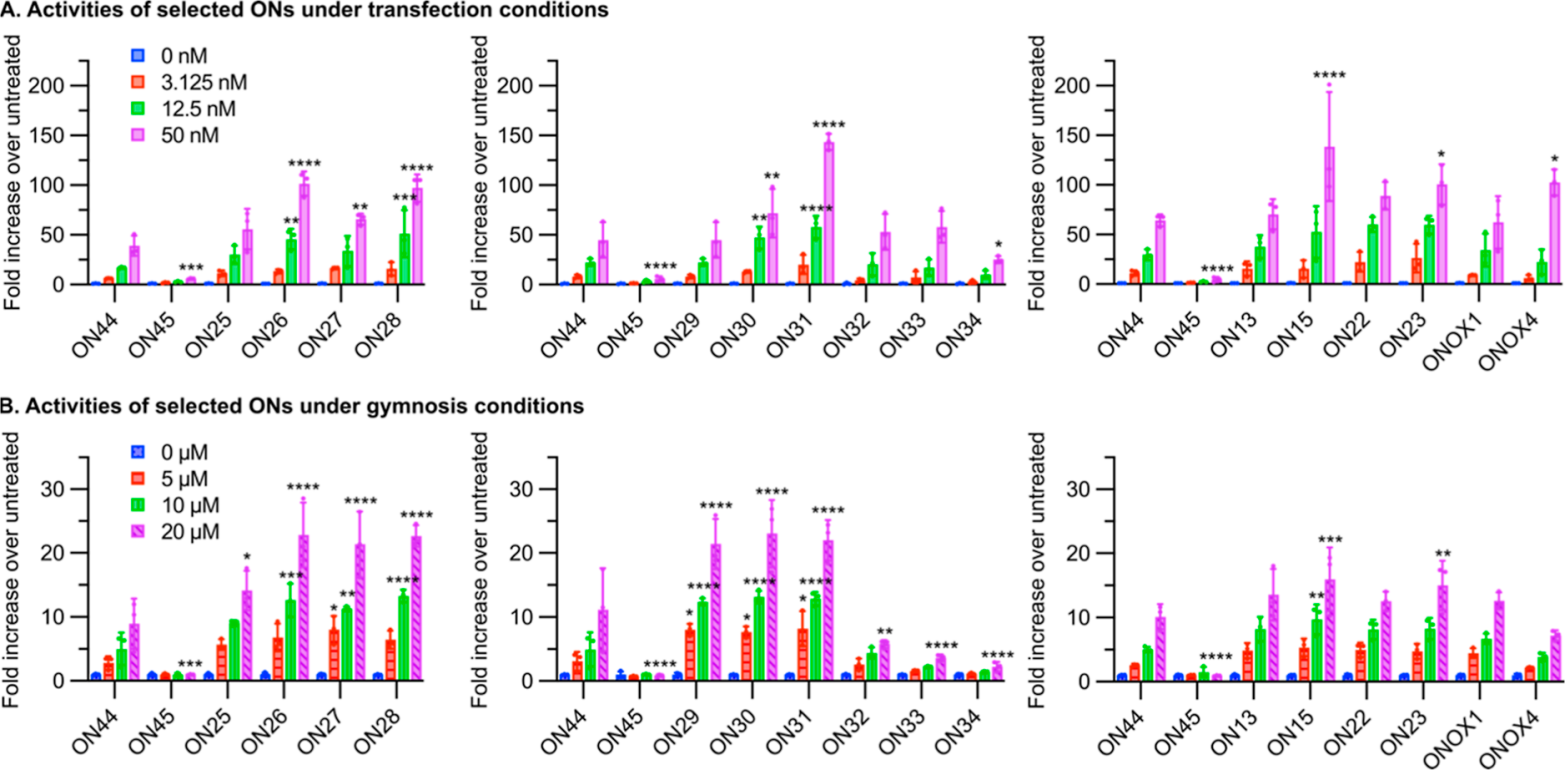
Activities of selected ONs in HeLa pLuc/705 cells. (A) ONs were transfected into HeLa pLuc/705 cells at the indicated concentrations using Lipofectamine 2000, and luciferase activity was measured 48 h later. (B) ONs were applied to HeLa pLuc/705 cells at the indicated concentrations in the absence of a transfection reagent, and luciferase activity was measured 72 h later. In all cases, luminescence was normalized to total protein quantity and untreated cells. Data are means ± standard deviations for three biological replicates (*n* = 3), where each biological replicate was performed in technical triplicate. Statistics are two-way analysis of variance (ANOVA) with Dunnett’s multiple comparisons test against **ON44**, α = 0.05: *P ≤ 0.05, **P ≤ 0.01, ***P ≤ 0.001, and ****P ≤ 0.0001. None of the oligonucleotides in this study displayed any significant toxicity as judged by cell growth (Supporting Information Figure S228).

In a preliminary study of the therapeutic potential of the LNA-phosphothiotriester backbone in the modulation of splicing, a series of 18-mer splice-switching oligonucleotides (SSOs) containing LNA-THP PTTE linkages (**ON25** to **ON34** in Table 1) were evaluated in a luciferase exon-skipping cell assay (Figure 4).^56^ These oligonucleotides contain between one and nine phosphothiotriesters and were designed to determine the relationship between activity and the number of LNA-PTTE linkages. We also evaluated oligonucleotides with varying LNA-phosphorothioate content (i.e. without triesters) (**ON22** and **ON23**). Finally, we compared the activity of oligonucleotides containing LNA-iPr-PTE and LNA-iPr-PTTE linkages (**ON13** vs **ONOX1** and **ON15** vs **ONOX4**). Our primary control oligonucleotide throughout was 2′-*O*-methyl phosphorothioate **ON44**. Exon-skipping activity was determined following both transfection and gymnosis in order to determine whether the origin of any increased performance was due to improved uptake into the cells or enhanced exon-skipping efficiency.

Under transfection conditions, **ON26**, **ON27**, and **ON28**, which contain two, two, and three LNA-THP PTTE linkages respectively at A-nucleotides, showed improved activity relative to the control **ON44**. The mean improvements were between 1.7 and 3.0-fold depending on oligonucleotide concentration. Similarly, **ON30** and **ON31**, which have two and four LNA-THP PTTE linkages at T-nucleotides, showed between 1.6 and 3.2-fold improved activity relative to **ON44**. Additionally, **ON15**, with four iPr-PTTE linkages at T-nucleotides, showed 2.2-fold increased activity at 50 nM relative to **ON44**.

Under gymnosis conditions, **ON25**, **ON26**, **ON27**, and **ON28**, which have one, two, two, and three LNA-THP PTTE linkages respectively at A-nucleotides, all showed enhanced activity relative to **ON44**. The mean improvements ranged from 1.6 to 2.6-fold at 20 μM. **ON29**, **ON30**, and **ON31**, which have one, two, and four LNA-THP PTTE linkages at T-nucleotides, showed similar levels of improved activity relative to **ON44**. **ON15**, which has four iPr PTTE linkages at T-nucleotides also showed improved activity at both 10 and 20 μM, where the mean improvements were 1.9 and 3.2-fold, respectively. In contrast, oligonucleotides with greater numbers of LNA-neutral linkages were less active: **ON32**, which has six LNA-THP PTTE linkages, and **ON33** and **ON34**, which have seven and nine LNA-THP PTTEs respectively, showed greatly reduced activities.

**Figure 5:**
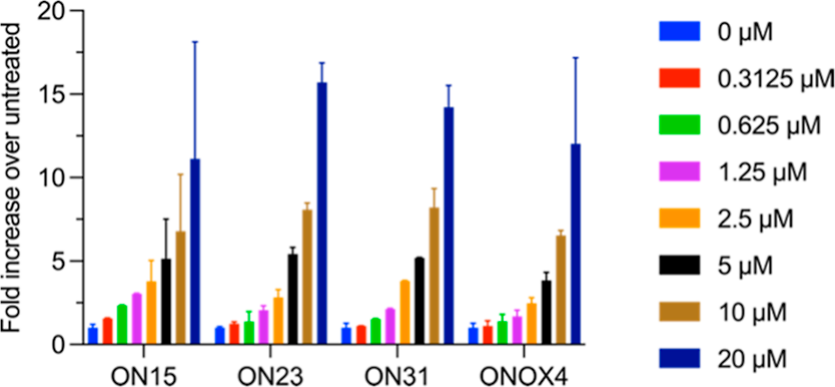
Seven-point dose response of selected ONs in HeLa pLuc/705 cells. ONs were applied to HeLa pLuc/705 cells at the indicated concentrations in the absence of a transfection reagent, and luciferase activity was measured 72 h later. In all cases, luminescence was normalized to total protein quantity and untreated cells. Data are means ± standard deviations for two biological replicates (*n* = 2), where each biological replicate was performed in technical triplicate.

Comparing the activities of **ON15** (4 x iPr), **ON23** (4 x LNA phosphorothioate control), **ON31** (4 x THP-PTTE) and **ONOX4** (4 x iPr-PTE) under gymnosis conditions at seven concentrations on a single plate indicates that the LNA phosphothiotriester and phosphotriester modifications, as well as the LNA phosphorothioate control, all have similar activity (Figure 5). Importantly, both **ON15** and **ONOX4** which contain four LNA-iPr PTTE and PTE respectively, and therefore differ in the number of sulfur atoms in the oligonucleotide backbone, have comparable splice-switching activities. This is in line with their similar duplex stabilities with complementary RNA (Table 1) and the stability of the LNA-PTE linkage to enzymatic digestion discussed above. The use of LNA-PTE linkages in therapeutic oligonucleotides could facilitate alternative delivery mechanisms that do not depend on binding of phosphorothioates to serum proteins.^59^ Moderating the number of phosphorothioates has been suggested as a method to prevent excessive oligonucleotide-protein binding in vivo,^58^ and reducing PS content has been shown to improve the toxicity profile and acute tolerability of ASOs in vivo.^60^

Our study shows that oligonucleotides which have moderately but not *excessively* high melting temperatures relative to the control **ON44** have improved splice-switching activities. Release of the oligonucleotide from the spliced-out intron will allow interactions with more pre-mRNA molecules, and this could explain why oligonucleotides with large numbers of LNA sugars (i.e., with high Tms) have decreased splice-switching activities. On the other hand, if the oligonucleotide/RNA duplex is too *unstable* it will not block aberrant splicing, so “low-Tm” oligonucleotides will be inactive. In this study the optimum Tm against complementary RNA is ∼8 °C above that of the control **ON44** (Supporting Information Figure S229). Other mechanisms/factors may be involved and these need to be investigated in future.

## Conclusions

To conclude, we have synthesized oligonucleotides containing multiple charge-neutral LNA-phosphothiotriester linkages by straightforward solid phase phosphoramidite methods, inserting methoxypropyl, isopropyl, THP, C16 lipid, and hexynyl into the PTTE backbone. We show that it is critically important to select appropriate alkyl functionalities, with secondary alkyl groups being the most suitable. To demonstrate the efficiency of our methodology we have introduced nine charge-neutral THP-PTTE linkages into a modified 18-mer oligonucleotide, reducing overall negative charge by more than 50%. The LNA-PTTE backbones stabilize duplexes with complementary DNA or RNA and do not distort their structures. With in vivo applications in mind, 2′-*O*-methyl phosphorothioate oligonucleotides containing LNA-PTTEs and LNA-PTEs are stable in the presence of nuclease enzymes. Our approach is applicable to the incorporation of multiple alkynes across the oligonucleotide backbone, enabling conjugation with azide derivatives, demonstrated here by glucose. This post-labeling click strategy has potential for the incorporation of other carbohydrate and peptide-based cell-receptor ligands.^61−63^ We used final stage intermediates of LNA phosphoramidites as starting materials to make the modified LNA phosphoramidites. This is a strength of our approach, as such intermediates are available from companies that produce special phosphoramidite monomers for the synthesis of therapeutic oligonucleotides. It also means that similar work could readily be carried out on other therapeutically relevant nucleoside phosphoramidite precursors including protected 2′-*O*-alkyl and 2′-fluoro nucleosides which are manufactured on an industrial scale. Importantly the nucleobase protecting groups on these nucleosides are compatible with the deprotection conditions used for PTTE triester oligonucleotides bearing secondary alcohols. Initial cell studies show a large increase in exon-skipping activity for oligonucleotides containing between two and four LNA-THP phosphothiotriester modifications whereas oligonucleotides with greater numbers of LNA-phosphothiotriesters were much less active. The fact that increased activity was observed in both transfection and gymnotic delivery suggests improved steric blocking of the enzymatic cleavage that is required for mRNA splicing. Toxicity has been an obstacle to the use of LNA oligonucleotides clinically.^64,65^ It will therefore be important to study the toxicological and pharmacokinetic properties of oligonucleotides containing LNA-phospho(thio)triesters in depth, as they might have more favorable toxicological properties than the equivalent well-studied LNA-diesters. Protein-binding, cell and animal studies will be carried out to investigate this.

As is the case for all clinically approved oligonucleotides containing phosphorothioates, our LNA-phospho(thio)triesters are diastereomeric mixtures at phosphorus. Sterically pure phosphorothioates can be synthesized by P(V) and P(III) chemistry but have not yet reached the clinic.^66,67^ Synthetic efforts to produce chirally pure LNA-PTTE/PTE oligonucleotides are worth considering, and might lead to improved properties. Finally, applications of the oligonucleotides reported here may stretch beyond therapeutics, for example into several fields such as triplexes and modified aptamers where flexibility of functionality and in vivo stability are important considerations.^68^

## Supporting Information Available

Supplementary Tables S1–S5. Figures S1–S229. Experimental procedures, analytical data of novel compounds including 1D (^1^H, ^13^C, ^31^P) NMR spectra and HRMS. UPLC-ESI-MS of oligonucleotides. (PDF). UV meting data, CD curves, PAGE analysis, trityl reading, micrographs of Hela pLuc/705 cells, etc (PDF)
