# Supplementary material for: Synthesis, Biophysical and Biological Evaluation of Splice-Switching Oligonucleotides with Multiple LNA-Phosphothiotriester Backbones
Source: J Am Chem Soc. 2024 Oct 14;146(43):29773–81. doi: 10.1021/jacs.4c11402 (PMC11528411; doi:10.1021/jacs.4c11402)
Supplement: Supplementary file 1 — ja4c11402_si_001.pdf [file ja4c11402_si_001.pdf]

Supporting Information

Synthesis, Biophysical and Biological Evaluation of Splice-Switching Oligonucleotides with Multiple LNA-Phosphothiotriester Backbones

Debashis Dhara[1], Alyssa C. Hill[2], Abinaya Ramesh[2], Matthew J.A. Wood[2], Afaf H. El-Sagheer[1,3] and Tom Brown[1]*

[1]Department of Chemistry, University of Oxford, Chemistry Research Laboratory, 12 Mansfield Road, Oxford OX1 3TA, United Kingdom

[2]Department of Paediatrics, Institute of Developmental and Regenerative Medicine (IDRM), University of Oxford, Oxford OX3 7TY, United Kingdom

[3]School of Chemistry, University of Southampton, Highfield, Southampton, SO17 1BJ, United Kingdom.

Contents

1.0 Chemical synthesis ... 3
1.1 General experimental procedure ... 3
1.2 Experimental procedure ... 4
2.0 Oligonucleotide synthesis and UPLC-MS of oligonucleotides ... 13
2.1 MeOPr PTTE oligonucleotides ... 25
2.2 ^{i}Pr PTTE oligonucleotides ... 40
2.3 LNA phosphorothiaote oligonucleotides ... 48
2.3.1 Cleavage of tert-butyl group during oxidation step: ... 56
2.4 THP PTTE oligonucleotides ... 59
2.5 C16 PTTE oligonucleotides ... 79
2.6 Hexynyl PTTE oligonucleotides ... 83
2.7 Phosphotriester (PTE) and phosphothiotriester (PTTE) ... 89
2.8 Complementary DNA and RNA and control oligonucleotide ... 92
3.0 Click reaction and UPLC-MS of oligonucleotide conjugates ... 95
4.0 UV meting experiments ... 99
4.4 MeOPr PTTE oligonucleotides ... 100
4.2 iPr PTTE oligonucleotides ... 102
4.3 LNA phosphorothiaote oligonucleotides ... 104
4.4 THP PTTE oligonucleotides ... 106
4.5 C16 PTTE oligonucleotides ... 108
4.6 Hexyn PTTE oligonucleotides ... 109
4.7 UV melting study at different salt concentration ... 110
4.8 UV melting experiments without NaCl salt ... 111
5.0 Circular dichroism (CD) ... 112
5.1 CD spectra of oligonucleotide:RNA duplex ... 113
5.2 CD spectra of oligonucleotide:DNA duplex ... 115
6.0 Enzymatic stability of selected oligonucleotide ... 120
6.1 Enzymatic digestion study with model oligonucleotides ... 123
7.0 Trityl monitor readings ... 128
8.0 NMR spectra of novel compounds ... 134
9.0 Biological experiments, materials and methods ... 159
10.0 References ... 163

1.0 Chemical synthesis

1.1 General experimental procedure

Anhydrous (Anhyd.) solvents – including dichloromethane (DCM), diethyl ether (Et_2O) pentane, acetonitrile (MeCN), triethylamine (Et_3N), were collected from an mBraun SPS-800 bench top solvent purification system, having passed through anhydrous alumina columns. Solvents for phosphitylation reaction were degassed by bubbling argon through before use. Solvents for purification such as hexane (Hex) and ethyl acetate (EtOAc) were degassed by bubbling argon through and mixed with 1% triethylamine. Reactions requiring anhydrous conditions were run under an Ar atmosphere, using oven-dried glassware which was allowed to attain room temperature whilst flowing dry argon through. Freshly activated molecular sieves (MS 4Å) were prepared before use by heating with a heat gun (20-30 minutes) under high vacuum. Thin layer chromatography (TLC) was performed using Merck pre-coated 0.23 mm thick plates (Kieselgel 60 F254) and visualised using UV light (λ = 254 nm). Flash column chromatography was carried out using silica gel (60 µm particle size). ^{1}H, ^{13}C and ^{31}P NMR spectra were recorded on a Bruker AVIIIHD 400 or Bruker NEO 600 (with broadband helium cryoprobe) spectrometer operating at 400 or 600 MHz respectively using an internal deuterium lock at ambient probe temperatures. ^{1}H NMR chemical shifts (δ) are quoted to the nearest 0.01 ppm and are referenced relative to residual solvent peaks. Spectra were recorded in $CDCl_3$. Chemical shifts are reported in ppm (δ) relative to residual solvent peaks, $CHCl_3$ in the case of $CDCl_3$ at 7.28/77.0 ppm for the ^{1}H and ^{13}C spectra, respectively. Coupling constants are reported in Hertz (Hz). Elucidation of chemical structures is based on ^{1}H, COSY, ^{1}H decoupled ^{13}C, DEPT-135, HSQC, and HMBC. Signals are reported as s (singlet), d (doublet), t (triplet), dd (doublet of doublet), q (quartet), dt (doublet of triplet), dq (doublet of quarlet), ddd (doublet of doublet of doublet), m (multiplet). Mass Spectroscopy: High-resolution mass spectra (HRMS) were recorded by the Chemistry Department Mass Spec. Service, University of Oxford on a Thermo Scientific Exactive Mass Spectrometer (Waters Equity autosampler and pump) - electrospray ionization (ESI) and an Agilent 7200 Accurate Mass QTOF GCMS (SIM Direct Insertion Probe) for electron ionization (EI) and chemical ionization (CI).

1.2 Experimental procedure

***N,N,N',N'*-tetraisopropyl-1-(3-methoxypropoxy)phosphanediamine (7):** To a suspension of bis(diisopropylamino)chlorophosphine (1.0 g, 3.784 mmol, 1.0 equiv.) in anhydrous Et_2O (10.0 mL) was added triethylamine (1.15 mL, 8.246 mmol, 2.2 equiv.) followed by the addition of 3-methoxy-1-propanol **1** (394 µL, 4.123 mmol, 1.1 equiv.) slowly dropwise by a syringe. The reaction was left to stirred for 2h at rt under an atmosphere of argon. The solution was filtered from the triethylamine salt by cannula filtration. Solids were washed with degassed, anhydrous Et_2O (2 x 10 mL). The combined filtrate was concentrated at first under reduced pressure and then left under high vacuum for 30 mins. The remaining liquid was diluted with 10 mL of MeCN and 15 mL of pentane. The biphasic mixture was transferred to a separating funnel. The pentane part was collected and dried to give the desired product **7** as a colourless oil (crude weight, 900 mg, 2.81 mmol, 75%). The crude product **7** had **^{31}P NMR** (162 MHz, $CDCl_3$) δ 124.12. **HRMS (ESI$^+$):** *m/z* $[M+H]^+$ calc. for $C_{16}H_{38}N_2O_2P$ 321.2665; found 321.2665.

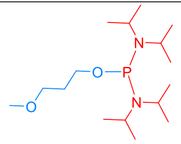
Chemical Formula: $C_{16}H_{37}N_2O_2P$
Exact Mass: 320.26
7

1-Isopropoxy-*N,N,N',N'*-tetraisopropylphosphanediamine (8): *Procedure 1:* To a suspension of bis(diisopropylamino)chlorophosphine (1.0 g, 3.78 mmol, 1.0 equiv.) in anhydrous Et_2O (10.0 mL) was added triethylamine (1.57 ml, 11.24 mmol, 3.0 equiv.). Isopropanol (0.574 ml, 7.49 mmol, 2.0 equiv.) was then added slowly dropwise by a syringe. The reaction mixture was left to stir vigorously at rt for 2 hrs under an atmosphere of argon. The solution was filtered from the triethylamine salt by cannula filtration. Solids were washed with anhydrous Et_2O (2 x 10 mL). The combined filtrate (~30 mL) was concentrated under reduced pressure. The crude product was dried under high vacuum for 30 minutes. To the crude material was added 10 mL of anhydrous MeCN and 10 mL of pentane. The pentane part was collected and concentrated to give the desired product **8** as a colourless oil (crude weight, 800 mg, 2.75 mmol, 73%). The analytical data were consistent with the previously reported data.[1]

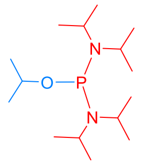
Chemical Formula: $C_{15}H_{35}N_2OP$
Exact Mass: 290.25
8

1-Tert-butyloxy-*N,N,N',N'*-tetraisopropylphosphanediamine (9): To a suspension of bis(diisopropylamino)chlorophosphine (850 mg, 3.19 mmol, 1.0 equiv.) in anhydrous Et_2O (10.0 mL) was added triethylamine (888 µL, 6.37 mmol, 2.0 equiv.). Tert-butanol (472 mg, 6.37 mmol, 2.0 equiv.) was then added. The reaction mixture was left to stir vigorously at rt for 2 hrs under an atmosphere of argon. The solution was filtered from the triethylamine salt by cannula filtration. Solids were washed with anhydrous Et_2O (2 x 10 mL). The combined filtrate (~30 mL) was concentrated under reduced pressure. The crude product was dried under high vacuum for 30 minutes. To the crude material was added 15 mL of anhydrous MeCN and 10 mL of pentane. The pentane part was collected and washed again anhydrous MeCN (10 mL) then concentrated to give the desired crude

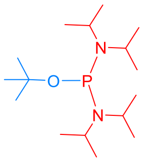
Chemical Formula: $C_{16}H_{37}N_2OP$
Exact Mass: 304.26
9

product **9** as a white solid (crude weight, 750 mg, 3.91 mol, 77%). The analytical data were consistent with the previously reported data.[2]

***N,N,N',N'*-tetraisopropyl-1-((tetrahydro-2H-pyran-4-yl)oxy)phosphanediamine (10):** To a suspension of bis(diisopropylamino)chlorophosphine (1.5 g, 5.62 mmol, 1.0 equiv.) in dry, degassed Et_2O (20.0 mL) was added dry, degassed triethylamine (2.35 mL, 16.87 mmol, 3.0 equiv.) followed by the addition of tetrahydro-4-pyranol **4** (0.804 mL, 8.43 mmol, 1.5 equiv.). The reaction was left to stir for 3h under an atmosphere of argon. The solution was then filtered from the triethylamine salt by cannula filtration. Solids were washed with Et_2O (2 x 10 mL). The combined filtrate was concentrated under reduced pressure and the crude product was dried under high vacuum for 1 hr. To the crude material was added anhydrous MeCN (15 mL) and dry pentane (15 mL). The biphasic mixture was transferred to a separatory funnel and the pentane part was collected and concentrated on a rotary evaporator to give the desired product as a white solid (crude weight, 1.7 g, 5.62 mmol, 68%). The crude phosphine reagent **10** had **^{31}P NMR** (162 MHz, $CDCl_3$) δ 112.31. **HRMS (ESI$^+$):** *m/z* $[M+H]^+$ calc. for $C_{17}H_{38}N_2O_2P$ 333.2665; found 333.2707.

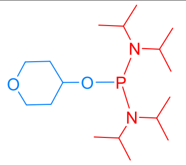

Chemical Formula: $C_{17}H_{37}N_2O_2P$
Exact Mass: 332.26
10

1-(Hexadecyloxy)-*N,N,N',N'*-tetraisopropylphosphanediamine (11): To a suspension of bis(diisopropylamino)chlorophosphine (1.0 g, 3.784 mmol, 1.0 equiv.) in anhydrous, degassed Et_2O (10.0 mL) was added anhydrous triethylamine (1.57 mL, 11.24 mmol, 3.0 equiv.). Then 1-hexadecanol **5** (908 mg, 3.784 mmol, 1.0 equiv.) was then added. The reaction was left to stir for 6h at rt under an atmosphere of argon. The solution was then filtered from the triethylamine salt by cannula filtration. Solids were washed with anhydrous Et_2O (2 x 15 mL). The combined filtrate was concentrated under reduced pressure and the crude product was dried under high vacuum for 1 hour. To the crude material was added anhydrous MeCN (15 mL) and dry pentane (15 mL). The biphasic mixture was transferred to a separatory funnel and the pentane part was collected and concentrated to give the desired product **11** as a white solid (crude weight, 1500 mg, 3.17 mmol, 84%). **^{31}P NMR** (162 MHz, $CDCl_3$) δ 124.21. **HRMS (ESI$^+$):** *m/z* $[M+H]^+$ calc. for $C_{28}H_{62}N_2OP$ 473.4594; found 473.4606.

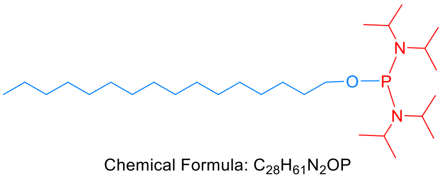

Chemical Formula: $C_{28}H_{61}N_2OP$
Exact Mass: 472.45
11

1-(Hex-5-yn-1-yloxy)-*N,N,N',N'*-tetraisopropylphosphanediamine (12): To a suspension of bis(diisopropylamino)chlorophosphine (1.0 g, 3.74 mmol, 1.0 equiv.) in anhydrous Et_2O (10.0 mL) was added dry triethylamine (1.57 mL, 11.24 mmol, 3.0 equiv.). Afterwards, 5-hexyn-1-ol **6** (0.5 mL, 4.49 mmol, 1.2 equiv.) was added. The reaction was left to stir for 4h at rt under Argon. At completion of the reaction, the solution was filtered from the triethylamine salt by cannula filtration. Solids was washed with Et_2O (2 x 15 mL). The combined filtrate was concentrated under reduced pressure and the crude product was left to dry over high vacuum for 2 hours. To the

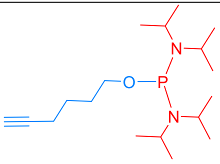

Chemical Formula: $C_{18}H_{37}N_2OP$
Exact Mass: 328.26
12

crude material was added 10 mL of MeCN, the suspension was transferred to a separatory funnel and dry pentane (15 mL) was added. The MeCN part was discarded and the pentane part was washed again with dry MeCN (10 mL). The pentane part was collected and concentrated to give the desired product **12** as a colourless oil (crude weight, 1100 mg, 3.34 mmol, 89%). The crude **12** had **^{31}P NMR** (162 MHz, $CDCl_3$) δ 123.74. **HRMS (ESI$^+$):** *m/z* $[M+H]^+$ calc. for $C_{18}H_{38}N_2OP$ 329.2717; found 329.2716.

5′-*O*-(4,4′-dimethoxytrityl)-2′-*O*,4′-*C*-methylenethymidine-3′-*O*-[(*O*-(3-methoxypropyl)-*N,N*′-diisopropylphosphoramidite] (14): Locked nucleic acid monomer **13** (400 mg, 699 µmol, 1.0 equiv.) was dissolved in dry, degassed DCM (10 ml). Phosphitylating reagent **7** (448 mg, 1.40 mmol, 2.0 equiv.) was added followed by the addition of tetrazole (~0.45 M in MeCN, 1.39 mL, 699 µmol, 1.0 equiv.). The reaction mixture was left to stir at rt for 16h under an atmosphere of argon. At completion, the reaction was diluted with 10 mL of DCM and washed with saturated KCl solution (10 mL). The DCM part was dried over anhydrous $MgSO_4$, filtered and concentrated. The crude residue was purified by flash chromatography (60:40→50:50 Hex:EtOAc, both solvent were mixed with 1% triethylamine) to give the desired phosphoramidite **14** (420 mg, 530 µmol, 75%) as a white solid. The phosphoramidite **14** had R_f 0.43,0.53 (Hex:EtOAc, 1:1). **^{1}H NMR** (400 MHz, $CDCl_3$, mixture of R/S-isomers) δ 8.83 (s, 2H), 7.63 (dd, J = 6.9, 1.4 Hz, 2H), 7.44 – 7.35 (m, 4H), 7.27 (dd, J = 4.6, 2.9 Hz, 4H), 7.27 – 7.13 (m, 5H), 6.82 – 6.72 (m, 7H), 5.59 (dd, J = 2.0, 0.7 Hz, 2H), 4.47 (d, J = 14.8 Hz, 2H), 4.29 (d, J = 8.9 Hz, 1H), 4.18 (d, J = 6.6 Hz, 1H), 3.80 (dd, J = 7.7, 2.4 Hz, 2H), 3.72 (t, J = 1.0 Hz, 13H), 3.69 – 3.23 (m, 14H), 3.21 (s, 3H), 3.17 (s, 2H), 1.68 (dd, J = 36.7, 6.2 Hz, 4H), 1.53 (dd, J = 12.9, 1.2 Hz, 5H), 1.11 – 0.97 (m, 16H), 0.93 (s, 3H). **^{13}C NMR (**101 MHz, $CDCl_3$**,** ^{1}H decoupled**,** mixture of R/S-isomer**)** δ 163.79, 163.77, 158.61, 149.61, 144.27, 144.22, 135.36, 135.30, 135.24, 135.20, 134.60, 134.57, 130.18, 130.12, 130.05, 128.21, 128.12, 127.87, 127.02, 127.00, 113.16, 113.13, 113.12, 110.21, 110.18, 88.00, 87.96, 87.90, 87.84, 87.39, 87.36, 86.57, 78.68, 78.05, 77.28, 76.96, 76.64, 72.21, 72.08, 71.09, 70.96, 70.45, 70.30, 69.04, 69.01, 68.70, 60.71, 60.54, 60.18, 60.01, 58.49, 58.44, 58.30, 58.04, 55.12, 43.10, 42.98, 24.56, 24.47, 24.45, 24.41, 24.38, 24.34, 24.31, 24.24, 12.38, 12.35. **^{31}P NMR** (162 MHz, $CDCl_3$) δ 147.89, 147.82. **HRMS (ESI$^+$):** *m/z* $[M+H]^+$ calc. for $C_{42}H_{55}N_3O_{10}P$ 792.3620; found 792.3588.

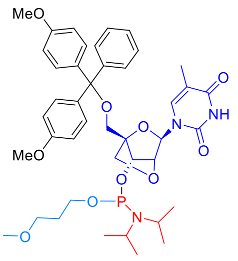

Chemical Formula: $C_{42}H_{54}N_3O_{10}P$
Exact Mass: 791.35
14

5′-*O*-(4,4′-dimethoxytrityl)-2′-*O*,4′-*C*-methylenethymidine-3′-*O*-[(*O*-(*iso*-propyl)-*N,N*′-diisopropylphosphoramidite] (15): Nucleic acid monomer **13** (300 mg, 524 µmol, 1.0 equiv.) was dissolved in dry, degassed DCM (10 mL). Tetrazole (~0.45M in MeCN, 1.16 mL, 524 µmol, 1.0 equiv.) and phosphitylating reagent **8** (304 mg, 1.048 mmol, 2.0 equiv.) were added. The reaction was left to stir at rt for 24 h under an argon atmosphere. At completion, the reaction was diluted with dry DCM (10 mL) and washed with saturated KCl solution (10 mL). The DCM part was dried over Na_2SO_4 and then concentrated under reduced pressure. The residue was purified by flash chromatography (50:50 Hex:EOAc, mixed with 1% triethylamine) to

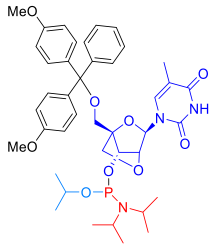

Chemical Formula: $C_{41}H_{52}N_3O_9P$
Exact Mass: 761.34
15

give the desired phosphoramidite **15** (260 mg, 341 µmol, 65%) as a white solid. The phosphoramidite **15** had R_f 0.62 (1:1 Hex:EtOAc, mixed with 0.5% triethylamine). **^{1}H NMR** (400 MHz, $CDCl_3$, mixture of R/S-isomers) δ 8.54 (s, 2H), 7.67 – 7.57 (m, 2H), 7.40 (qt, *J* = 7.0, 3.7 Hz, 4H), 7.33 – 7.14 (m, 11H), 6.77 (ddp, *J* = 7.3, 4.9, 2.3 Hz, 7H), 6.13 – 6.05 (m, 0H), 5.58 (dd, *J* = 5.4, 3.0 Hz, 2H), 4.64 – 4.40 (m, 2H), 4.23 (dt, *J* = 9.3, 2.4 Hz, 1H), 4.12 (dd, *J* = 7.2, 2.3 Hz, 1H), 4.09 – 3.92 (m, 1H), 3.86 – 3.67 (m, 15H), 3.52 – 3.32 (m, 8H), 1.56 (s, *J* = 1.1 Hz, 2H), 1.51 (s, 1H), 1.30 – 1.23 (m, 5H), 1.22 – 1.15 (m, 12H), 1.06 (ddd, *J* = 6.2, 4.6, 2.3 Hz, 14H), 0.98 (dd, *J* = 6.8, 2.0 Hz, 4H). **^{13}C NMR** (101 MHz, $CDCl_3$, ^{1}H decoupled**,** mixture of R/S-isomer) δ 163.61, 158.61, 149.51, 144.35, 144.27, 135.36, 135.24, 135.20, 134.62, 130.22, 130.16, 130.12, 130.06, 128.24, 128.10, 127.87, 127.01, 126.97, 116.37, 113.17, 113.14, 113.11, 110.08, 110.01, 87.85, 87.37, 86.56, 78.76, 77.36, 77.04, 76.72, 72.27, 72.21, 70.84, 68.64, 68.59, 67.21, 67.10, 67.00, 66.89, 58.25, 55.14, 45.07, 45.01, 43.09, 43.02, 42.97, 42.90, 24.58, 24.50, 24.43, 24.36, 24.30, 24.18, 24.11, 23.97, 23.92, 22.89, 22.87, 22.74, 22.72, 12.37, 12.30. **^{31}P NMR** (162 MHz, $CDCl_3$) δ 146.4, 146.2. **HRMS (ESI$^+$):** *m/z* $[M+H]^+$ calc. for $C_{41}H_{53}N_3O_9P$ 762.3514; found 762.3504.

5′-*O*-(4,4′-dimethoxytrityl)-2′-*O*,4′-*C*-methylenethymidine-3′-*O*-[(*O*-(*tert*-butyl)-*N,N*′-diisopropylphosphoramidite] (16): To a solution of locked nucleic acid monomer **13** (300 mg, 524 µmol, 1.0 equiv.) in dry, degassed DCM (10 mL) was added 1-tert-butoxy-*N,N,N′,N′*-tetraisopropylphosphanediamine **9** (319 mg, 1.05 mmol, 2.0 equiv.). Tetrazole (~0.45M in MeCN, 1.16 mL 524 µmol, 1.0 equiv.) was added slowly by a syringe. The reaction mixture was left to stir at rt for 16h under an atmosphere of argon. At completion, the reaction was diluted with DCM (10 mL). The DCM part was washed with saturated KCl solution (10 mL). The DCM part was collected and passed though $MgSO_4$ and then concentrated under reduced pressure. All the work-up were performed under an atmosphere of argon. The crude product was purified by flash chromatography (50:50 Hex:EtOAc, mixed with 1% triethylamine) to give the DMT-LNA-T- phosphoramidite **16** (245 mg, 316 µmol, 60%) as a white solid. The **16** had R_f 0.45 (1:1 Hex:EtOAc, mixed with 1% triethylamine). **^{1}H NMR** (600 MHz, $CDCl_3$, mixture of R/S-isomers) δ 7.74 (t, *J* = 1.6 Hz, 2H), 7.53 – 7.44 (m, 4H), 7.41 – 7.28 (m, 12H), 7.30 – 7.22 (m, 2H), 6.91 – 6.82 (m, 9H), 5.68 (dd, *J* = 11.9, 0.7 Hz, 2H), 4.58 (d, *J* = 17.8 Hz, 2H), 4.26 (d, *J* = 9.7 Hz, 1H), 4.15 (d, *J* = 7.0 Hz, 1H), 3.93 – 3.84 (m, 2H), 3.86 – 3.78 (m, 15H), 3.59 (dt, *J* = 10.6, 3.3 Hz, 2H), 3.59 – 3.49 (m, 2H), 3.46 (dd, *J* = 10.8, 6.3 Hz, 2H), 1.65 (d, *J* = 1.3 Hz, 3H), 1.61 (d, *J* = 1.2 Hz, 2H), 1.30 (s, 9H), 1.20 (s, 9H), 1.16 (dd, *J* = 12.2, 6.8 Hz, 11H), 1.05 (d, *J* = 6.8 Hz, 5H), 1.02 (d, *J* = 6.8 Hz, 6H), 0.90 (t, *J* = 7.1 Hz, 1H). **^{13}C NMR** (151 MHz, $CDCl_3$, ^{1}H decoupled**,** mixture of R/S-isomer) δ 162.70, 157.68, 157.66, 157.64, 148.53, 143.42, 143.33, 134.46, 134.41, 134.33, 134.29, 133.77, 129.29, 129.23, 129.19, 129.14, 127.32, 127.19, 126.93, 126.06, 126.02, 112.35, 112.33, 112.23, 112.20, 109.12, 109.04, 87.05, 87.02, 86.94, 86.90, 86.49, 86.47, 85.65, 85.60, 77.79, 77.77, 77.11, 77.09, 76.22, 76.01, 75.80, 74.34, 74.32, 74.27, 74.25, 71.41, 71.32, 69.50, 69.44, 68.93, 68.85, 57.46, 57.32, 54.23, 54.19, 42.20, 42.16, 42.11, 42.07, 29.79, 29.74, 29.69, 29.64, 23.60, 23.55, 23.44, 23.39, 23.11, 23.06, 22.97, 22.93, 11.42, 11.39. **^{31}P NMR** (162 MHz, $CDCl_3$) δ 140.46, 139.26. **HRMS (ESI$^+$):** *m/z* $[M+H]^+$ calc. for $C_{42}H_{55}N_3O_9P$ 776.3671; found 776.3606.

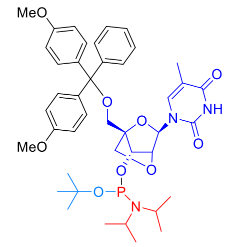

Chemical Formula: $C_{42}H_{54}N_3O_9P$
Exact Mass: 775.36

16

5′-*O*-(4,4′-dimethoxytrityl)-2′-*O*,4′-*C*-methylenethymidine-3′-*O*-[(*O*-(tetrahydro-2H-pyran-4-yl)-*N*,*N*′-diisopropylphosphoramidite] (17): Locked nucleic acid monomer **13** (300 mg, 524 µmol, 1.0 equiv.) was dissolved in dry, degassed DCM (10 mL). Tetrazole (0.45M in MeCN, 1.16 mL, 1.0 equiv.) and crude phosphitylating reagent **10** (348 mg, 1.05 mmol, 2.0 equiv.) were added. The reaction mixture was left to stir at rt for 16h under an atmosphere of argon. At completion, the reaction was diluted with dry, degassed DCM (10 mL). The reaction mixture was washed with saturated KCl solution (10 mL). The DCM part was passed though $MgSO_4$ and then concentrated in a rotary evaporator. The crude product was purified by flash chromatography (50:50 Hex:EtOAc, mixed with 1% triethylamine) to give the DMT-LNA-T-THP phosphoramidite **17** (255 mg, 317 µmol, 60%) as a white solid. The phosphoramidite **17** had R_f 0.31 (1:1, Hex:EtOAc, mix with 1% Et_3N). **^{1}H NMR** (400 MHz, $CDCl_3$, mixture of R/S-isomer) δ 7.75 (s, 1H), 7.68 (dd, *J* = 3.8, 1.5 Hz, 2H), 7.44 (ddq, *J* = 7.4, 3.7, 2.1 Hz, 4H), 7.38 – 7.17 (m, 13H), 6.87 – 6.76 (m, 8H), 5.64 (d, *J* = 4.2 Hz, 2H), 4.59 (s, 1H), 4.58 – 4.52 (m, 2H), 4.51 (s, 1H), 4.31 (d, *J* = 9.3 Hz, 1H), 4.17 (d, *J* = 6.9 Hz, 1H), 3.90 (ddt, *J* = 12.2, 6.5, 3.3 Hz, 4H), 3.86 – 3.73 (m, 20H), 3.60 – 3.41 (m, 15H), 2.00 – 1.92 (m, 6H), 1.81 – 1.70 (m, 6H), 1.63 (d, *J* = 1.2 Hz, 2H), 1.56 (d, *J* = 1.2 Hz, 3H), 1.12 (dd, *J* = 11.9, 6.8 Hz, 12H), 1.05 (d, *J* = 6.8 Hz, 5H), 0.99 (d, *J* = 6.7 Hz, 5H). **^{13}C NMR** (101 MHz, $CDCl_3$, ^{1}H decoupled**,** mixture of R/S-isomer) δ 163.91, 163.88, 158.71, 158.66, 149.74, 144.36, 144.22, 135.37, 135.28, 135.26, 135.20, 134.53, 134.45, 130.26, 130.20, 130.15, 130.07, 128.30, 128.12, 127.92, 127.13, 127.04, 113.22, 113.20, 113.18, 113.16, 110.27, 110.18, 87.98, 87.96, 87.94, 87.90, 87.43, 87.41, 86.66, 86.64, 78.66, 78.05, 77.37, 77.05, 76.74, 72.33, 72.19, 70.83, 70.72, 70.29, 70.14, 69.77, 69.72, 68.72, 64.98, 64.87, 55.17, 45.81, 45.18, 45.12, 43.25, 43.20, 43.13, 43.07, 34.08, 33.91, 33.87, 33.73, 33.69, 24.62, 24.54, 24.46, 24.43, 24.39, 24.36, 24.28, 24.21, 12.49, 12.39. **^{31}P NMR** (162 MHz, $CDCl_3$) *δ* 147.19, 146.57. **HRMS (ESI$^+$):** *m/z* $[M+H]^+$ calc. for $C_{43}H_{55}N_3O_{10}P$ 804.3620; found 804.3619.

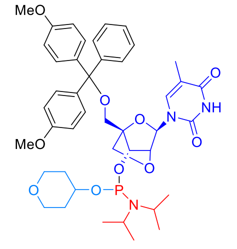

Chemical Formula: $C_{43}H_{54}N_3O_{10}P$
Exact Mass: 803.35
17

5′-*O*-dimethoxytrityl-2′-*O*,4′-*C*-methyleneadenine-3′-*O*-[(*O*-(3-methoxypropyl)-*N*,*N*′-diisopropylphosphoramidite (19): Locked nucleoside monomer **18** (300 mg, 437 µmol, 1.0 equiv.) was dissolved in dry, degassed DCM (10 mL). Phosphitylating reagent **7** (210 mg, 650 µmol, 1.5 equiv.) was added followed by the addition of tetrazole (~0.45M in MeCN, 0.971 mL, 437 µmol, 1.0 equiv.). The reaction was left to stir at rt for 16h under an atmosphere of argon. At completion, the reaction mixture was diluted with anhydrous DCM (10 mL) and saturated KCl solution (10 mL) was added. The biphasic mixture was transferred to a separating funnel. The DCM part was collected and dried over $MgSO_4$ and then concentrated under rotavapor. The work up and evaporation were performed under Argon atmosphere. The crude residue was purified by flash chromatography (20:80→10:90, Hex:EtOAc, both solvent were mixed with 1% triethylamine) to give the desired product **19** as a white solid (280 mg, 309 µmol, 71%). The phosphoramidite **19** had R_f 0.56,0.64 (EtOAc). **^{1}H NMR** (400 MHz, $CDCl_3$, mixture of R/S-isomers) *δ* 8.97 (s, 2H), 8.72 (d, *J* = 3.2 Hz, 1H), 8.25 (d, *J* = 4.1 Hz, 2H), 8.07 – 7.83 (m, 3H), 7.60 – 7.38 (m, 9H), 7.34 – 7.10 (m, 12H), 6.81 – 6.72 (m, 7H), 6.09 (d, *J* = 3.2 Hz, 2H), 4.82 (d, *J* = 2.7 Hz,

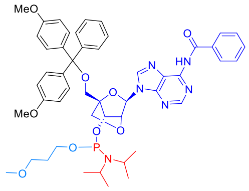

Chemical Formula: $C_{49}H_{57}N_6O_9P$
Exact Mass: 904.39
19

2H), 4.37 (d, J = 8.7 Hz, 1H), 4.29 (d, J = 6.7 Hz, 1H), 4.04 – 3.93 (m, 4H), 3.71 (d, J = 1.2 Hz, 11H), 3.58 – 3.31 (m, 10H), 3.23 – 3.14 (m, 3H), 3.12 (d, J = 2.9 Hz, 5H), 1.58 (dd, J = 12.5, 6.2 Hz, 3H), 1.25 – 1.15 (m, 4H), 1.00 (dd, J = 6.8, 5.5 Hz, 11H), 0.89 (d, J = 6.8 Hz, 4H), 0.81 (dd, J = 13.7, 7.0 Hz, 8H). **^{13}C NMR** (101 MHz, $CDCl_3$, ^{1}H decoupled**,** mixture of R/S-isomers) δ 164.42, 158.52, 152.72, 150.68, 150.59, 149.41, 144.32, 144.29, 140.36, 135.54, 135.50, 135.37, 135.34, 133.58, 132.72, 130.03, 130.00, 129.96, 128.82, 128.11, 128.04, 127.84, 127.76, 126.88, 123.44, 113.13, 113.12, 87.89, 87.84, 87.72, 87.66, 86.88, 86.86, 86.34, 86.31, 78.79, 78.16, 77.27, 76.96, 76.64, 72.93, 72.82, 72.32, 72.19, 71.72, 71.57, 69.01, 68.93, 60.62, 60.45, 60.14, 59.97, 59.35, 59.12, 58.44, 58.42, 55.11, 43.04, 42.92, 31.17, 31.10, 24.45, 24.38, 24.31, 24.28, 24.21. **^{31}P NMR** (162 MHz, $CDCl_3$) δ 148.15, 147.95. **HRMS (ESI$^+$):** *m/z* $[M+H]^+$ calc. for $C_{49}H_{58}N_6O_9P$ 905.3997; found 905.3995.

5′-*O*-dimethoxytrityl-2′-*O*,4′-*C*-methyleneadenine-3′-*O*-[(*O*-(isopropyl)-*N*,*N*´-diisopropylphosphoramidite (20): Locked nucleic acid monomer **18** (300 mg, 437 µmol, 1.0 equiv.) was dissolved in dry, degassed DCM (10 mL). Phosphitylating reagent 1-isopropoxy-*N*,*N*,*N'*,*N'*-tetraisopropylphosphanediamine **8** (2 eq, 0.2 mL) was added followed by the addition of and tetrazole (0.45M in MeCN, 971 µL, 437 µmol, 1.0 eqv.). The reaction mixture was left to stir under argon at rt for 16h. Following a TLC analysis, the reaction mixture was diluted with anhydrous DCM (10 mL) and washed with saturated KCl (10 mL).

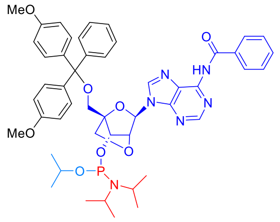

Chemical Formula: $C_{48}H_{55}N_6O_8P$
Exact Mass: 874.38
20

The DCM part was collected, dried over MgSO4, and concentrated. The residue was purified by flash chromatography (40:60 Hex:EtOAc, mixed with 1% triethylamine) to give the desired phosphoramidite **20** (260 mg, 297 µmol, 68%) as a white solid. The **20** had R_f 0.74 (EtOAc, mixed with 1% triethylamine). **^{1}H NMR** (400 MHz, $CDCl_3$, mixture of R/S-isomers) δ 9.02 (s, 2H), 8.81 (s, 1H), 8.32 (s, 1H), 8.07 – 7.97 (m, 4H), 7.66 – 7.42 (m, 8H), 7.41 – 7.15 (m, 14H), 6.89 – 6.80 (m, 9H), 6.16 (d, J = 1.6 Hz, 2H), 4.91 (d, J = 4.8 Hz, 2H), 4.37 (d, J = 8.9 Hz, 1H), 4.28 (d, J = 7.0 Hz, 1H), 4.14 – 3.99 (m, 4H), 3.98 – 3.86 (m, 1H), 3.83 – 3.73 (m, 13H), 3.59 – 3.35 (m, 7H), 1.27 – 1.21 (m, 4H), 1.09 – 1.05 (m, 15H), 0.97 – 0.89 (m, 16H). **^{13}C NMR** (101 MHz, $CDCl_3$, ^{1}H decoupled**,** mixture of R/S-isomers) δ 164.46, 158.58, 152.82, 149.45, 144.43, 140.40, 135.63, 135.43, 133.69, 132.79, 130.13, 130.09, 130.04, 128.89, 128.20, 128.11, 127.91, 127.89, 127.82, 126.94, 123.40, 113.20, 113.18, 87.97, 87.71, 87.00, 86.41, 86.36, 78.80, 78.13, 77.33, 77.01, 76.70, 73.05, 71.55, 67.03, 66.83, 59.46, 59.38, 55.18, 43.06, 42.95, 42.93, 24.47, 24.39, 24.31, 24.27, 24.22, 24.13, 22.64, 22.32. **^{31}P NMR** (162 MHz, $CDCl_3$) δ 147.22, 146.21. **HRMS (ESI$^+$):** *m/z* $[M+H]^+$ calc. for $C_{48}H_{56}N_6O_8P$ 875.3892; found 875.3882.

5′-*O*-dimethoxytrityl-2′-*O*,4′-*C*-methyleneadenine-3′-*O*-[(*O*-(*tert*-butyl)-*N*,*N*´-diisopropylphosphoramidite (21):

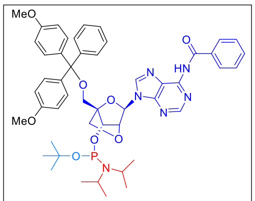

Chemical Formula: $C_{49}H_{57}N_6O_8P$
Exact Mass: 888.40
21

To a solution of locked nucleic acid adenine monomer **18** (400 mg, 583 µmol, 1.0 equiv.) in anhydrous, degassed DCM (12 mL) was added 1-tert-butoxy-*N*,*N*,*N'*,*N'*-tetraisopropylphosphanediamine **9** (533 mg, 1.75 mmol, 3.0 equiv.). Tetrazole (~0.45 M in MeCN, 1.55 mL, 700 µmol, 1.2 equiv.) was added by a syringe. The reaction was left to stir under argon for 16h, At completion, the reaction was diluted with dry DCM (10 mL) and transferred to a separating funnel. The organic phase washed saturated KCl solution (15 mL), dried over Na_2SO_4, then concentrated under reduced pressure. The crude product was purified by flash chromatography (20:80→10:90 Hex:EtOAc, mixed with 1% Et_3N) to give the desired phosphoramidite **21** as a white solid (240 mg, 270 µmol, 46%). The **21** had R_f 0.83 (20:80 Hex:EtOAc, mixed with 1% triethylamine). **^{1}H NMR** (600 MHz, $CDCl_3$, mixture of R/S-isomers) δ 9.09 (s, 2H), 8.82 (d, J = 8.8 Hz, 2H), 8.35 (s, 2H), 8.06 (d, J = 7.3 Hz, 4H), 7.62 (td, J = 7.2, 1.4 Hz, 2H), 7.57 – 7.49 (m, 8H), 7.40 (dt, J = 9.0, 2.8 Hz, 8H), 7.36 – 7.30 (m, 4H), 7.28 – 7.22 (m, 2H), 6.90 – 6.84 (m, 8H), 6.18 (dd, J = 4.5, 0.7 Hz, 2H), 4.93 (d, J = 6.3 Hz, 2H), 4.31 (d, J = 9.4 Hz, 1H), 4.20 (d, J = 7.1 Hz, 1H), 4.11 (dd, J = 17.4, 7.7 Hz, 2H), 4.05 (t, J = 8.1 Hz, 2H), 3.81 (s, 11H), 3.62 – 3.55 (m, 3H), 3.58 – 3.48 (m, 3H), 3.52 – 3.42 (m, 1H), 1.12 – 1.06 (m, 28H), 0.93 – 0.87 (m, 15H). **^{13}C NMR** (151 MHz, $CDCl_3$, ^{1}H decoupled, mixture of R/S-isomers) δ 164.63, 158.69, 152.93, 150.61, 149.61, 144.58, 144.55, 140.45, 140.41, 135.77, 135.75, 135.56, 135.55, 133.84, 132.87, 130.24, 130.20, 130.16, 130.13, 128.98, 128.29, 128.23, 128.18, 128.15, 128.12, 128.02, 127.96, 127.05, 123.51, 113.44, 113.42, 113.32, 88.11, 88.08, 87.94, 87.90, 87.25, 87.22, 86.51, 86.48, 78.81, 78.79, 78.26, 78.24, 77.37, 77.16, 76.95, 75.39, 75.32, 73.20, 73.13, 71.76, 71.69, 71.33, 71.24, 59.63, 59.50, 55.30, 43.21, 43.12, 30.73, 30.68, 24.59, 24.54, 24.38, 24.34, 24.16, 24.11, 24.03, 23.98. **^{31}P NMR** (162 MHz, $CDCl_3$) δ 140.45, 139.40. **HRMS (ESI$^+$):** *m/z* $[M+H]^+$ calc. for $C_{49}H_{58}N_6O_8P$ 889.4048; found 889.4004.

5′-*O*-dimethoxytrityl-2′-*O*,4′-*C*-methyleneadenine-3′-*O*-[(*O*-(tetrahydro-2H-pyran-4-yl)-*N*,*N*´-diisopropylphosphoramidite (22):

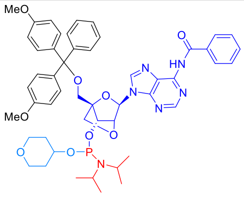

Chemical Formula: $C_{50}H_{57}N_6O_9P$
Exact Mass: 916.39
22

Locked nucleic acid monomer **18** (400 mg, 583 µmol, 1.0 equiv.) was dissolved in dry, degassed DCM (10 mL). Tetrazole (0.45M in MeCN, 1.29 mL, 1.0 equiv.) and phosphitylating reagent **10** (388 mg, 1.17 mmol, 2.0 eqv.) were added. The reaction mixture was left to stir at rt for 16h under argon. At completion, the reaction mixture was diluted with dry DCM (10 mL). The mixture was washed with saturated KCl solution (10 mL) and the DCM part was collected and dried over $MgSO_4$. The work up was performed under Argon. The DCM part was concentrated and the crude residue was purified by flash chromatography (90:10 Hex:EtOAc, mixed with 1% Et_3N) to give the desired phosphoramidite product **22** as a white solid (340 mg, 317 µmol 64%). The phosphoramidite **22** had R_f 0.81 (1:4 Hex:EtOAc, mixed with 1% triethylamine). **^{1}H NMR** (600 MHz, $CDCl_3$, mixture of R/S-isomer) δ 9.10 (s, 2H), 8.79 (s, 2H), 8.32 (d, J = 5.2 Hz, 2H), 8.06 – 8.01 (m, 5H), 7.63 – 7.57 (m, 2H), 7.55 – 7.42 (m, 9H), 7.39 – 7.33 (m, 8H), 7.36 – 7.27 (m, 5H), 7.27 – 7.19 (m, 2H), 6.87 – 6.81 (m, 9H), 6.16 (dd, J = 2.3, 0.7 Hz, 2H), 4.92 (d, J = 10.0 Hz, 2H), 4.39 (d, J = 9.0 Hz, 1H), 4.30

(d, J = 7.1 Hz, 1H), 4.09 (dd, J = 7.8, 5.6 Hz, 2H), 4.00 (dd, J = 7.8, 4.3 Hz, 2H), 3.93 – 3.83 (m, 1H), 3.79 (d, J = 3.0 Hz, 13H), 3.78 – 3.67 (m, 3H), 3.63 (tdd, J = 11.0, 6.1, 3.8 Hz, 2H), 3.58 – 3.35 (m, 10H), 3.28 (dddd, J = 23.2, 11.5, 8.2, 3.3 Hz, 2H), 1.76 – 1.63 (m, 2H), 1.60 – 1.40 (m, 3H), 1.35 – 1.24 (m, 6H), 1.07 (dd, J = 12.5, 6.8 Hz, 13H), 0.95 (d, J = 6.8 Hz, 6H), 0.89 (d, J = 3.7 Hz, 4H). **^{13}C NMR** (151 MHz, $CDCl_3$, ^{1}H decoupled, mixture of R/S-isomers) δ 164.56, 158.62, 158.60, 152.81, 150.68, 150.55, 149.56, 144.38, 144.32, 140.38, 140.35, 135.57, 135.52, 135.39, 133.62, 132.81, 132.79, 130.11, 130.08, 130.06, 130.02, 128.88, 128.86, 128.19, 128.09, 127.92, 127.89, 127.02, 126.97, 123.54, 123.41, 113.21, 113.20, 87.96, 87.93, 87.83, 87.79, 87.02, 86.45, 86.40, 78.69, 78.67, 78.09, 78.06, 77.25, 77.04, 76.83, 73.05, 72.94, 72.07, 71.99, 71.61, 71.52, 68.64, 68.51, 65.84, 65.02, 64.99, 64.90, 64.87, 59.30, 59.27, 55.20, 43.16, 43.08, 34.11, 34.02, 33.89, 33.84, 24.50, 24.45, 24.37, 24.32, 24.28, 24.24, 24.19. **^{31}P NMR** (162 MHz, $CDCl_3$) δ 147.31, 146.68. **HRMS (ESI$^+$):** m/z [M+H]$^+$ calc. for $C_{50}H_{58}N_6O_9P$ 917.3997; found 917.4005.

5′-*O*-dimethoxytrityl-2′-*O*,4′-*C*-methyleneadenine-3′-*O*-[(*O*-(1-(hexadecyl)-*N,N*´-diisopropylphosphoramidite (23): To a solution of DMT-LNA-A-Bz-OH **18** (400 mg, 583 µmol, 1.0 equiv.) in dry, degassed DCM (15 mL) was added tetrazole (0.45M in MeCN, 1.29 mL, 583 µmol, 1.0 eqv.). Phosphitylating reagent **11** (414 mg, 875 µmol, 1.5 equiv.) was then added and the reaction was stirred at rt for 16h under an atmosphere of argon. At completion the reaction was diluted with dry DCM (10 mL). The reaction mixture was then washed with degassed saturated KCl solution (10 mL) and the DCM part was dried over Magnesium sulfate. The work up was performed under Argon. The DCM part was concentrated and the residue was purified by flash chromatography (50:50 Hex:EtOAc, mixed with 1% Et_3N) to give the desired product as a white solid (400 mg, 378 µmol, 65%). The phosphoramidite **23** had R_f 0.60 (3:7 Hex;EtOAc, mixed with 1% triethylamine). **^{1}H NMR** (600 MHz, $CDCl_3$, mixture of R/S-isomer) δ 9.02 (s, 2H), 8.82 (d, J = 3.8 Hz, 2H), 8.34 (d, J = 4.6 Hz, 2H), 8.07 – 8.03 (m, 5H), 7.66 – 7.60 (m, 2H), 7.58 – 7.46 (m, 9H), 7.42 – 7.35 (m, 8H), 7.37 – 7.31 (m, 2H), 7.31 (dt, J = 6.6, 2.1 Hz, 2H), 7.28 – 7.21 (m, 2H), 6.89 – 6.83 (m, 9H), 6.18 (dd, J = 5.1, 0.7 Hz, 2H), 4.91 (d, J = 7.0 Hz, 2H), 4.46 (d, J = 8.6 Hz, 1H), 4.38 (d, J = 6.6 Hz, 1H), 4.17 – 4.08 (m, 6H), 4.05 (t, J = 8.1 Hz, 2H), 3.81 (d, J = 0.9 Hz, 14H), 3.61 – 3.42 (m, 8H), 3.36 (dtd, J = 8.0, 6.6, 1.2 Hz, 2H), 1.46 – 1.36 (m, 2H), 1.35 – 1.20 (m, 59H), 1.10 (t, J = 7.2 Hz, 13H), 0.98 (d, J = 6.8 Hz, 6H), 0.93 (d, J = 6.7 Hz, 6H). **^{13}C NMR** (151 MHz, $CDCl_3$, ^{1}H decoupled, mixture of R/S-isomers) δ 171.15, 164.45, 164.42, 158.60, 152.82, 150.79, 150.68, 149.47, 144.44, 144.39, 140.47, 140.43, 135.65, 135.61, 135.49, 135.45, 133.68, 132.81, 130.13, 130.10, 130.06, 128.92, 128.23, 128.14, 127.93, 127.91, 127.84, 126.97, 126.96, 123.50, 123.48, 113.22, 113.19, 87.97, 87.94, 87.79, 87.75, 86.97, 86.93, 86.42, 86.39, 78.92, 78.90, 78.28, 78.25, 77.26, 77.05, 76.84, 73.04, 72.93, 72.36, 72.27, 71.76, 71.66, 63.97, 63.86, 63.46, 63.35, 60.40, 59.47, 59.26, 55.20, 43.10, 43.08, 43.02, 43.00, 31.94, 29.71, 29.69, 29.67, 29.64, 29.58, 29.56, 29.37, 25.77, 24.53, 24.48, 24.46, 24.41, 24.37, 24.32, 22.70, 21.06, 14.21, 14.13. **^{31}P NMR** (162 MHz, $CDCl_3$) δ 147.97, 147.87. **HRMS (ESI$^+$):** m/z [M+H]$^+$ calc. for $C_{61}H_{82}N_6O_8P$ 1057.5926; found 1057.5959.

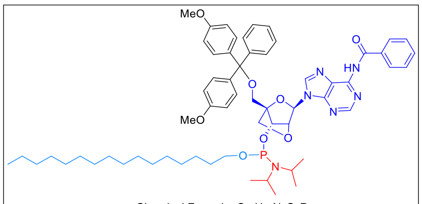

Chemical Formula: $C_{61}H_{81}N_6O_8P$
Exact Mass: 1056.59
23

5′-*O*-dimethoxytrityl-2′-*O*,4′-*C*-methyleneadenine-3′-*O*-[(*O*-1-(hex-5-yn-1-yl)-*N*,*N*´-diisopropylphosphoramidite (24): To a solution of DMT-LNA-A-Bz-OH **18** (420 mg, 612 µmol, 1.0 equiv.) in dry, degassed DCM (15 mL) was added tetrazole (0.45M in MeCN, 1.36 mL, 612 µmol, 1.0 equiv.) . Phosphitylating reagent **12** (302 mg, 919 µmol, 1.5 equiv.) was then added and the reaction was stirred at rt for 16h under argon at rt. At completion, the reaction was diluted with dry, degassed DCM (10 mL). The reaction mixture was washed with saturated, degassed KCl solution (10 mL) and the DCM part was dried over $MgSO_4$. The work up was performed under Argon. The DCM part was filtered, concentrated, and the crude residue was purified by flash chromatography (40:60 Hex:EtOAc, mixed with 1% Et_3N) to give the desired product **24** as a white solid (400 mg, 438 µmol, 71%).

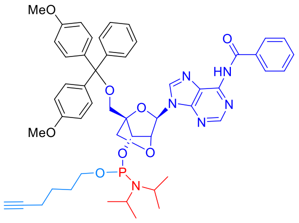

Chemical Formula: $C_{51}H_{57}N_6O_8P$
Exact Mass: 912.40
24

^{1}H NMR (400 MHz, $CDCl_3$, mixture of R/S-isomers) δ 9.05 (d, J = 3.4 Hz, 2H), 8.79 (d, J = 4.4 Hz, 1H), 8.32 (d, J = 4.8 Hz, 2H), 8.08 – 7.97 (m, 4H), 7.65 – 7.56 (m, 2H), 7.56 – 7.43 (m, 7H), 7.41 – 7.17 (m, 12H), 6.87 – 6.78 (m, 7H), 6.16 (d, J = 2.6 Hz, 2H), 4.89 (d, J = 3.8 Hz, 2H), 4.43 (d, J = 8.8 Hz, 1H), 4.36 (d, J = 6.8 Hz, 1H), 4.18 – 4.05 (m, 4H), 4.02 (dd, J = 7.9, 5.0 Hz, 2H), 3.78 (d, J = 1.7 Hz, 11H), 3.58 – 3.39 (m, 8H), 3.35 (dt, J = 7.9, 6.1 Hz, 1H), 2.08 (tdd, J = 6.7, 4.0, 2.7 Hz, 3H), 1.94 – 1.82 (m, 1H), 1.59 – 1.45 (m, 3H), 1.41 (dddd, J = 12.9, 7.1, 5.9, 1.8 Hz, 4H), 1.25 (t, J = 7.1 Hz, 3H), 1.07 (dd, J = 6.8, 4.8 Hz, 11H), 0.95 (d, J = 6.8 Hz, 4H), 0.90 (d, J = 6.8 Hz, 7H). **^{13}C NMR** (101 MHz, $CDCl_3$, ^{1}H decoupled, mixture of R/S-isomers) δ 171.24, 164.65, 158.71, 152.89, 150.91, 150.80, 149.61, 144.52, 144.46, 140.55, 135.73, 135.69, 135.57, 135.53, 133.77, 132.91, 130.23, 130.20, 130.15, 129.00, 128.32, 128.23, 128.03, 127.97, 127.10, 127.07, 123.69, 113.33, 113.30, 88.07, 88.03, 87.92, 87.86, 87.08, 87.04, 86.53, 86.49, 84.21, 84.13, 78.99, 78.34, 77.48, 77.36, 77.16, 76.84, 73.13, 73.03, 72.48, 72.34, 71.92, 71.76, 68.71, 68.67, 63.36, 63.19, 62.86, 62.69, 60.49, 59.57, 59.37, 55.31, 43.24, 43.22, 43.12, 43.09, 30.12, 30.09, 30.04, 30.02, 24.94, 24.88, 24.64, 24.60, 24.56, 24.52, 24.49, 24.47, 24.43, 24.39, 18.04. **^{31}P NMR** (162 MHz, $CDCl_3$) δ 148.07, 148.05. **HRMS (ESI$^+$):** *m/z* $[M+H]^+$ calc. for $C_{15}H_{58}N_6O_8P$ 913.4048; found 913.4048.

2.0 Oligonucleotide synthesis and UPLC-MS of oligonucleotides

2′OMe phosphothiotriester oligonucleotide synthesis and deprotection from the solid support (ON1-ON41, ONOX1, ONOX4 and ON44-ON45)

2′-OMe oligonucleotides were synthesised on an Applied Biosystems 394 automated DNA/RNA synthesiser using a standard phosphoramidite cycle of detritylation, coupling, capping, and sulfurisation/oxidation on a 1.0 µmole scale. Detritylation, coupling, capping, oxidation and activation reagents are identical to those used for standard DNA/RNA synthesis. Trichloroacetic acid (TCA) (3% in CH_2Cl_2) was used for detritylation, 5-Benzylthio-1*H*-tetrazole (BTT) (0.3 M in MeCN) was used as an activator, and oxidation was achieved using iodine (0.02 M in THF, pyridine and water). Pre-packed nucleoside SynBase™ CPG 1000/110 resins (Link Technologies) were used, and β-cyanoethyl phosphoramidite monomers (DMT-2′O-methyl-rA(Bz), DMT-2′O-methyl-rG(dmf), DMT-2′-O-methyl-rC(Ac) and DMT-2′O-methyl-rU) were dissolved in anhydrous MeCN (10% CH_2Cl_2 was added when 2′OMe U phosphoramidite was used) to a concentration of 0.1 M immediately prior to use with a coupling time of 6 min. LNA phosphoramidite monomers (**14-17** and **19-24**) were dissolved to a concentration of 0.1 M MeCN immediately prior to use with a coupling time of 6 min. Stepwise coupling efficiencies were determined by automated trityl cation conductivity monitoring and were >97% in all cases. Except for **ON35-ON38**, all the oligonucleotides have 5´-DMT protecting group still in place (DMTON). Cleavage and deprotection were achieved by treatment with 0.5 mL of THF and 0.5 mL of ethylenediamine (EDA) for 2 hrs room temperature. The mixture of EDA-THF and ethylenediamine was discarded and then the resin was washed with 1 mL of DNase free water to give the crude oligonucleotides. The DMTON oligonucleotides were purified by HPLC (condition of purification listed below) and then DMT group was removed by using the procedure below.

Condition of DMT-group removal

100 µL of 80% AcOH was added to the DMTON product. The reaction was left for 30 mins at rt. The solution became light red colour. The reaction was quenched by 600 µL of water and 300 µL of 2M TEAA buffer (pH 8.5). The de-tritylated oligonucleotide were desalted using NAP-10 (Cytiva) and then freeze dried or purified to give the final oligonucleotides. However, oligonucleotides **ON1-ON8** and **ON38-ON41,** gave mixture of products resulting from the cleavage of the primary alkyl groups from the PTTE backbone were finally purified by HPLC (condition of purification listed below).

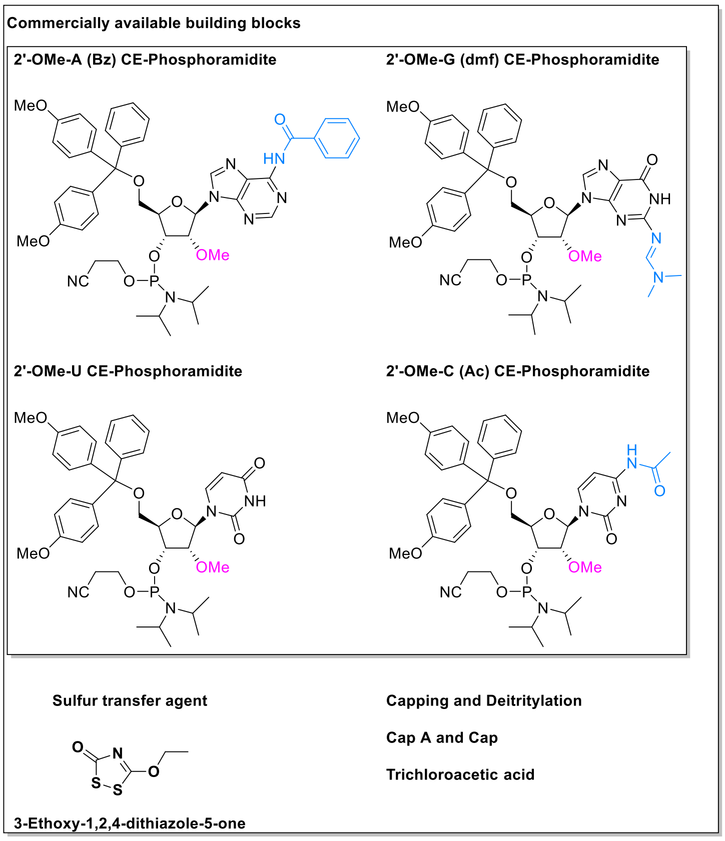

Supplementary Figure S1: List of monomers and sulfur transfer agent used in the solid phase oligonucleotide synthesis.

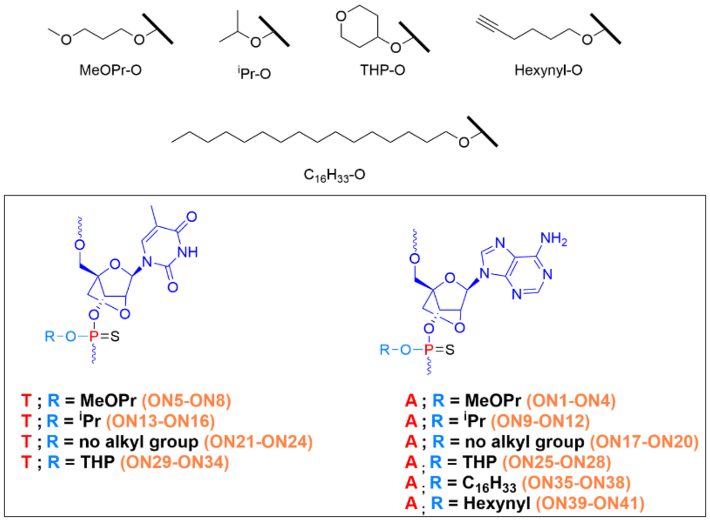

Supplementary Figure S2: Chemical structure of the T and A monomer units present in oligonucleotides **ON1-ON41**. The alkyl phosphothiotriester linkages present as a mixture of two diastereomers.

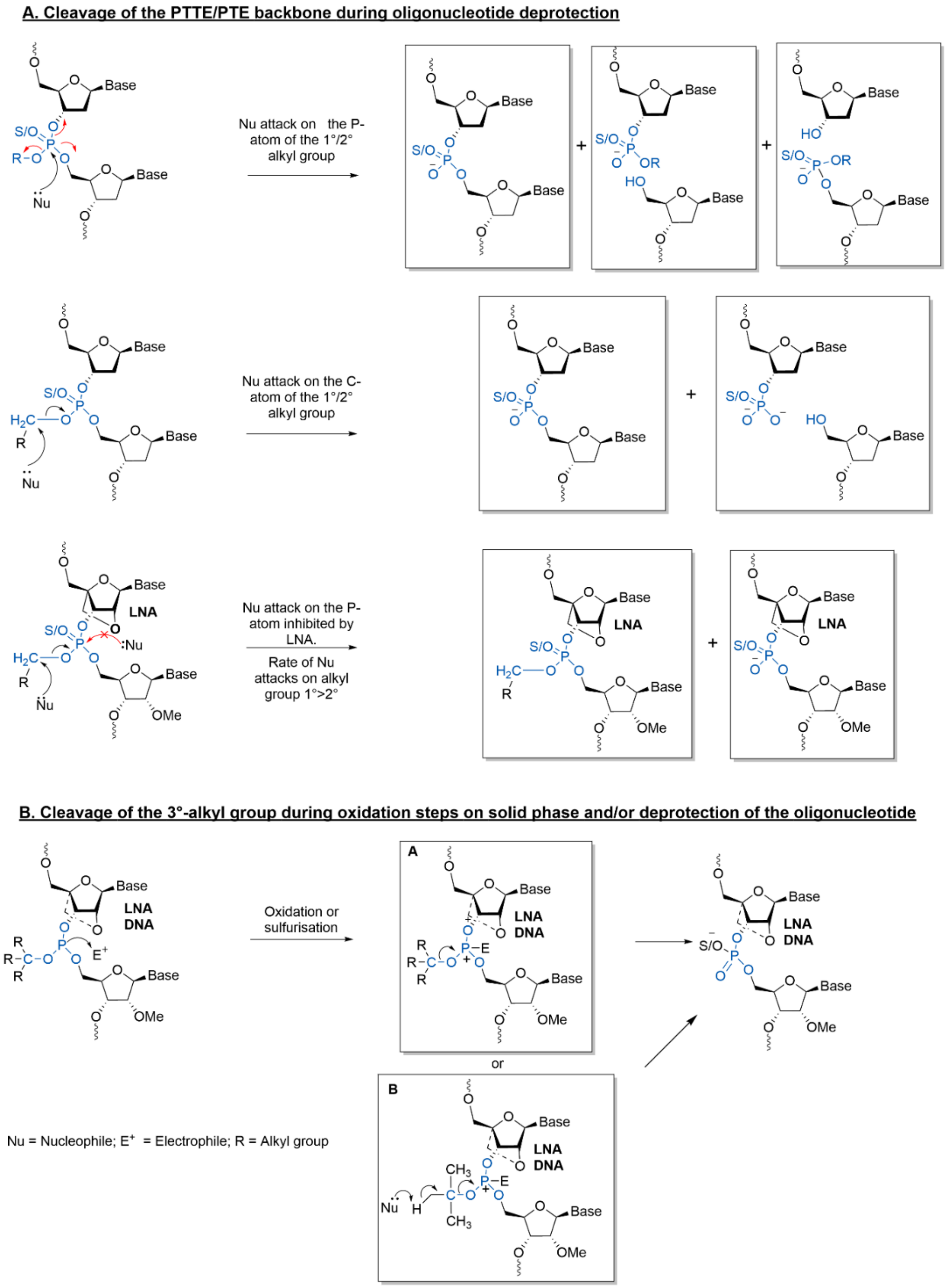

Supplementary Figure S3: A. Possible mechanisms for the cleavage of the phosphothiotriester (PTTE) and phosphotriester (PTE) backbones. **B.** Cleavage of *tert*-alkyl group during P(III) oxidation steps on solid phase.

Target DNA Synthesis (ON42) and deprotection from solid support: DNA synthesis was performed on an Applied Biosystems 394 automated DNA/RNA synthesiser using a standard phosphoramidite cycle of detritylation, coupling, capping, and oxidation on a 1.0 μmole scale. Trichloroacetic acid (TCA) (3% in dichloromethane) was used for detritylation, ethylthiotetrazole (ETT) (0.25 M in MeCN) was used as an activator, and oxidation was achieved using iodine (0.02 M in THF, pyridine and water). Pre-packed nucleoside SynBase™ CPG 1000/110 (Link Technologies) were used and β-cyanoethyl phosphoramidite monomers (dA(Bz), dG(iBu), dC+(Bz) and dT, Sigma-Aldrich) were dissolved in anhydrous MeCN (0.1 M) immediately prior to use with coupling time of 40 s. Stepwise coupling efficiencies were determined by automated trityl cation conductivity monitoring and were >98% in all cases. Cleavage and deprotection were achieved by exposure to concentrated aqueous ammonia solution for 60 min at room temperature followed by heating in a sealed tube for 5 h at 55 °C. The suspension was filtered and washed with water. The filtrate was concentrated under reduced pressure and dissolved in water (1 mL). The solution was transferred to a 10 mL falcon tube and then lyophilised. The crude product was purified by reversed-phase HPLC (Condition III) to give the target DNA **ON42**.

Target RNA synthesis (ON43) and cleavage: RNA synthesis was performed on an Applied Biosystems 394 automated DNA/RNA synthesiser using a standard phosphoramidite cycle of detritylation, coupling, capping, and oxidation on a 1.0 μmole scale. Coupling, capping and oxidation reagents were identical to those used for DNA synthesis except a solution of 5-benzylthio-1*H*-tetrazole (BTT) (0.3 M in MeCN, Link Technologies) was used. Standard CPG resin (Link Technologies) was used and 2'-OTBDMS protected monomers (A(Bz), C(Ac), G(iBu) and U, Sigma-Aldrich) were dissolved in anhydrous MeCN (0.1 M) immediately prior to use. The coupling time for all monomers was 6 min. Stepwise coupling efficiencies were determined by automated trityl cation conductivity monitoring and in all cases were >96%. The RNA was deprotected from solid support in two steps. **Step 1:** 1 mL of NH_4OH and 1 mL of methylamine were added to the solid support in a 5 mL glass vial. The vial was placed in heating bath at 65°C for 10 mins. The reaction mixture was allowed to attain rt. The suspension was filtered and washed with 40% MeCN in water (2*1 mL). The filtrate was concentrated under vacuum (55 °C) until the smell of ammonia goes off and the volume reduced to ~1 mL. The residual solution was transferred to a falcon tube and then lyophilised. **Step 2:** The crude mass was treated with 300 µL of anhydrous DMSO and 300 µL of $Et_3N.HF$. The mixture was left at 65 °C for 3h. The mixture was allowed to attain rt and 50 µL of NaOAc (3M, pH 5.2) and 3 mL of 1-butanol were added. The tube was centrifuged for 30 mins (rt, 5000 rpm). The supernatants were discarded. Then, 1 mL 70% aqueous ethanol was added and centrifuged for 30 mins (rt, 5000 rpm). The supernatant was discarded and the procedure repeated once more. The crude product was then lyophilised, dissolved in water (1.0 mL) and then centrifuged. The precipitate was discarded and the solution was collected which contained the desired RNA. The crude product was purified by reversed-phase HPLC (Condition III) to give the target RNA **ON43**.

Purification of the oligonucleotides (ON1-ON41)

Oligonucleotides were purified using a Gilson reverse-phase high performance liquid chromatography (RP HPLC) system with ACE® C8 column (particle size: 10 µm, pore size: 100 Å, column dimensions: 10 mm x 250 mm) with a gradient of buffer B (0.1 M TEAB, pH 7.5 containing 50%) or Buffer C (0.1 M TEAB in 70% v/v MeCN) in buffer A (0.1 M TEAB, pH 7.5) at a flow rate of 4 mL/min. Several conditions were used depending on the oligonucleotides and presence or absence of the DMT group (**condition I-V**).

Condition I: The gradient of buffer B (0.1 M TEAB, pH 7.5 containing 50% v/v MeCN) was increased linearly from 5% to 100 % buffer B over a period of 20 min.

Condition II: The gradient of buffer B (0.1 M TEAB, pH 7.5 containing 70% v/v MeCN) was increased linearly from 5% to 100% over a period of 20 min.

Condition III: The gradient of buffer B (0.1 M TEAB, pH 7.5 containing 50% v/v MeCN) was increased linearly from 5% to 60% over a period of 20 min.

Condition IV: The gradient of buffer B (0.1 M TEAB, pH 7.5 containing 70% v/v MeCN) was increased linearly from 20% to 80% buffer B over a period of 20 min.

Condition V: The gradient of buffer B (0.1 M TEAB, pH 7.5 containing 70% v/v MeCN) was increased linearly from 30% to 100% buffer C over a period of 20 min.

Elution was monitored by UV absorbance at 260/285/295 nm. After HPLC purification, oligonucleotides were freeze dried then dissolved in water without the need for desalting.

Supplementary Table T1: Condition of purification of oligonucleotides

Oligonucleotides were purified with trityl (DMT) group on in order to separate from polar side products (such as deletion sequence) except for oligonucleotide **ON35-ON38** as the non-polar $C_{16}H_{33}$ group does make much polarity difference in presence or absence of DMT group. Oligonucleotides which have primary alkyl phosphothiotriester ((such as MeOPr (**ON1-ON8**), 5-Hexynyl (**ON39-ON41**)) were purified both after deprotection from the solid support and removal of the DMT group. This is due to the phosphodiester side products obtained from the cleavage of the primary alkyl groups from the PTTE backbone were inseparable during the purification of DMTON oligonucleotides.

**Oligonucleotides**	**HPLC purification condition**	**Oligonucleotides**	**HPLC purification condition**
**DMT-ON1 MeOPr**	Condition I	**ON1 MeOPr**	Condition III
**DMT-ON2 MeOPr**	Condition I	**ON2 MeOPr**	Condition III
**DMT-ON3 MeOPr**	Condition I	**ON3 MeOPr**	Condition III
**DMT-ON4 MeOPr**	Condition II	**ON4 MeOPr**	Condition III
**DMT-ON5 MeOPr**	Condition I	**ON5 MeOPr**	Condition III

**DMT-ON6 MeOPr**	Condition II	**ON6 MeOPr**	Condition III
**DMT-ON7 MeOPr**	Condition II	**ON7 MeOPr**	Condition IV
**DMT-ON8 MeOPr**	Condition II	**ON8 MeOPr**	Condition IV
**DMT-ON9 iPr**	Condition I	**ON9 iPr**	--
**DMT-ON10 iPr**	Condition I	**ON10 iPr**	--
**DMT-ON11 iPr**	Condition I	**ON11 iPr**	--
**DMT-ON12 iPr**	Condition I	**ON12 iPr**	--
**DMT-ON13 iPr**	Condition I	**ON13 iPr**	--
**DMT-ON14 iPr**	Condition I	**ON14 iPr**	--
**DMT-ON15 iPr**	Condition II	**ON15 iPr**	--
**DMT-ON16 iPr**	Condition II	**ON16 iPr**	--
**DMT-ON17 LNA control**	Condition I	**ON39 LNA control**	--
**DMT-ON18 LNA control**	Condition I	**ON17 LNA control**	--
**DMT-ON19 LNA control**	Condition I	**ON18 LNA control**	--
**DMT-ON20 LNA control**	Condition I	**ON19 LNA control**	--
**DMT-ON21 LNA control**	Condition I	**ON20 LNA control**	--
**DMT-ON22 LNA control**	Condition I	**ON21 LNA control**	--
**DMT-ON23 LNA control**	Condition II	**ON22 LNA control**	--
**DMT-ON24 LNA control**	Condition II	**ON24 LNA control**	--
**DMT-ON25 THP**	Condition I	**ON25 THP**	--
**DMT-ON26 THP**	Condition I	**ON26 THP**	--
**DMT-ON27 THP**	Condition I	**ON27 THP**	--
**DMT-ON28 THP**	Condition II	**ON28 THP**	--
**DMT-ON29 THP**	Condition I	**ON29 THP**	--
**DMT-ON30 THP**	Condition I	**ON30 THP**	--
**DMT-ON31 THP**	Condition II	**ON31 THP**	--
**DMT-ON32 THP**	Condition II	**ON32 THP**	--
**DMT-ON33 THP**	Condition V	**ON33 THP**	Condition I
**DMT-ON34 THP**	Condition V	**ON34 THP**	Condition IV
**DMT-ON35 C16**	--	**ON35 C16**	Condition V
**DMT-ON36 C16**	--	**ON36 C16**	Condition V
**DMT-ON37 C16**	--	**ON37 C16**	Condition V
**DMT-ON38 C16**	--	**ON38 C16**	Condition V
**DMT-ON39 Hexynyl**	Condition I	**ON39 Hexynyl**	Condition III
**DMT-ON40 Hexynyl**	Condition I	**ON40 Hexynyl**	Condition III

**DMT-ON41 Hexynyl**	Condition I	**ON41 Hexynyl**	Condition IV
**DMT-ONOX1 iPr**	Condition I	**ONOX1 iPr**	Condition III
**DMT-ONOX4 iPr**	Condition I	**ONOX4 iPr**	Condition III

Oligonucleotide analysis

All oligonucleotides were characterised by negative-mode ultra-performance liquid chromatography (UPLC) mass spectrometry using a Waters Xevo G2-XS QT of mass spectrometer with an Acquity UPLC system. The system is equipped with an Acquity UPLC oligonucleotide BEH C18 column (particle size: 1.7 µm; pore size: 130 Å; column dimensions: 2.1 mm x 50 mm). Data were analysed using Waters MassLynx software v 4.1 or Waters UNIFI Scientific Information System software.

Supplementary Table T2: Yield of solid phase synthesis

Nucleotides in black have 2´-OMe ribose sugars and phosphorothioate internucleoside linkages. Nucleotides in red are locked nucleic acid phosphorothioate triesters.

ON	Sequence	Modification	Alkyl group	Product obtained (nM)	Yield (%)
ON1 MeOPr	CCU CUU ACC UCA GUU ACA	1	MeOPr	216	22[I]
ON2 MeOPr	CCU CUU ACC UCA GUU ACA	2	MeOPr	189	19[I]
ON3 MeOPr	CCU CUU ACC UCA GUU ACA	2	MeOPr	189	19[I]
ON4 MeOPr	CCU CUU ACC UCA GUU ACA	3	MeOPr	122	12[I]
ON5 MeOPr	CCU CUU ACC UCA GUT ACA	1	MeOPr	218	22[I,d]
ON6 MeOPr	CCU CUT ACC UCA GUT ACA	2	MeOPr	170	17[I,c]
ON7 MeOPr	CCT CUT ACC TCA GUT ACA	4	MeOPr	77	8[I,b]
ON8 MeOPr	CCT CTT ACC TCA GTT ACA	6	MeOPr	52	5[I,a]
ON9 iPr	CCU CUU ACC UCA GUU ACA	1	^{i}Pr	424	42
ON10 iPr	CCU CUU ACC UCA GUU ACA	2	^{i}Pr	391	39
ON11 iPr	CCU CUU ACC UCA GUU ACA	2	^{i}Pr	406	41
ON12 iPr	CCU CUU ACC UCA GUU ACA	3	^{i}Pr	219	22
ON13 iPr	CCU CUU ACC UCA GUT ACA	1	^{i}Pr	370	37
ON14 iPr	CCU CUT ACC UCA GUT ACA	2	^{i}Pr	403	40
ON15 iPr	CCT CUT ACC TCA GUT ACA	4	^{i}Pr	291	29
ON16 iPr	CCT CTT ACC TCA GTT ACA	6	^{i}Pr	181	18
ON17 LNA control	CCU CUU ACC UCA GUU ACA	1	--	335	34
ON18 LNA control	CCU CUU ACC UCA GUU ACA	2	--	238	24
ON19 LNA control	CCU CUU ACC UCA GUU ACA	2	--	282	28
ON20 LNA control	CCU CUU ACC UCA GUU ACA	3	--	297	30
ON21 LNA control	CCU CUU ACC UCA GUT ACA	1	--	306	31
ON22 LNA control	CCU CUT ACC UCA GUT ACA	2	--	293	29
ON23 LNA control	CCT CUT ACC TCA GUT ACA	4	--	168	17
ON24 LNA control	CCT CTT ACC TCA GTT ACA	6	--	162	16
ON25 THP	CCU CUU ACC UCA GUU ACA	1	THP	363	36
ON26 THP	CCU CUU ACC UCA GUU ACA	2	THP	133	13
ON27 THP	CCU CUU ACC UCA GUU ACA	2	THP	398	40
ON28 THP	CCU CUU ACC UCA GUU ACA	3	THP	461	46

ON29 THP	CCU CUU ACC UCA GUT ACA	1	THP	352	35
ON30 THP	CCU CUT ACC UCA GUT ACA	2	THP	324	32
ON31 THP	CCT CUT ACC TCA GUT ACA	4	THP	286	29
ON32 THP	CCT CTT$_X$ ACC TCA GTT ACA	6	THP	267	27
ON33 THP	CCT CUT ACC TCA GUT ACA	7	THP	307	31
ON34 THP	CCT CTT ACC TCA GTT ACA	9	THP	209	21
ON35 C16	CCU CUU ACC UCA GUU ACA	1	C16	304	30 [II]
ON36 C16	CCU CUU ACC UCA GUU ACA	2	C16	149	15[II]
ON37 C16	CCU CUU ACC UCA GUU ACA	2	C16	175	18[II]
ON38 C16	CCU CUU ACC UCA GUU ACA	3	C16	163	16[II]
ON39 Hexynyl	CCU CUU ACC UCA GUU ACA	1	Hexynyl	245	25 [I,d]
ON40 Hexynyl	CCU CUU ACC UCA GUU ACA	2	Hexynyl	151	15 [I,e]
ON41 Hexynyl	CCU CUU ACC UCA GUU ACA	3	Hexynyl	102	10 [I,f]
ONOX1 iPr	CCUCUUACCUCAGUT$_o$ACA	1	^{i}Pr	360	36
ONOX4 iPr	CCT$_o$CUT$_o$ACCT$_o$CAGUT$_o$ACA	4	^{i}Pr	269	27

Oligonucleotide synthesis was performed in 1 µmole scale. The yield is based on the total amount of product obtained. All oligonucleotides were obtained as white solids. [I]The final products were purified by HPLC to remove the phosphodiester group-containing side products. [II]The DMT group was removed during solid phase synthesis and the final product was purified by HPLC. [a]Side product containing less than six methoxypropyl phosphothiotriester was obtained 173 nmol (17%); [b]side product containing less than four methoxypropyl phosphothiotriester was obtained 158 nmol (16%); [c]side product containing less than two methoxypropyl phosphothiotriester obtained 145 nmol (15%); [d]side product containing only phosphodiester backbones was obtained 79 nmol (8%). [e]Side products containing only one 5-hexyn phosphothiotriester was obtained 115 nmol (11%); [f]Side products containing less than three 5-hexyn phosphothiotriester was obtained 289 nmol (29%). ^{t}Bu: tert-butyl group was cleaved during the automated synthesis resulting only phosphodiester oligonucleotides.

Supplementary Table T3: Observed Masses of synthesised oligonucleotides

Nucleotides in black have 2´-OMe ribose sugars and phosphorothioate internucleoside linkages. Nucleotides in red are locked nucleic acid phosphorothioate triesters.

ON	Sequence (5'→3')	PTTE	Alkyl Group (R)	Mass Expected	Mass Observed
ON1 MeOPr	CCU CUU ACC UCA GUU ACA	1	MeOPr	6168.97	6168.50
ON2 MeOPr	CCU CUU ACC UCA GUU ACA	2	MeOPr	6239.06	6238.30
ON3 MeOPr	CCU CUU ACC UCA GUU ACA	2	MeOPr	6239.06	6238.70
ON4 MeOPr	CCU CUU ACC UCA GUU ACA	3	MeOPr	6309.15	6309.30
ON5 MeOPr	CCU CUU ACC UCA GUT ACA	1	MeOPr	6183.00	6181.20
ON6 MeOPr	CCU CUT ACC UCA GUT ACA	2	MeOPr	6267.11	6266.50
ON7 MeOPr	CCT CUT ACC TCA GUT ACA	4	MeOPr	6435.35	6434.00
ON8 MeOPr	CCT CTT ACC TCA GTT ACA	6	MeOPr	6603.59	6603.30
ON9 iPr	CCU CUU ACC UCA GUU ACA	1	^{i}Pr	6138.94	6138.70
ON10 iPr	CCU CUU ACC UCA GUU ACA	2	^{i}Pr	6179.01	6179.00
ON11 iPr	CCU CUU ACC UCA GUU ACA	2	^{i}Pr	6179.01	6178.90
ON12 iPr	CCU CUU ACC UCA GUU ACA	3	^{i}Pr	6219.07	6219.00
ON13 iPr	CCU CUU ACC UCA GUT ACA	1	^{i}Pr	6152.97	6152.60
ON14 iPr	CCU CUT ACC UCA GUT ACA	2	^{i}Pr	6207.06	6207.30
ON15 iPr	CCT CUT ACC TCA GUT ACA	4	^{i}Pr	6315.25	6315.60
ON16 iPr	CCT CTT ACC TCA GTT ACA	6	^{i}Pr	6423.43	6423.60
ON17 LNA control	CCU CUU ACC UCA GUU ACA	--[a]	--	6096.86	6096.20
ON18 LNA control	CCU CUU ACC UCA GUU ACA	--[a]	--	6094.85	6094.10
ON19 LNA control	CCU CUU ACC UCA GUU ACA	--[a]	--	6094.85	6093.00
ON20 LNA control	CCU CUU ACC UCA GUU ACA	--[a]	--	6092.83	6091.20
ON21 LNA control	CCU CUU ACC UCA GUT ACA	--[a]	--	6110.89	6110.20
ON22 LNA control	CCU CUT ACC UCA GUT ACA	--[a]	--	6122.90	6122.10
ON23 LNA control	CCT CUT ACC TCA GUT ACA	--[a]	--	6146.92	6144.10
ON24 LNA control	CCT CTT ACC TCA GTT ACA	--[a]	--	6170.94	6169.20
ON25 THP	CCU CUU ACC UCA GUU ACA	1	THP	6180.98	6181.40
ON26 THP	CCU CUU ACC UCA GUU ACA	2	THP	6263.08	6247.53
ON27 THP	CCU CUU ACC UCA GUU ACA	2	THP	6263.08	6263.50
ON28 THP	CCU CUU ACC UCA GUU ACA	3	THP	6345.18	6345.40
ON29 THP	CCU CUU ACC UCA GUT ACA	1	THP	6195.01	6195.60
ON30 THP	CCU CUT ACC UCA GUT ACA	2	THP	6291.14	6291.70
ON31 THP	CCT CUT ACC TCA GUT ACA	4	THP	6483.39	6483.40
ON32 THP	CCT CTT$_{X}$ ACC TCA GTT ACA	6	THP	6675.65	6676.00

ON33 THP	CCT CUT ACC TCA GUT ACA	7	THP	6729.70	6729.90
ON34 THP	CCT CTT ACC TCA GTT ACA	9	THP	6921.96	6921.80
ON35 C16	CCU CUU ACC UCA GUU ACA	1	C16	6321.29	6321.12
ON36 C16	CCU CUU ACC UCA GUU ACA	2	C16	6543.71	ESI-MS[b] $[M+3H]^{3+}$ 2182.04
ON37 C16	CCU CUU ACC UCA GUU ACA	2	C16	6543.71	ESI-MS[b] $[M+3H]^{3+}$ 2182.04
ON38 C16	CCU CUU ACC UCA GUU ACA	3	C16	6766.13	ESI-MS[b] $[M+3H]^{3+}$ 2256.12
ON39 Hexynyl	CCU CUU ACC UCA GUU ACA	1	Hexynyl	6176.99	6176.80
ON40 Hexynyl	CCU CUU ACC UCA GUU ACA	2	Hexynyl	6255.10	6255.10
ON41 Hexynyl	CCU CUU ACC UCA GUU ACA	3	Hexynyl	6333.22	6332.30
ONOX1 iPr	CCUCUUACCUCAGUT_oACA	1	^{i}Pr	6136.91	6137.00
ONOX4 iPr	CCT_oCUT_oACCT_oCAGUT_oACA	4	^{i}Pr	6251.00	6251.00
ON42	TGT AAC TGA GGT AAG AGG (target DNA)	--	--	5627.71	5628.40
ON43	UGU AAC UGA GGU AAG AGG (target RNA)	--	--	5859.59	5860.30
ON44	CCU CUU ACC UCA GUU ACA (2´-OMe-PS Control)	--	--	6098.88	6098.70

[a]Oligonucleotides contain only phosphorothioate internucleotide linkages, as tert-butyl groups cleaved during the oligonucleotide synthesis. Hydrolysis of the phosphorothioate group to phosphodiester was observed for **ON17-ON24.**

[b]We were unable to obtain UPLC-MS data of **ON36-ON38** as they are highly lipophilic so we obtained ESI-MS data

2.1 MeOPr PTTE oligonucleotides

ON1 MeOPr	CCU CUU ACC UCA GUU ACA	**MeOPr**	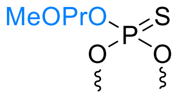

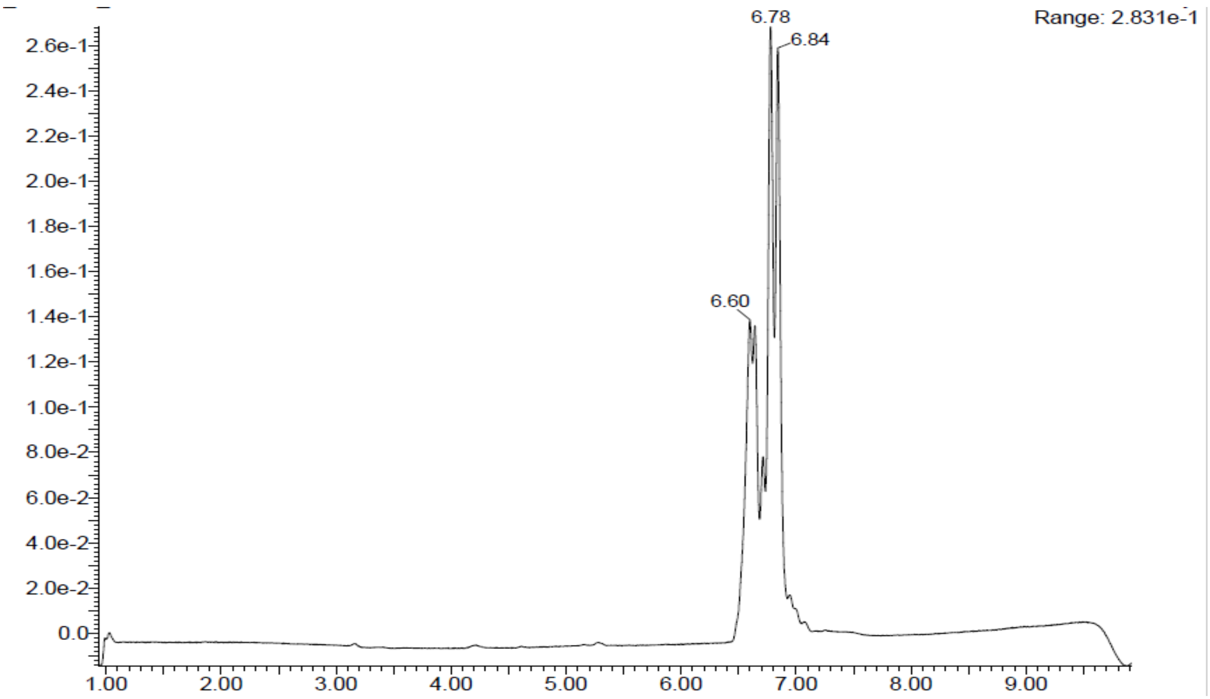

Supplementary Figure S4: Reverse-phase UPLC of **DMTON ON1** (UV absorbance at 260 nm vs time in min).

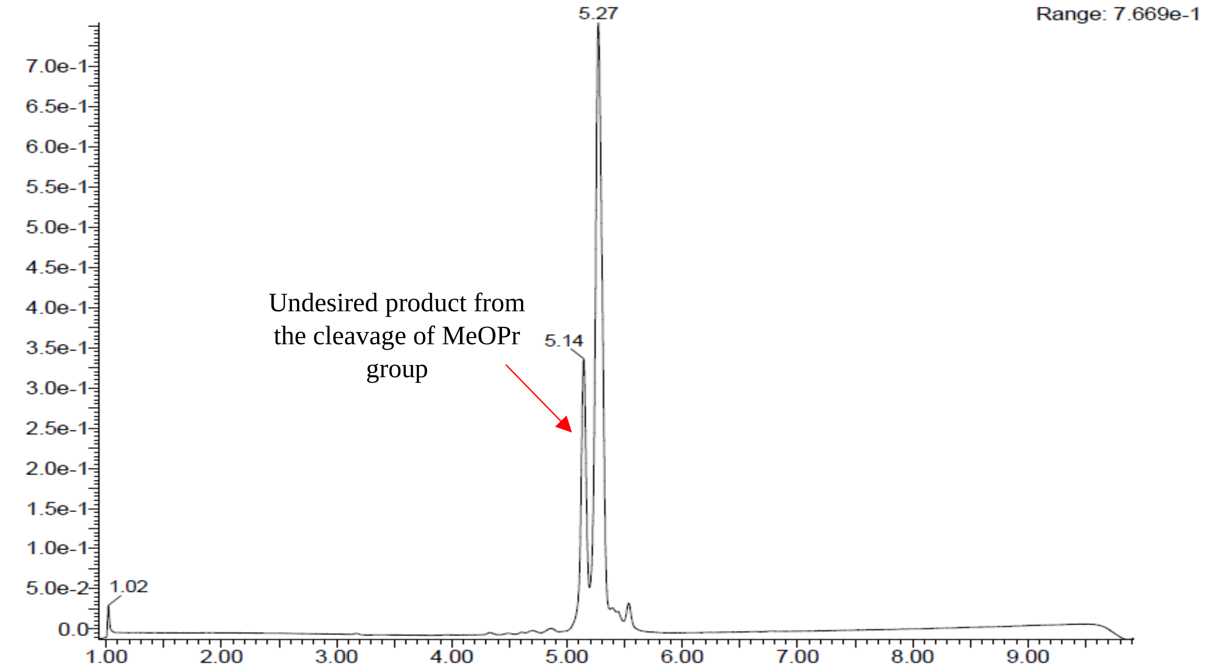

Supplementary Figure S5: Reverse-phase UPLC of **crude ON1** after removal of DMT group (UV absorbance at 260 nm vs time in min).

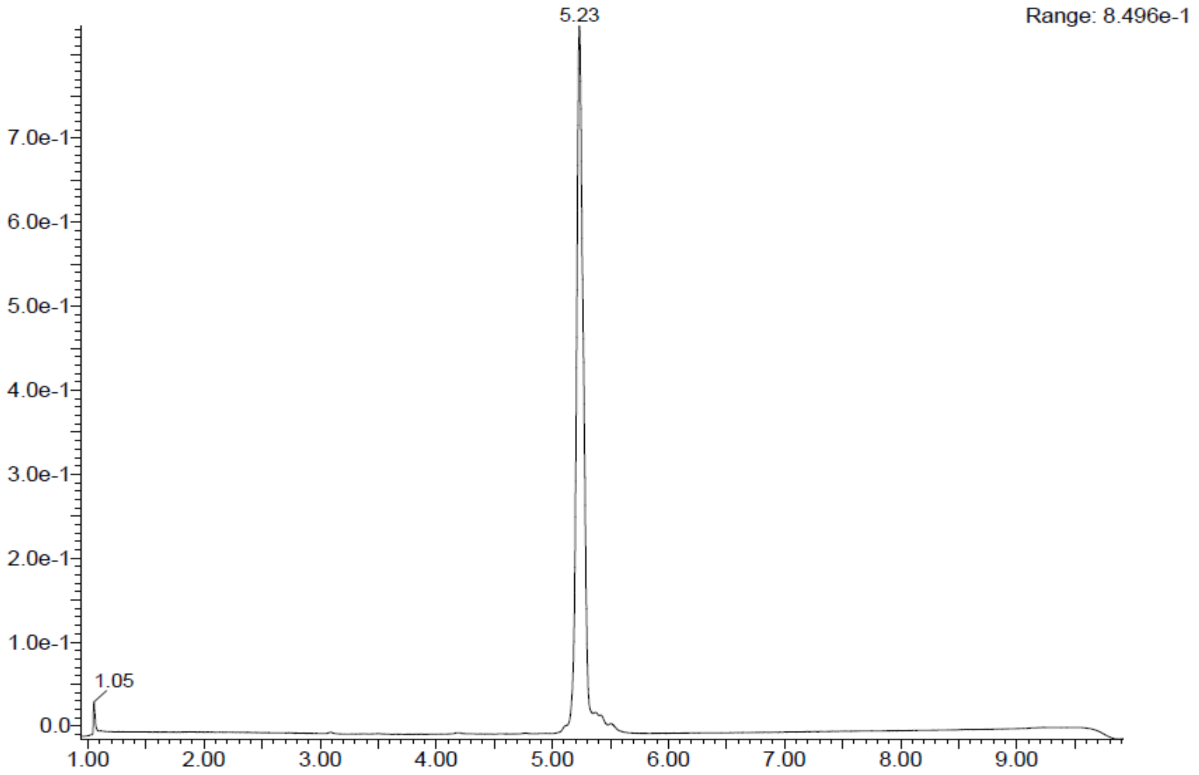

Supplementary Figure S6: Reverse-phase UPLC of **ON1** after HPLC purification (UV absorbance at 260 nm vs time in min).

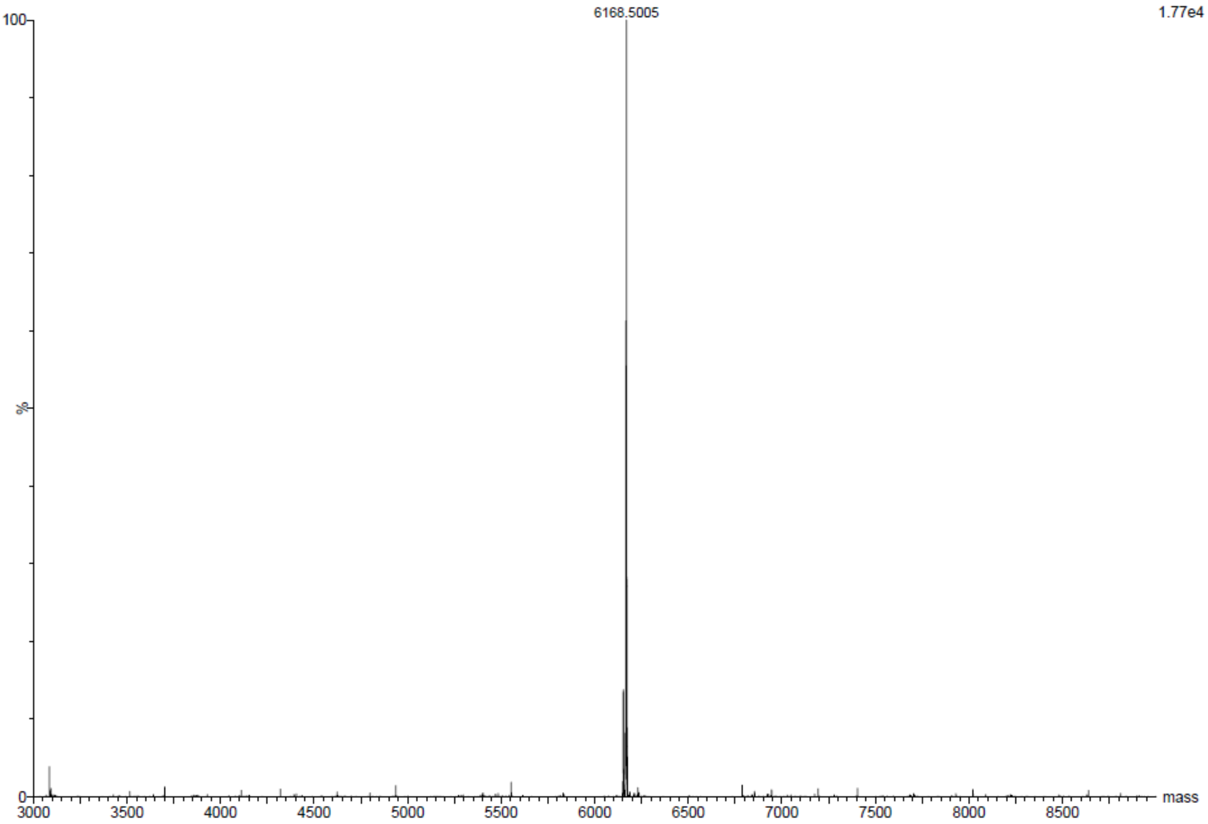

Supplementary Figure S7: Mass spectrum (ES-) of **ON1**. Required **6168.97** Da, found **6168.50** Da. y-axis = relative intensity (%), x-axis = mass in Da.

ON2 MeOPr	CCU CUU ACC UCA GUU ACA	**2.MeOPr**	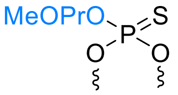

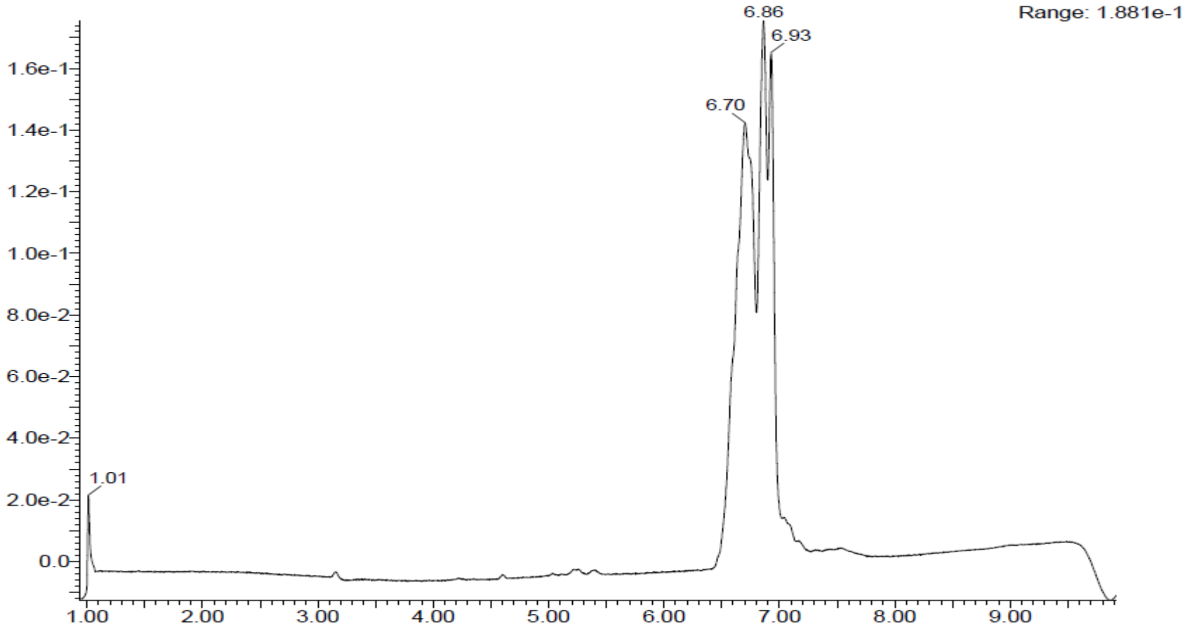

Supplementary Figure S8: Reverse-phase UPLC of **DMTON ON2** (UV absorbance at 260 nm vs time in min).

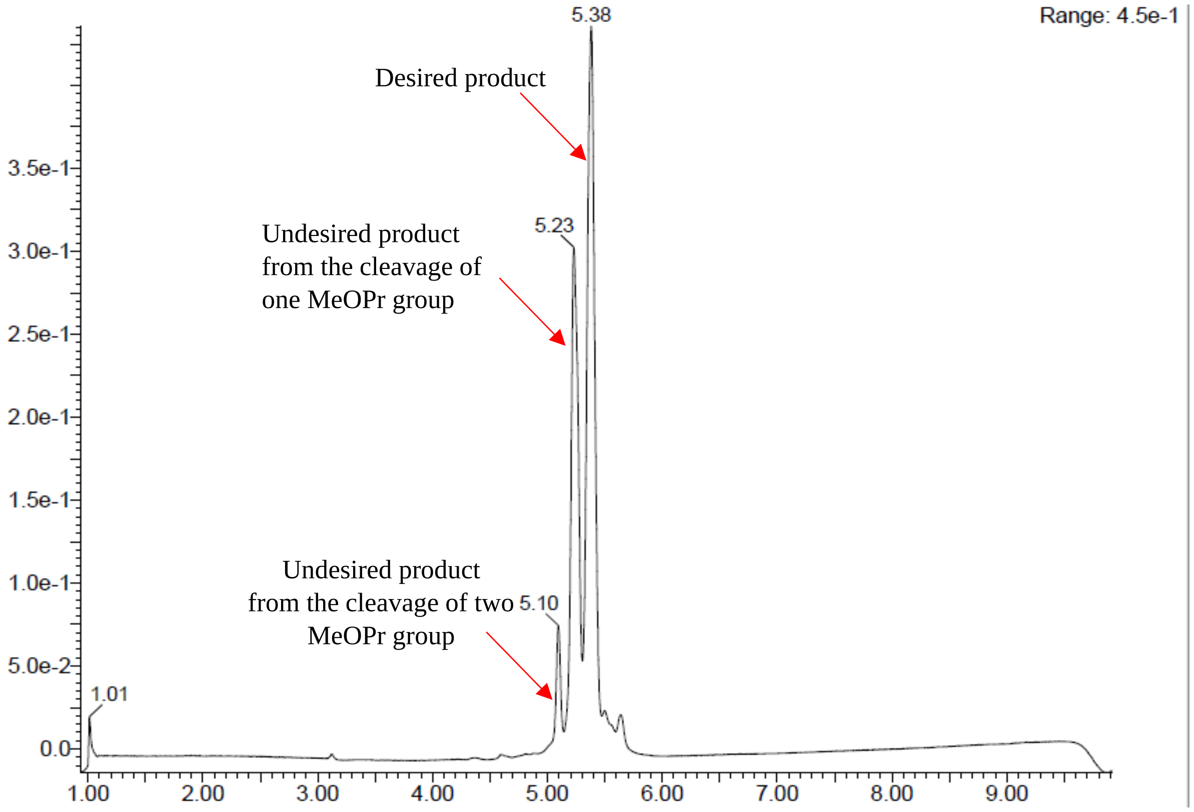

Supplementary Figure S9: Reverse-phase UPLC of **crude ON2** after removal of the DMT group (UV absorbance at 260 nm vs time in min).

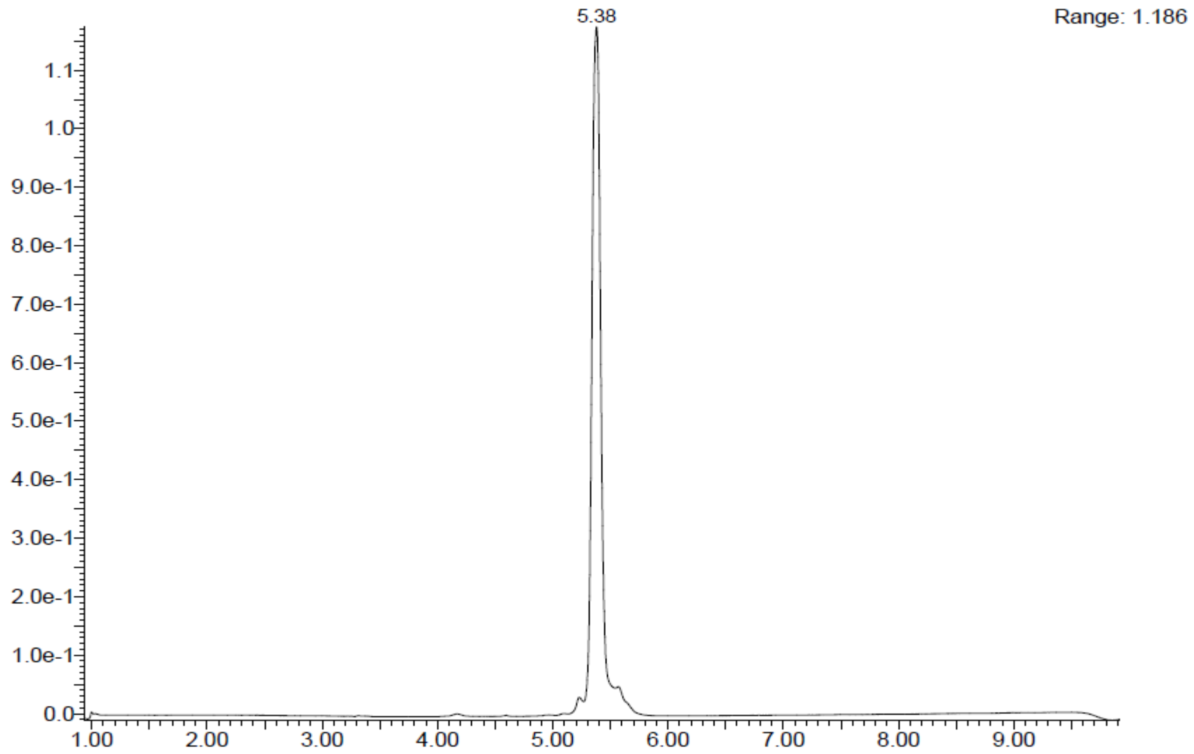

Supplementary Figure S10: Reverse-phase UPLC of **ON2** after HPLC purification (UV absorbance at 260 nm vs time in min).

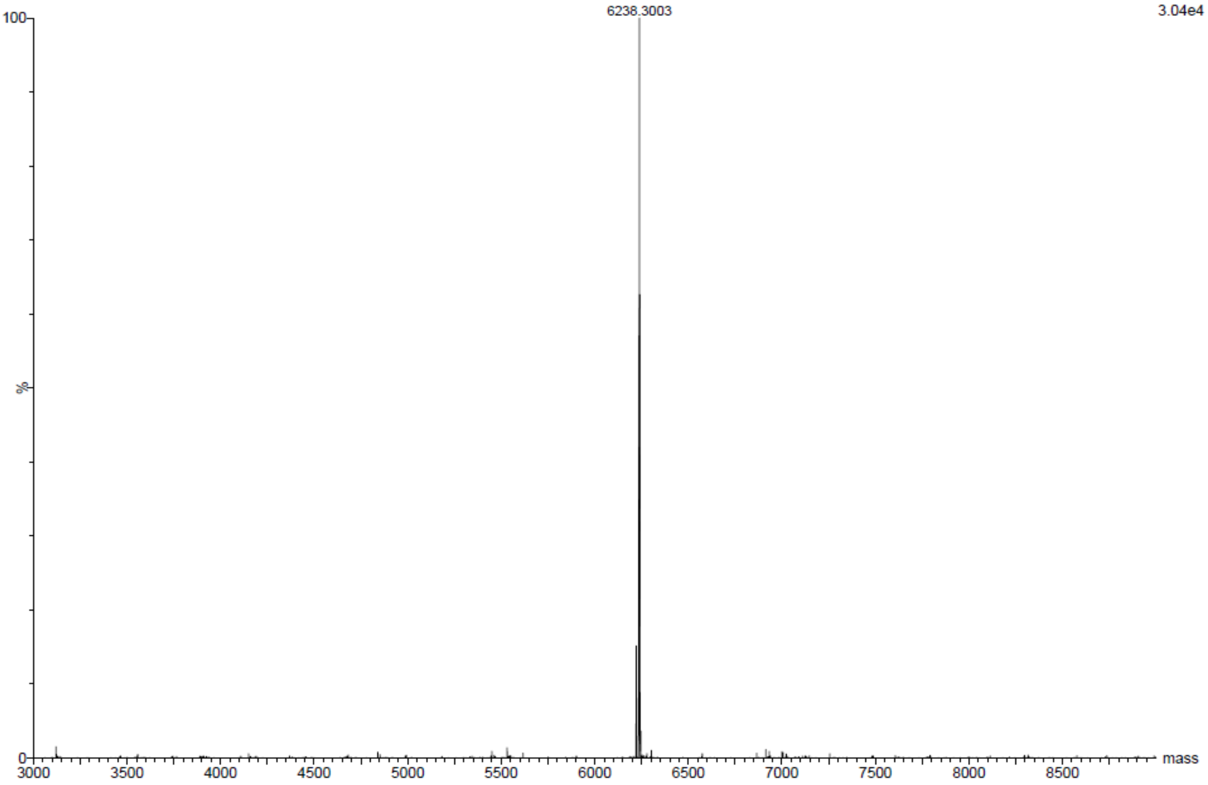

Supplementary Figure S11: Mass spectrum (ES-) of **ON2**. Required **6239.06** Da, found **6238.30** Da. y-axis = relative intensity (%), x-axis = mass in Da.

ON3 MeOPr	CCU CUU ACC UCA GUU ACA	**2.MeOPr**	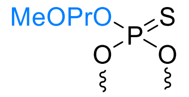

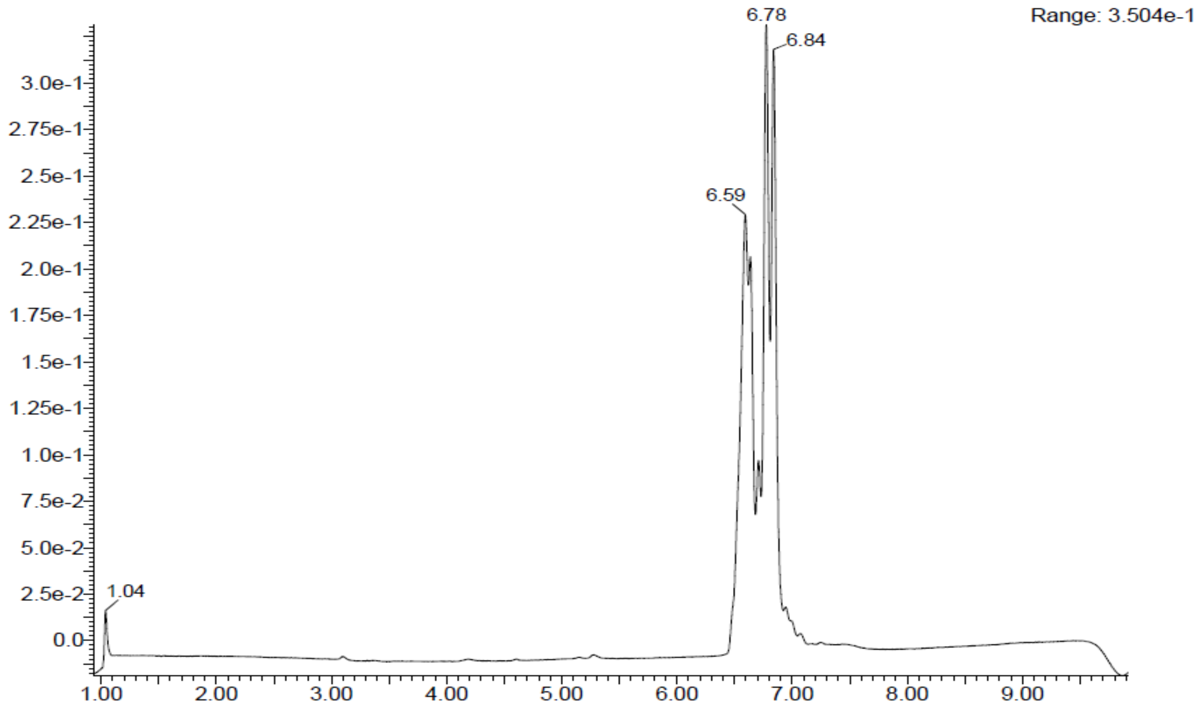

Supplementary Figure S12: Reverse-phase UPLC of **DMTON ON3** (UV absorbance at 260 nm vs time in min).

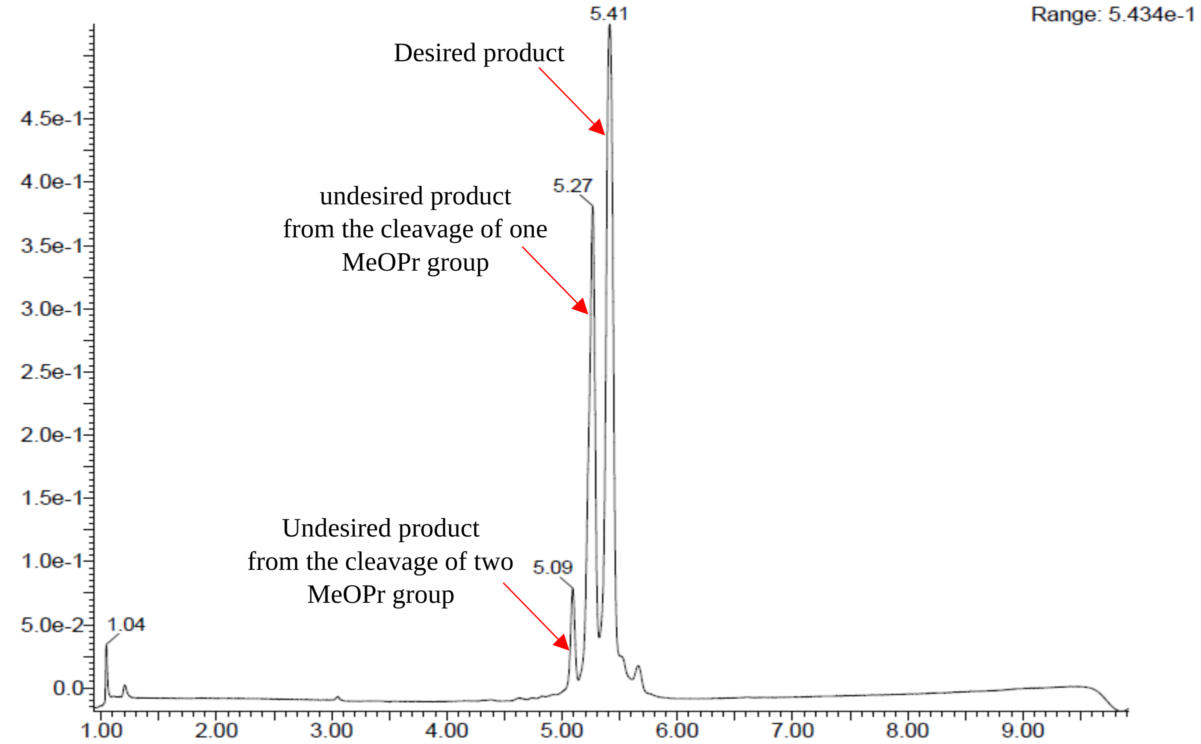

Supplementary Figure S13: Reverse-phase UPLC of **crude ON3** after removal of DMT group (UV absorbance at 260 nm vs time in min).

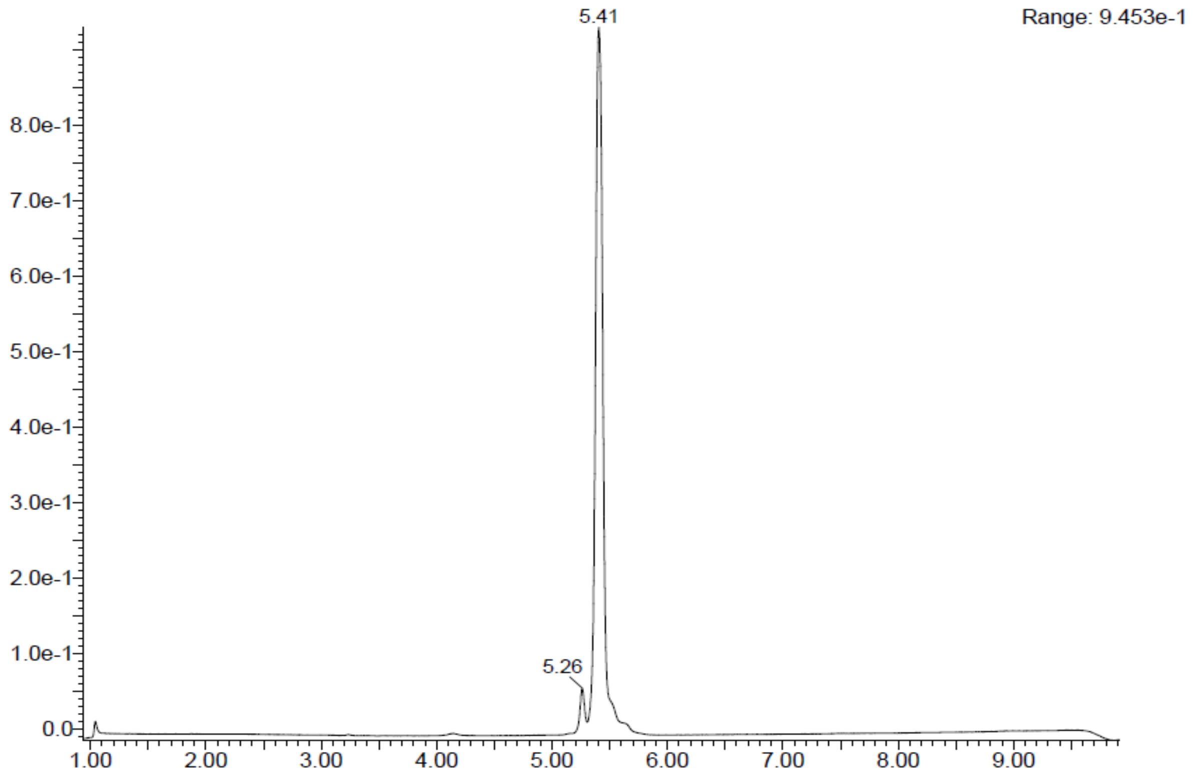

Supplementary Figure S14: Reverse-phase UPLC of **ON3** after HPLC purification (UV absorbance at 260 nm vs time in min).

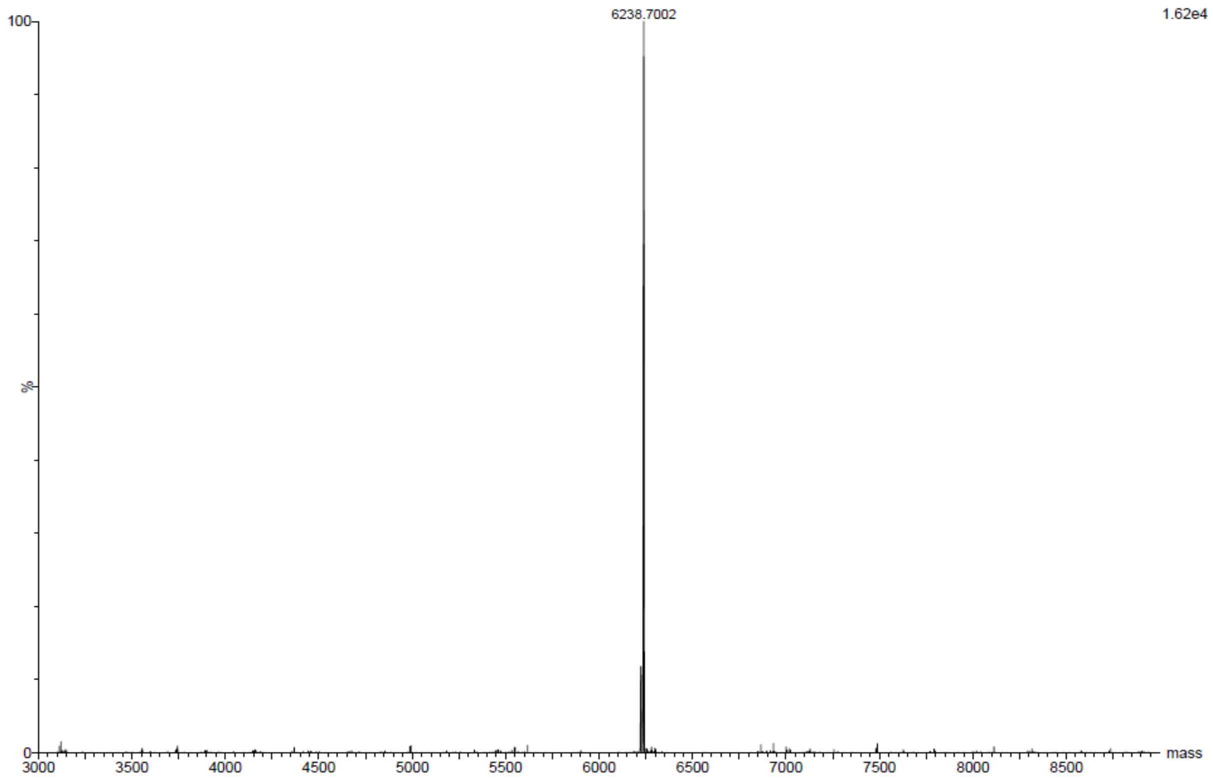

Supplementary Figure S15: Mass spectrum (ES-) of **ON3**. Required **6239.06** Da, found **6238.70** Da. y-axis = relative intensity (%), x-axis = mass in Da.

ON4 MeOPr	CCU CUU ACC UCA GUU ACA	**3.MeOPr**	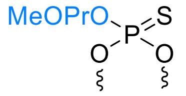

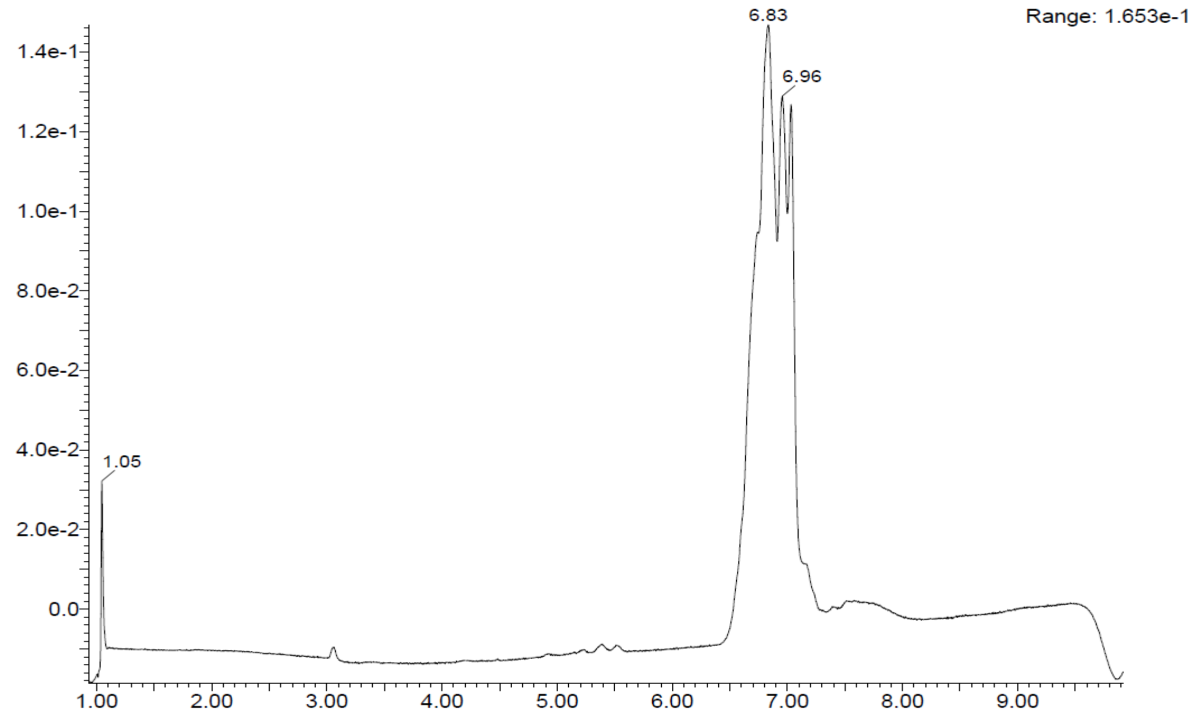

Supplementary Figure S16: Reverse-phase UPLC of **DMTON ON4** (UV absorbance at 260 nm vs time in min).

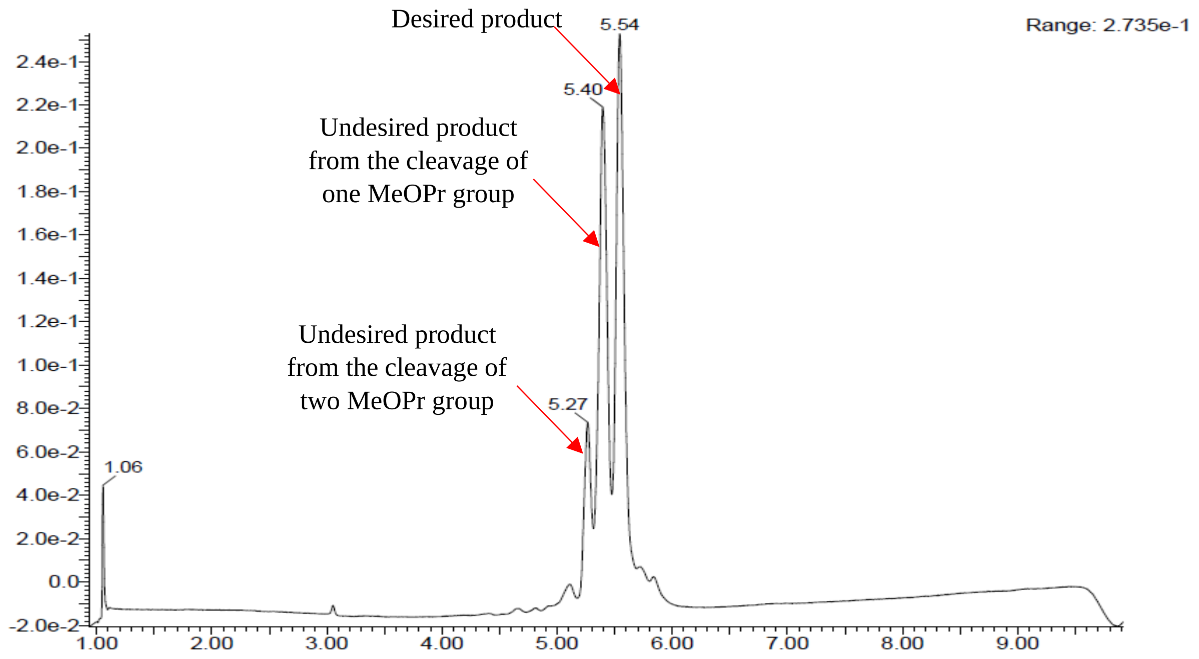

Supplementary Figure S17: Reverse-phase UPLC of crude **ON4** after removal of the DMT group (UV absorbance at 260 nm vs time in min).

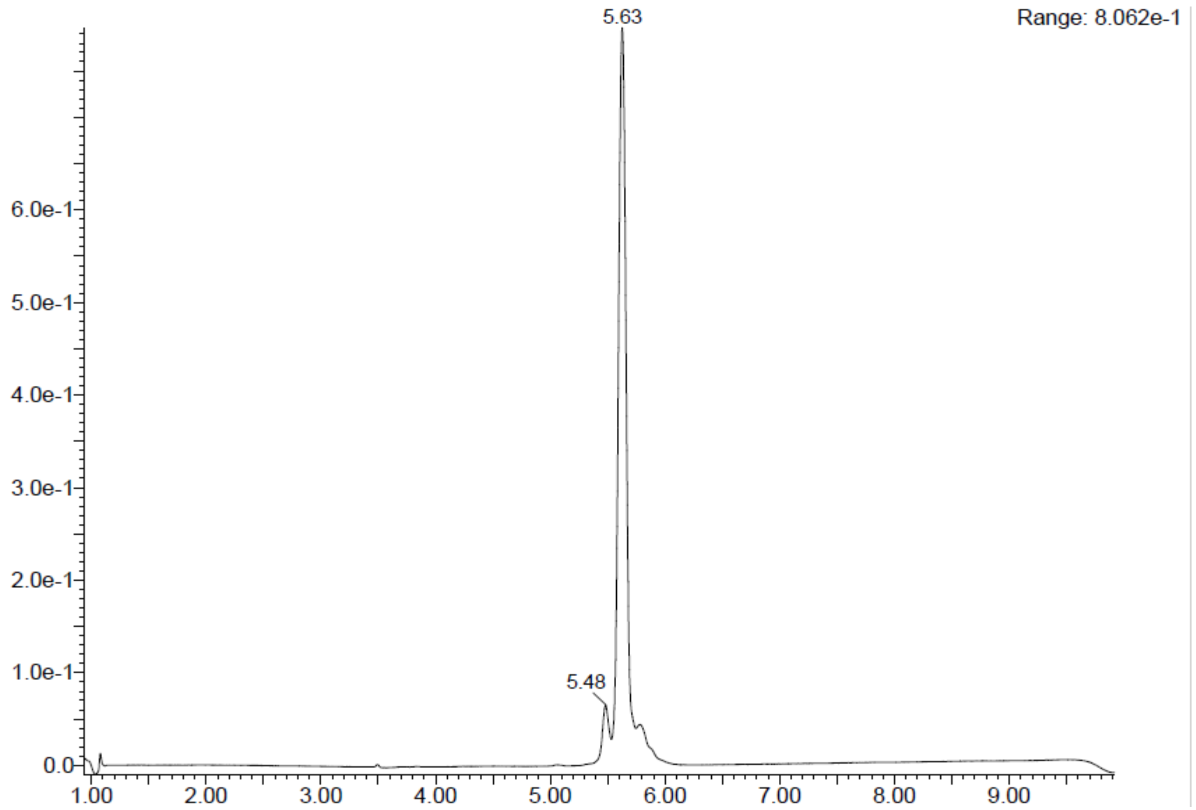

Supplementary Figure S18: Reverse-phase UPLC of **ON4** after HPLC purification (UV absorbance at 260 nm vs time in min).

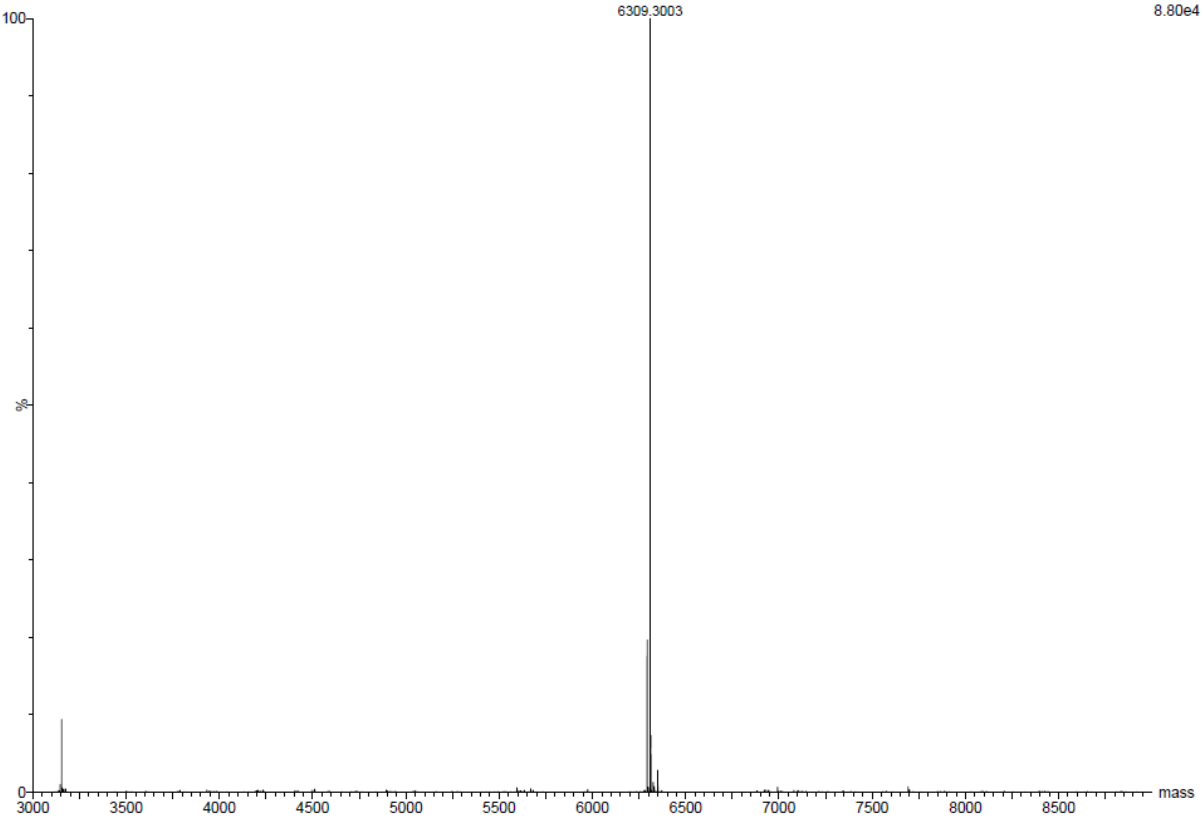

Supplementary Figure S19: Mass spectrum (ES-) of **ON4**. Required **3609.15** Da, found **6309.30** Da. y-axis = relative intensity (%), x-axis = mass in Da.

ON5 MeOPr	CCU CUU ACC UCA GUT ACA	**MeOPr**	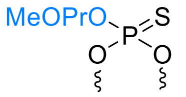

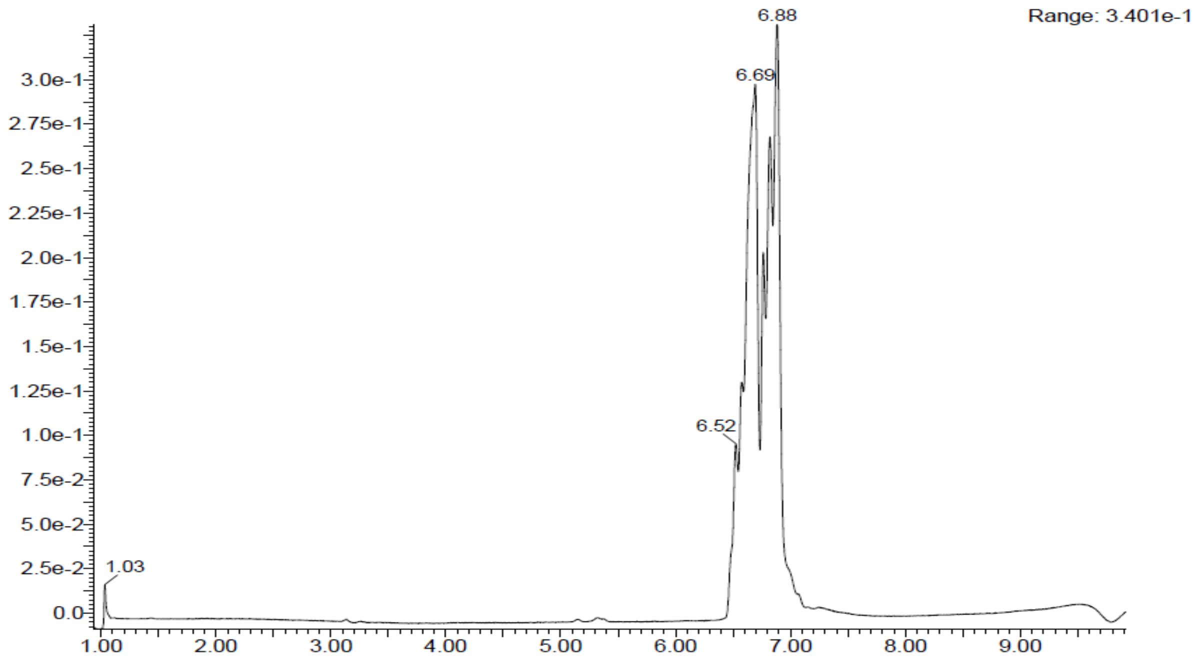

Supplementary Figure S20: Reverse-phase UPLC of **DMTON ON5** (UV absorbance at 260 nm vs time in min).

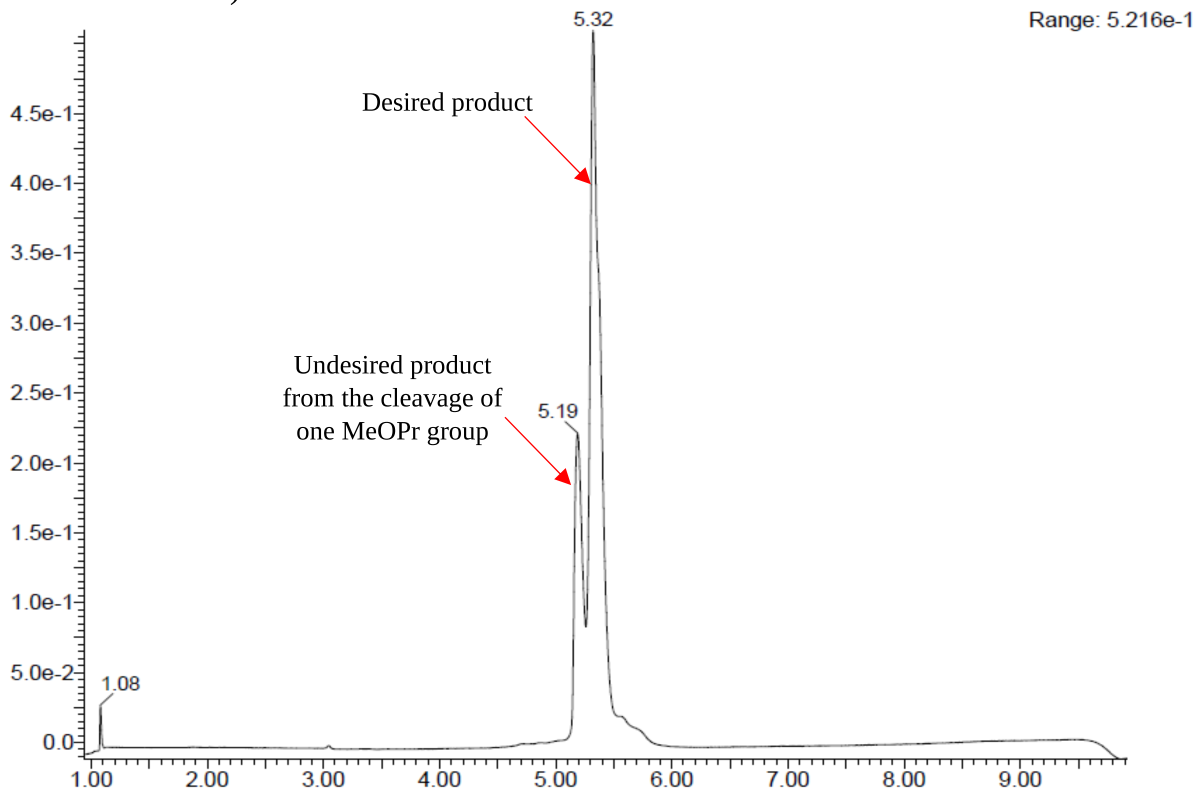

Supplementary Figure S21: Reverse-phase UPLC of crude **ON5** after removal of the DMT group (UV absorbance at 260 nm vs time in min).

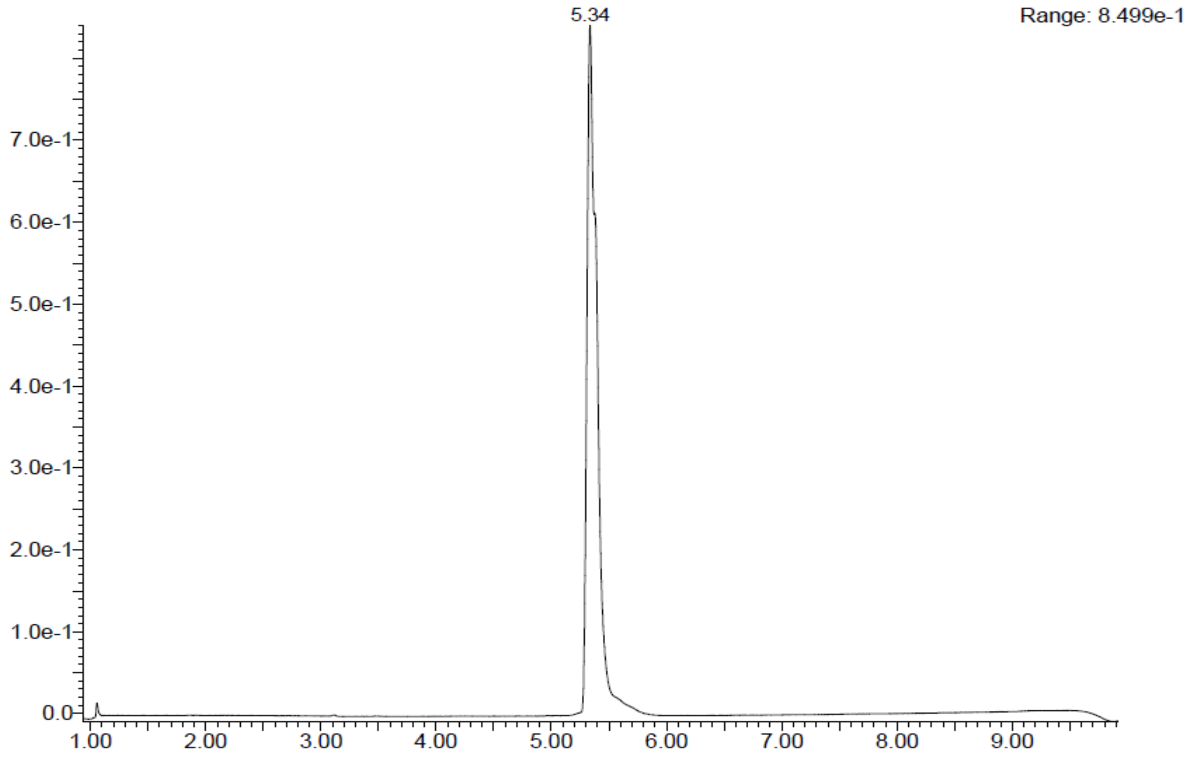

Supplementary Figure S22: Reverse-phase UPLC of **ON5** after HPLC purification (UV absorbance at 260 nm vs time in min).

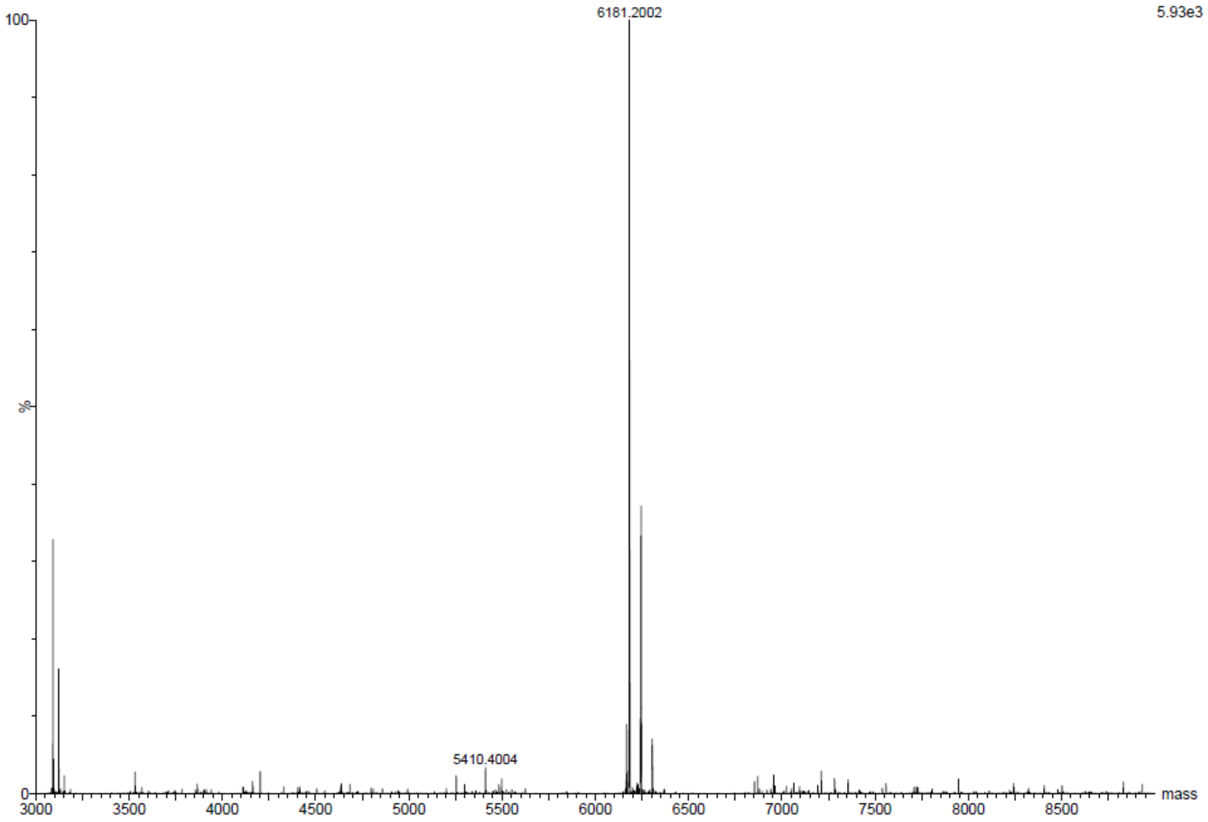

Supplementary Figure S23: Mass spectrum (ES-) of final **ON5**. Required **6183.00** Da, found **6181.20** Da. y-axis = relative intensity (%), x-axis = mass in Da.

ON6 MeOPr	CCU CUT ACC UCA GUT ACA	**2.MeOPr**	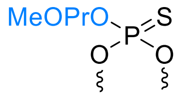

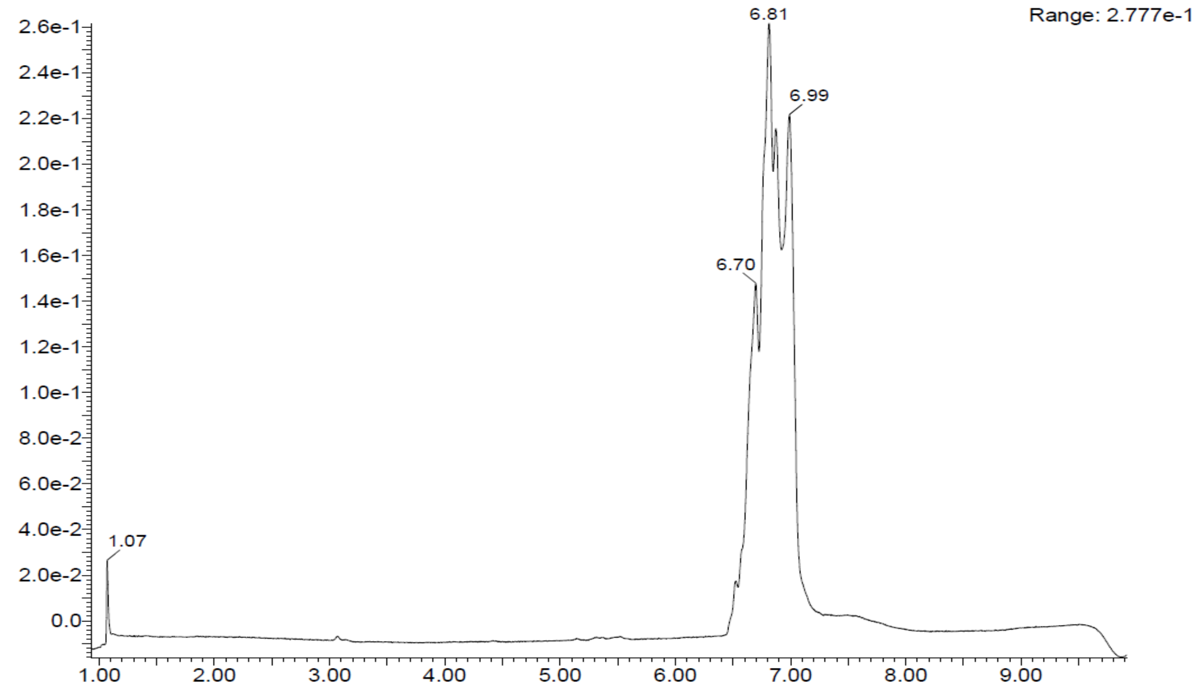

Supplementary Figure S24: Reverse-phase UPLC of **DMTON ON6** (UV absorbance at 260 nm vs time in min).

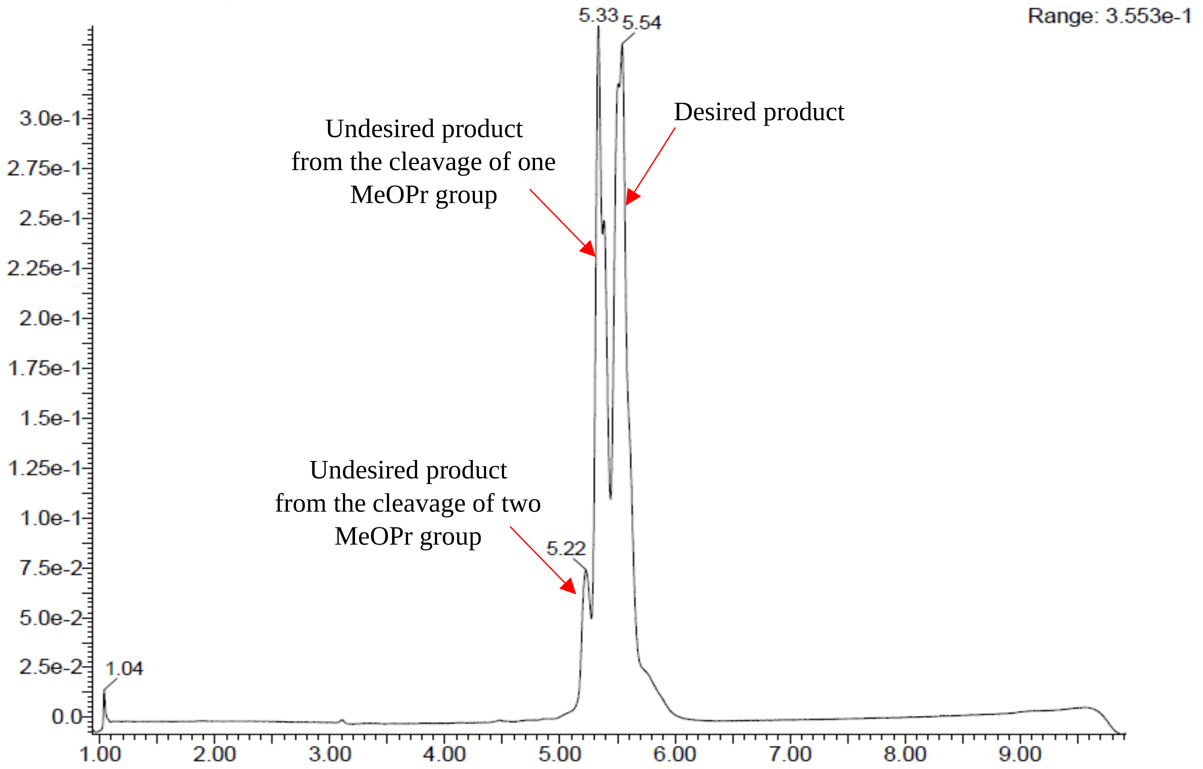

Supplementary Figure S25: Reverse-phase UPLC of **crude ON6** after removal of the DMT group (UV absorbance at 260 nm vs time in min).

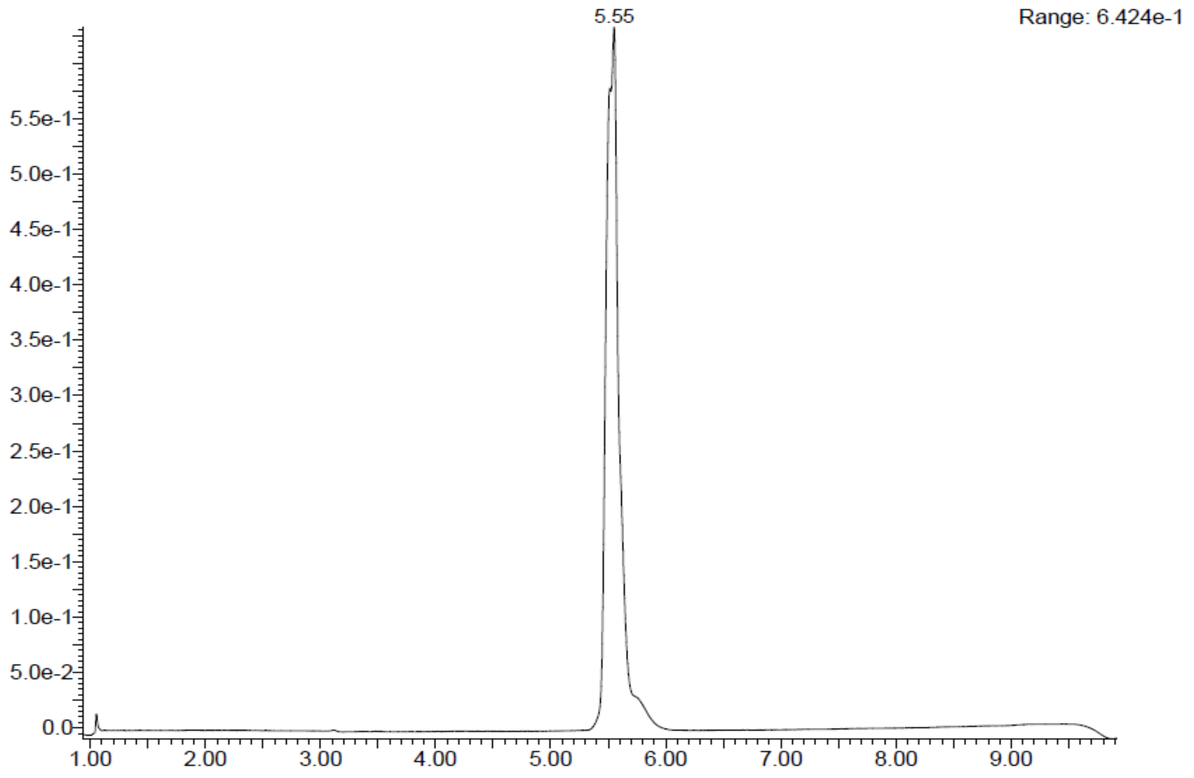

Supplementary Figure S26: Reverse-phase UPLC of **ON6** after HPLC purification (UV absorbance at 260 nm vs time in min).

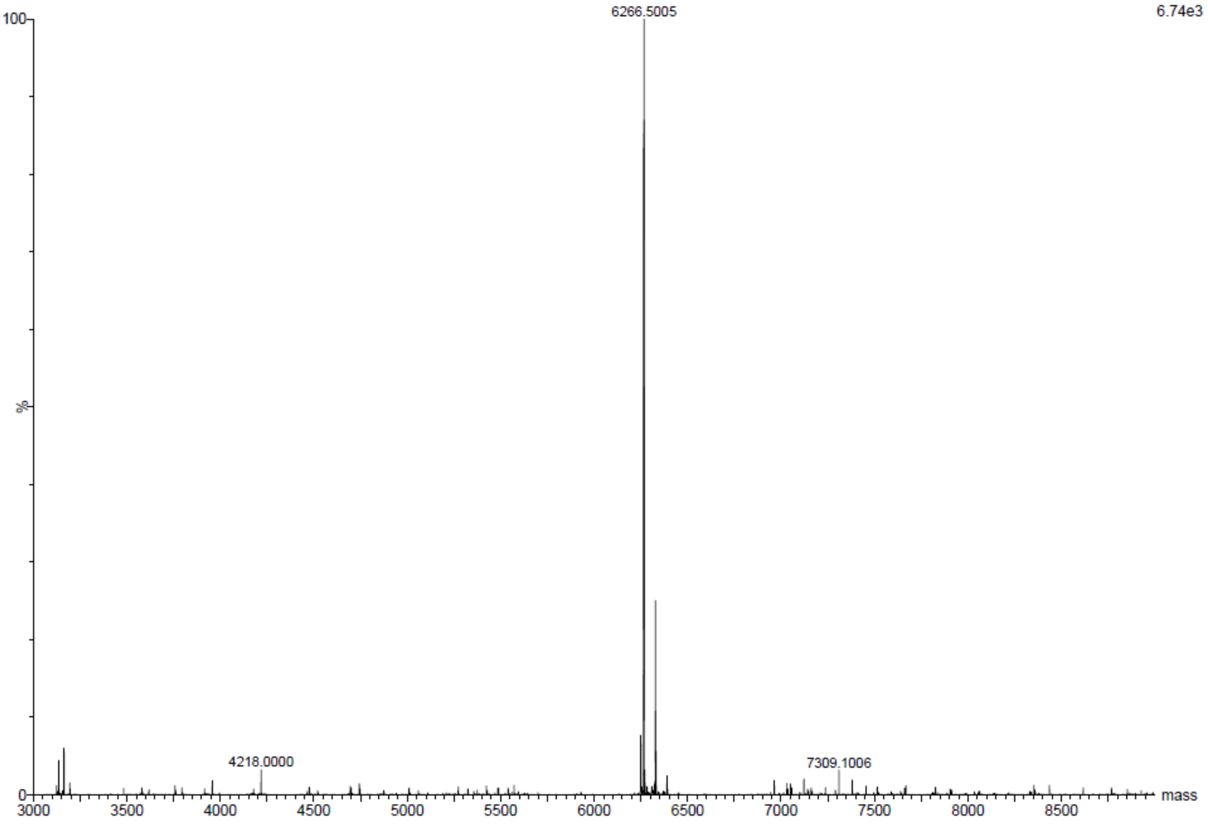

Supplementary Figure S27: Mass spectrum (ES-) of final **ON6**. Required **6267.11** Da, found **6266.50** Da. y-axis = relative intensity (%), x-axis = mass in Da.

ON7 MeOPr	CCT CUT ACC TCA GUT ACA	**4.MeOPr**	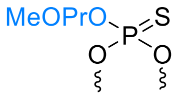

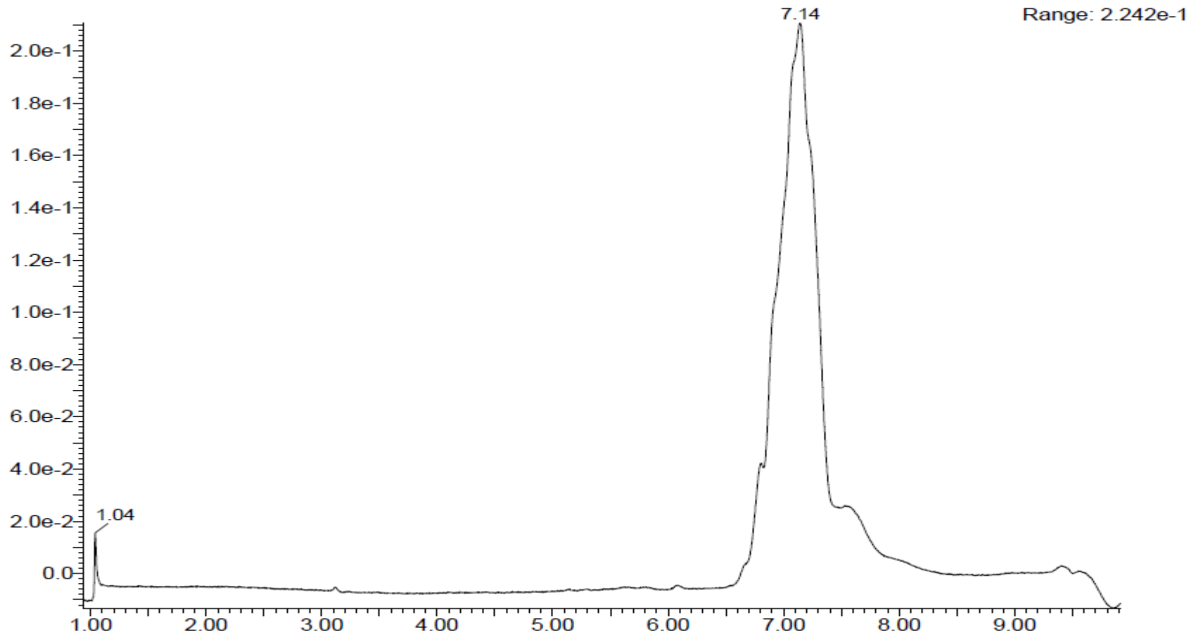

Supplementary Figure S28: Reverse-phase UPLC of **DMTON ON7** (UV absorbance at 260 nm vs time in min).

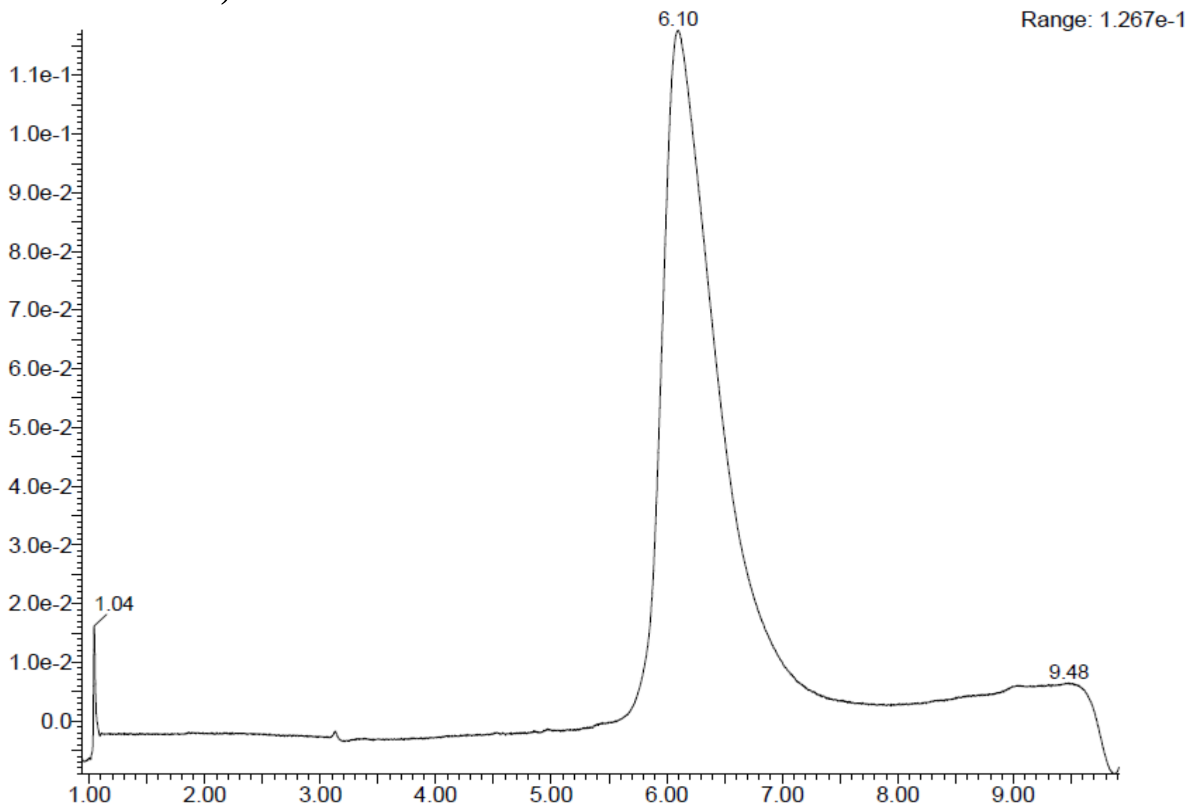

Supplementary Figure S29: Reverse-phase UPLC of crude **ON7** after removal of the DMT group (UV absorbance at 260 nm vs time in min). Undesired products are not seen and separable. Peaks with highest retention time was collected to get the desired product **ON7**.

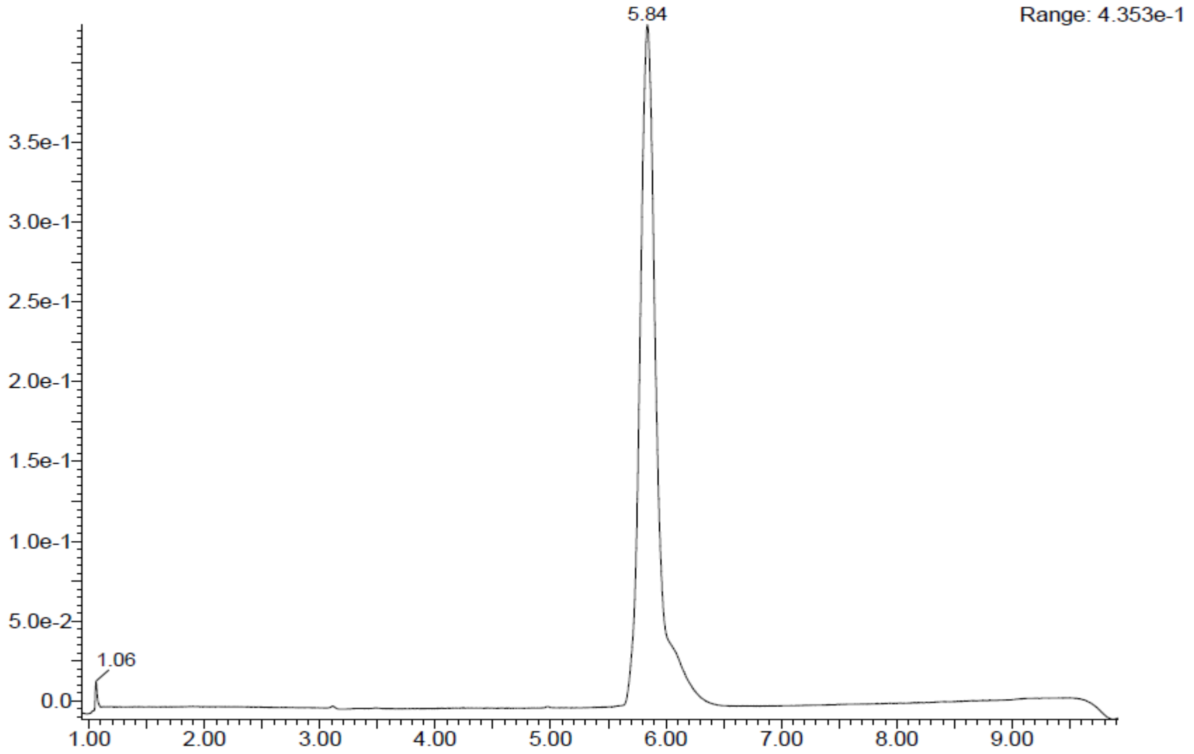

Supplementary Figure S30: Reverse-phase UPLC of **ON7** after HPLC purification (UV absorbance at 260 nm vs time in min).

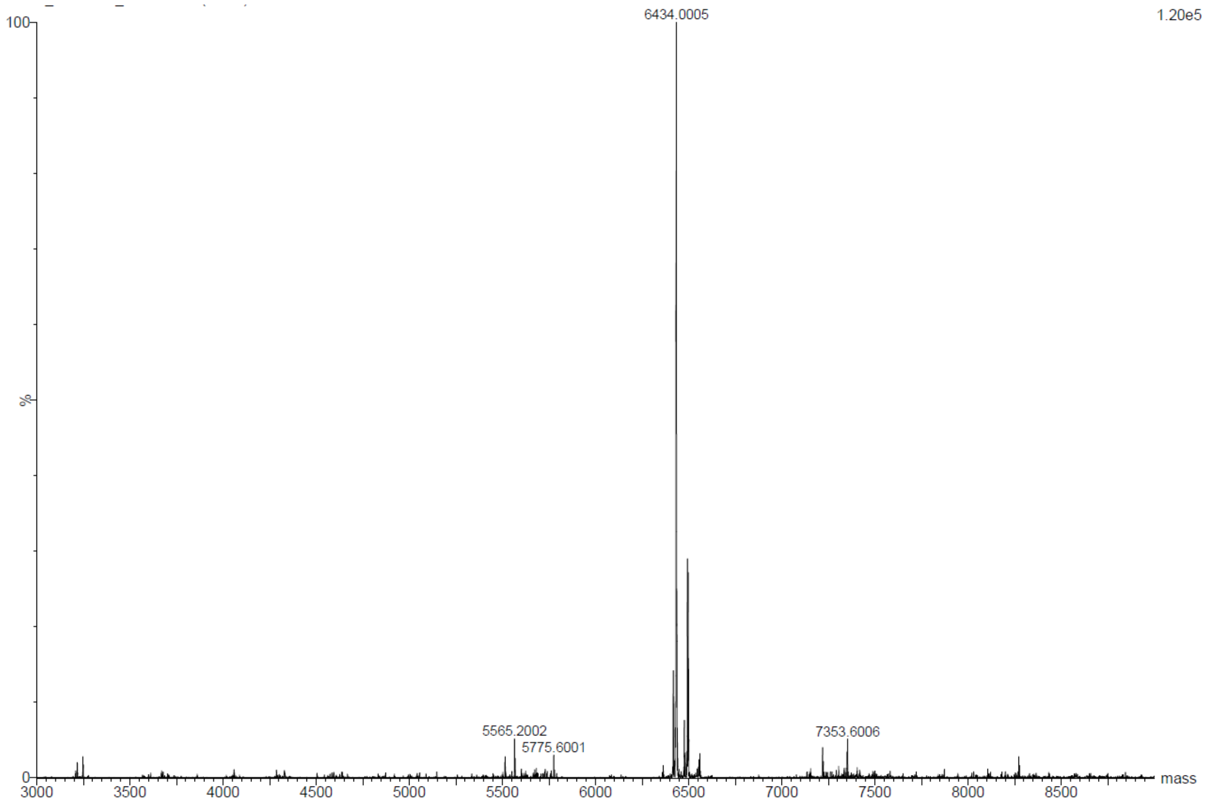

Supplementary Figure S31: Mass spectrum (ES-) of final **ON7**. Required **6435.35** Da, found **6434.00** Da. y-axis = relative intensity (%), x-axis = mass in Da.

ON8 MeOPr	CCT CTT ACC TCA GTT ACA	**6.MeOPr**	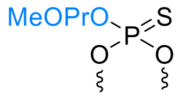

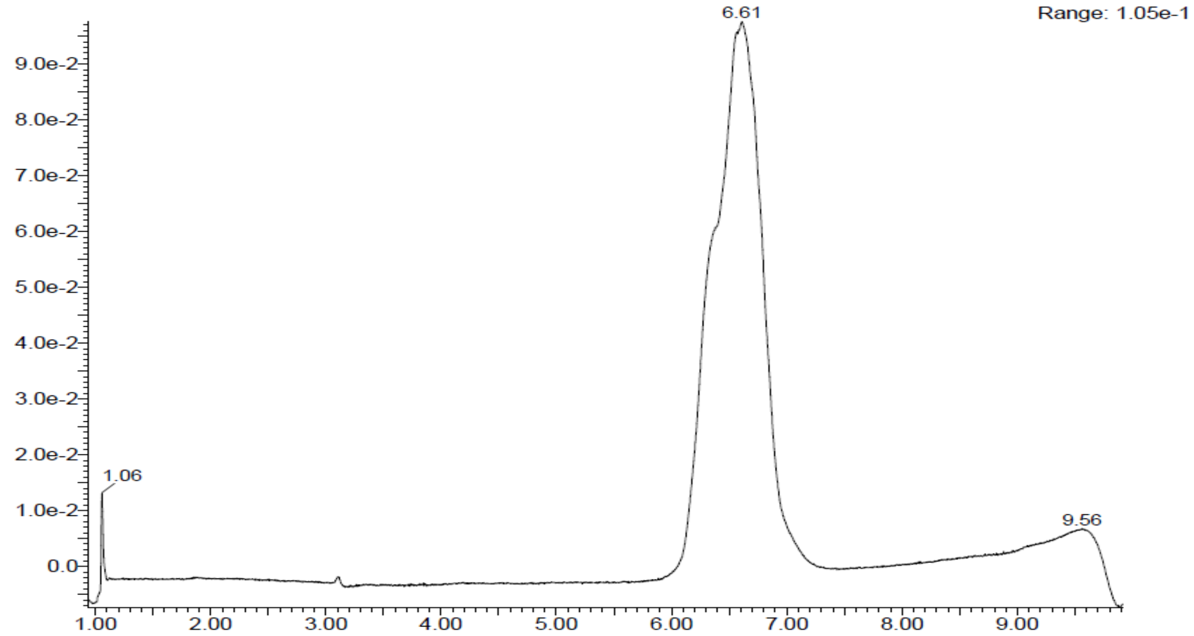

Supplementary Figure S32: Reverse-phase UPLC of **ON8** after HPLC purification (UV absorbance at 260 nm vs time in min).

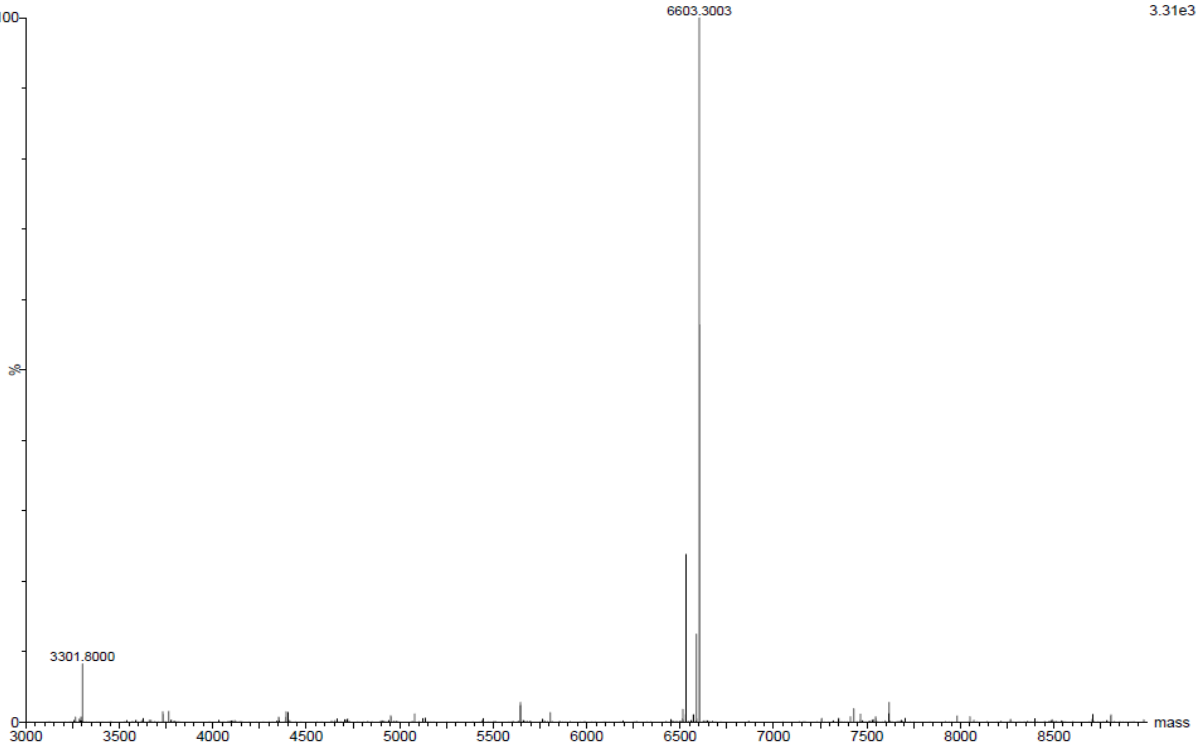

Supplementary Figure S33: Mass spectrum (ES-) of **ON8** after HPLC purification. Required **6603.59** Da, found **6603.30** Da. y-axis = relative intensity (%), x-axis = mass in Da.

2.2 ^{i}Pr PTTE oligonucleotides

ON9 iPr	CCU CUU ACC UCA GUU ACA	^{i}Pr	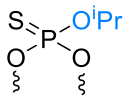

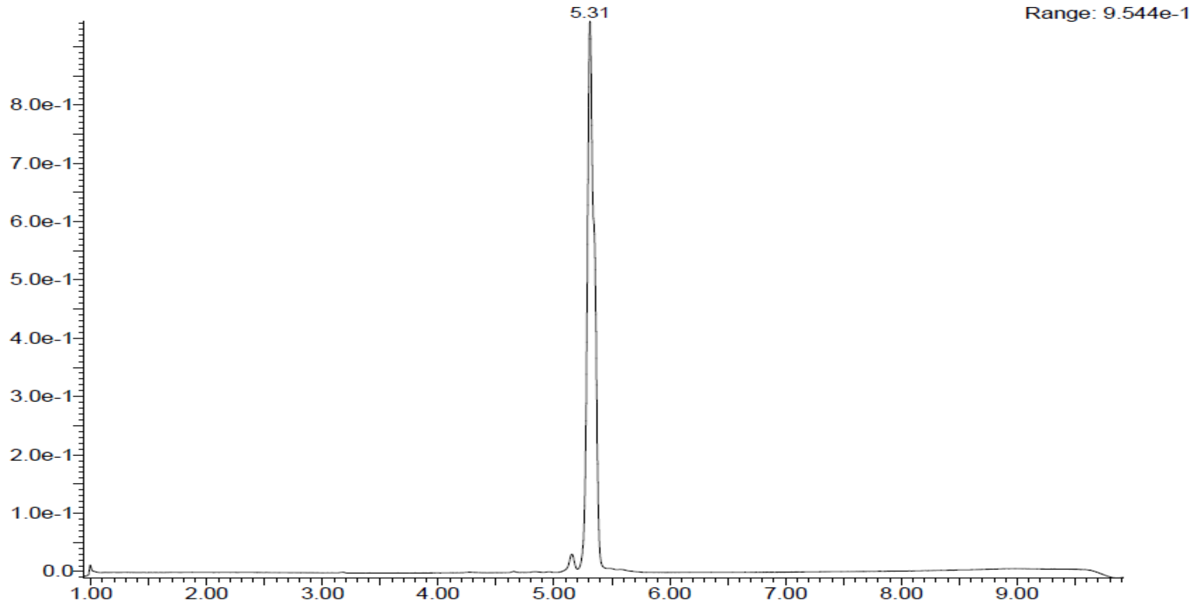

Supplementary Figure S34: Reverse-phase UPLC of crude **ON9** after DMT group removal without purification (UV absorbance at 260 nm vs time in min).

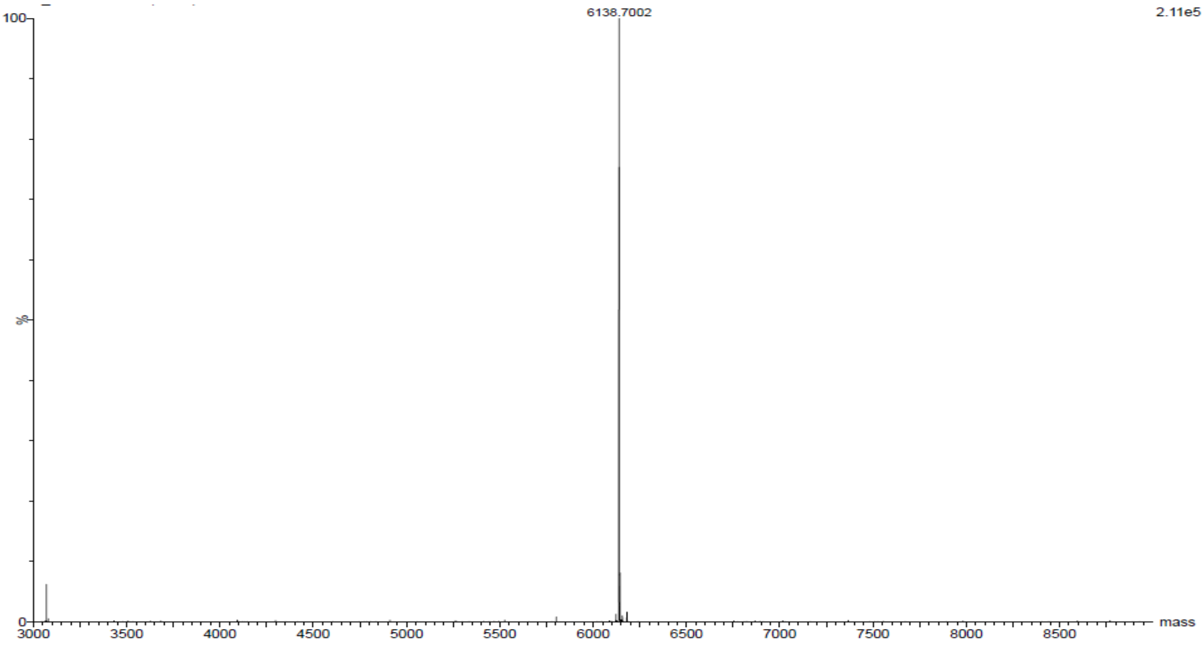

Supplementary Figure S35: Mass spectrum (ES-) of crude **ON9** after DMT group removal without purification. Required **6138.94** Da, found **6138.70** Da. y-axis = relative intensity (%), x-axis = mass in Da.

ON10 iPr	CCU CUU ACC UCA GUU ACA	**2.^{i}Pr**	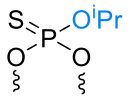

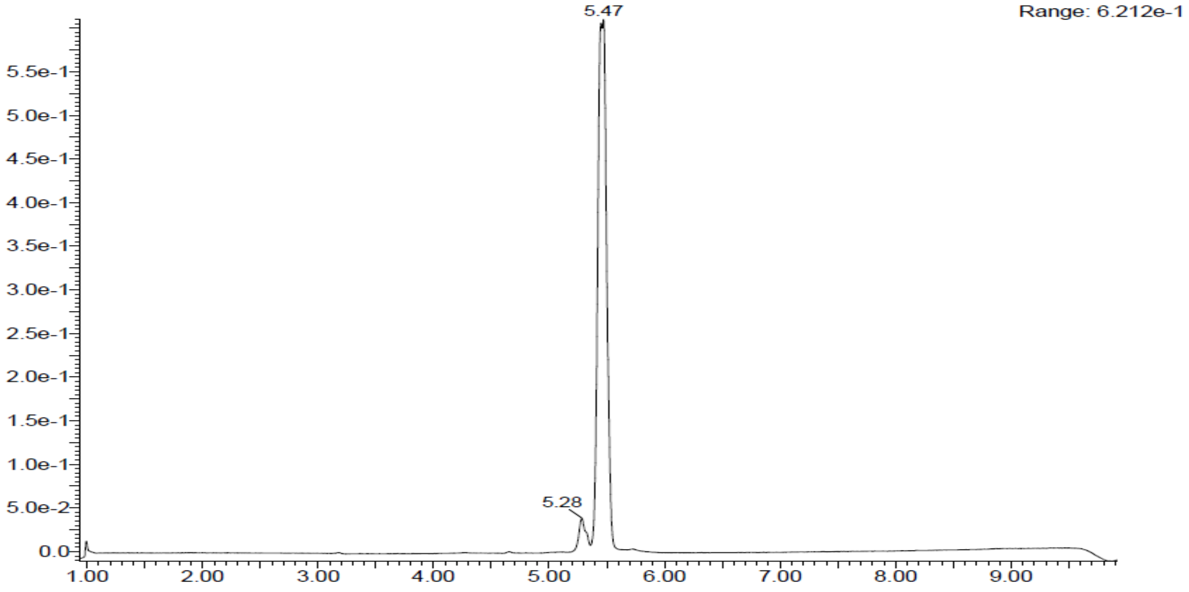

Supplementary Figure S36: Reverse-phase UPLC of crude **ON10** after DMT group removal without purification (UV absorbance at 260 nm vs time in min).

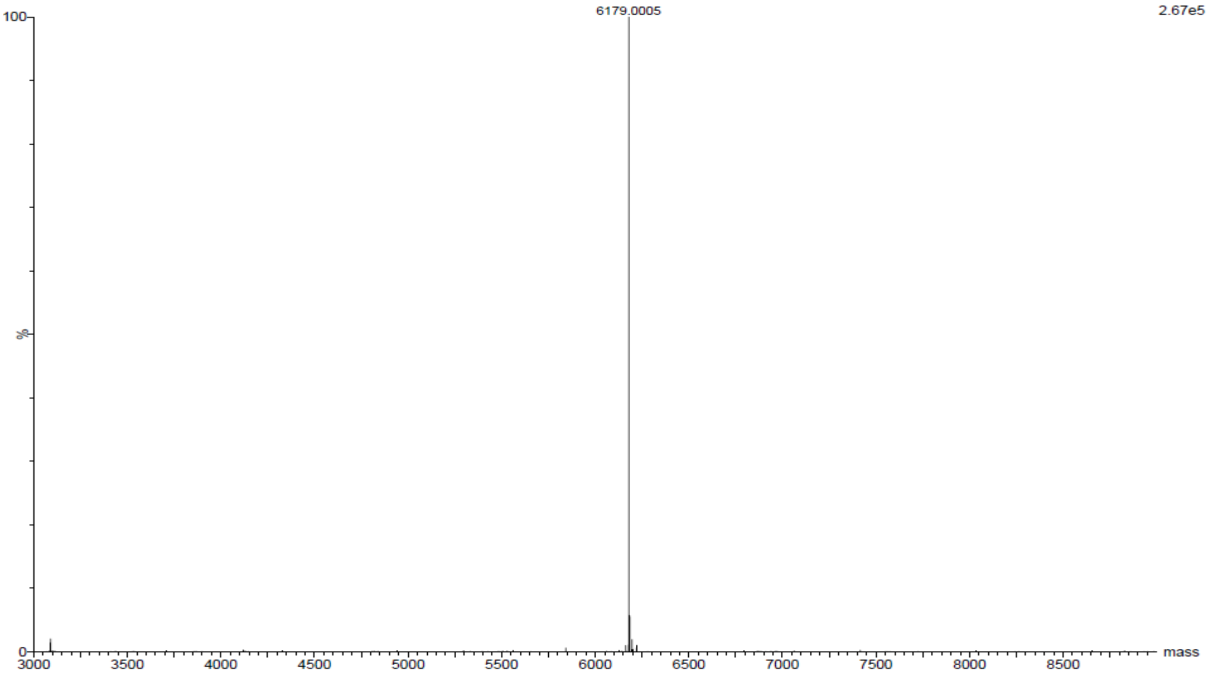

Supplementary Figure S37: Mass spectrum (ES-) of crude **ON10** after DMT group removal without purification. Required **6179.01** Da, found **6179.00** Da. y-axis = relative intensity (%), x-axis = mass in Da.

ON11 iPr	CCU CUU ACC UCA GUU ACA	**2.^{i}Pr**	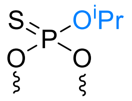

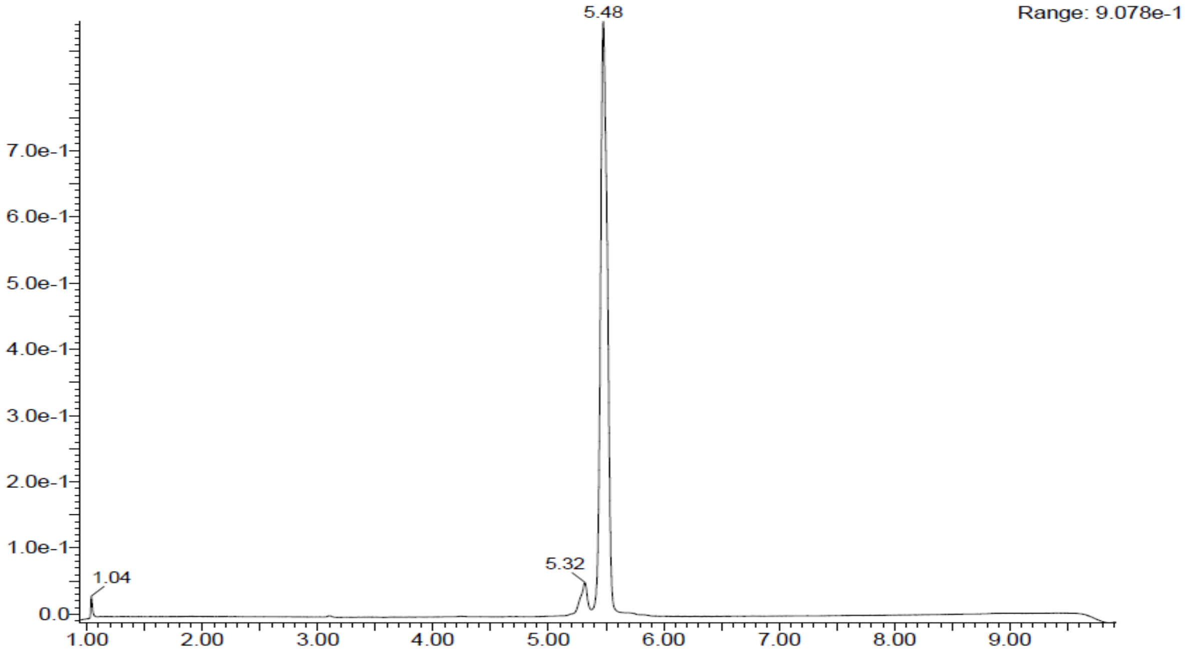

Supplementary Figure S38: Reverse-phase UPLC of crude **ON11** after DMT group removal without purification (UV absorbance at 260 nm vs time in min).

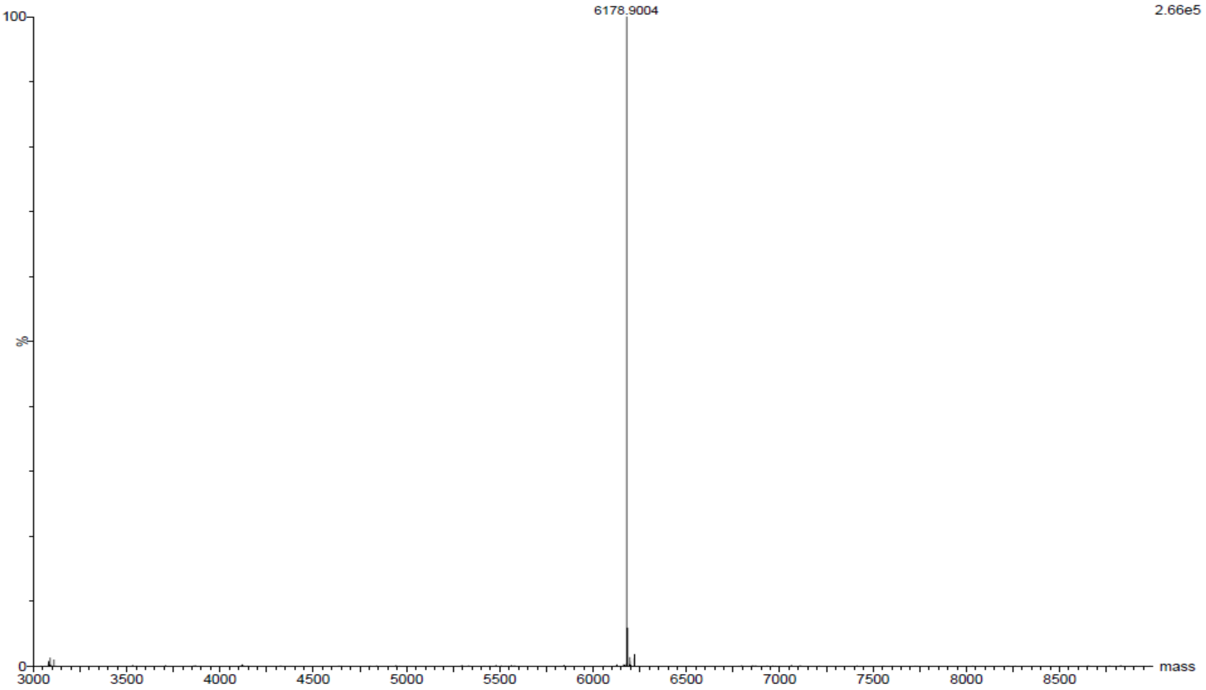

Supplementary Figure S39: Mass spectrum (ES-) of **ON11** after DMT group removal without purification. Required **6179.01** Da, found **6178.90** Da. y-axis = relative intensity (%), x-axis = mass in Da.

ON12 iPr	CCU CUU ACC UCA GUU ACA	**3.^{i}Pr**	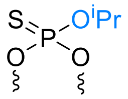

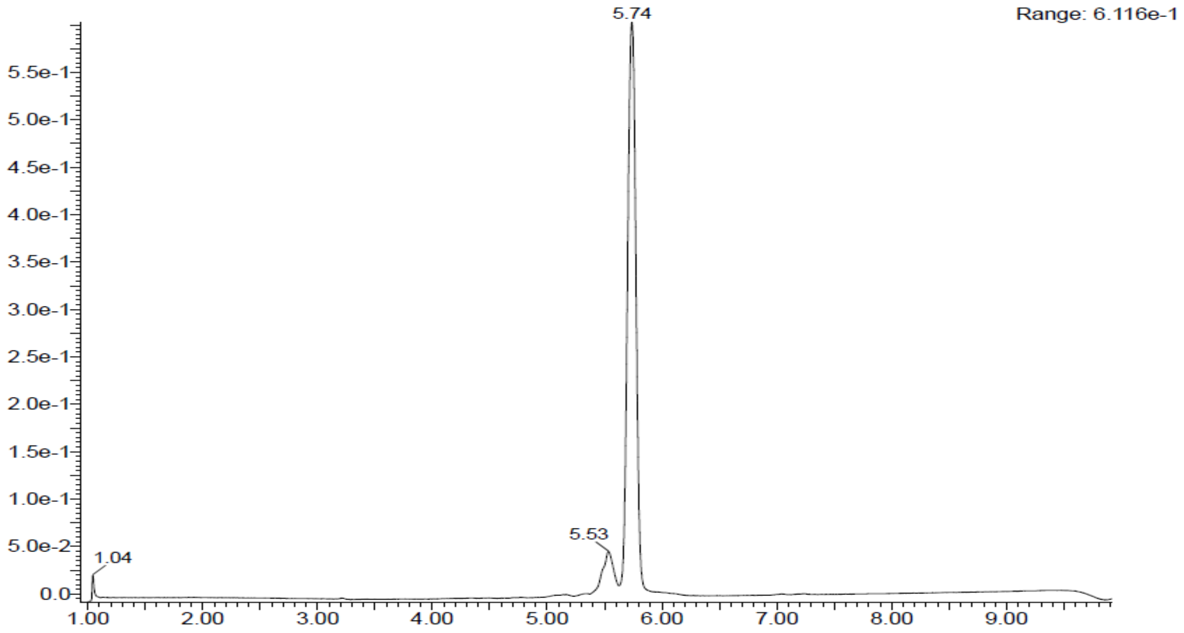

Supplementary Figure S40: Reverse-phase UPLC of crude **ON12** after DMT group removal without purification (UV absorbance at 260 nm vs time in min).

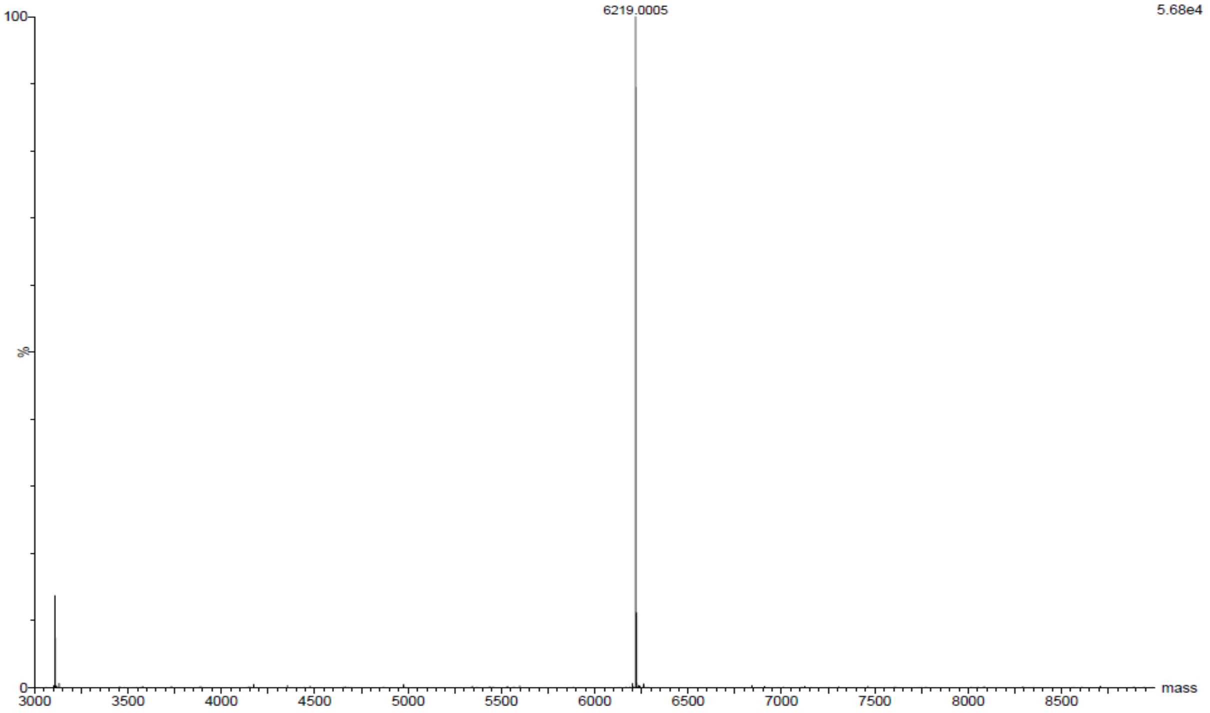

Supplementary Figure S41: Mass spectrum (ES-) of **ON12** after DMT group removal without purification. Required **6219.07** Da, found **6219.00** Da. y-axis = relative intensity (%), x-axis = mass in Da.

ON13 iPr	CCU CUU ACC UCA GUT ACA	**1 ^{i}Pr**	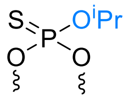

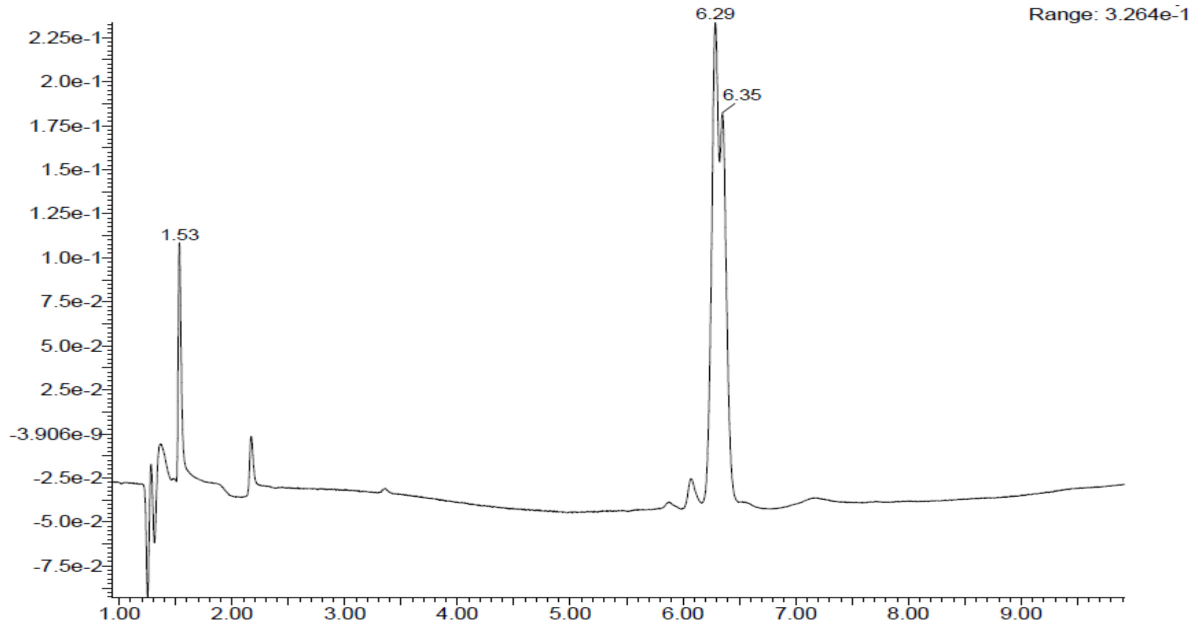

Supplementary Figure S42: Reverse-phase UPLC of crude **ON13** after DMT group removal without purification (UV absorbance at 260 nm vs time in min).

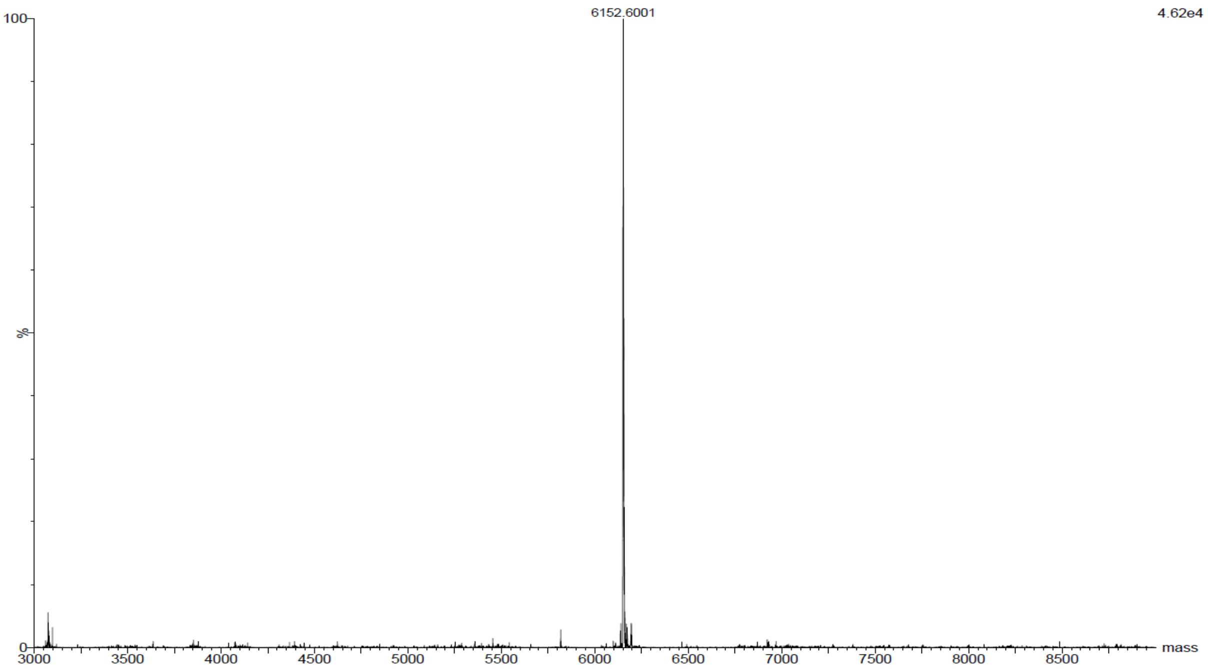

Supplementary Figure S43: Mass spectrum (ES-) of **ON13** after DMT group removal without purification. Required **6152.97** Da, found **6152.60** Da. y-axis = relative intensity (%), x-axis = mass in Da.

ON14 iPr	CCU CUT ACC UCA GUT ACA	**2.^{i}Pr**	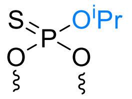

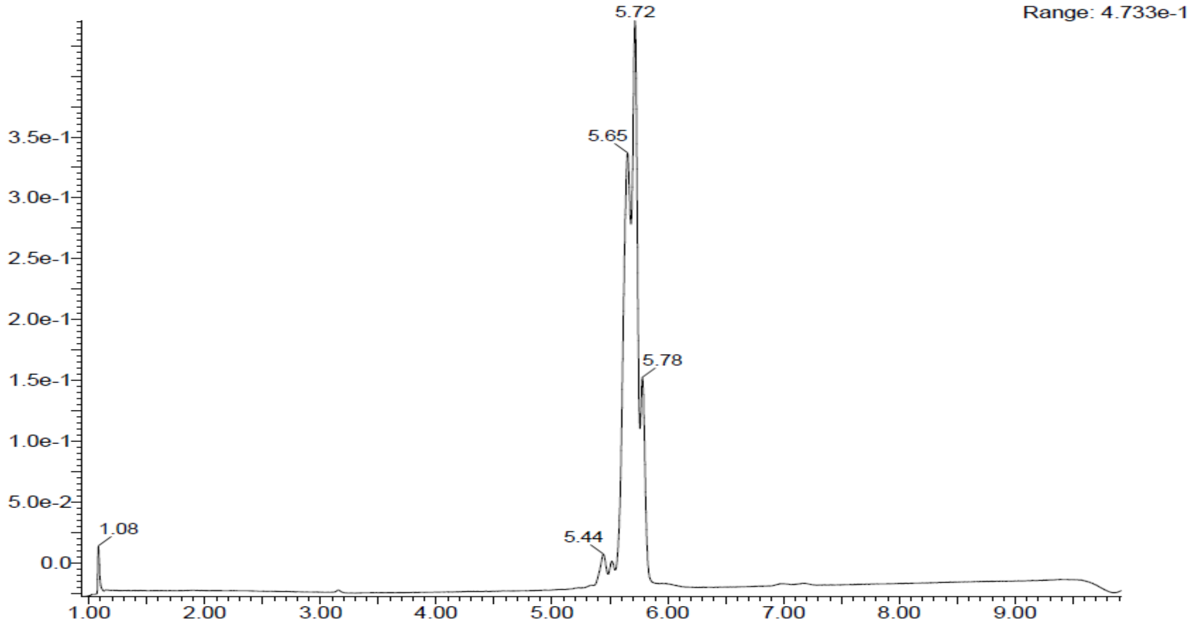

Supplementary Figure S44: Reverse-phase UPLC of crude **ON14** after DMT group removal without purification (UV absorbance at 260 nm vs time in min).

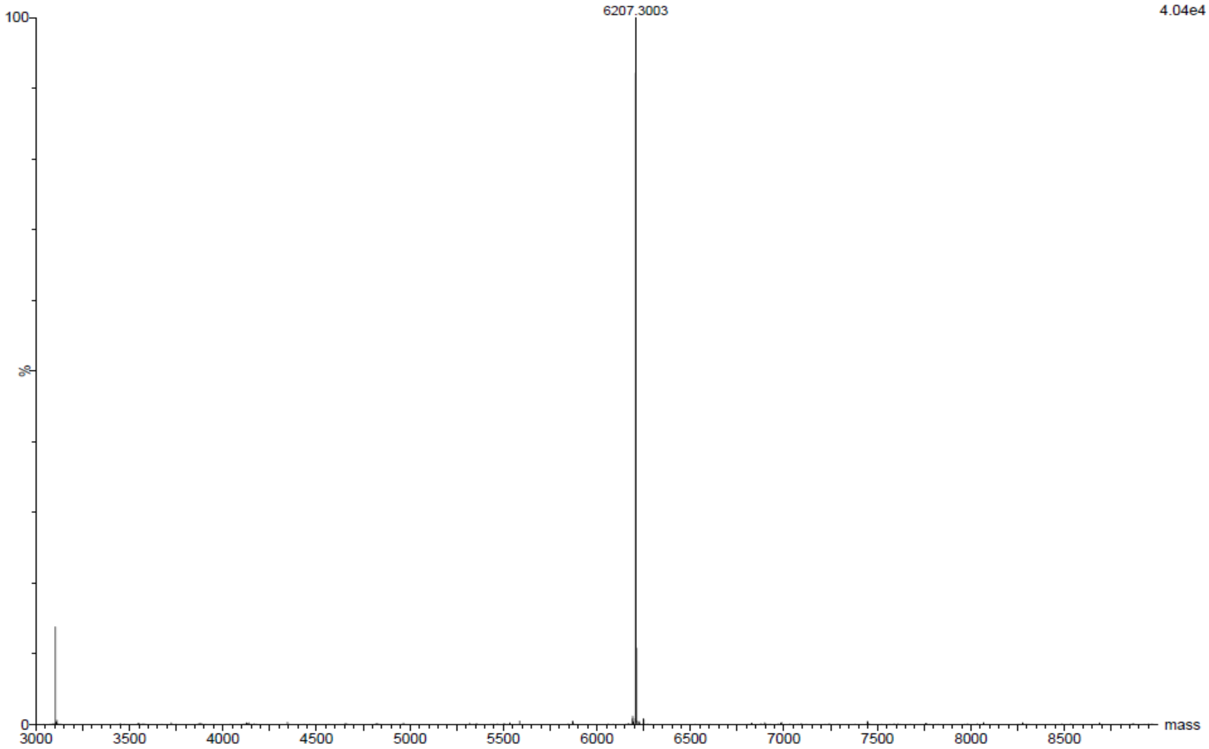

Supplementary Figure S45: Mass spectrum (ES-) of crude **ON14** after DMT group removal without purification. Required **6207.06** Da, found **6207.30** Da. y-axis = relative intensity (%), x-axis = mass in Da.

ON15 iPr	CCT CUT ACC TCA GUT ACA	**4.^{i}Pr**	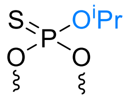

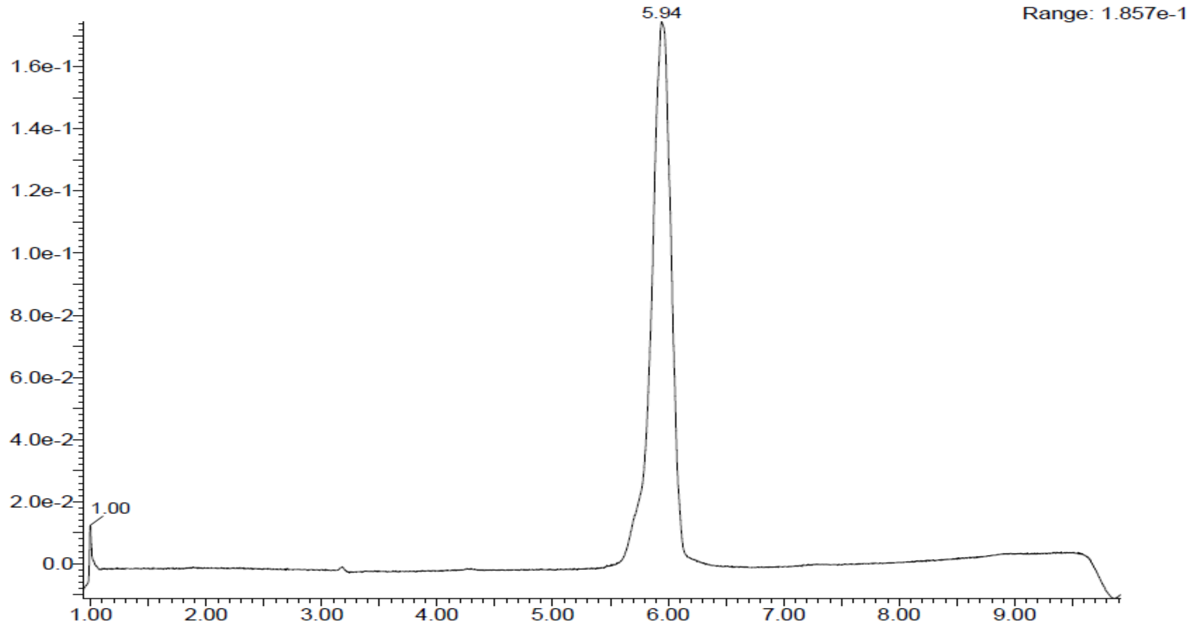

Supplementary Figure S46: Reverse-phase UPLC of crude **ON15** after DMT group removal without purification (UV absorbance at 260 nm vs time in min).

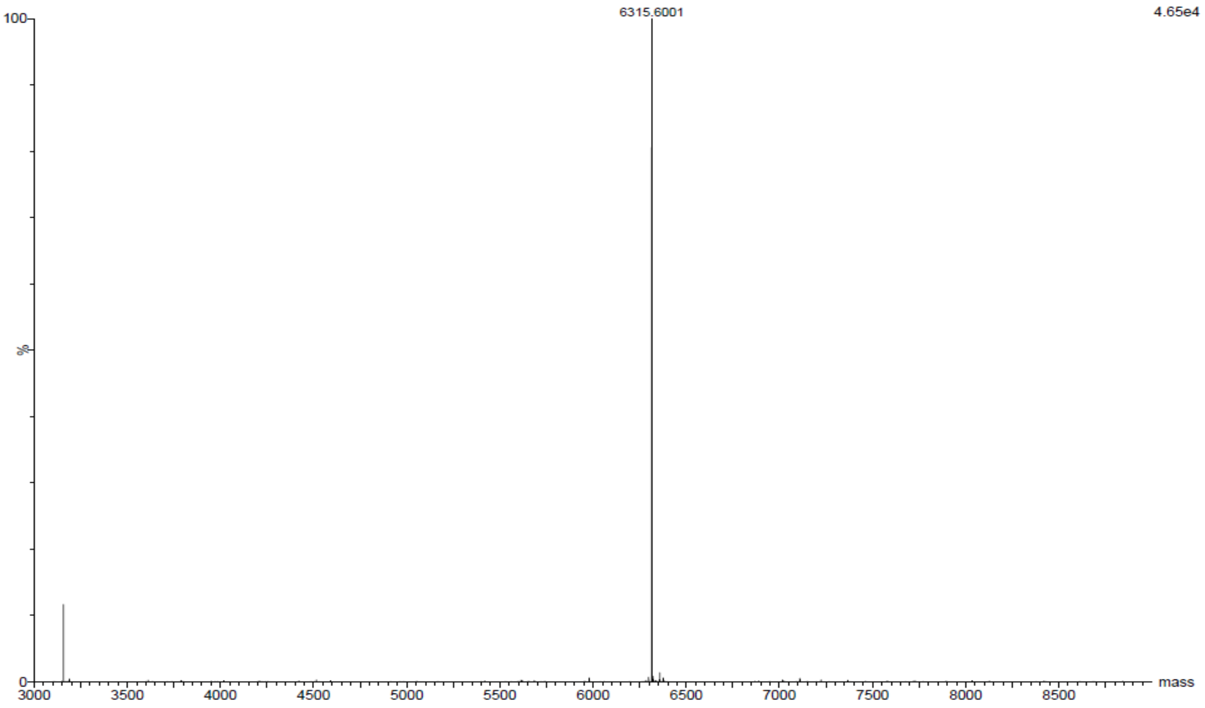

Supplementary Figure S47: Mass spectrum (ES-) of crude **ON15** after DMT group removal without purification. Required **6315.25** Da, found **6315.60** Da. y-axis = relative intensity (%), x-axis = mass in Da.

ON16 iPr	CCT CTT ACC TCA GTT ACA	**6.^{i}Pr**	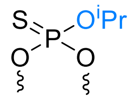

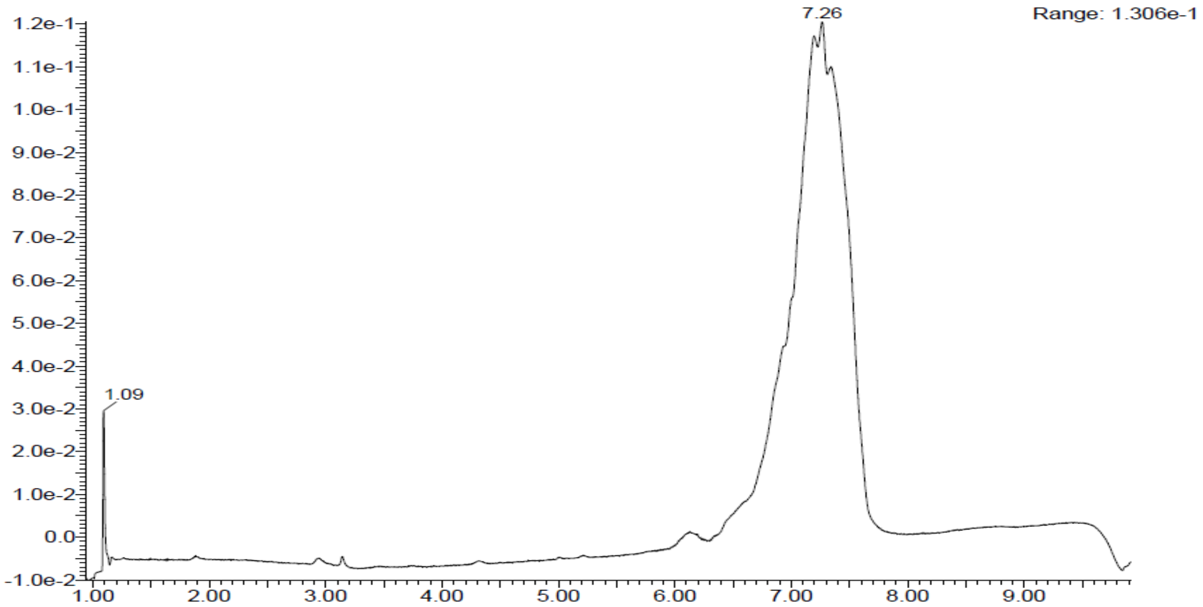

Supplementary Figure S48: Reverse-phase UPLC of crude **ON16** after DMT group removal without purification (UV absorbance at 260 nm vs time in min).

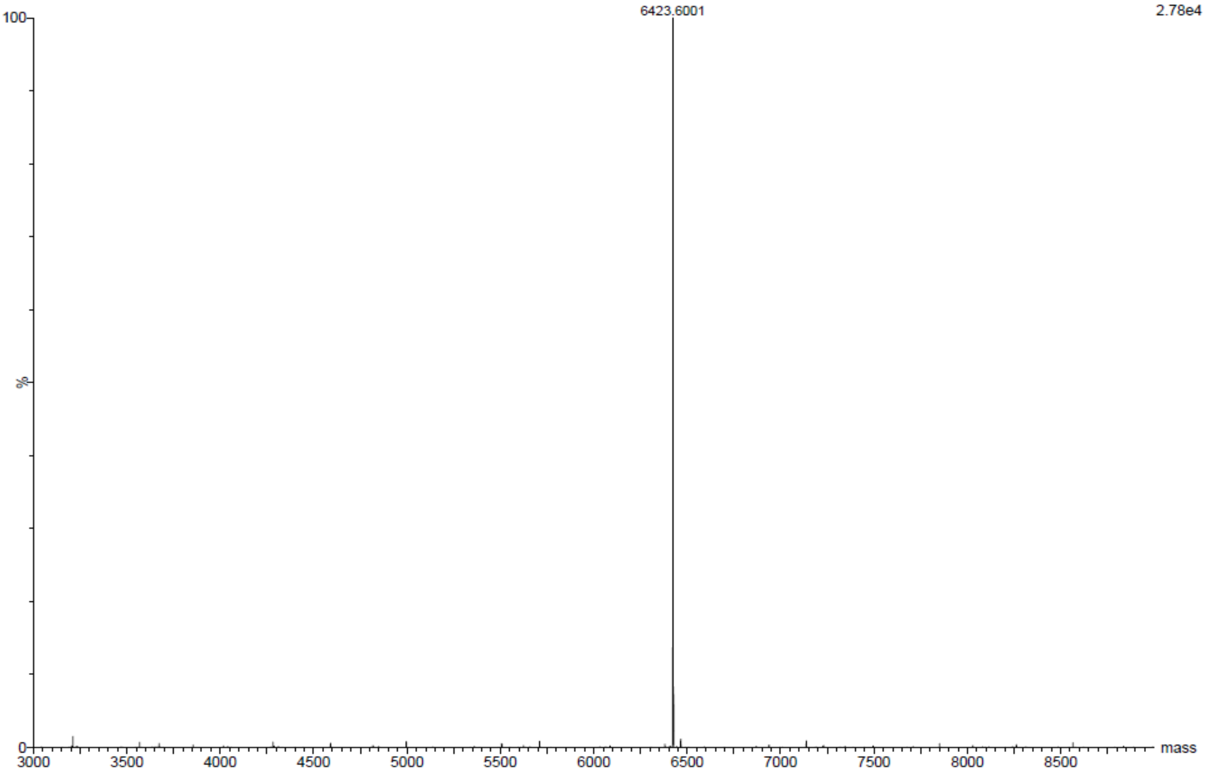

Supplementary Figure S49: Mass spectrum (ES-) of crude **ON16** after DMT group removal without purification. Required **6423.43** Da, found **6423.60** Da. y-axis = relative intensity (%), x-axis = mass in Da.

2.3 LNA phosphorothiaote oligonucleotides

ON17 LNA control	CCU CUU ACC UCA GUU ACA	**phosphorothiaote**	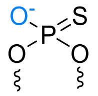

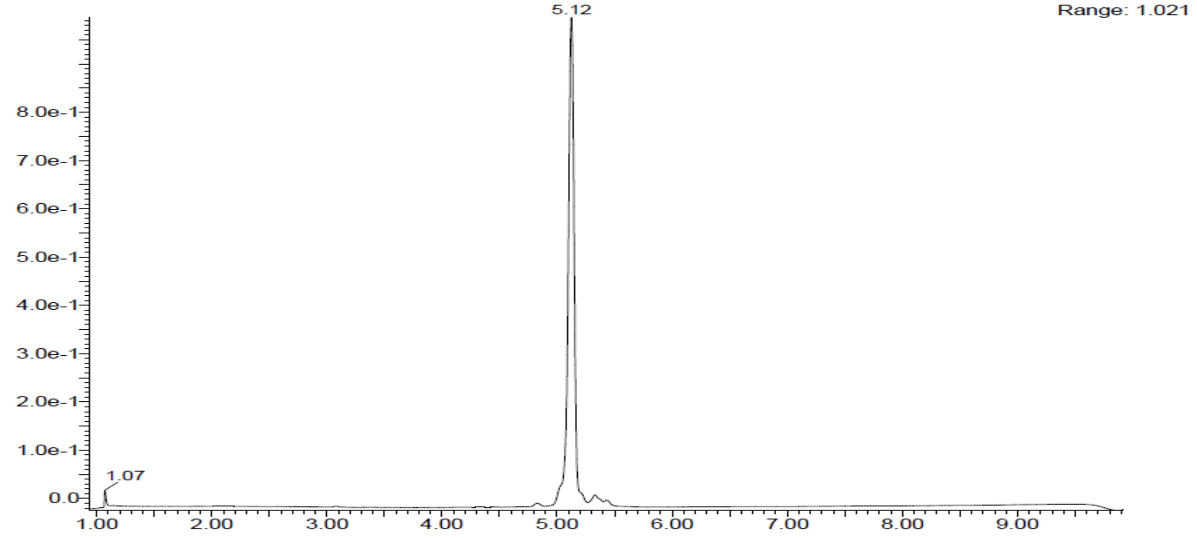

Supplementary Figure S50: Reverse-phase UPLC of crude **ON17** after DMT group removal without purification (UV absorbance at 260 nm vs time in min).

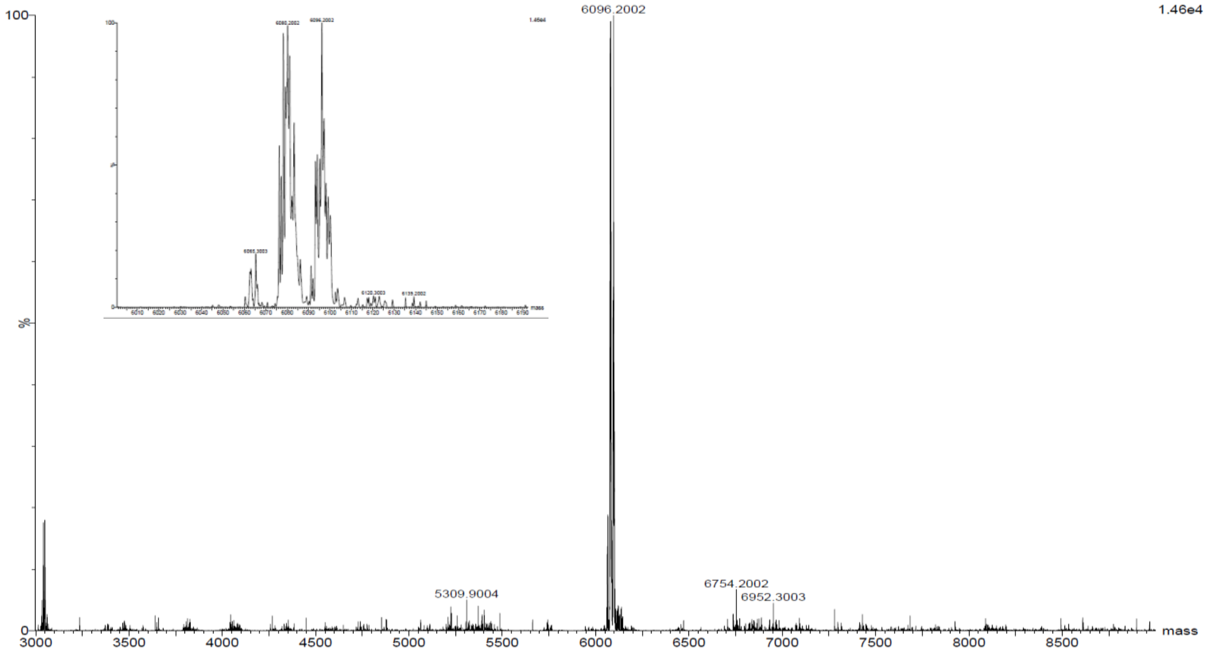

Supplementary Figure S51. Mass spectrum (ES-) of crude **ON17** after DMT group removal without purification. Required **6096.86** Da, found **6096.20** Da. y-axis = relative intensity (%), x-axis = mass in Da.

ON18 LNA control	CCU CUU ACC UCA GUU ACA	**phosphorothiaote**	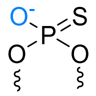

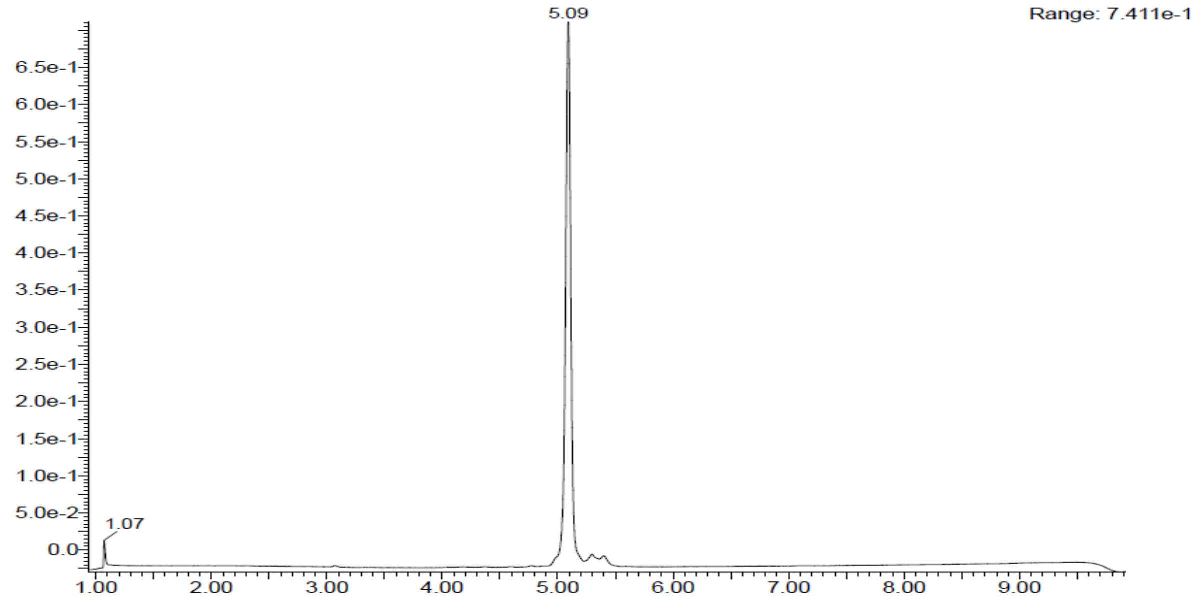

Supplementary Figure S52: Reverse-phase UPLC of crude **ON18** after DMT group removal without purification (UV absorbance at 260 nm vs time in min).

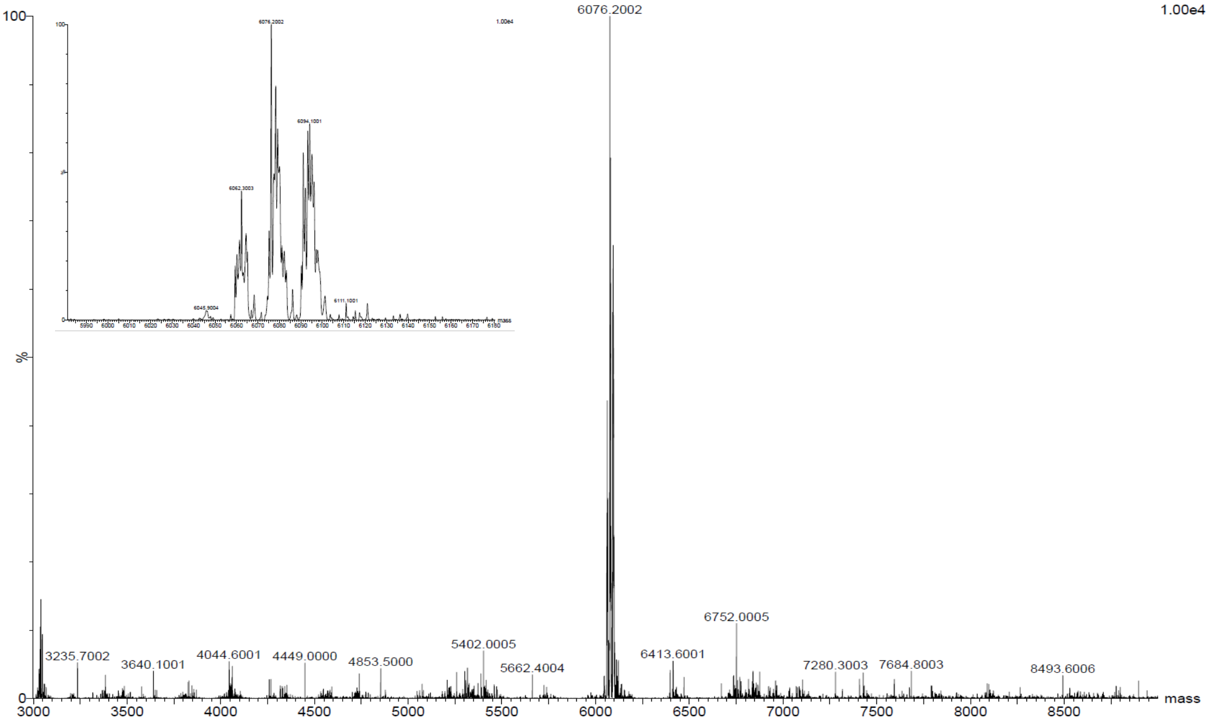

Supplementary Figure S53: Mass spectrum (ES-) of crude **ON18** after DMT removal without purification. Required **6094.85** Da, found **6094.10** Da. y-axis = relative intensity (%), x-axis = mass in Da.

ON19 LNA control	CCU CUU ACC UCA GUU ACA	**phosphorothiaote**	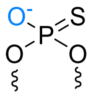

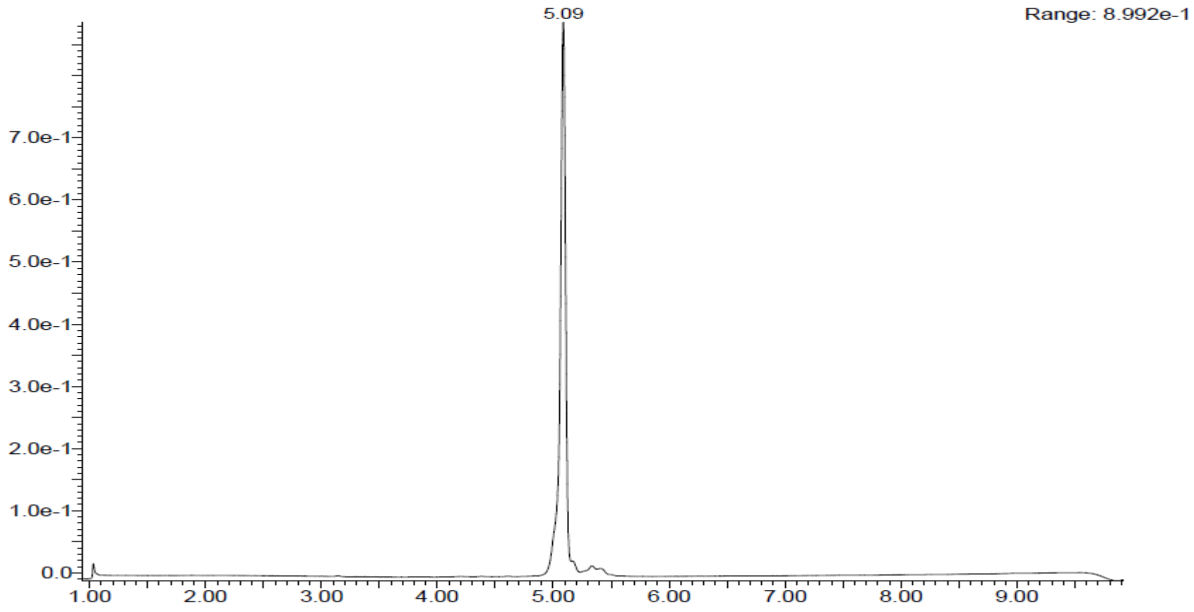

Supplementary Figure S54: Reverse-phase UPLC of crude **ON19** after DMT group removal without purification (UV absorbance at 260 nm vs time in min).

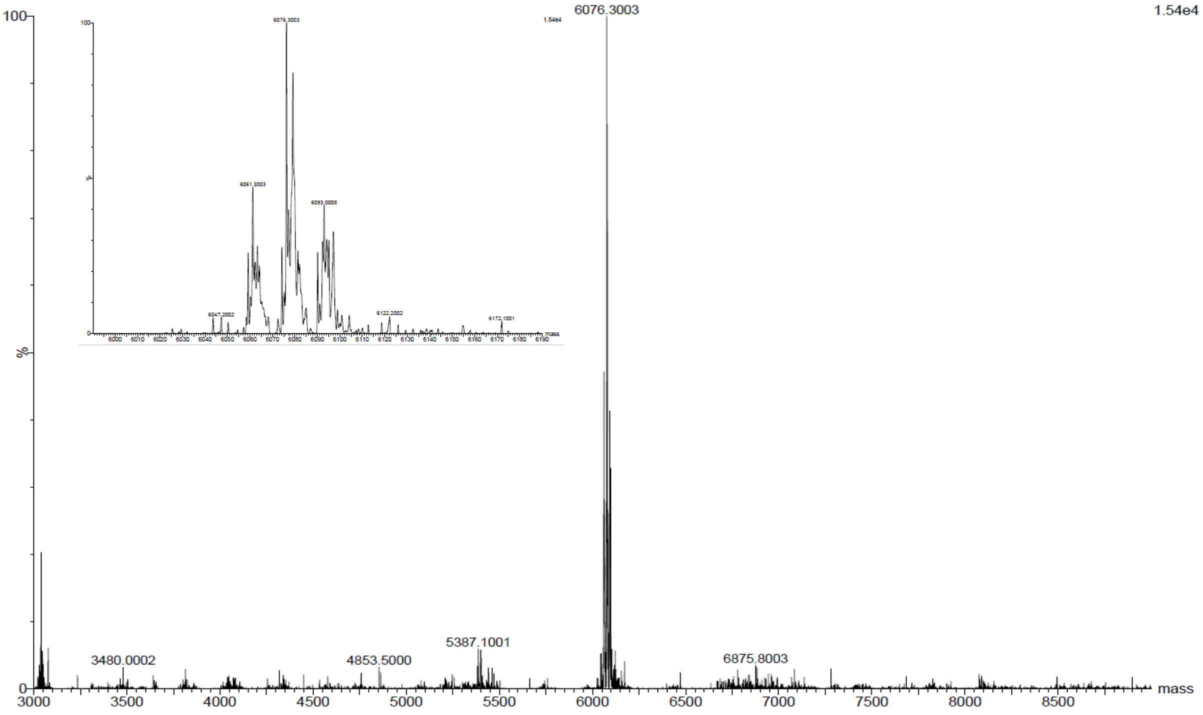

Supplementary Figure S55: Mass spectrum (ES-) of crude **ON19** after DMT group removal without purification. Required **6094.85** Da, found **6093.00** Da. y-axis = relative intensity (%), x-axis = mass in Da.

ON20 LNA control	CCU CUU ACC UCA GUU ACA	**phosphorothiaote**	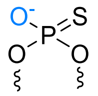

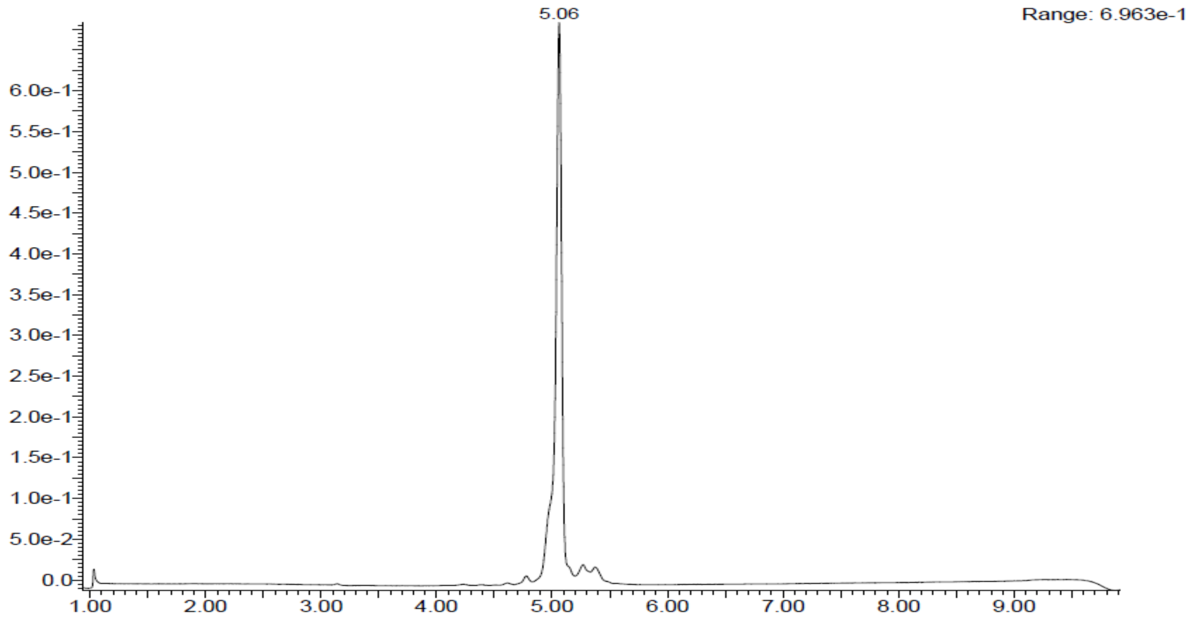

Supplementary Figure S56: Reverse-phase UPLC of crude **ON20** after DMT group removal without purification (UV absorbance at 260 nm vs time in min).

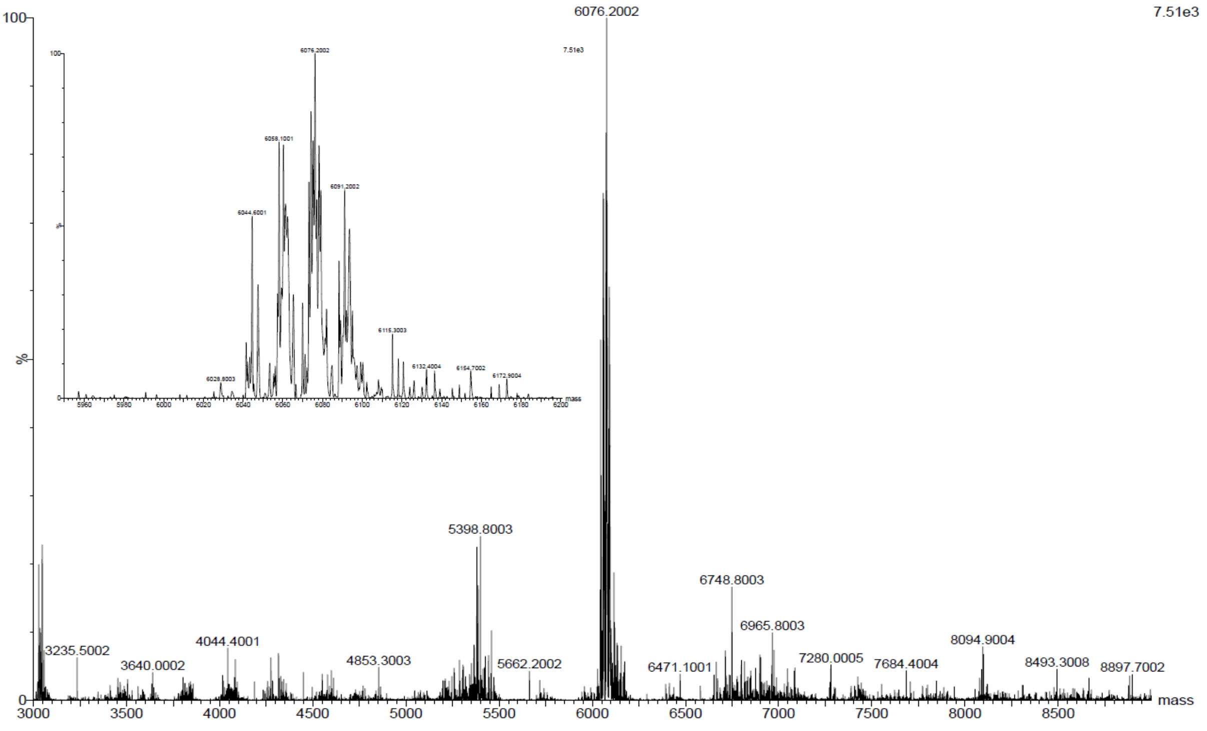

Supplementary Figure S57: Mass spectrum (ES-) of crude **ON20** after DMT group removal without purification. Required **6092.83** Da, found **6091.20** Da. y-axis = relative intensity (%), x-axis = mass in Da.

ON21 LNA control	CCU CUU ACC UCA GUT ACA	**phosphorothiaote**	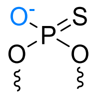

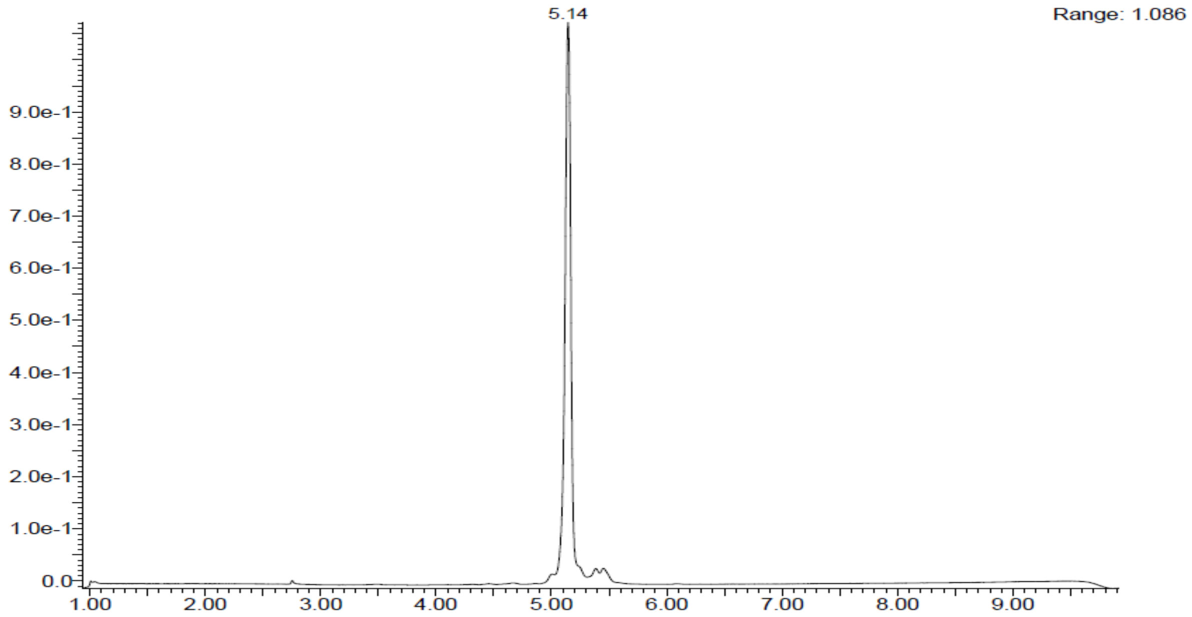

Supplementary Figure S58: Reverse-phase UPLC of crude **ON21** after DMT group removal without purification (UV absorbance at 260 nm vs time in min).

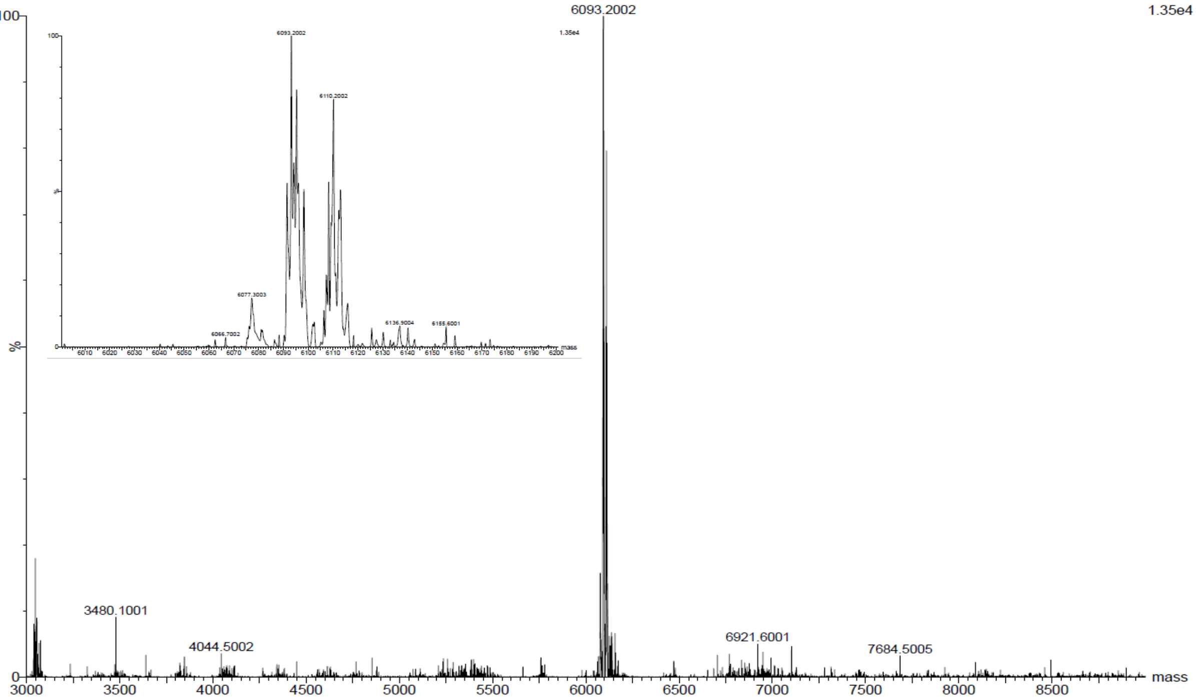

Supplementary Figure S59: Mass spectrum (ES-) of crude **ON21** after DMT removal without purification. Required **6110.89** Da, found **6110.20** Da. y-axis = relative intensity (%), x-axis = mass in Da.

ON22 LNA control	CCU CUT ACC UCA GUT ACA	**phosphorothiaote**	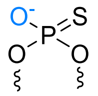

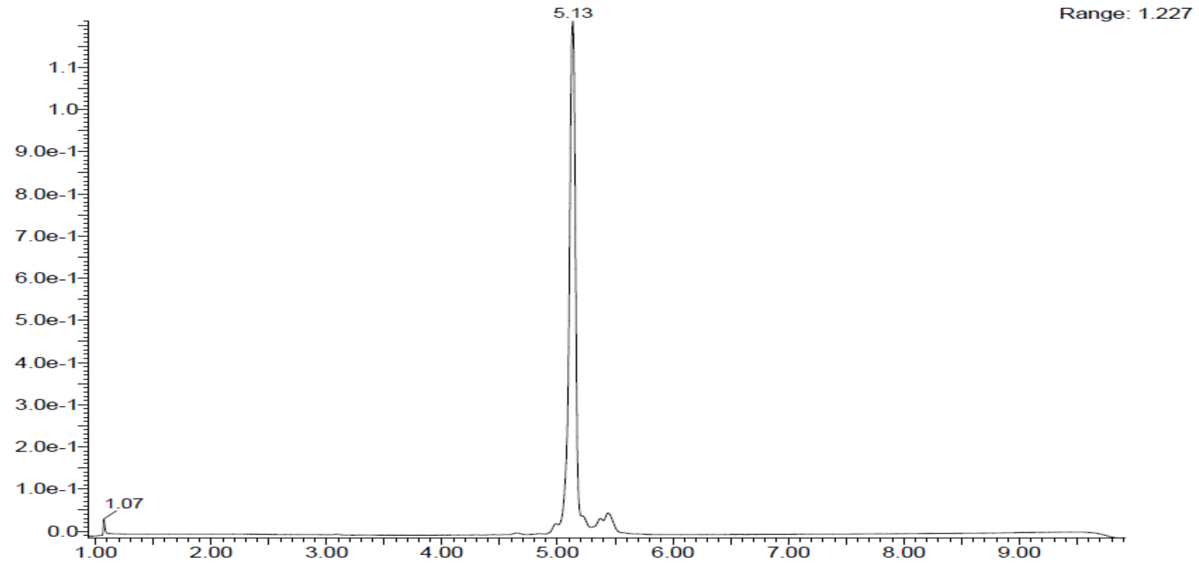

Supplementary Figure S60: Reverse-phase UPLC of crude **ON22** after DMT group removal without purification (UV absorbance at 260 nm vs time in min).

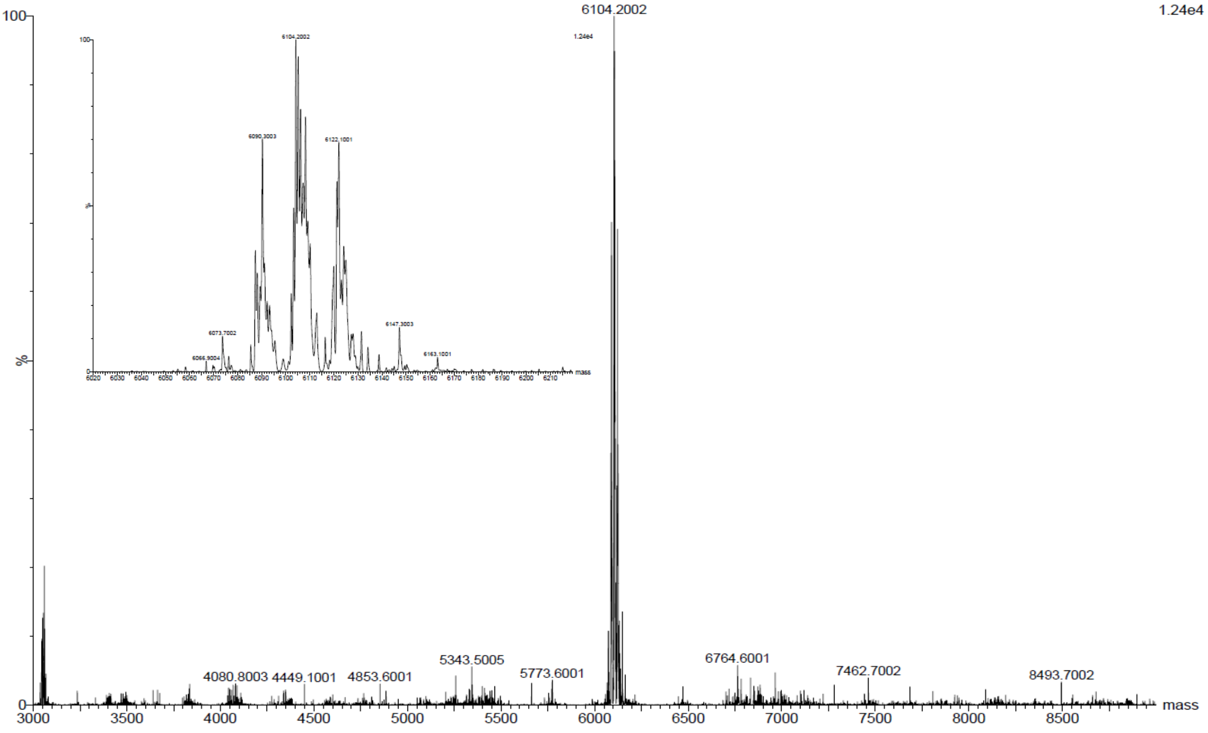

Supplementary Figure S61: Mass spectrum (ES-) of crude **ON22** after DMT group removal without purification. Required **6122.90** Da, found **6122.10** Da. y-axis = relative intensity (%), x-axis = mass in Da.

ON23 LNA control	CCT CUT ACC TCA GUT ACA	**phosphorothiaote**	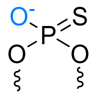

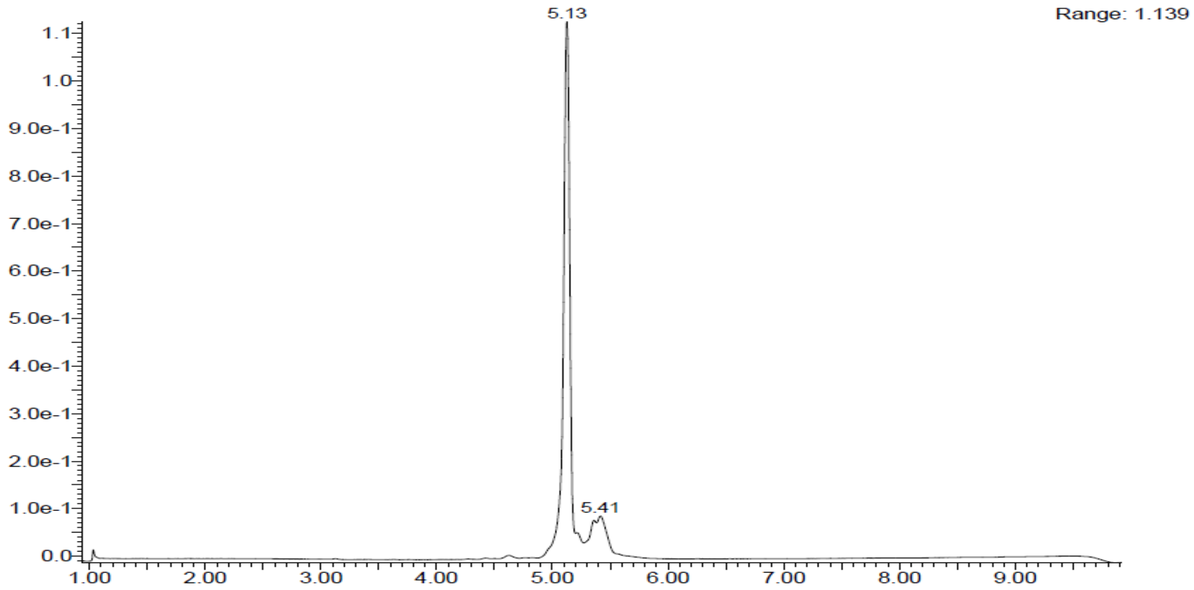

Supplementary Figure S62: Reverse-phase UPLC of crude **ON23** after DMT group removal without purification (UV absorbance at 260 nm vs time in min).

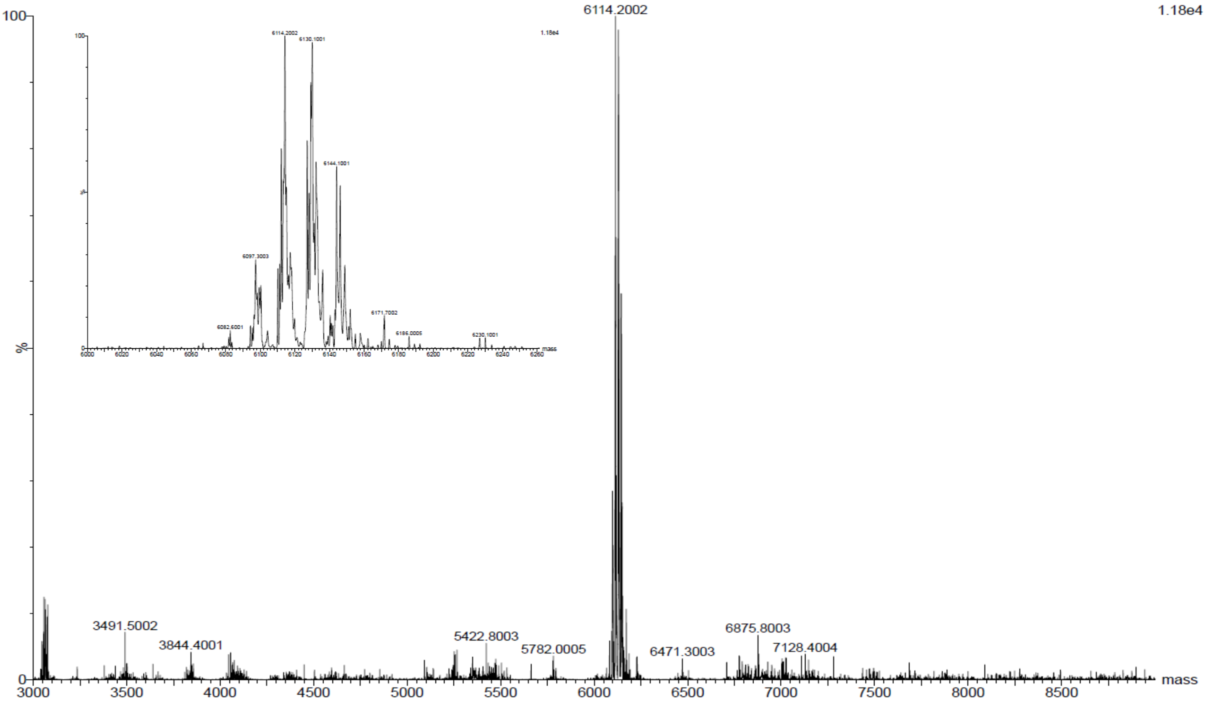

Supplementary Figure S63: Mass spectrum (ES-) of crude **ON23** after DMT group removal without purification. Required **6146.92** Da, found **6144.10** Da. y-axis = relative intensity (%), x-axis = mass in Da.

ON24 LNA control	CCT CTT ACC TCA GTT ACA	**phosphorothiaote**	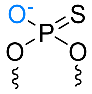

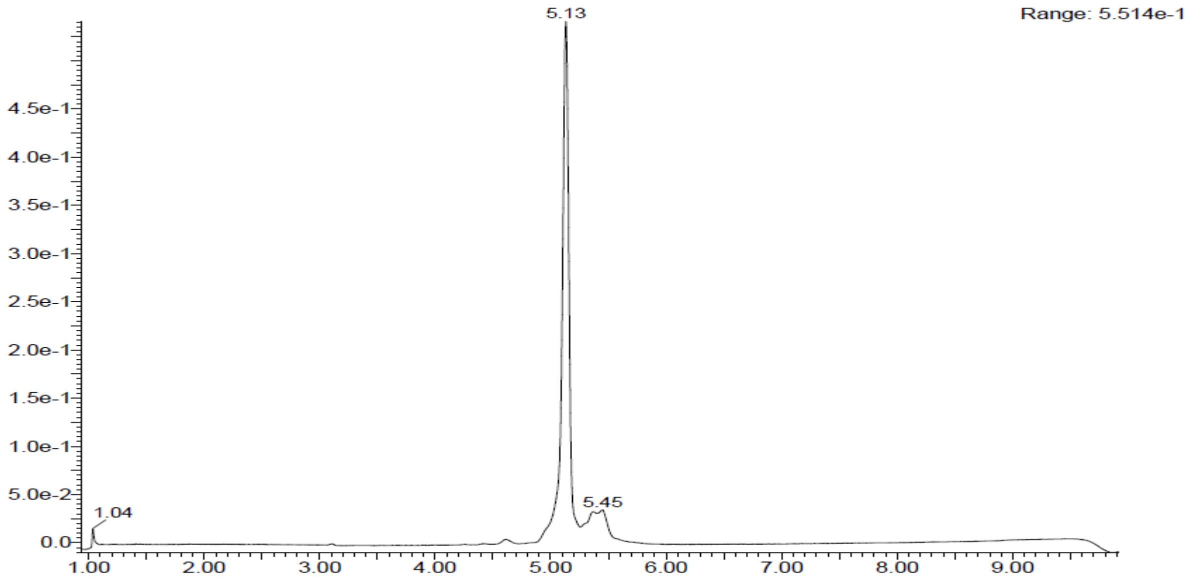

Supplementary Figure S64: Reverse-phase UPLC of crude **ON24** after DMT removal without purification (UV absorbance at 260 nm vs time in min).

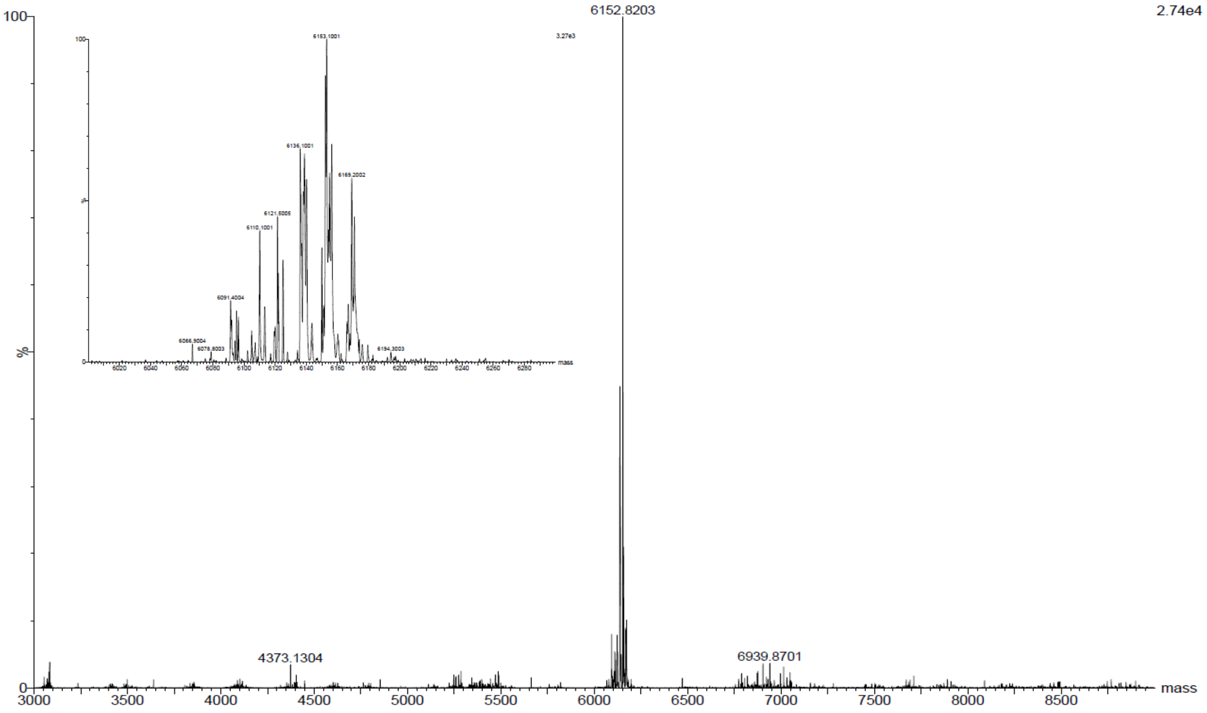

Supplementary Figure S65: Mass spectrum (ES-) of crude **ON24** after DMT removal without purification. Required **6170.94** Da, found **6169.20** Da. y-axis = relative intensity (%), x-axis = mass in Da.

2.3.1 Cleavage of tert-butyl group during oxidation step:

Oligonucleotide **ONS1-ONS4** were synthesise using the monomer **16** varying the oxidation and DMT removal steps after the coupling steps. An oligomer of ten thymines (T_{10}) was synthesized, in which **16** was incorporated as the last phosphoramidite (**A-T_{10}**). **ONS1** was synthesis by oxidising **A-T10** with iodine-water. **ONS2** was synthesised by removal of the DMT group of the **ONS1**. **ONS3** was synthesis from **A-T_{10}** by sulfurising with 3-Ethoxy-1,2,4-dithiazole-5-one (EDITH). **ONS4** was synthesised by removal of the DMT group from **ONS3**. In all cases cleavage of the tert-butyl group was observe by MS analysis.

Supplementary Table T4: Sequence of ONS1-ONS4 and observed mass

Oligonucleotide	Sequence (5′→3′)	Steps after coupling<br>ii. Oxidation/sulfurization<br>iii. detritylation	MS Expected (without ^{t}Bu group)	MS observed
ONS1	AT TTT TTT TTT	ii. I_2-oxidation	3623.52	3622.80
ONS2	AT TTT TTT TTT	ii. I_2-oxidation<br>iii. DMT removal	3321.15	3320.60
ONS3	AT TTT TTT TTT	ii. EDITH sulfurization	3639.50	3639.70
ONS4	AT TTT TTT TTT	ii. EDITH sulfurization<br>iii. DMT removal	3337.13	3338.50

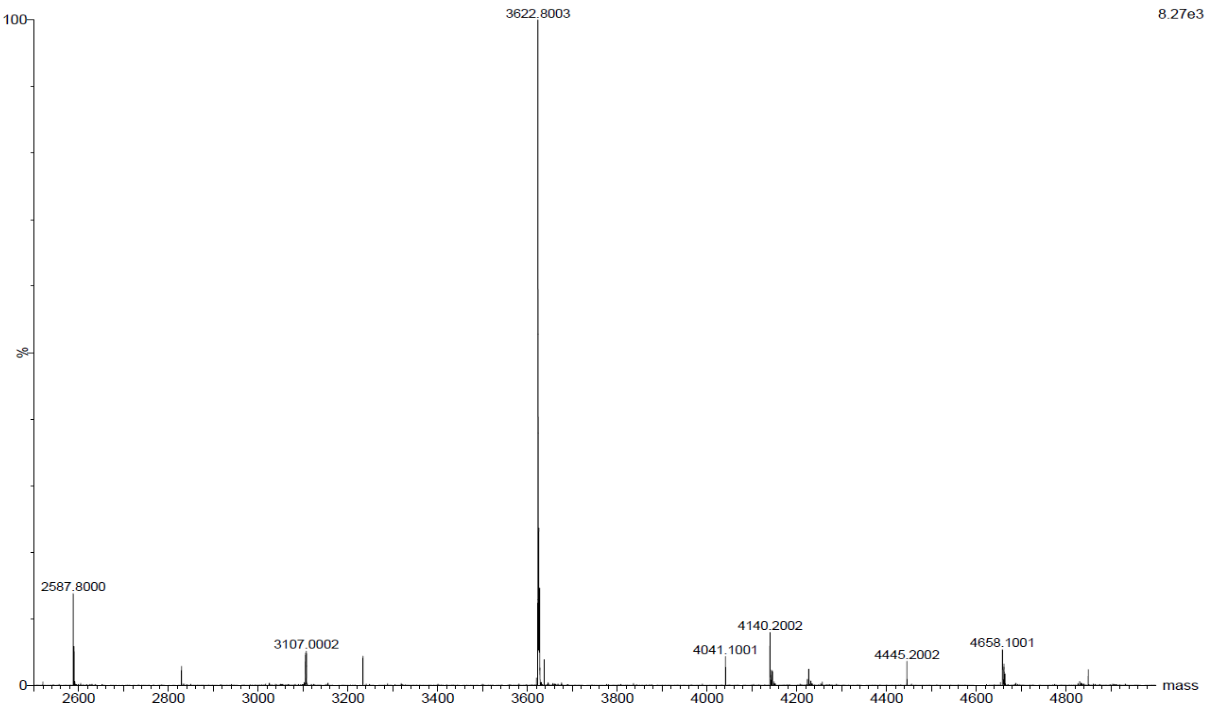

Supplementary Figure S66: Mass spectrum (ES-) of crude **ONS1** after cleavage from the solid support. Required **3623.52** Da, found **3622.80** Da. y-axis = relative intensity (%), x-axis = mass in Da.

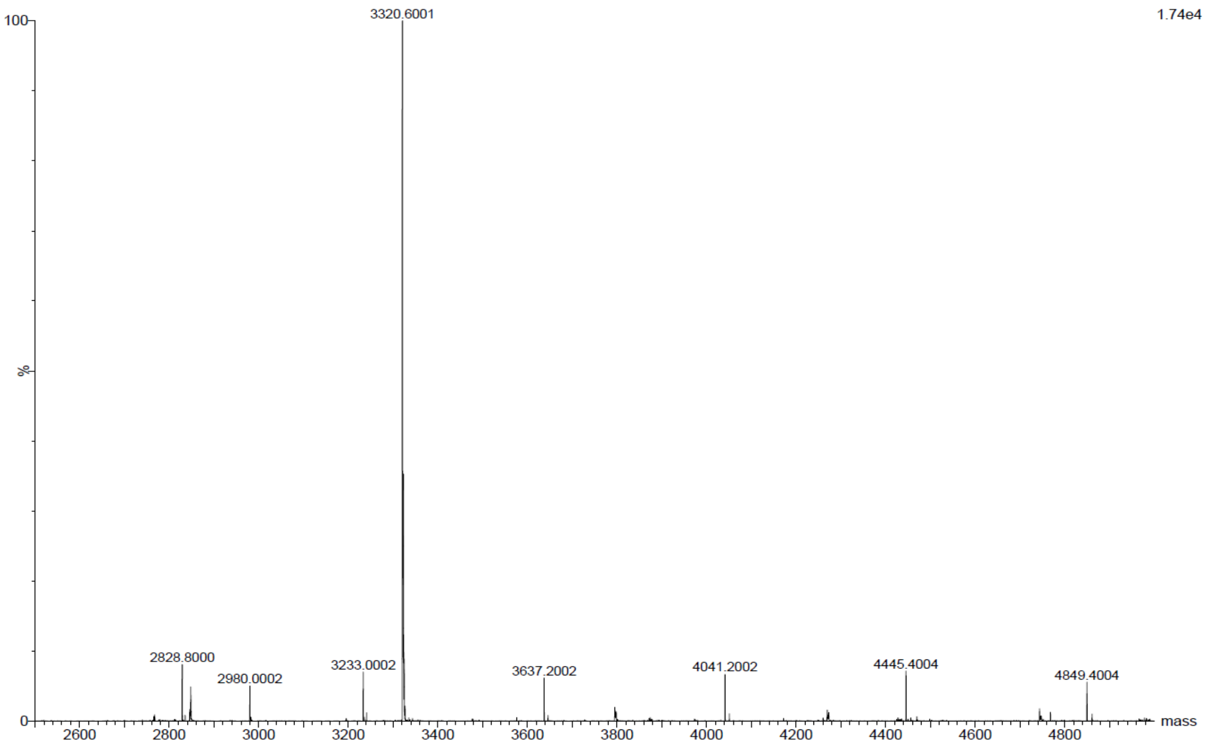

Supplementary Figure S67: Mass spectrum (ES-) of crude **ONS2** after cleavage from the solid support. Required **3321.15** Da, found **3320.60** Da. y-axis = relative intensity (%), x-axis = mass in Da.

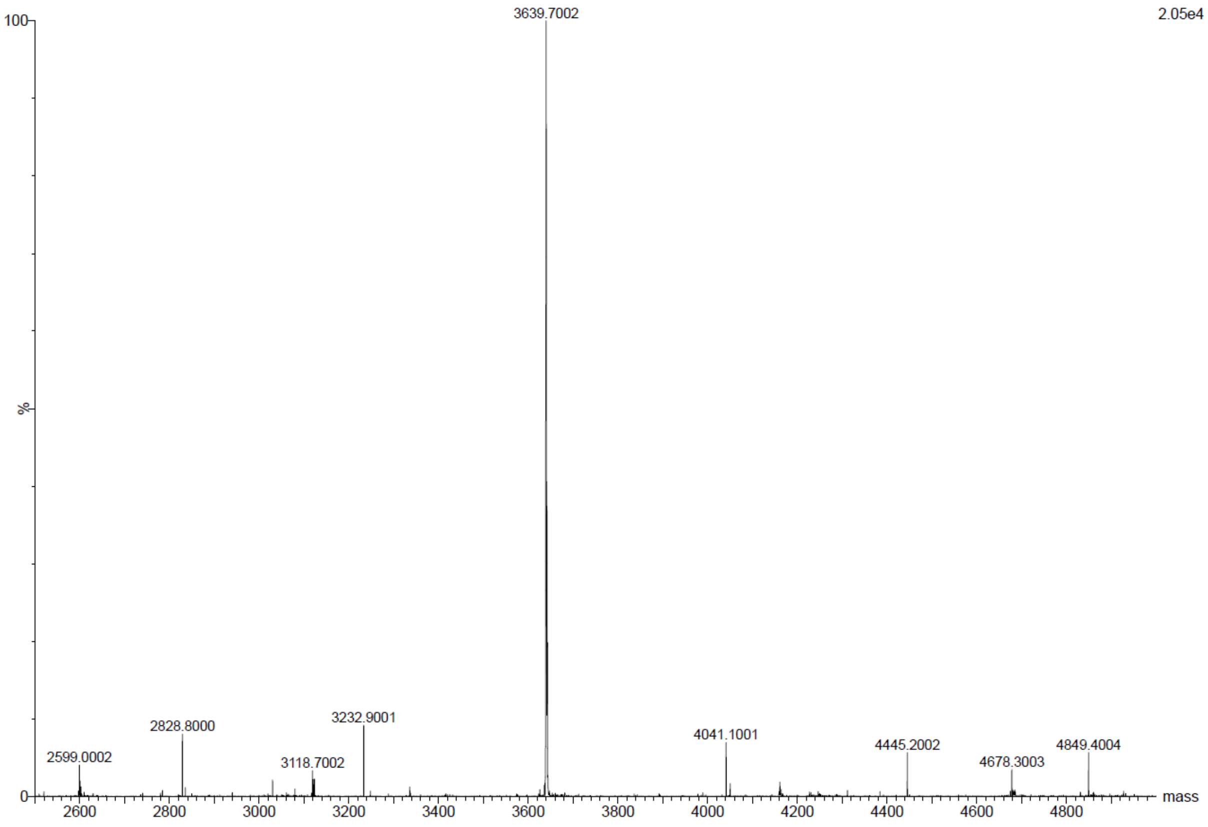

Supplementary Figure S68: Mass spectrum (ES-) of crude **ONS3** after cleavage from the solid support. Required **3639.50** Da, found **3639.70** Da. y-axis = relative intensity (%), x-axis = mass in Da.

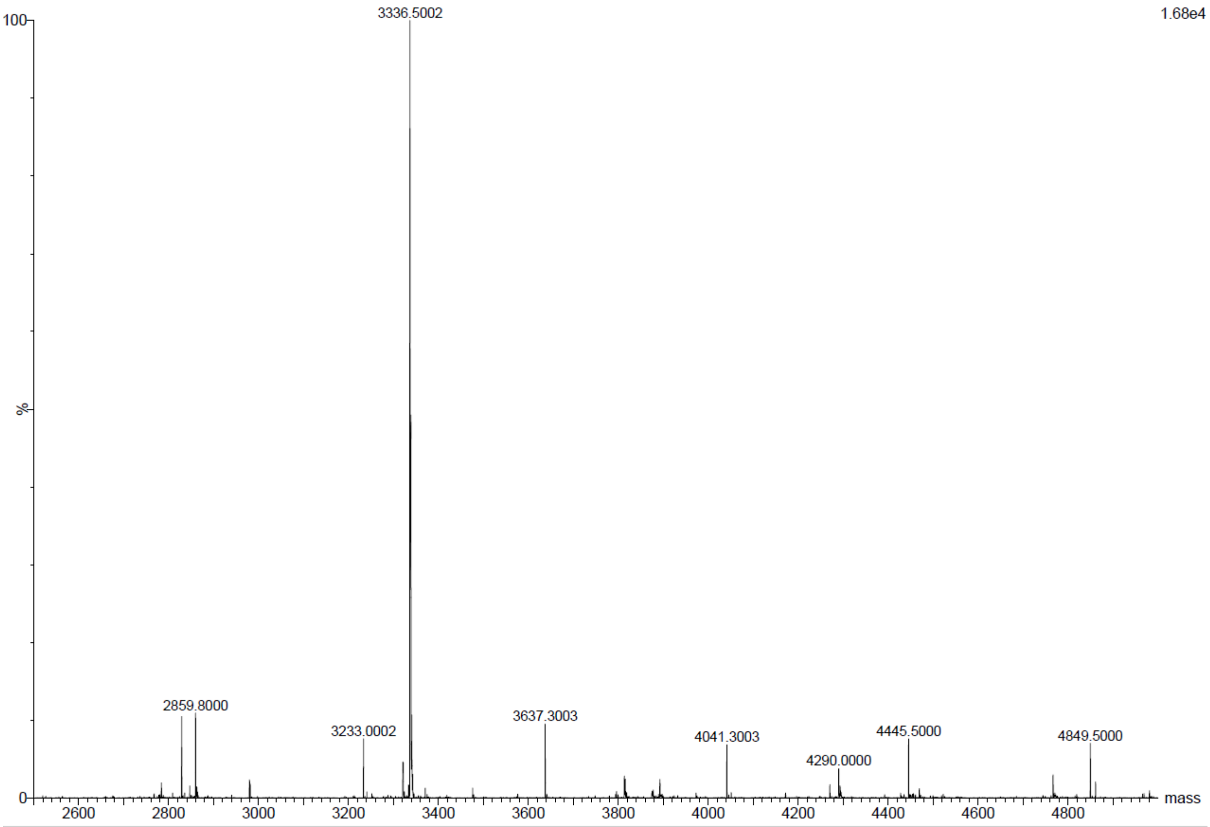

Supplementary Figure S69: Mass spectrum (ES-) of crude **ONS4** after cleavage from the solid support. Required **3337.13** Da, found **3336.50** Da. y-axis = relative intensity (%), x-axis = mass in Da.

2.4 THP PTTE oligonucleotides

ON25 THP	CCU CUU ACC UCA GUU ACA	**THP**	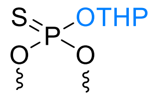

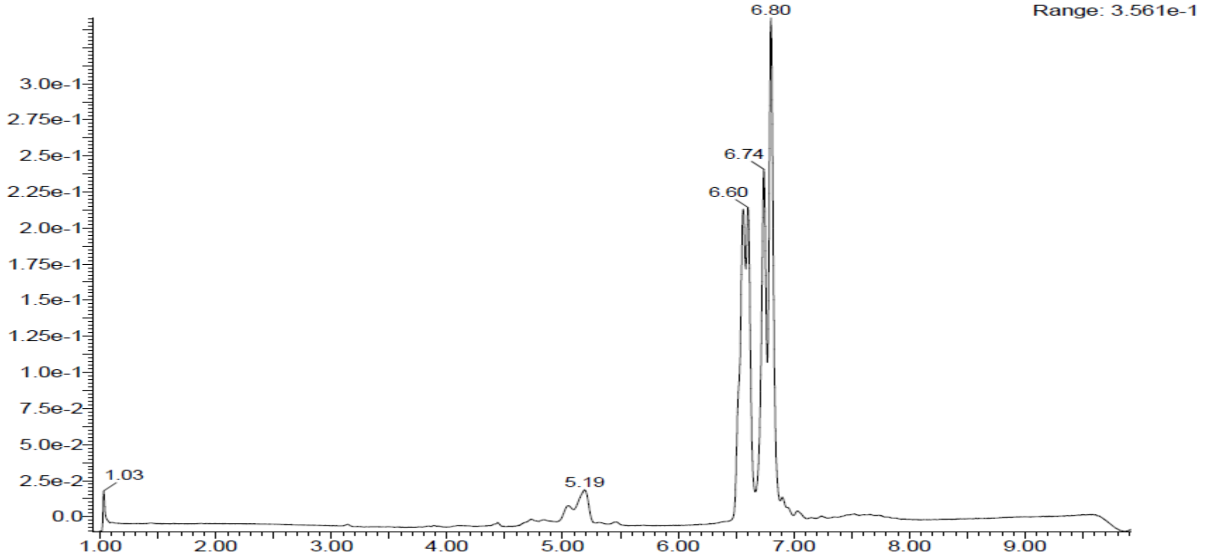

Supplementary Figure S70: Reverse-phase UPLC of **DMTON ON25** after deprotection from solid support (UV absorbance at 260 nm vs time in min)

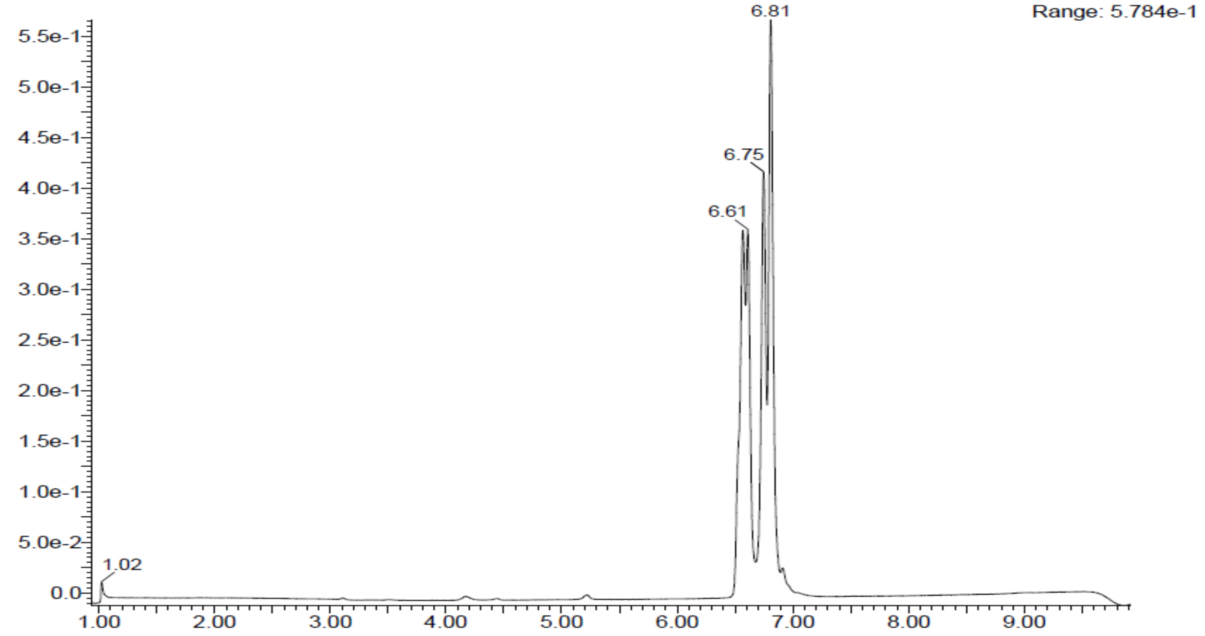

Supplementary Figure S71: Reverse-phase UPLC of **DMTON ON25** purification by HPLC (UV absorbance at 260 nm vs time in min).

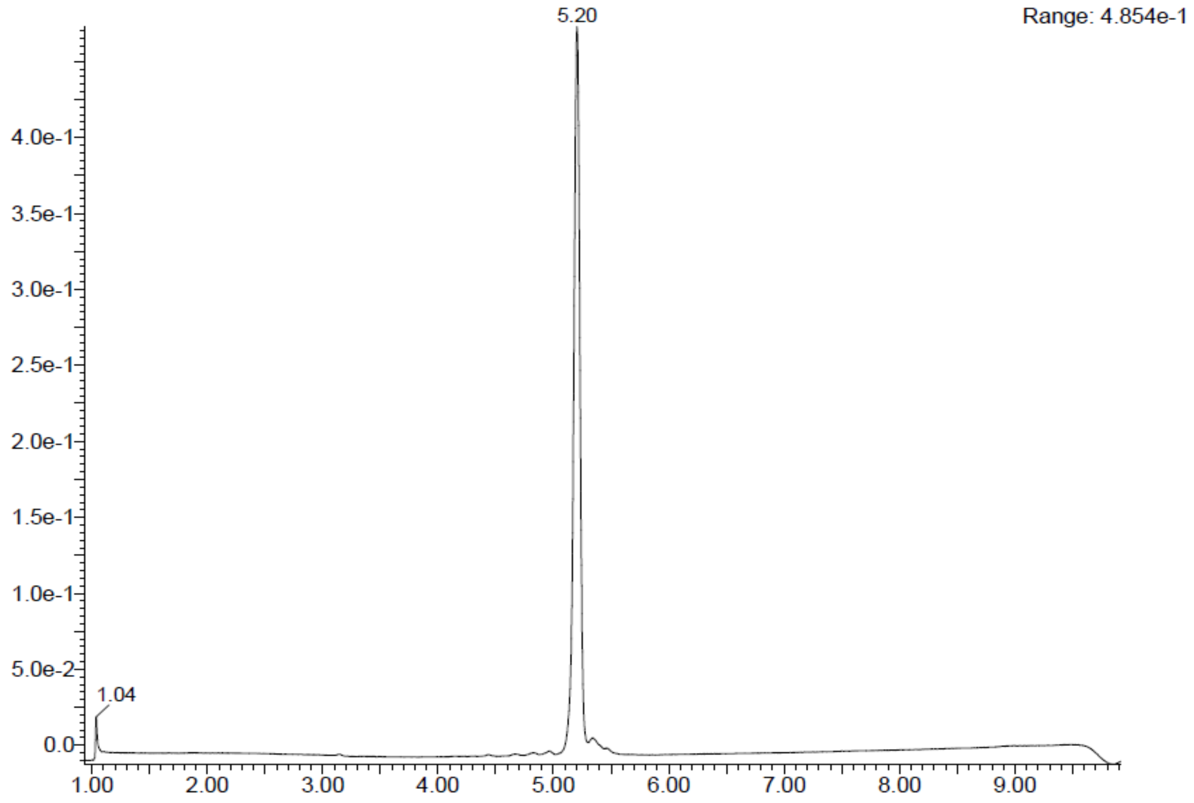

Supplementary Figure S72: Reverse-phase UPLC of crude **ON25** after DMT group removal without purification (UV absorbance at 260 nm vs time in min).

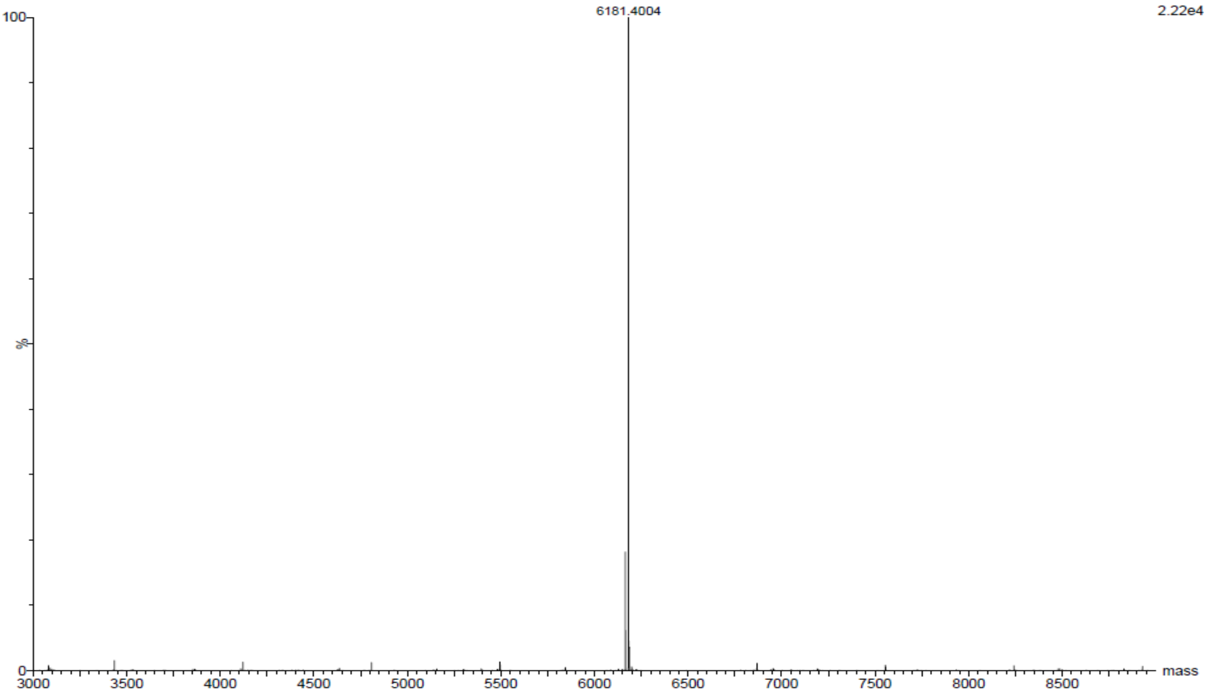

Supplementary Figure S73: Mass spectrum (ES-) of crude **ON25** after DMT group removal without purification. Required **6180.98** Da, found **6181.40** Da. y-axis = relative intensity (%), x-axis = mass in Da.

ON26 THP	CCU CUU ACC UCA GUU ACA	**2.THP**	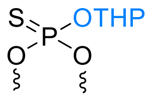

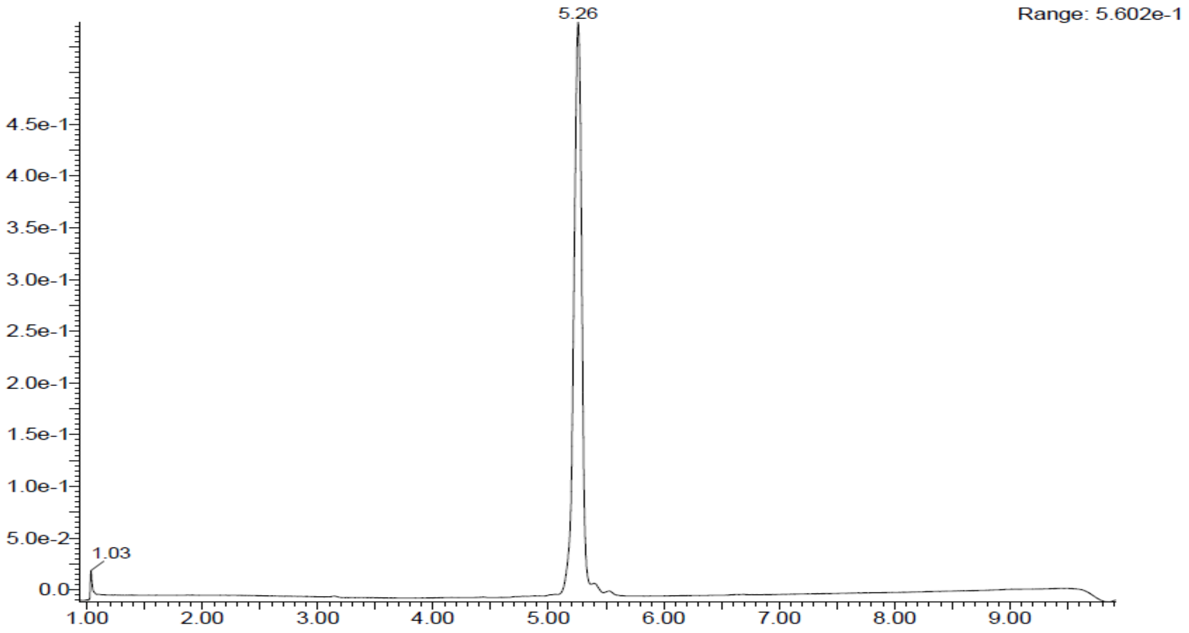

Supplementary Figure S74: Reverse-phase UPLC of crude **ON26** after DMT group removal without purification (UV absorbance at 260 nm vs time in min).

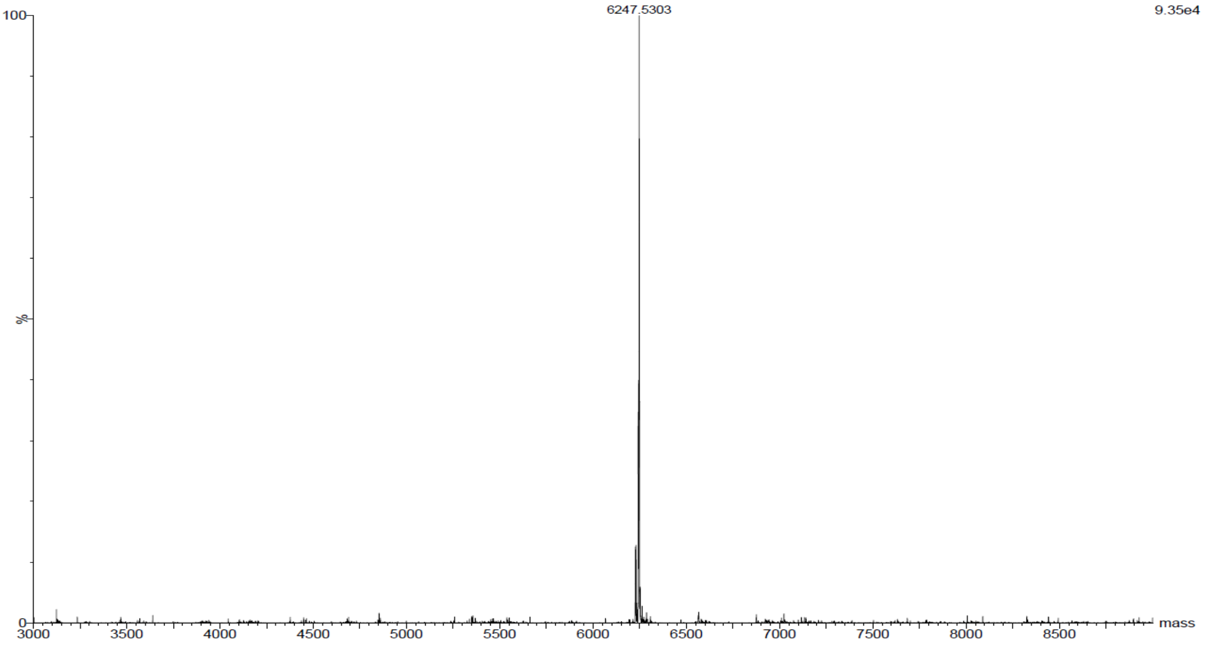

Supplementary Figure S75: Mass spectrum (ES-) of crude **ON26** after DMT group removal without purification. Required **6263.08** Da, found **6247.53** Da. y-axis = relative intensity (%), x-axis = mass in Da.

ON27 THP	CCU CUU ACC UCA GUU ACA	**2.THP**	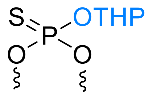

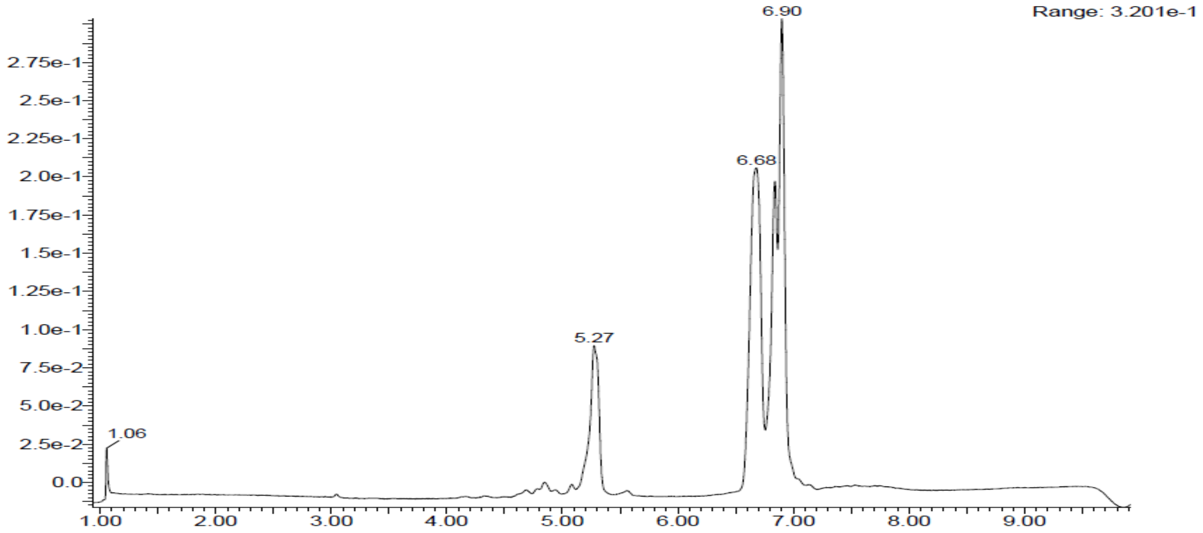

Supplementary Figure S76: Reverse-phase UPLC of **DMTON ON27** after deprotection from solid support (UV absorbance at 260 nm vs time in min).

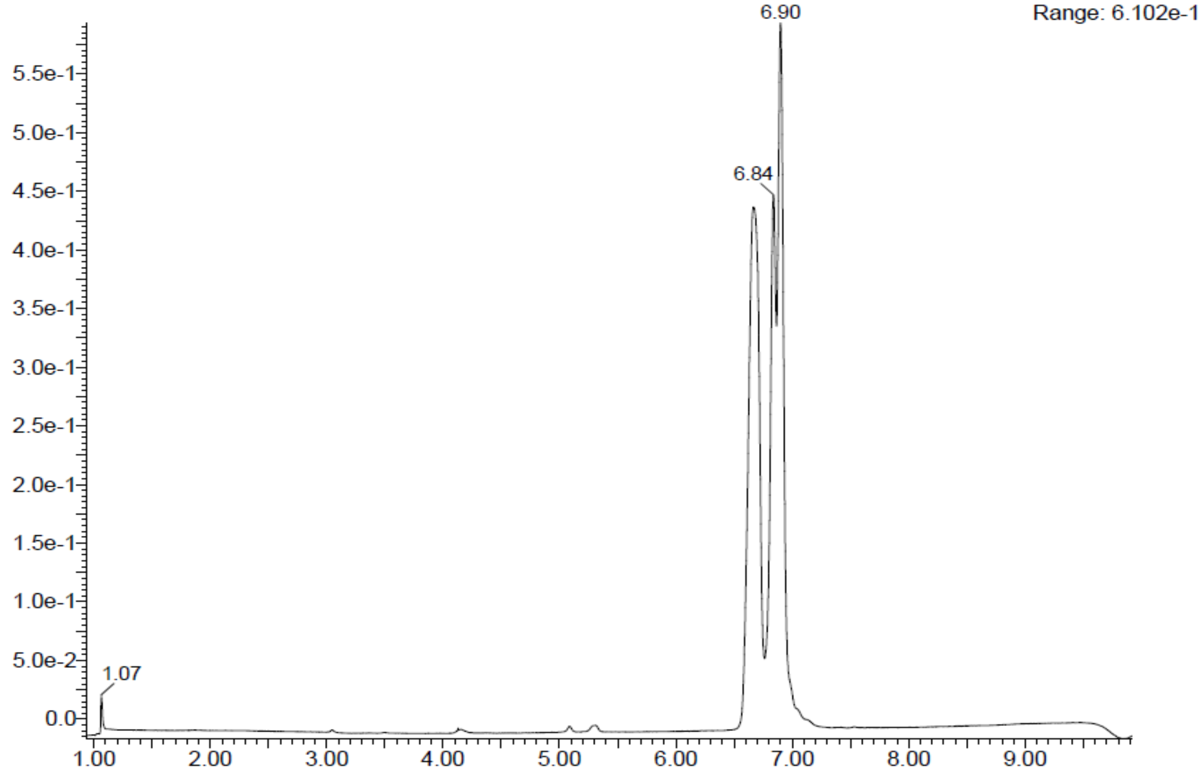

Supplementary Figure S77: Reverse-phase UPLC of **DMTON ON27** purification by HPLC (UV absorbance at 260 nm vs time in min).

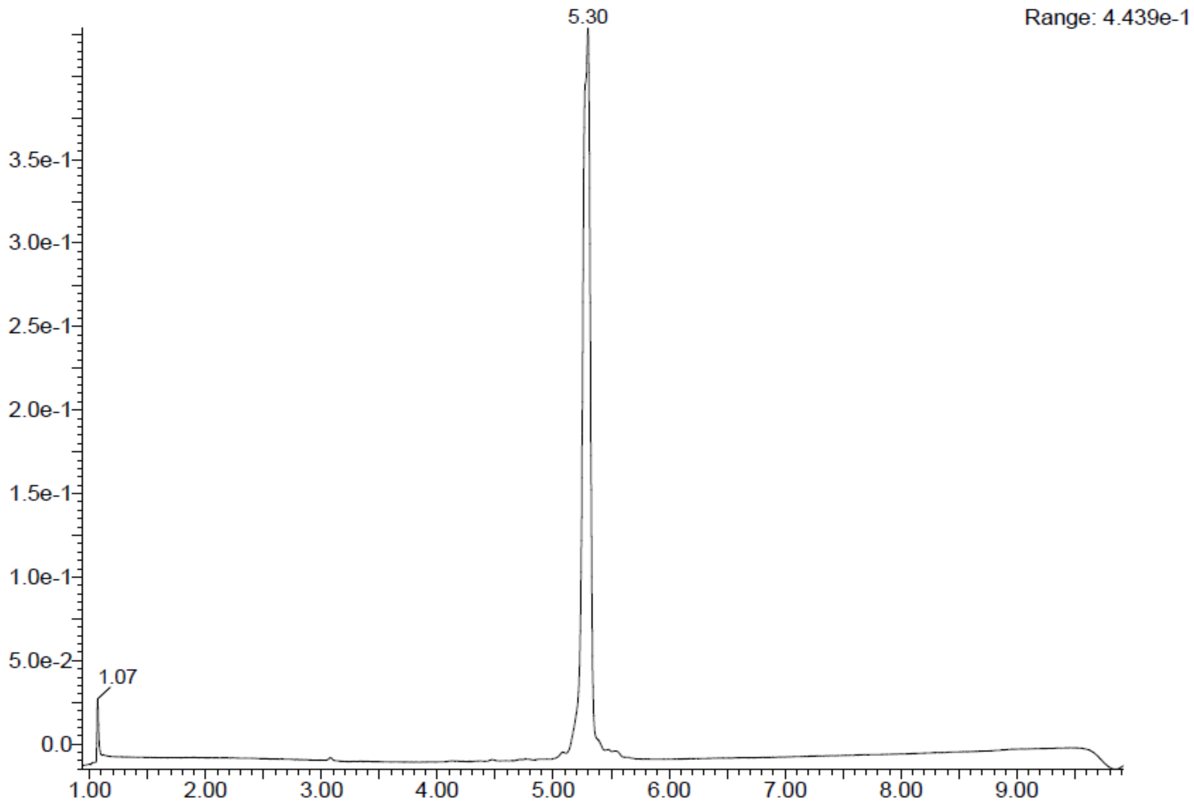

Supplementary Figure S78: Reverse-phase UPLC of crude **ON27** after DMT group removal without purification (UV absorbance at 260 nm vs time in min).

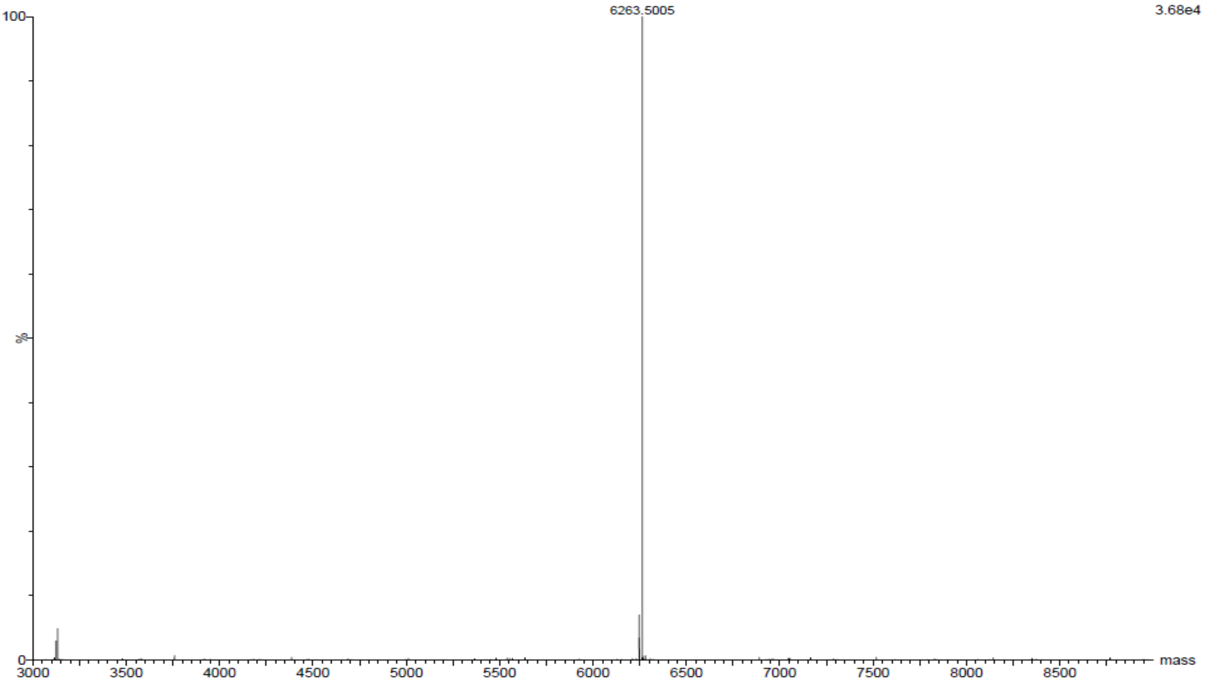

Supplementary Figure S79: Mass spectrum (ES-) of crude **ON27** after DMT group removal without purification. Required **6263.08** Da, found **6263.50** Da. y-axis = relative intensity (%), x-axis = mass in Da.

ON28 THP	CCU CUU ACC UCA GUU ACA	**3.THP**	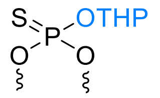

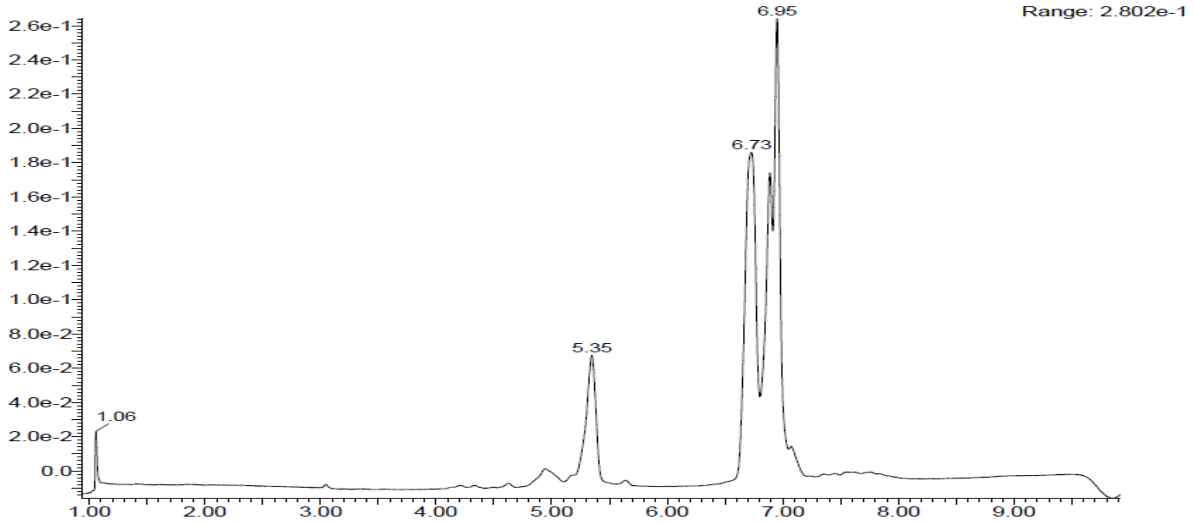

Supplementary Figure S80: Reverse-phase UPLC of **DMTON ON28** after deprotection from solid support (UV absorbance at 260 nm vs time in min).

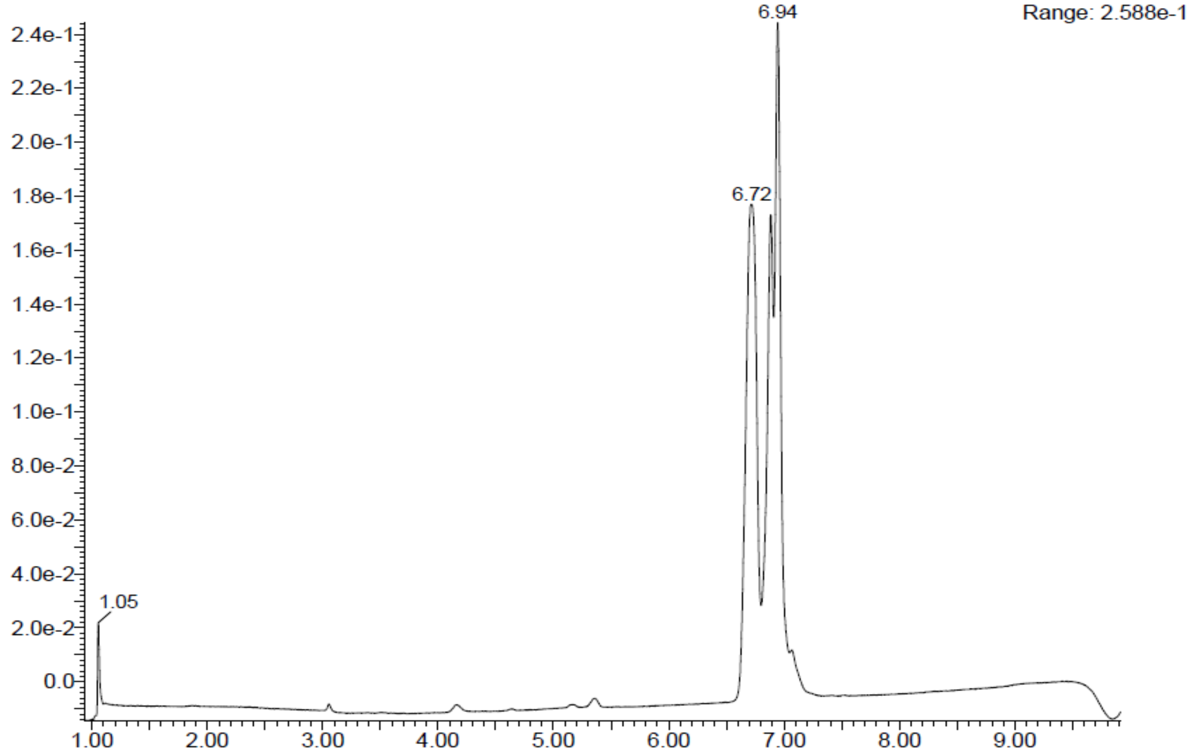

Supplementary Figure S81: Reverse-phase UPLC of **DMTON ON28** after purification by HPLC (UV absorbance at 260 nm vs time in min).

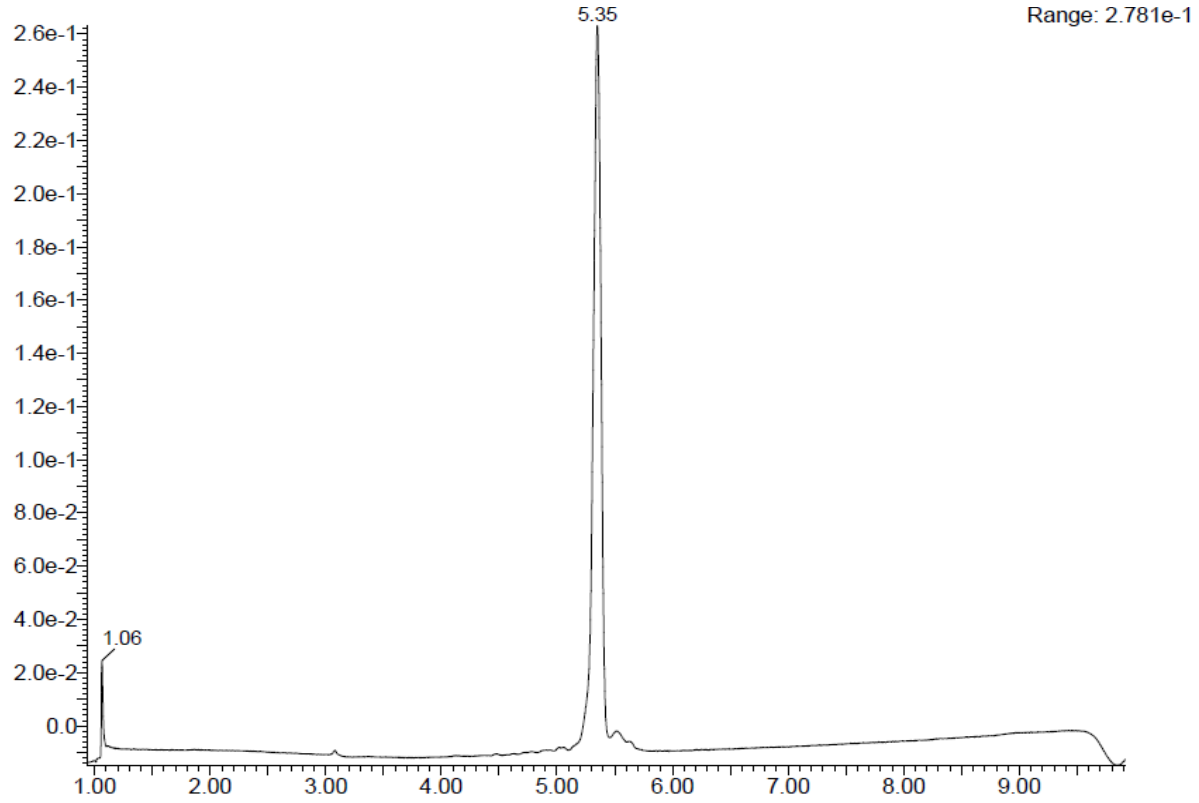

Supplementary Figure S82: Reverse-phase UPLC of crude **ON28** after DMT group removal without purification (UV absorbance at 260 nm vs time in min).

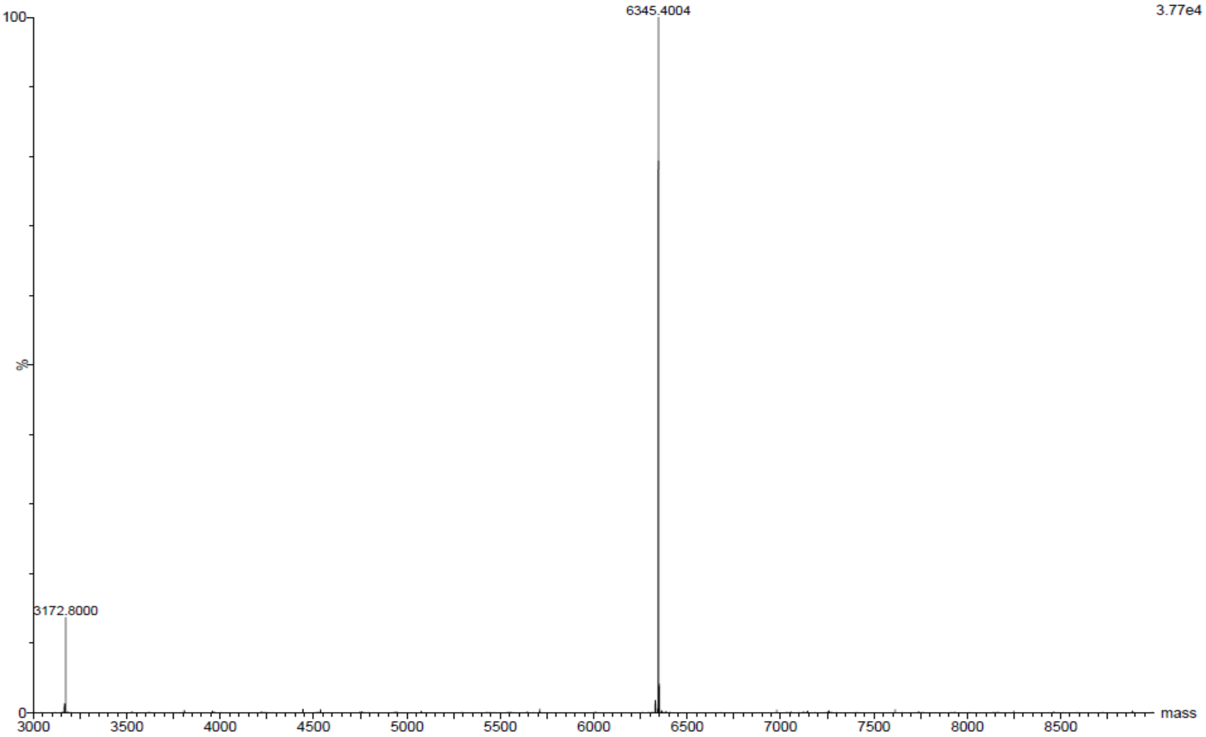

Supplementary Figure S83: Mass spectrum (ES-) of crude **ON28** after DMT group removal without purification. Required **6345.18** Da, found **6345.40** Da. y-axis = relative intensity (%), x-axis = mass in Da.

ON29 THP	CCU CUU ACC UCA GUT ACA	**THP**	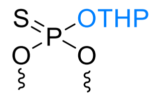

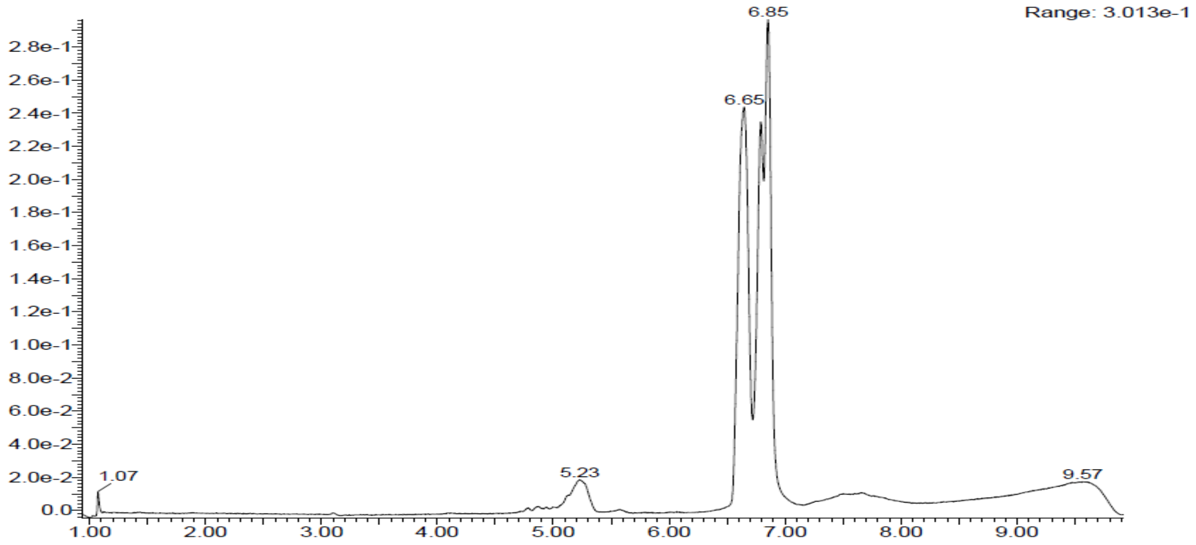

Supplementary Figure S84: Reverse-phase UPLC of **DMTON ON29** after deprotection from solid support (UV absorbance at 260 nm vs time in min).

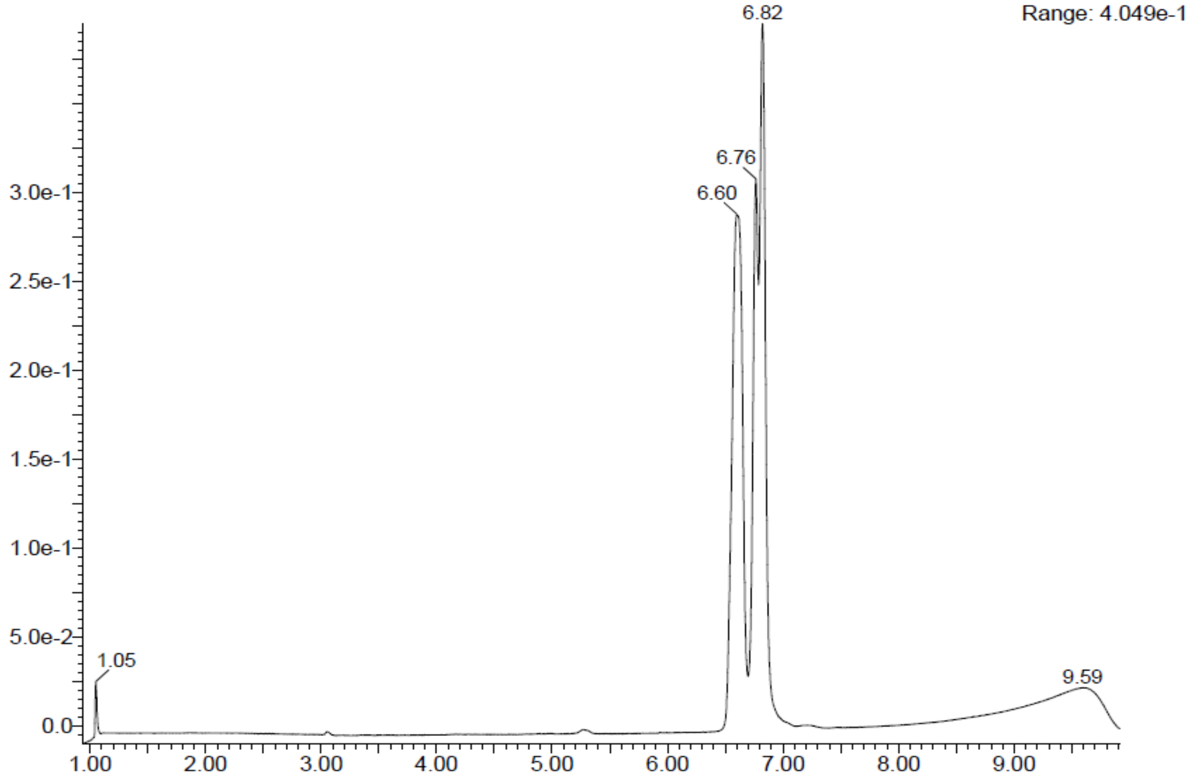

Supplementary Figure S85: Reverse-phase UPLC of **DMTON ON29** after purification by HPLC (UV absorbance at 260 nm vs time in min).

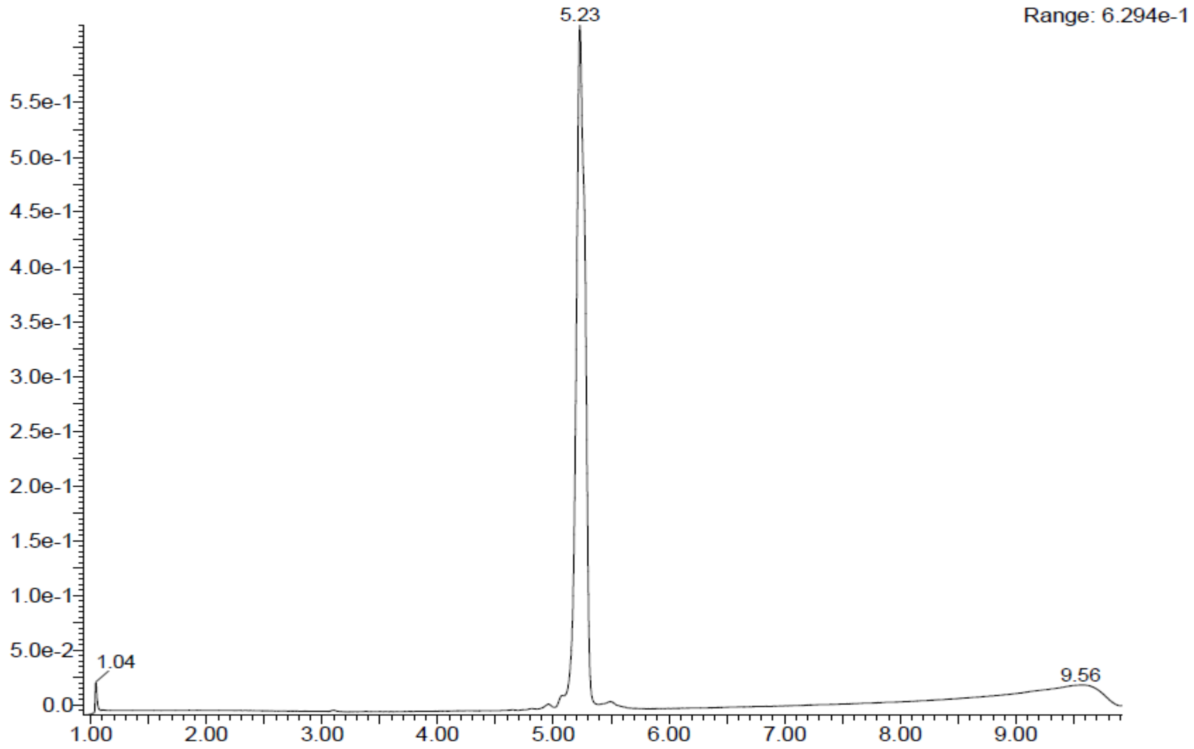

Supplementary Figure S86: Reverse-phase UPLC of crude **ON29** after DMT group removal without purification (UV absorbance at 260 nm vs time in min).

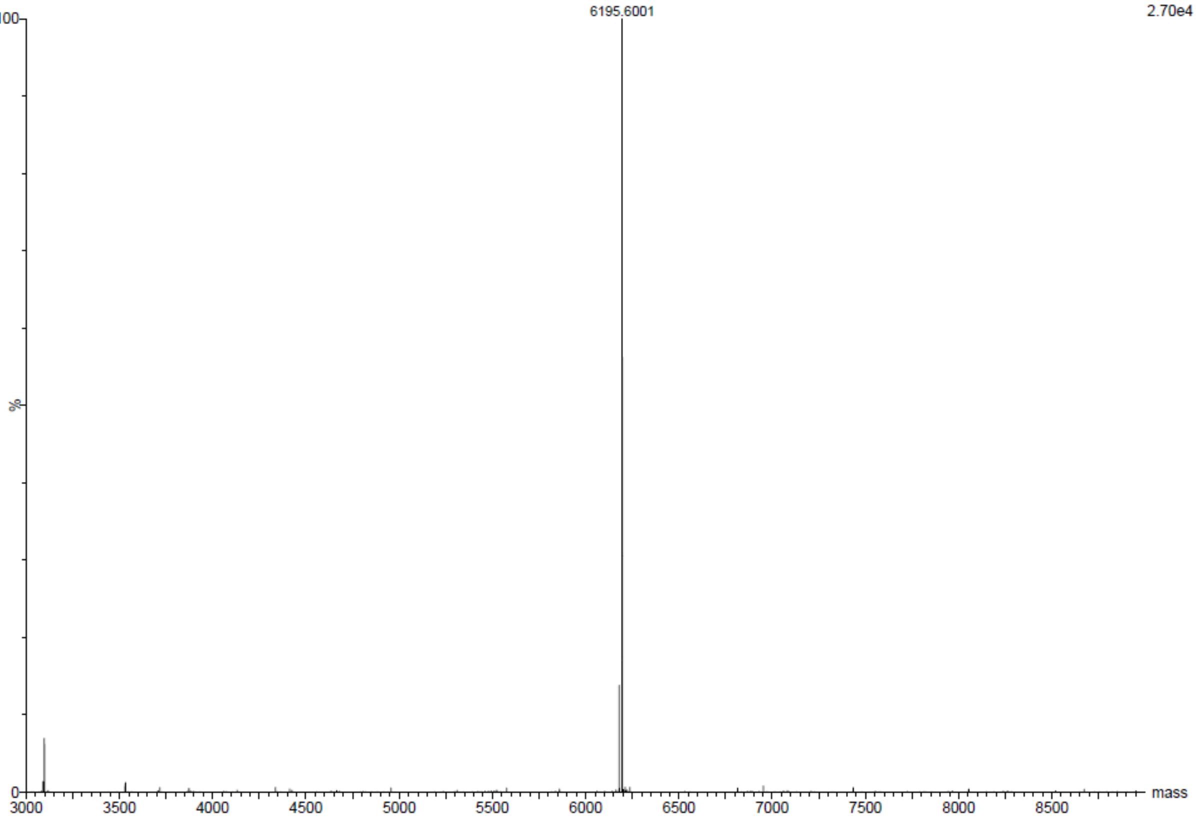

Supplementary Figure S87: Mass spectrum (ES-) of crude **ON29** after DMT group removal without purification. Required **6195.01** Da, found **6195.60** Da. y-axis = relative intensity (%), x-axis = mass in Da.

ON30 THP	CCU CUT ACC UCA GUT ACA	**2.THP**	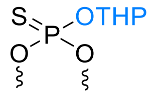

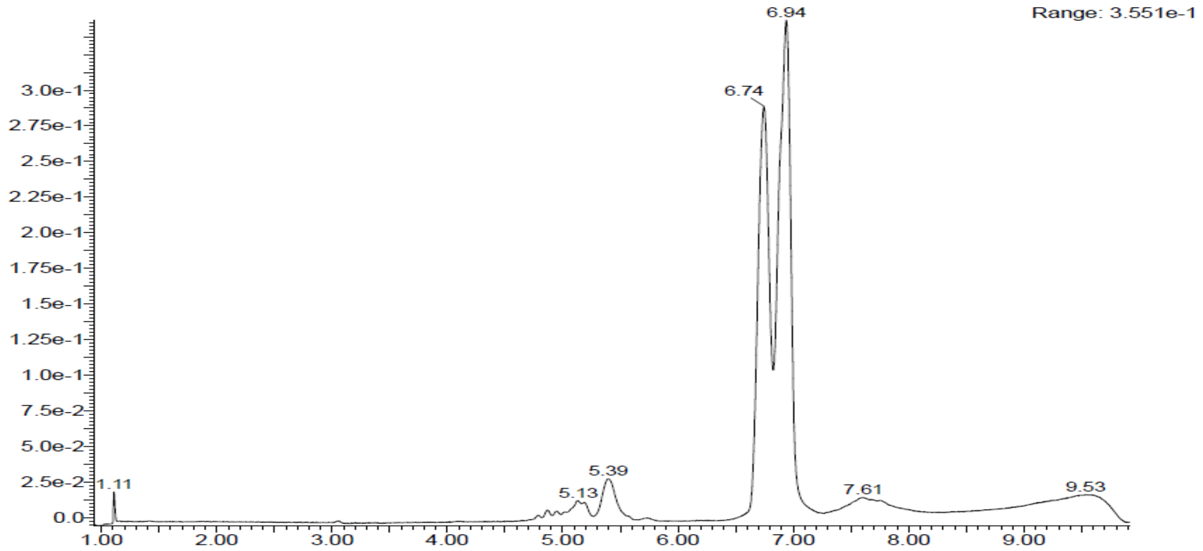

Supplementary Figure S88: Reverse-phase UPLC of crude **DMTON ON30** after deprotection from solid support (UV absorbance at 260 nm vs time in min).

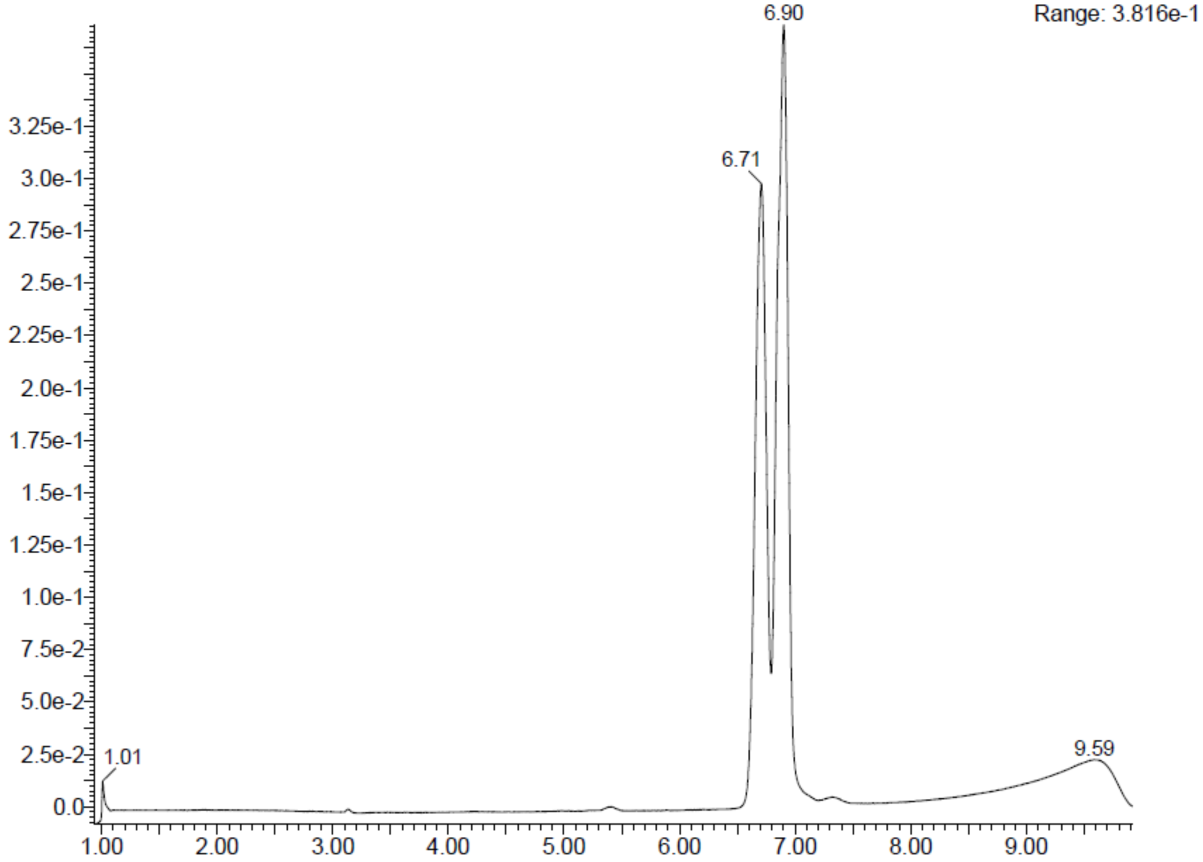

Supplementary Figure S89: Reverse-phase UPLC of **DMTON ON30** after purification by HPLC (UV absorbance at 260 nm vs time in min).

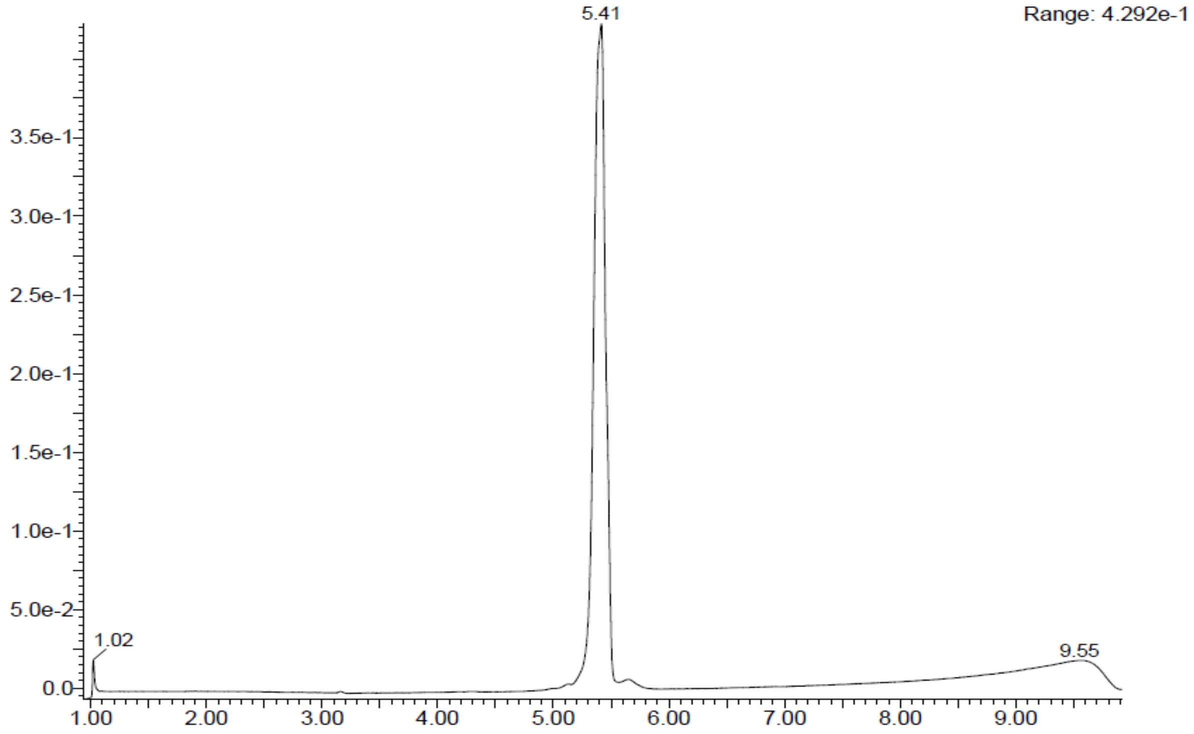

Supplementary Figure S90: Reverse-phase UPLC crude **ON30** (UV absorbance at 260 nm vs time in min)

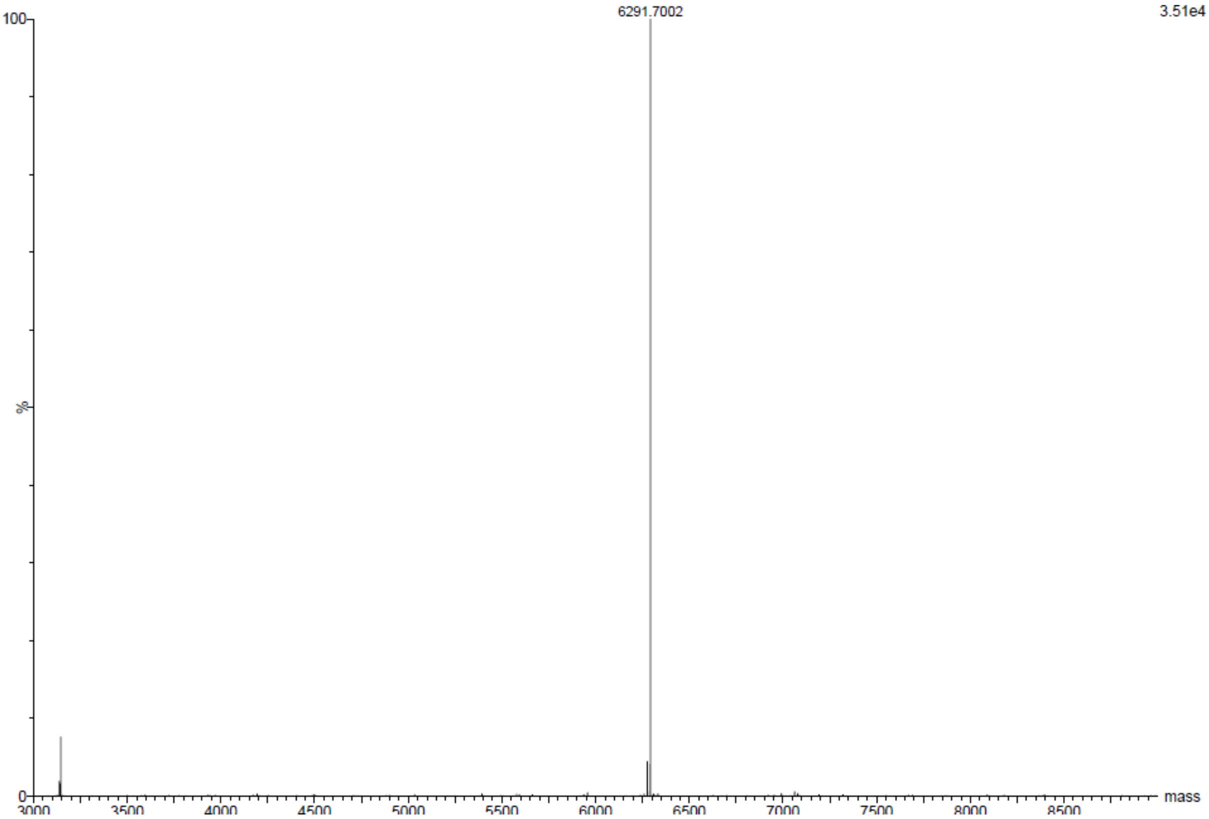

Supplementary Figure S91: Mass spectrum (ES-) of crude **ON30** after DMT group removal without purification. Required **6291.14** Da, found **6291.70** Da. y-axis = relative intensity (%), x-axis = mass in Da.

ON31 THP	CCT CUT ACC TCA GUT ACA	**4.THP**	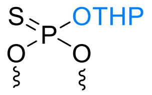

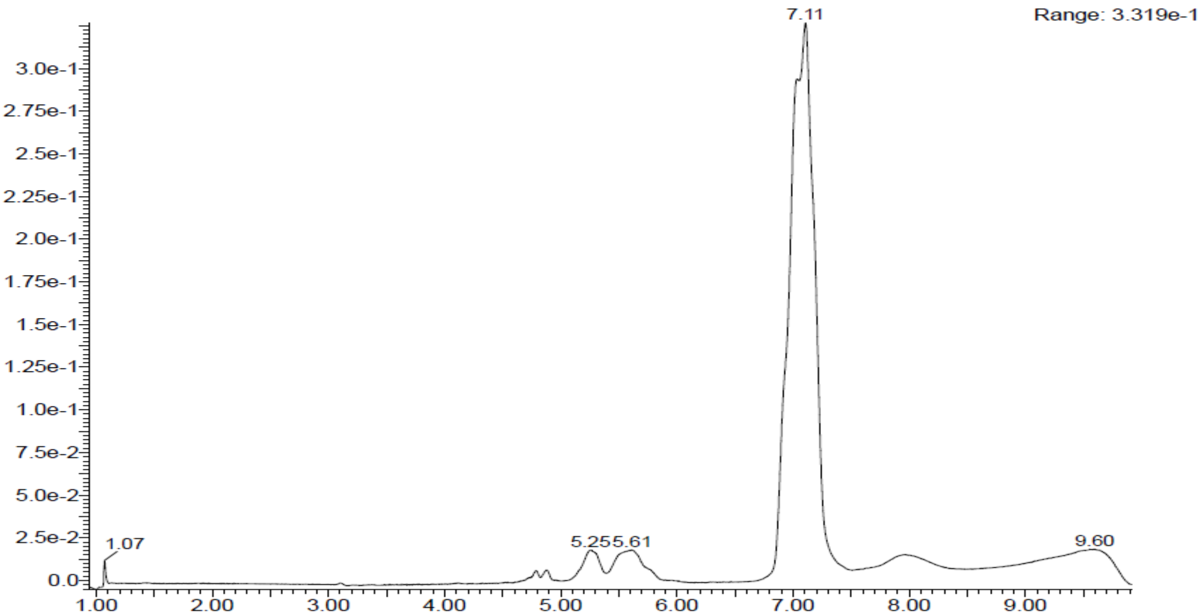

Supplementary Figure S92: Reverse-phase UPLC of crude **DMTON ON31** after deprotection from solid support (UV absorbance at 260 nm vs time in min)

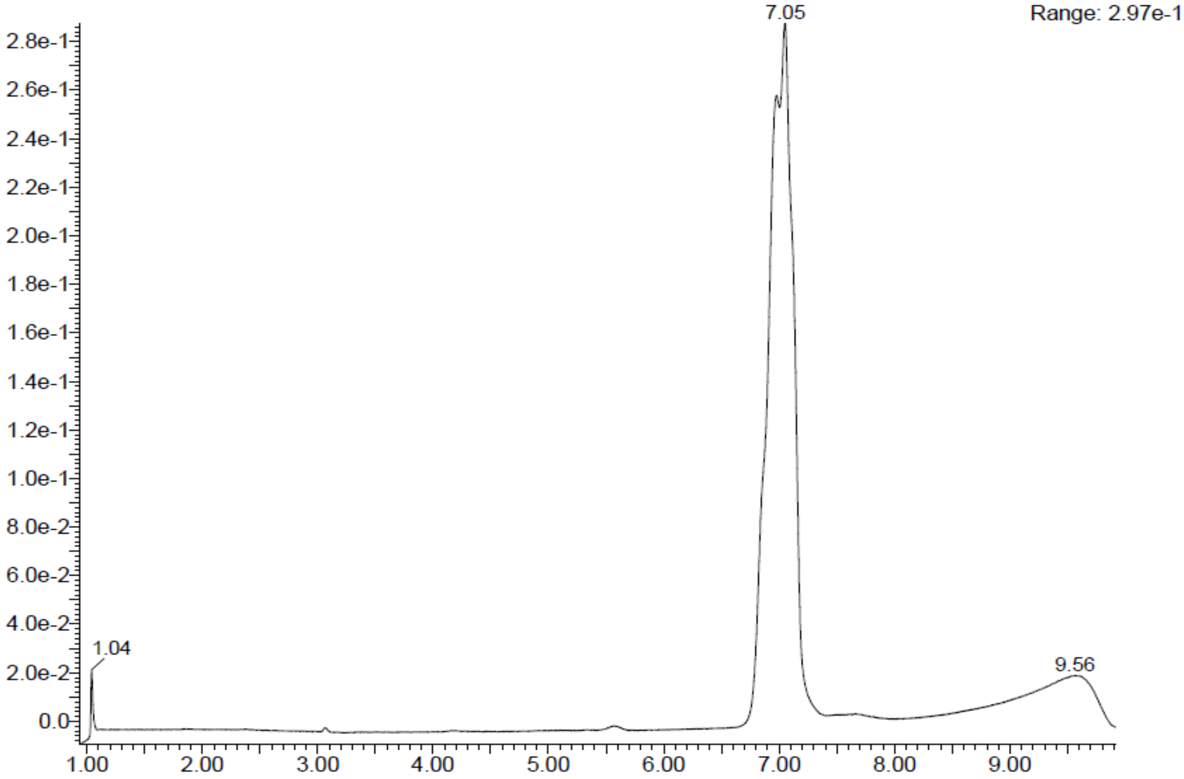

Supplementary Figure S93: Reverse-phase UPLC of **DMTON ON31** after purification by HPLC (UV absorbance at 260 nm vs time in min).

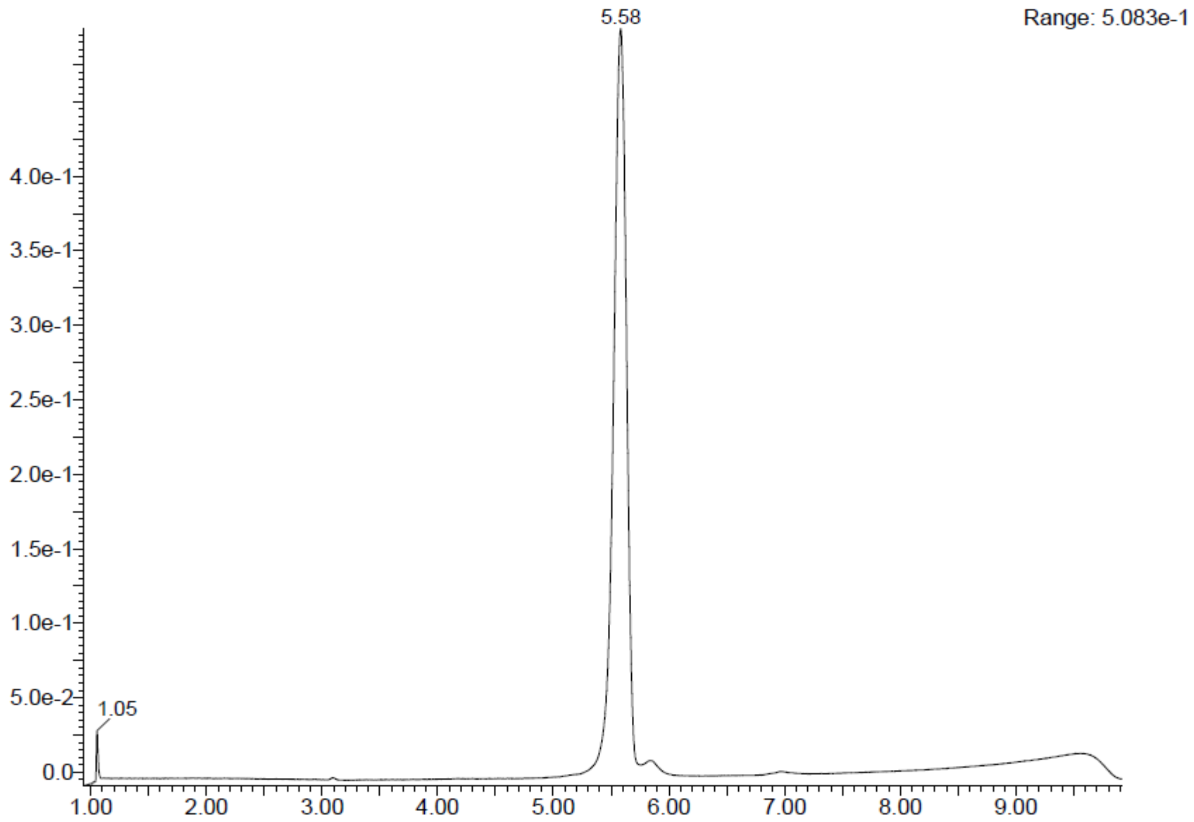

Supplementary Figure S94: Reverse-phase UPLC crude **ON31** after DMT group removal without purification. (UV absorbance at 260 nm vs time in min).

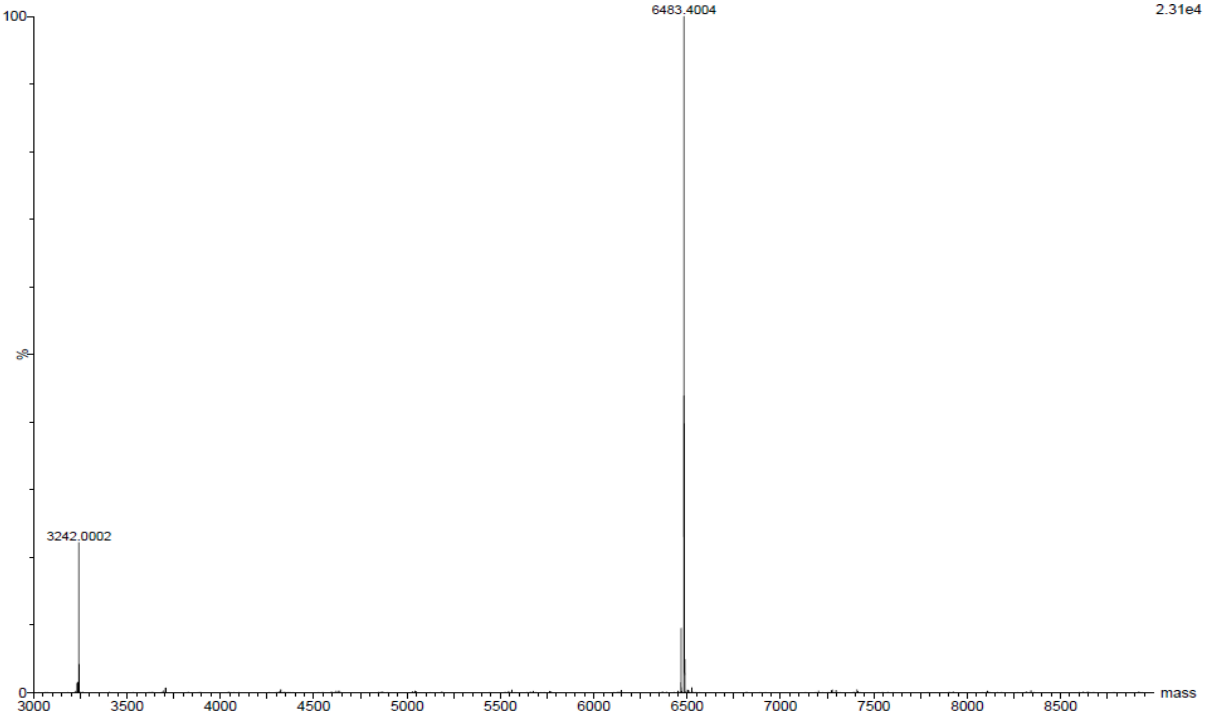

Supplementary Figure S95: Mass spectrum (ES-) of crude **ON31** after DMT group removal without purification. Required **6483.39** Da, found **6483.40** Da. y-axis = relative intensity (%), x-axis = mass in Da.

ON32 THP	CCT CTT ACC TCA GTT ACA	**6.THP**	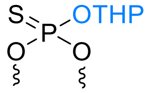

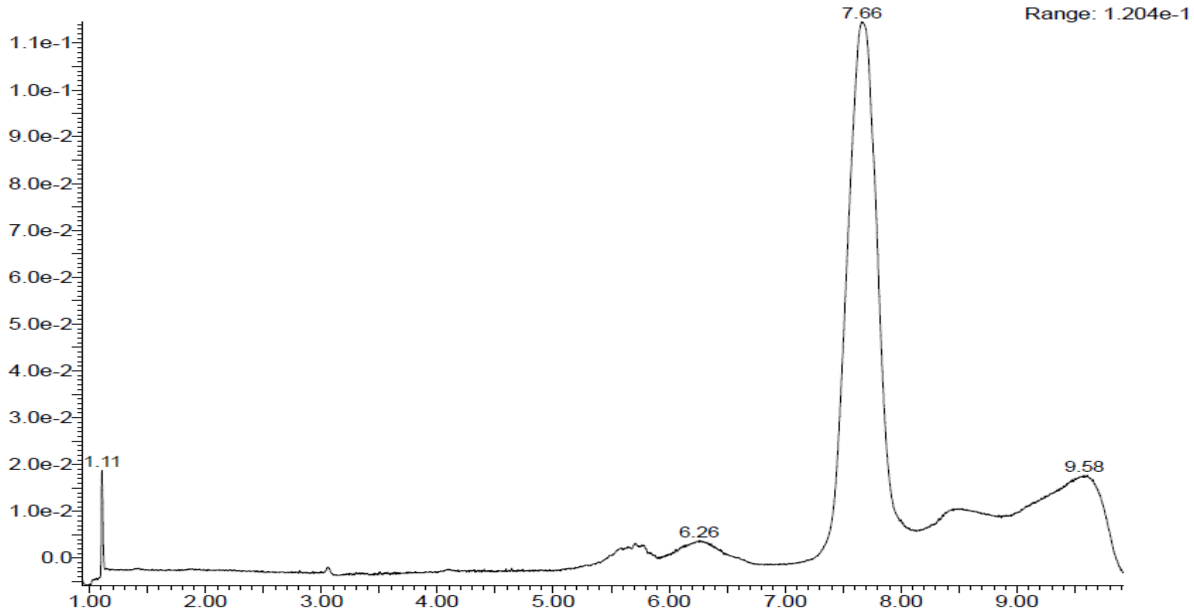

Supplementary Figure S96: Reverse-phase UPLC of crude DMTON **ON32** after deprotection from solid support (UV absorbance at 260 nm vs time in min).

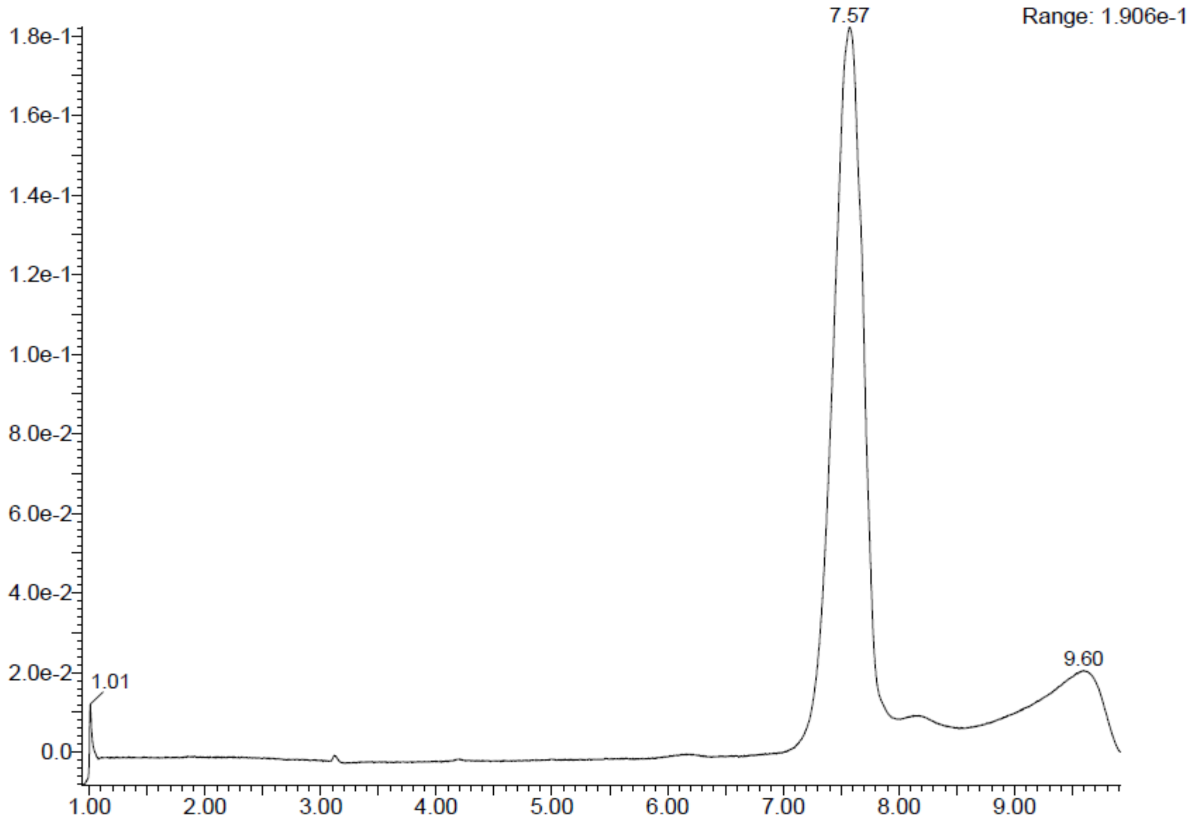

Supplementary Figure S97: Reverse-phase UPLC of **DMTON ON32** after purification by HPLC (UV absorbance at 260 nm vs time in min).

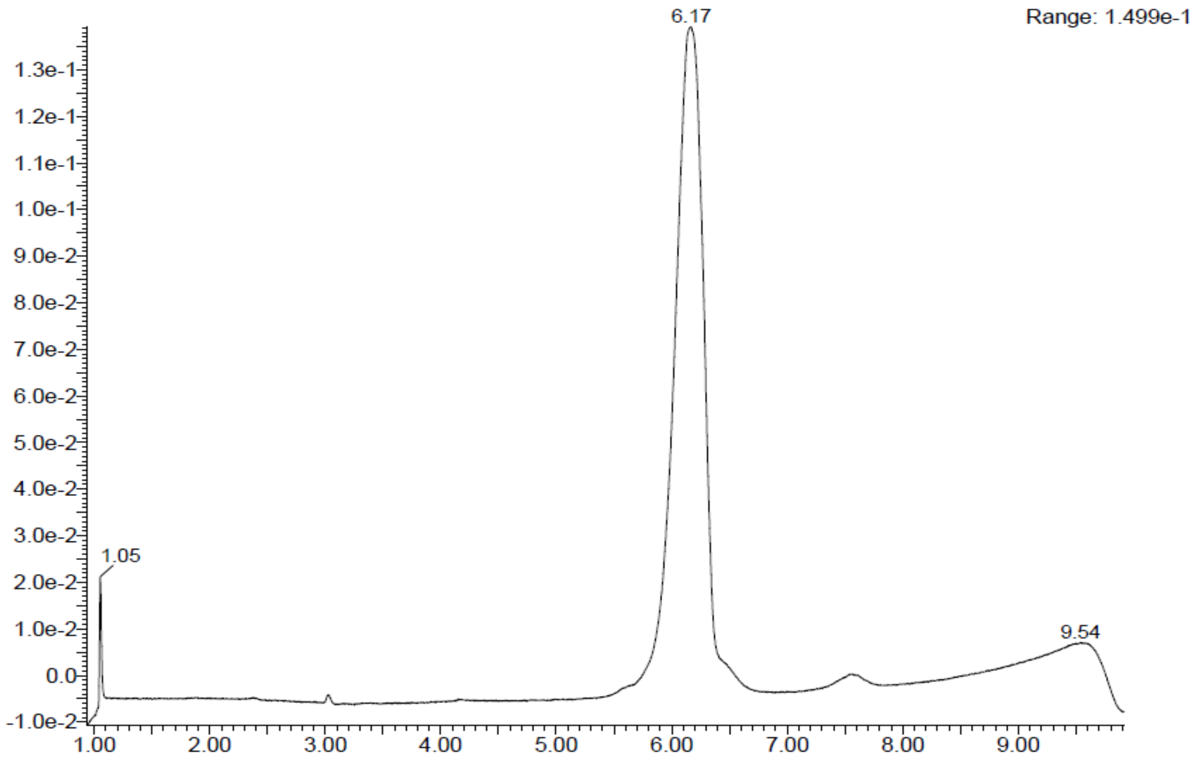

Supplementary Figure S98: Reverse-phase UPLC crude **ON32** after DMT group removal without purification (UV absorbance at 260 nm vs time in min).

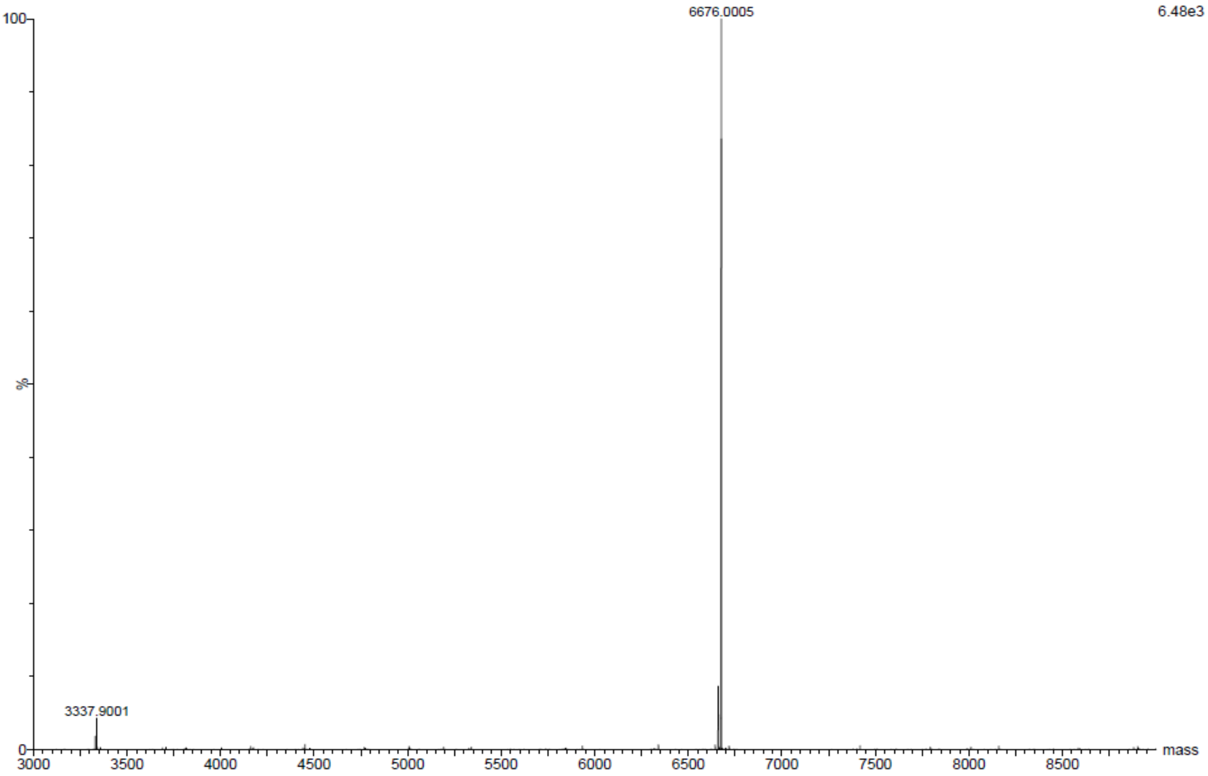

Supplementary Figure S99: Mass spectrum (ES-) of crude **ON32** after DMT group removal without purification. Required **6675.65** Da, found **6676.00** Da. y-axis = relative intensity (%), x-axis = mass in Da.

ON33 THP	CCT CUT ACC TCA GUT ACA	**7.THP**	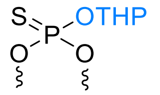

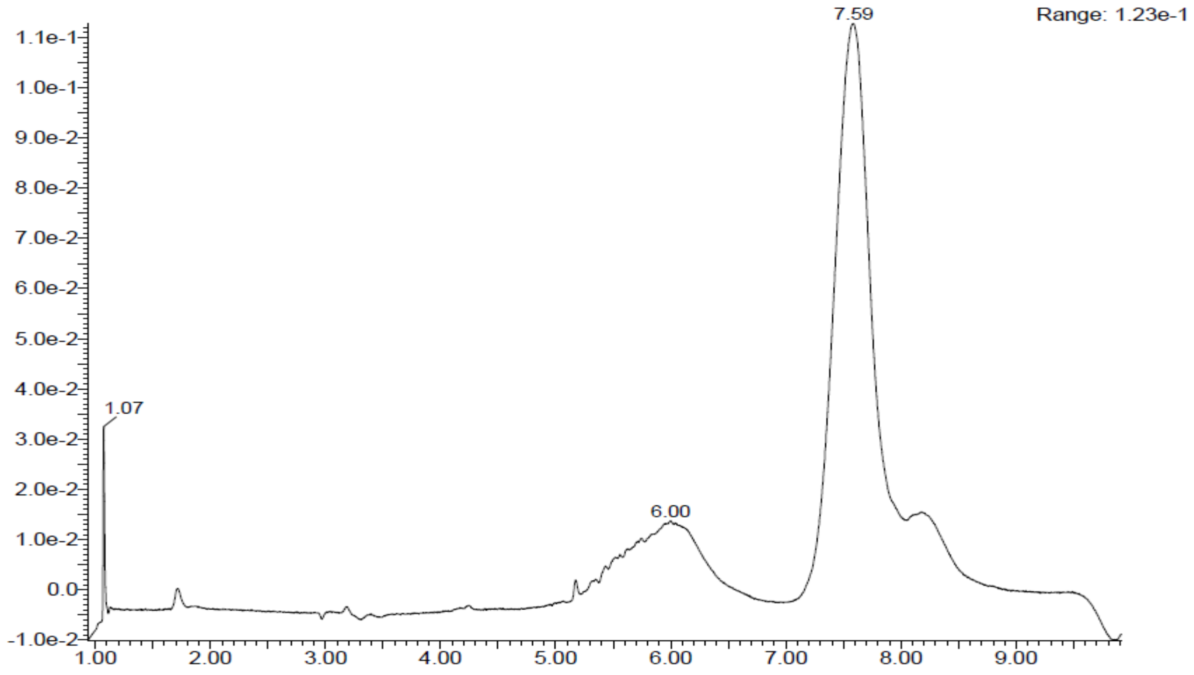

Supplementary Figure S100: Reverse-phase UPLC of crude **DMTON ON33** after deprotection from solid support (UV absorbance at 260 nm vs time in min).

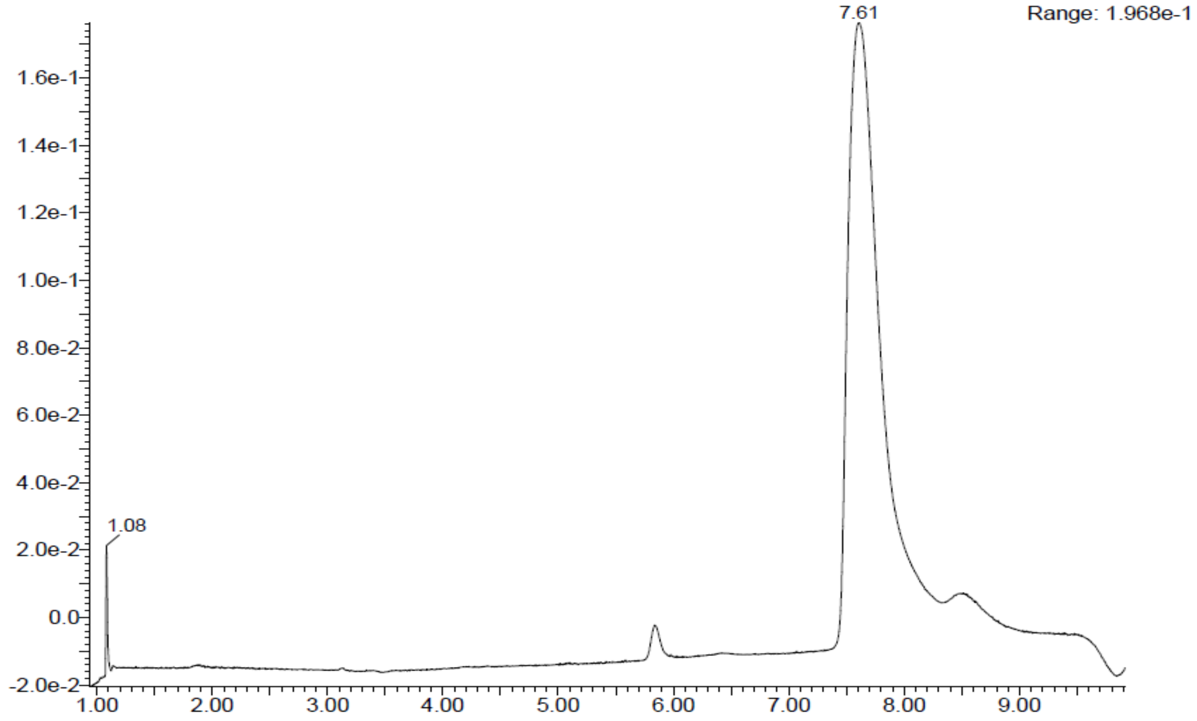

Supplementary Figure S101: Reverse-phase UPLC of **DMTON ON33** after purification by HPLC (UV absorbance at 260 nm vs time in min).

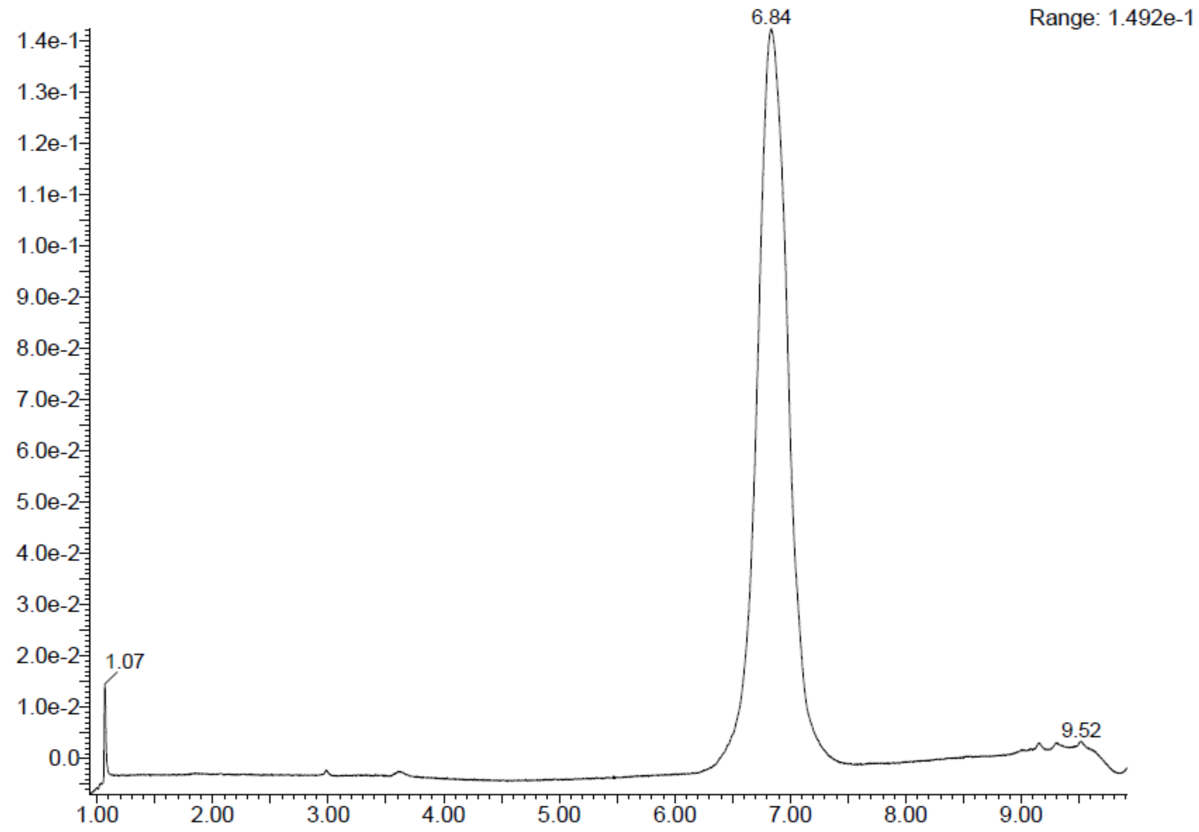

Supplementary Figure S102: Reverse-phase UPLC **ON33** after DMT group removal followed by HPLC purification (UV absorbance at 260 nm vs time in min).

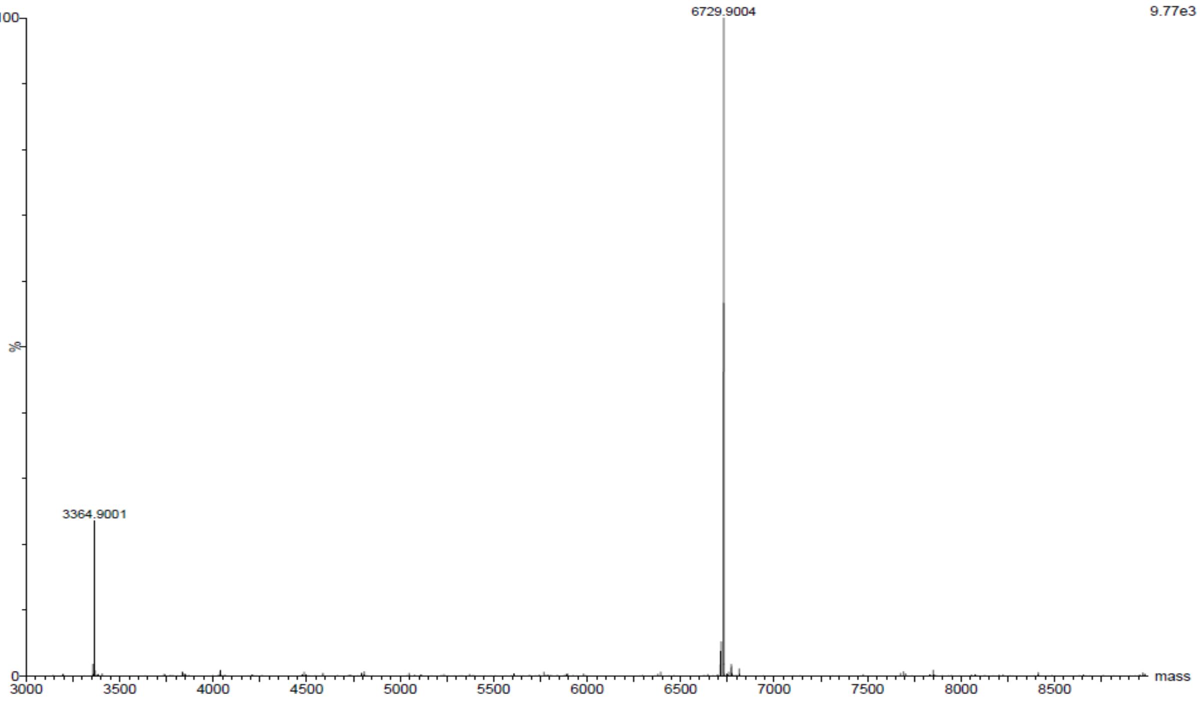

Supplementary Figure S103: Mass spectrum (ES-) of crude **ON33** after DMT group removal without purification. Required **6729.70** Da, found **6729.90** Da. y-axis = relative intensity (%), x-axis = mass in Da.

ON34 THP	CCT CTT ACC TCA GTT ACA	**9.THP**	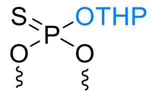

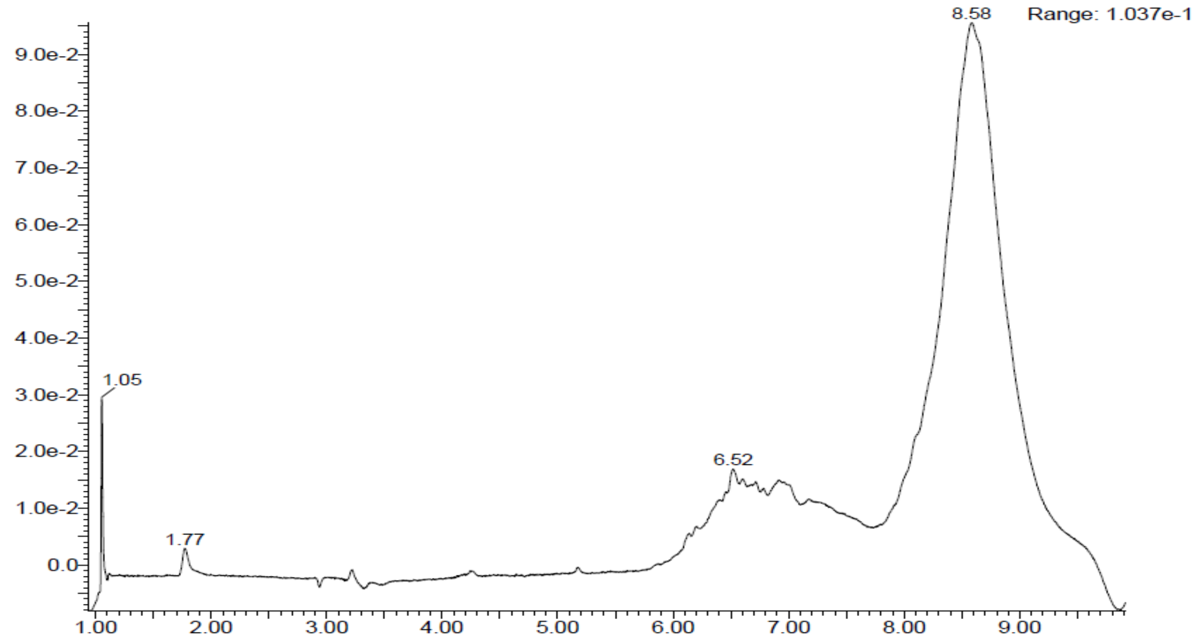

Supplementary Figure S104: Reverse-phase UPLC of crude DMTON **ON34** after deprotection from solid support (UV absorbance at 260 nm vs time in min).

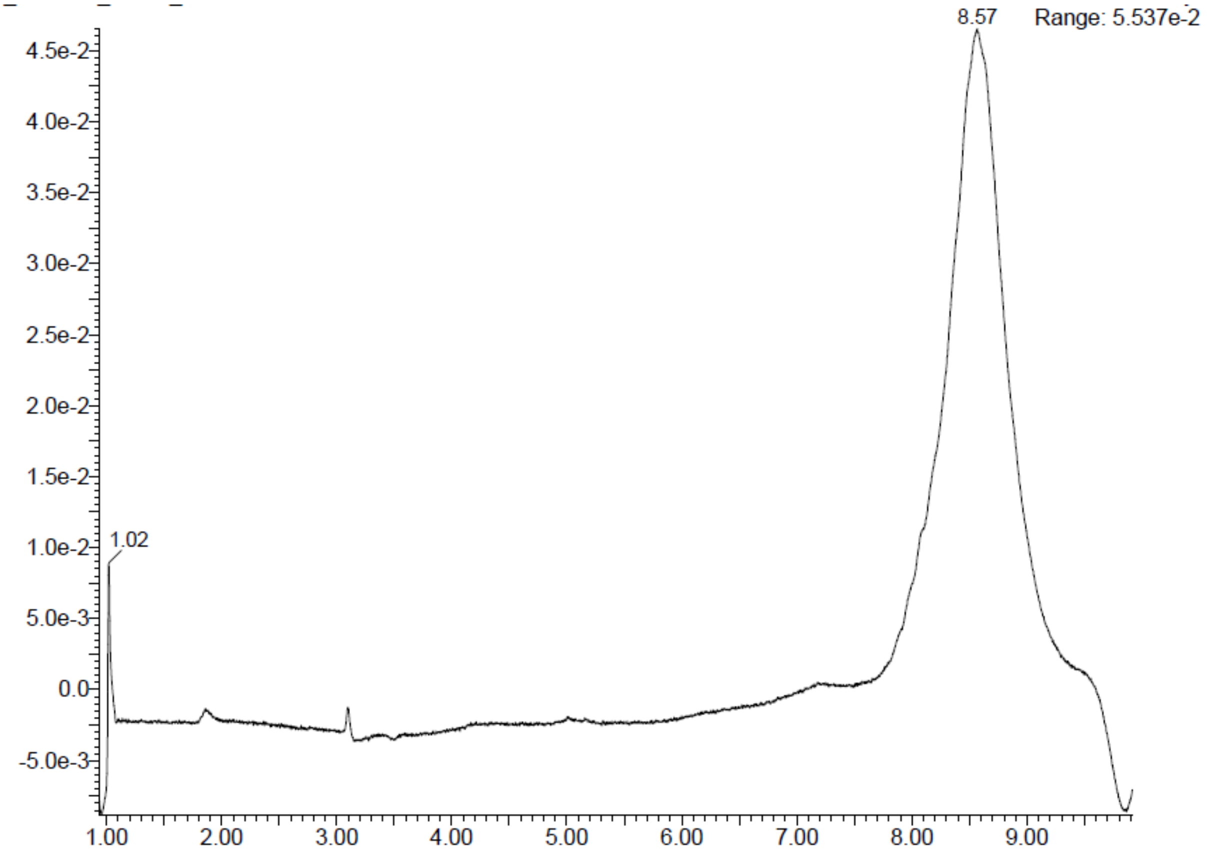

Supplementary Figure S105: Reverse-phase UPLC of **DMTON ON34** after purification by HPLC (UV absorbance at 260 nm vs time in min).

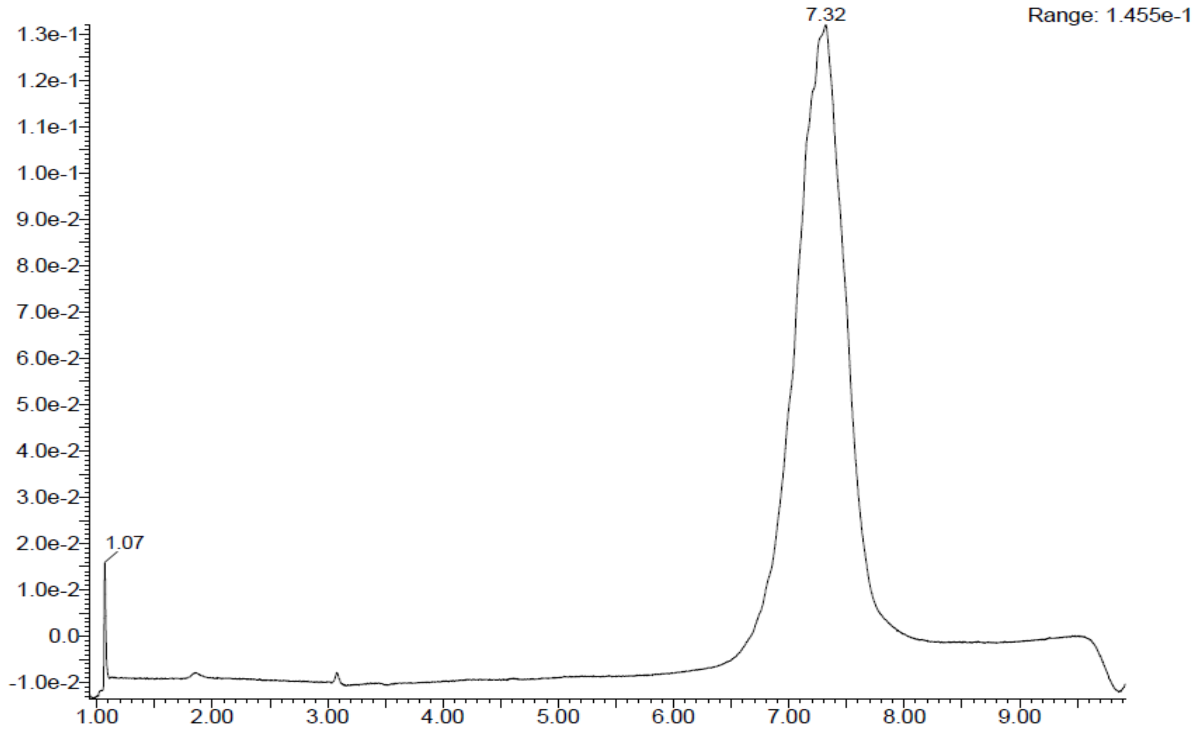

Supplementary Figure S106: Reverse-phase UPLC **ON34** after DMT group removal followed by HPLC purification (UV absorbance at 260 nm vs time in min).

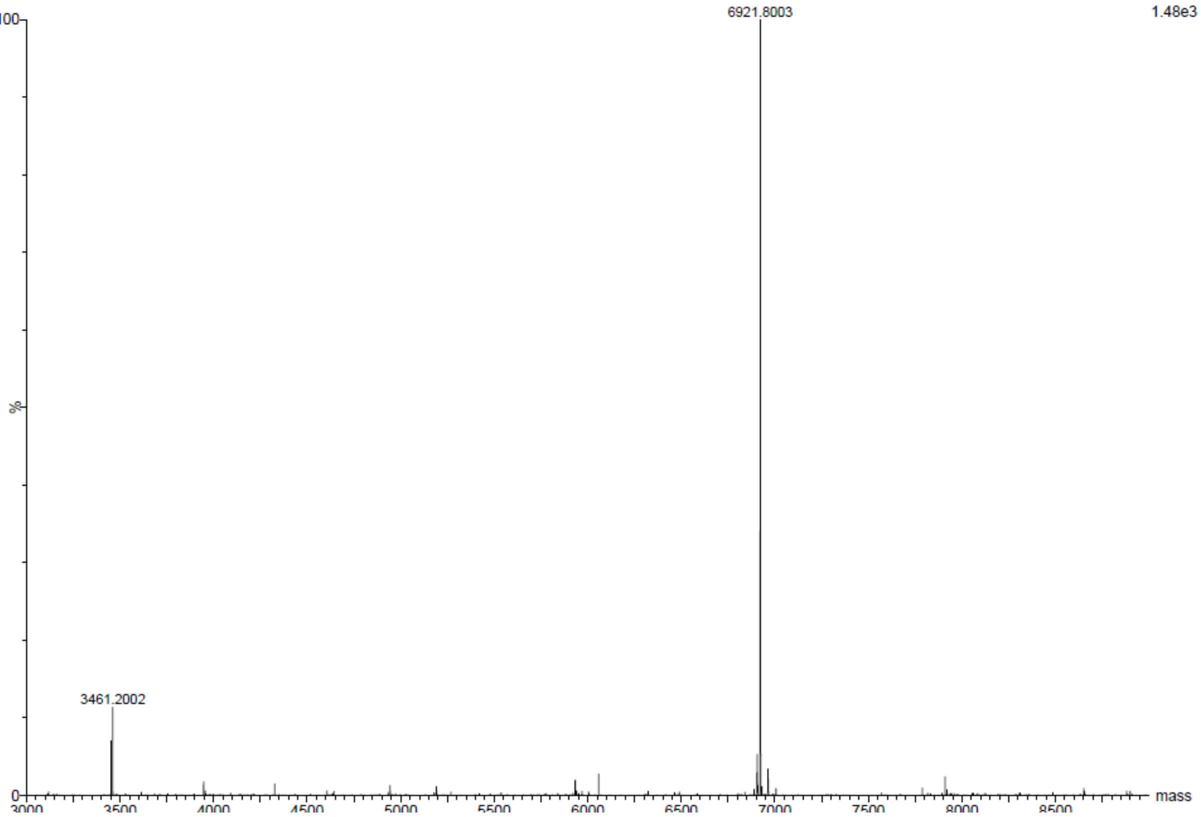

Supplementary Figure S107: Mass spectrum (ES-) of crude **ON34** after DMT removal without purification. Required **6921.96** Da, found **6921.80** Da. y-axis = relative intensity (%), x-axis = mass in Da.

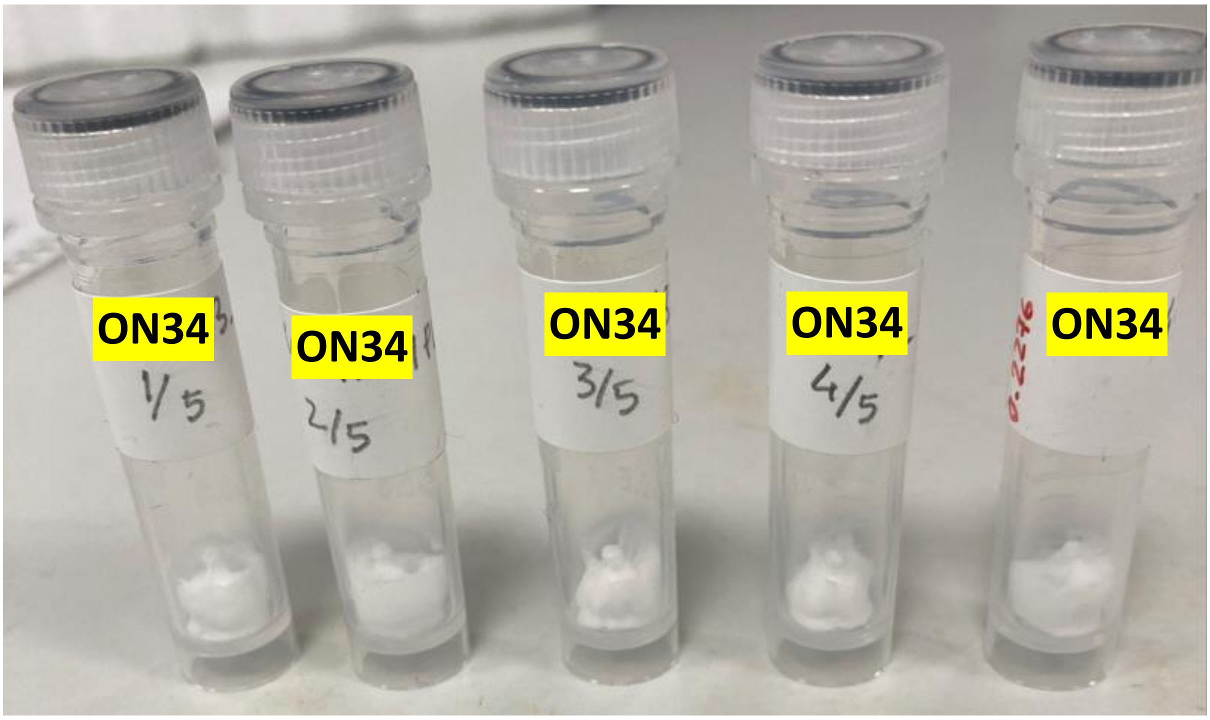

ON34 in five different vials (~40 nm each).

2.5 C16 PTTE oligonucleotides

ON35 C16	CCU CUU ACC UCA GUU ACA	**1.** $C_{16}H_{33}$	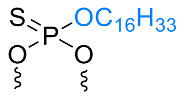

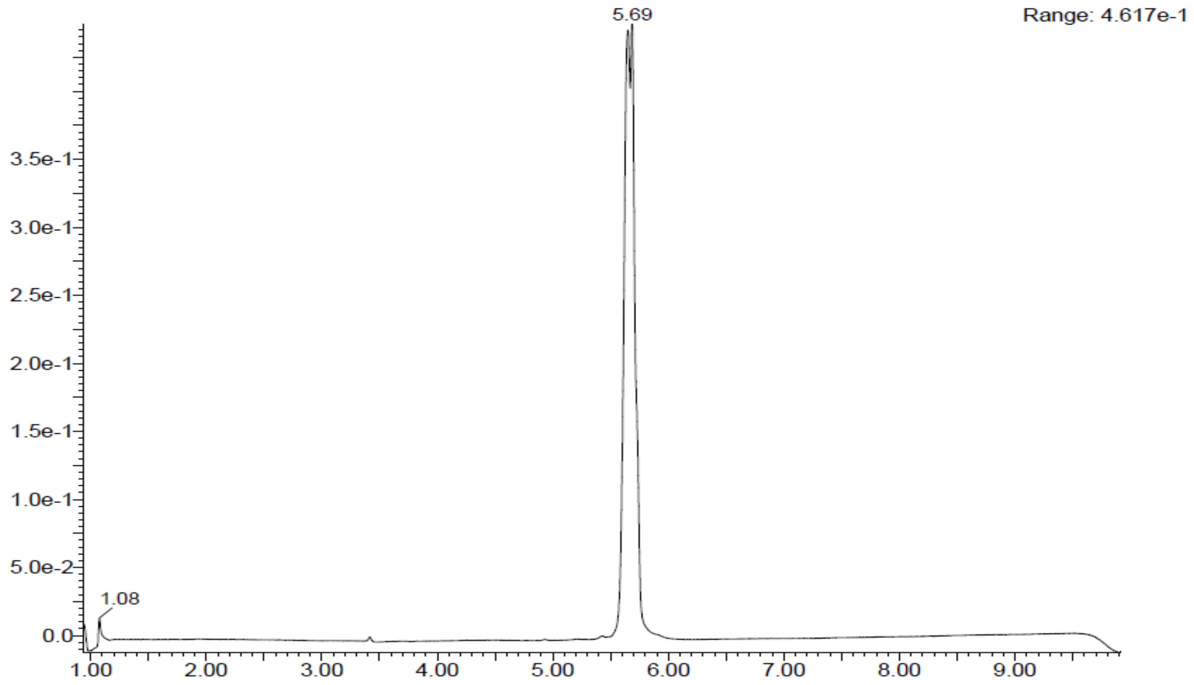

Supplementary Figure S108: Reverse-phase UPLC **ON35** (UV absorbance at 260 nm vs time in min).

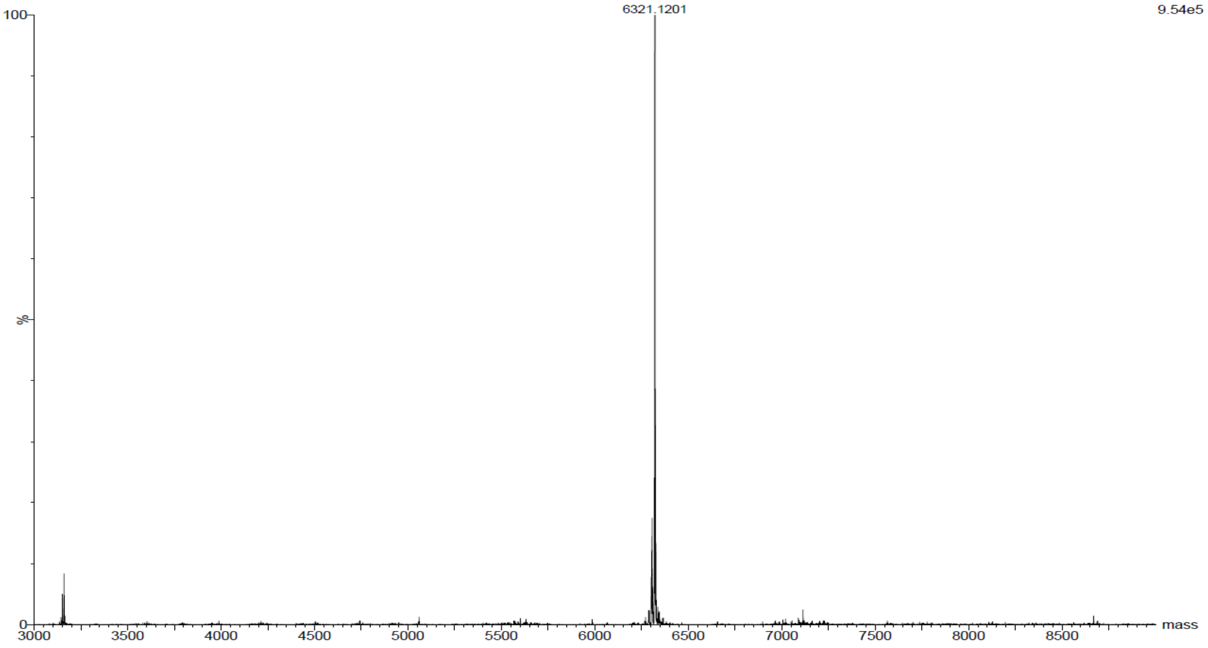

Supplementary Figure S109: Mass spectrum (ES-) of **ON35**. Required **6321.29** Da, found **6321.12** Da. y-axis = relative intensity (%), x-axis = mass in Da.

ON36 C16	CCU CUU ACC UCA GUU ACA	**2.$C_{16}H_{33}$**	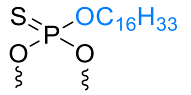

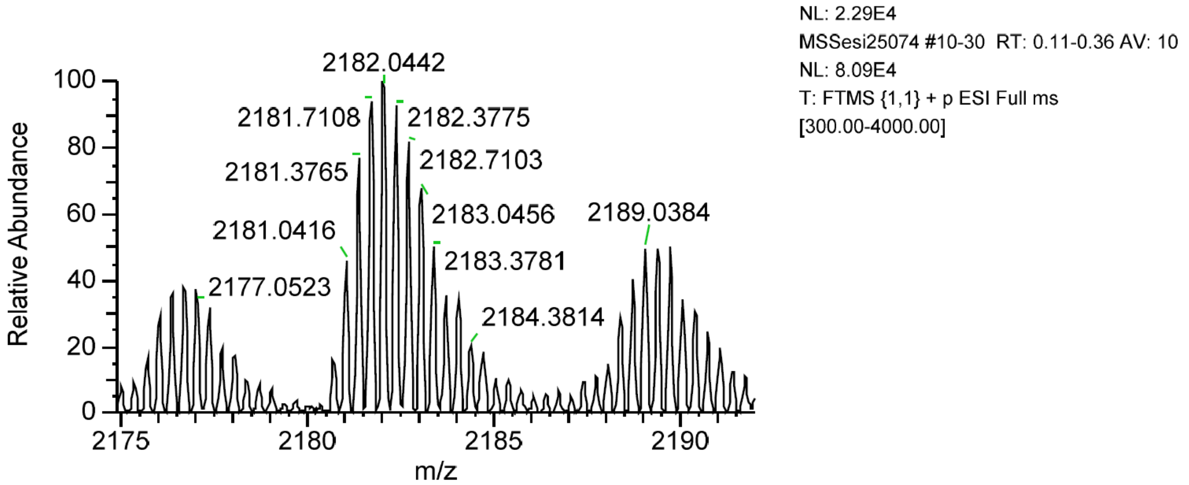

Supplementary Figure S110: Expanded Mass spectrum (ESI) of **ON36.** *m/z* $[M+3H]^{3+}$ calc. for $C_{217}H_{312}N_{58}O_{109}P_{17}S_{17}$ 2182.0499; found 2182.0442. Mass accuracy 2.6 ppm.

ON37 C16	CCU CUU ACC UCA GUU ACA	**$2.C_{16}H_{33}$**	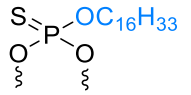

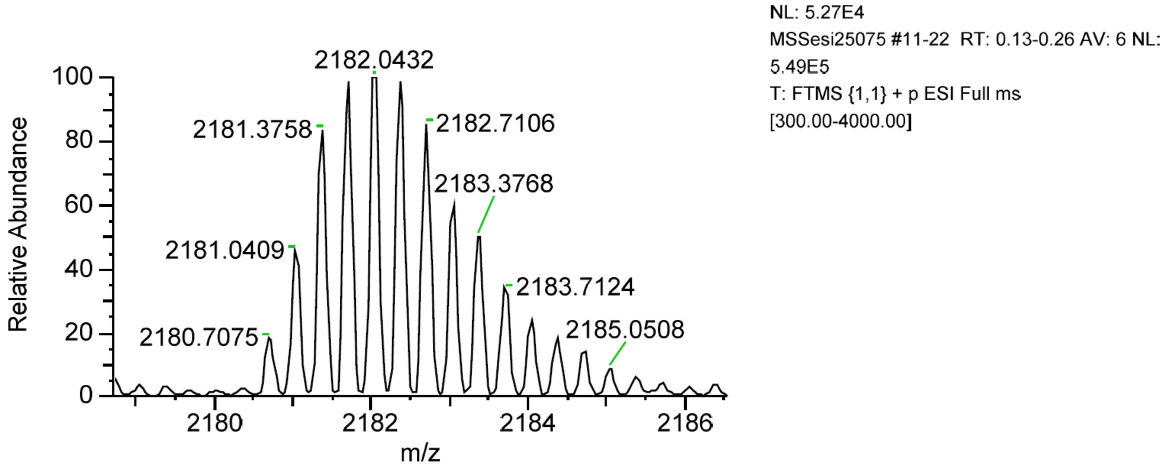

Supplementary Figure S111: Expanded Mass spectrum (ESI) of **ON37.** *m/z* $[M+3H]^{3+}$ calc. for $C_{217}H_{312}N_{58}O_{109}P_{17}S_{17}$ 2182.0499; found 2182.0432. Mass accuracy 3.1 ppm.

ON38 C16	CCU CUU ACC UCA GUU ACA	**3.$C_{16}H_{33}$**	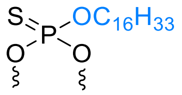

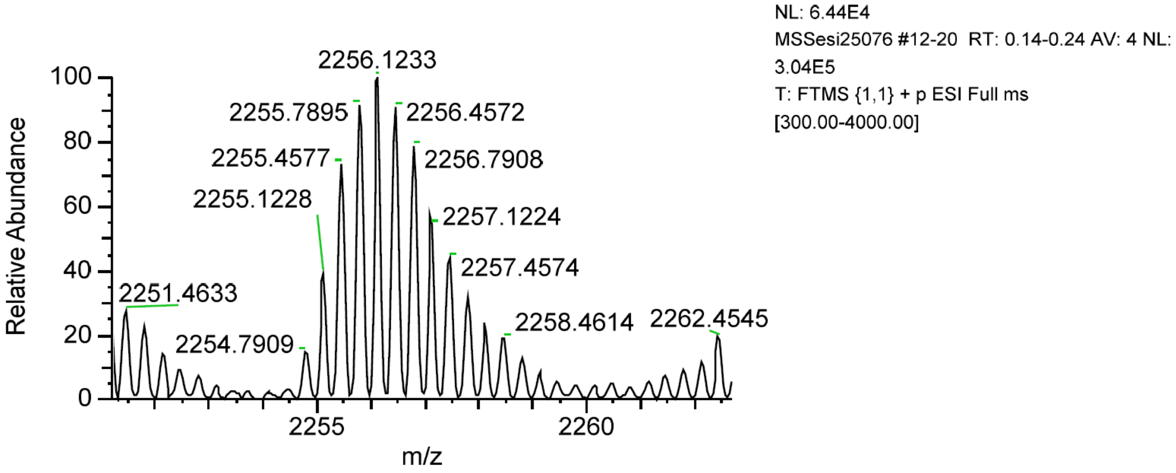

Supplementary Figure S112: Expanded Mass spectrum (ESI) of **ON38.** *m/z* $[M+3H]^{3+}$ calc. for $C_{233}H_{344}N_{58}O_{109}P_{17}S_{17}$ 2256.1311; found 2256.1233. Mass accuracy 3.4 ppm.

2.6 Hexynyl PTTE oligonucleotides

ON39 Hexynyl	CCU CUU ACC UCA GUU ACA	**Hexyn**	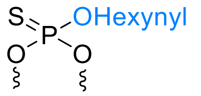

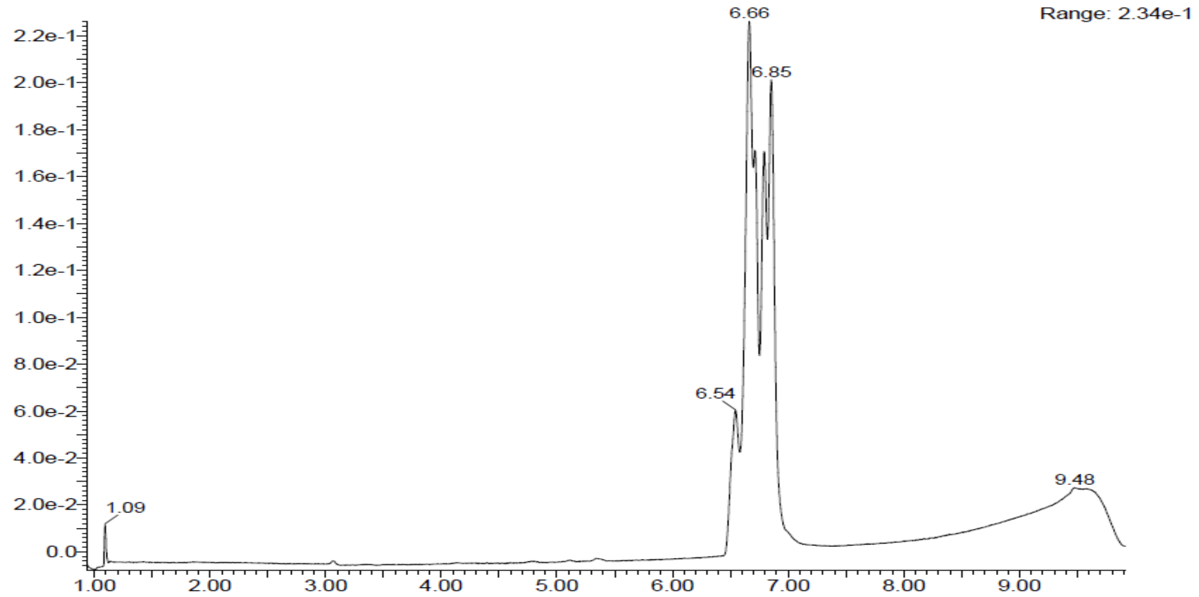

Supplementary Figure S113: Reverse-phase UPLC of **DMTON ON39** (UV absorbance at 260 nm vs time in min).

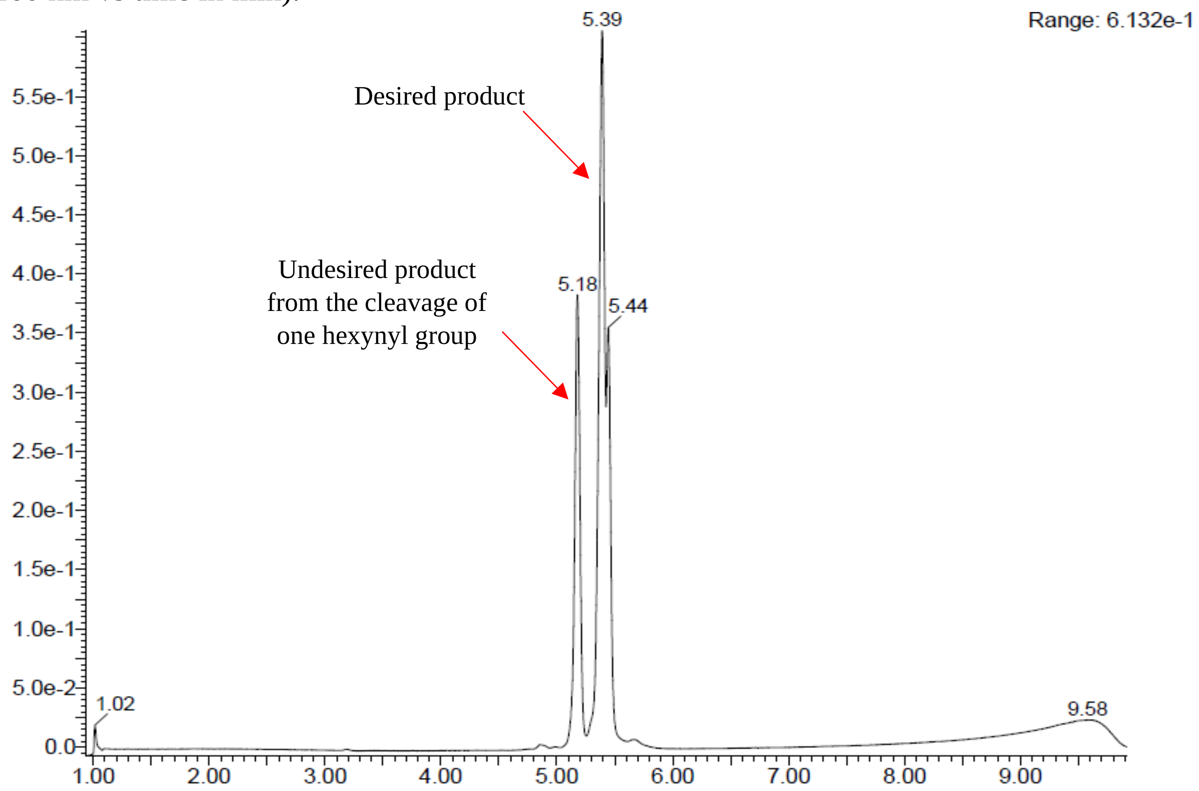

Supplementary Figure S114: Reverse-phase UPLC of crude **ON39** after removal of DMT group (UV absorbance at 260 nm vs time in min).

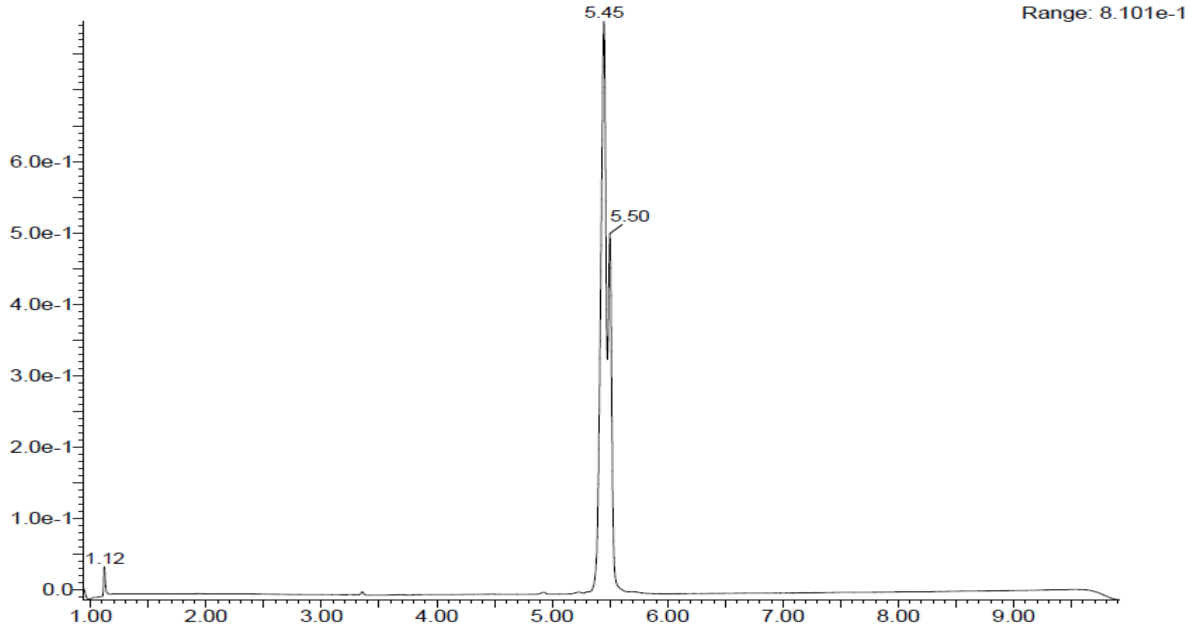

Supplementary Figure S115: Reverse-phase UPLC of **ON39** after HPLC purification (UV absorbance at 260 nm vs time in min).

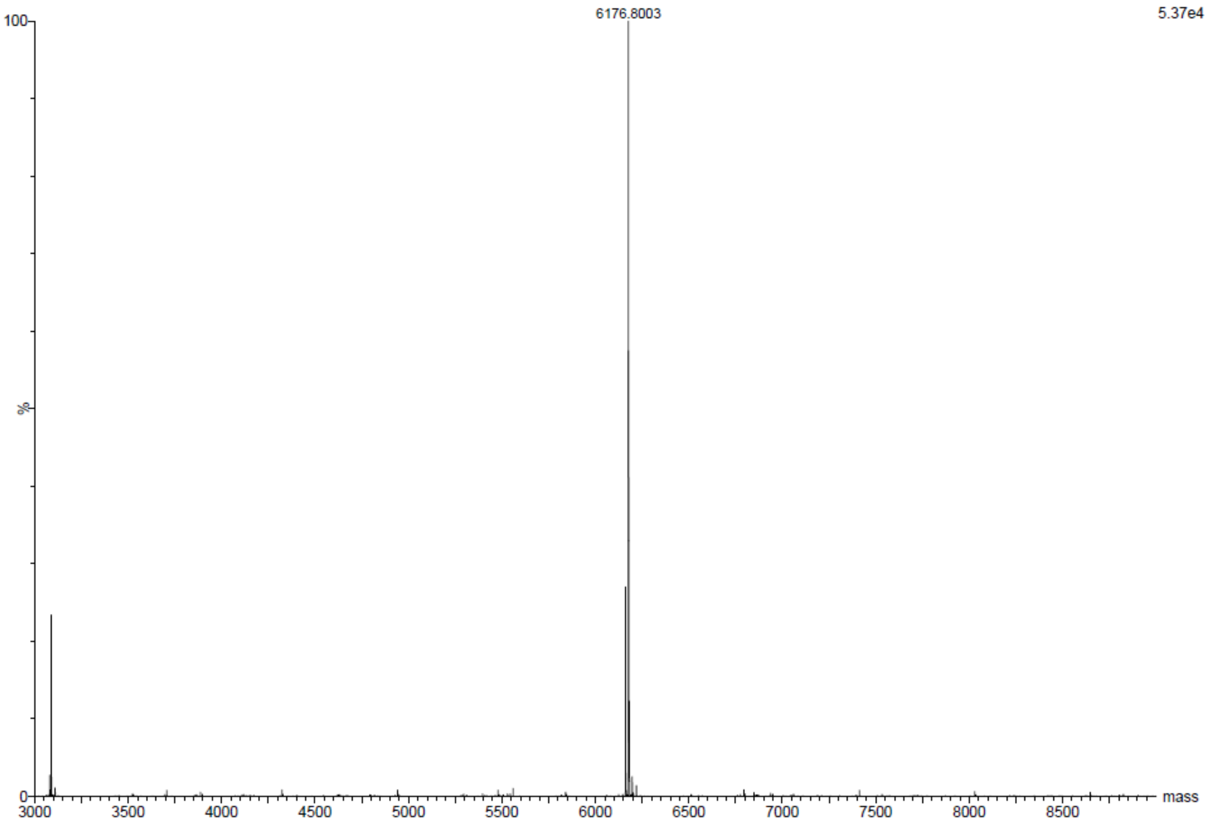

Supplementary Figure S116: Mass spectrum (ES-) of **ON39** after HPLC purification. Required **6176.99** Da, found **6176.80** Da. y-axis = relative intensity (%), x-axis = mass in Da.

ON40 Hexynyl	CCU CUU ACC UCA GUU ACA	**2.Hexyn**	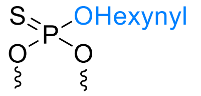

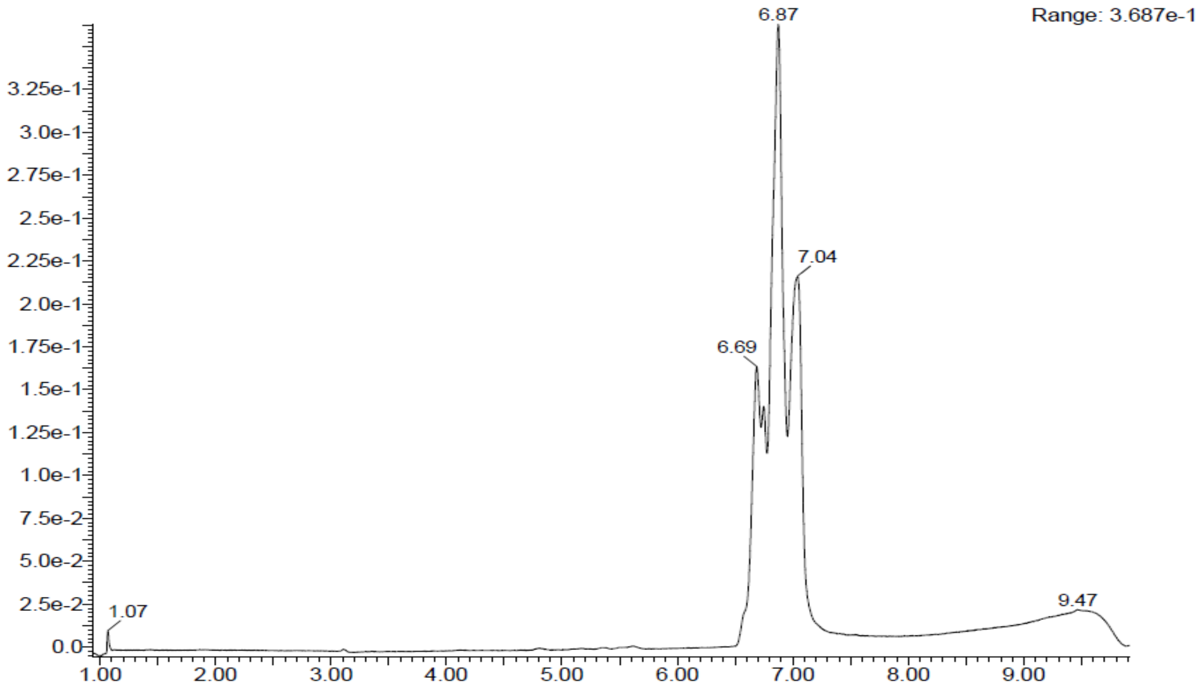

Supplementary Figure S117: Reverse-phase UPLC of **DMTON ON40** (UV absorbance at 260 nm vs time in min).

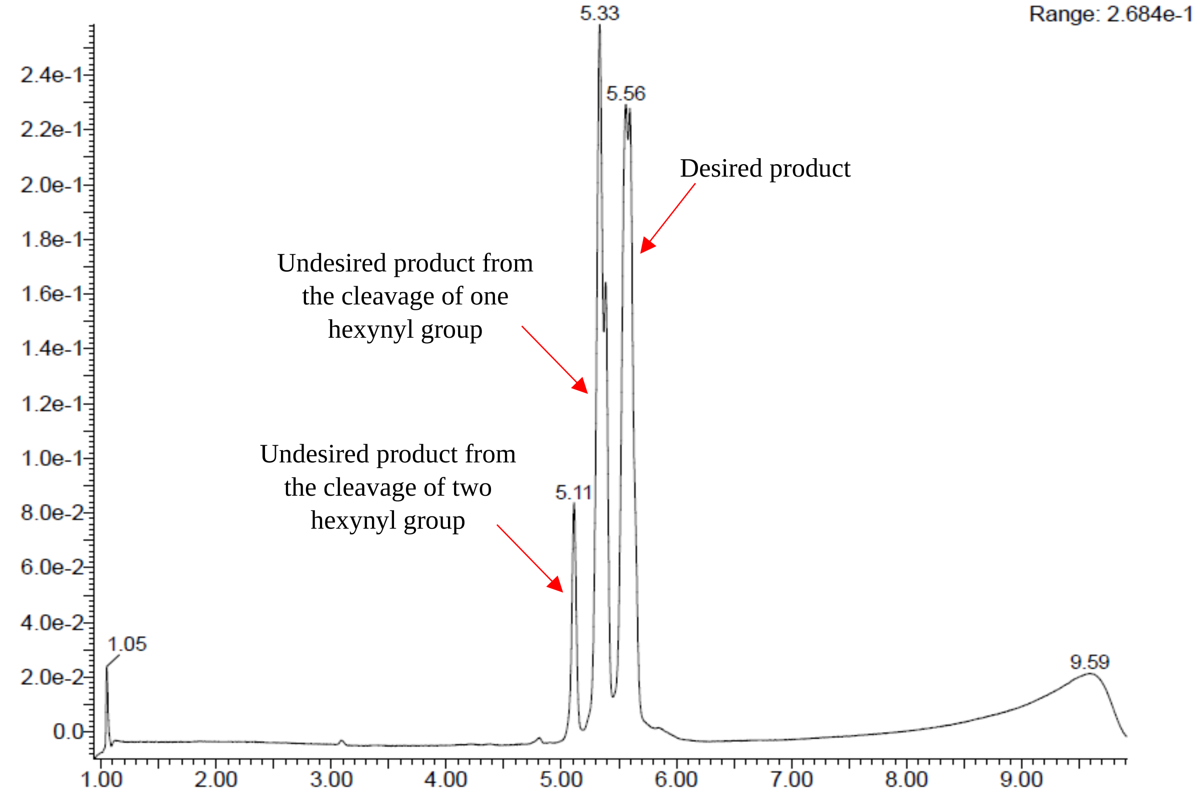

Supplementary Figure S118: Reverse-phase UPLC of crude **ON40** after removal of DMT group (UV absorbance at 260 nm vs time in min).

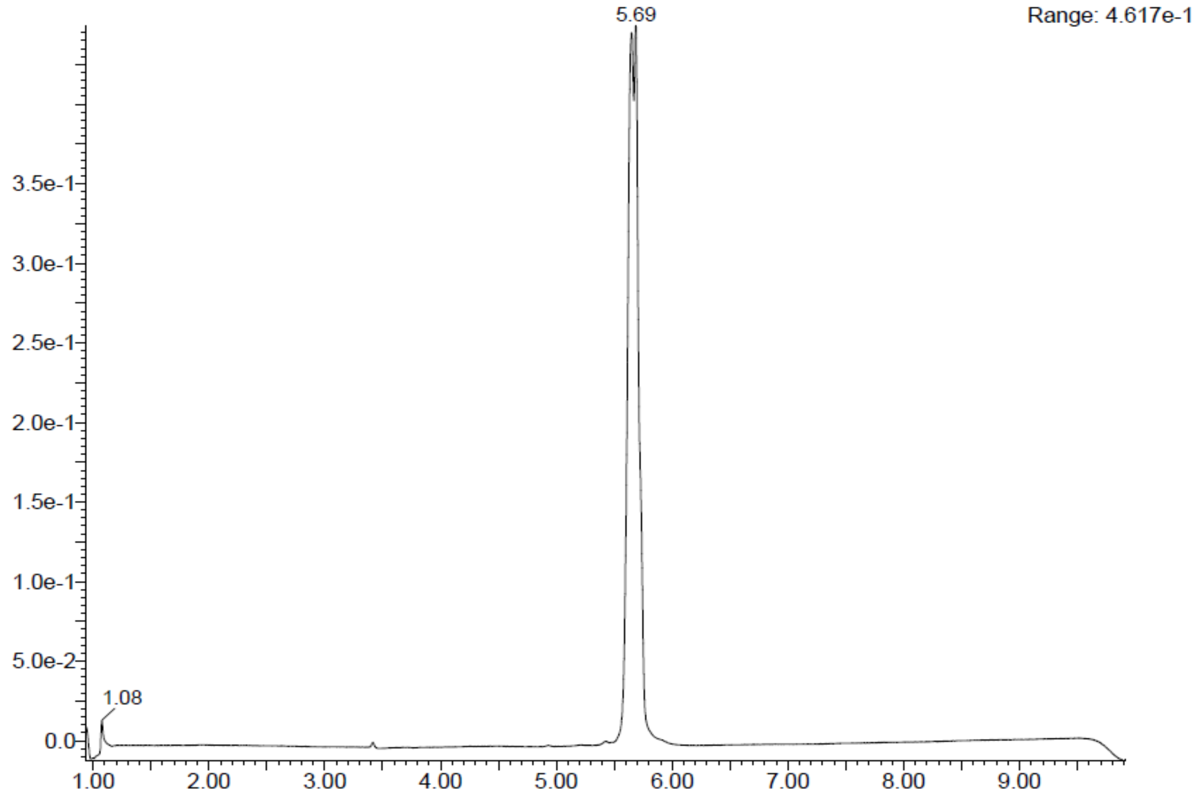

Supplementary Figure S119: Reverse-phase UPLC of **ON40** after HPLC purification (UV absorbance at 260 nm vs time in min).

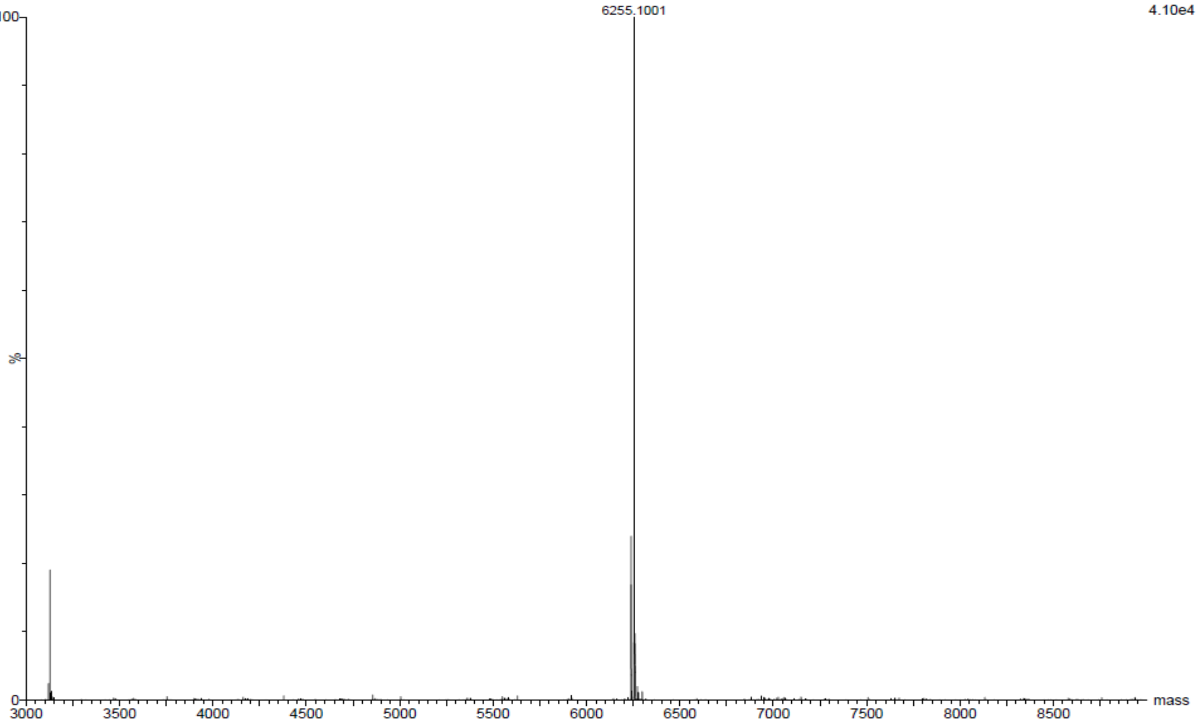

Supplementary Figure S120: Mass spectrum (ES-) of **ON40**. Required **6255.10** Da, found **6255.10** Da. y-axis = relative intensity (%), x-axis = mass in Da.

ON41 Hexynyl	CCU CUU ACC UCA GUU ACA	**3.Hexyn**	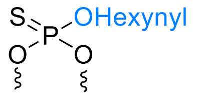

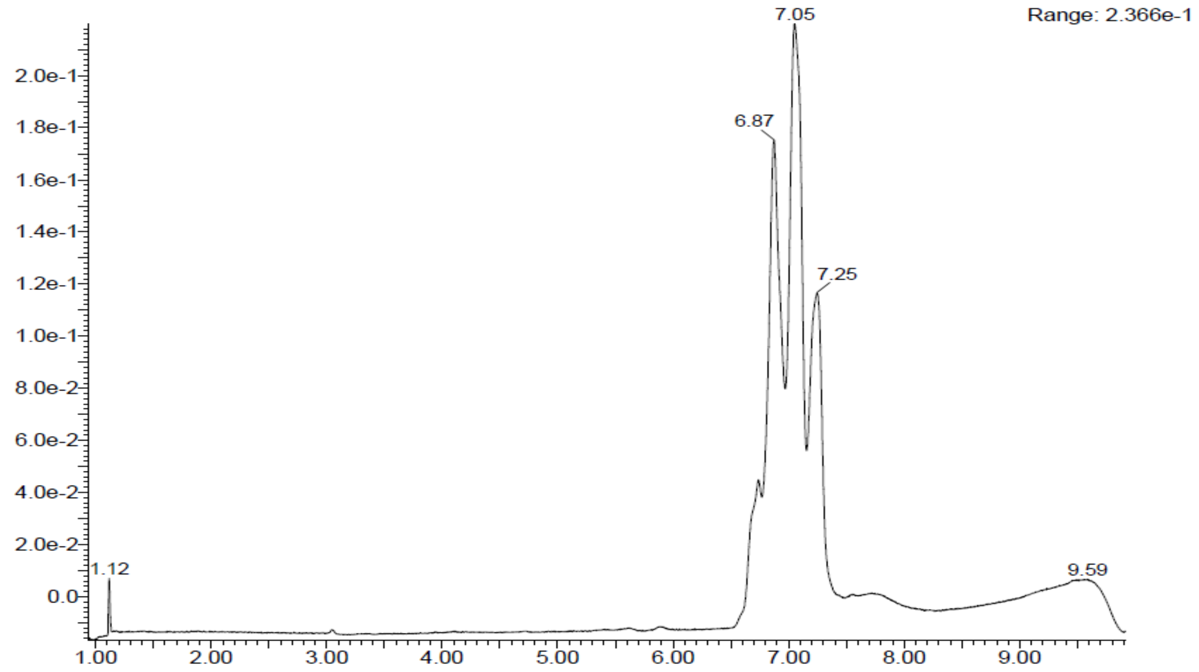

Supplementary Figure S121: Reverse-phase UPLC of **DMTON ON41** (UV absorbance at 260 nm vs time in min).

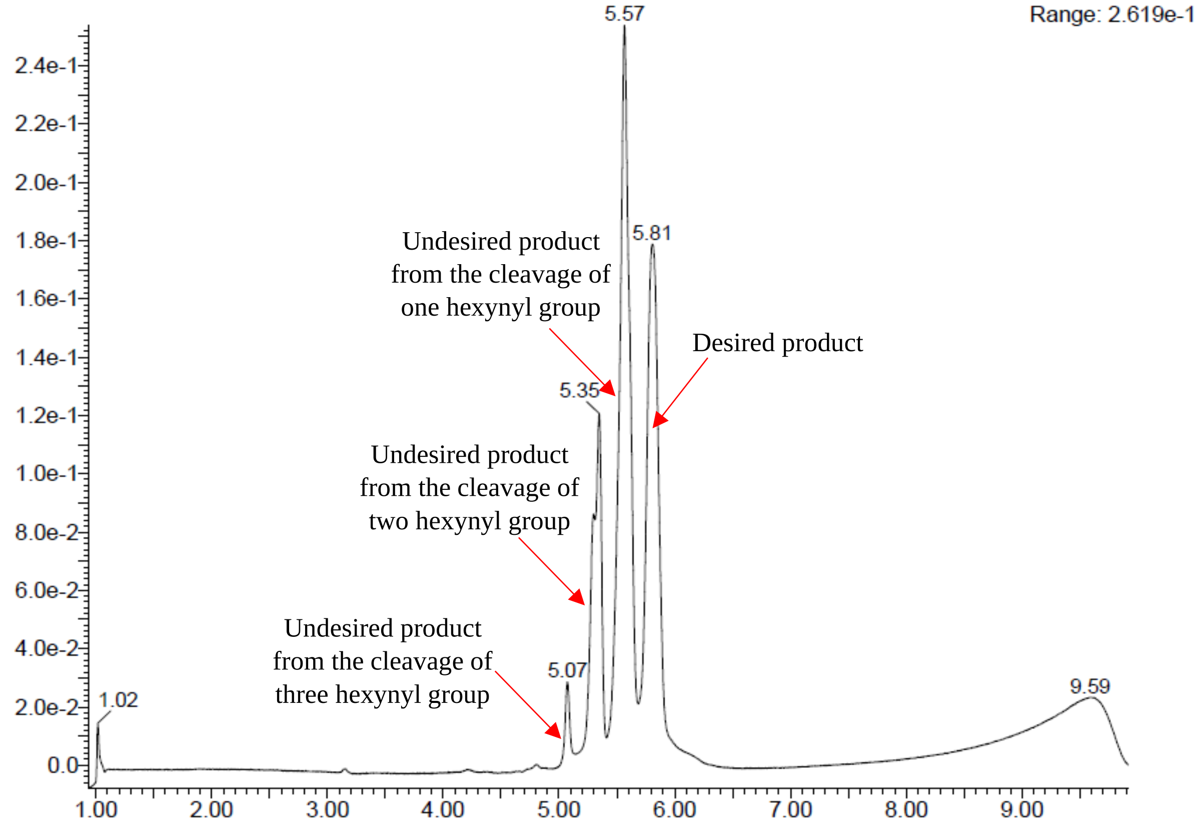

Supplementary Figure S122: Reverse-phase UPLC of crude **ON41** after removal of DMT group (UV absorbance at 260 nm vs time in min).

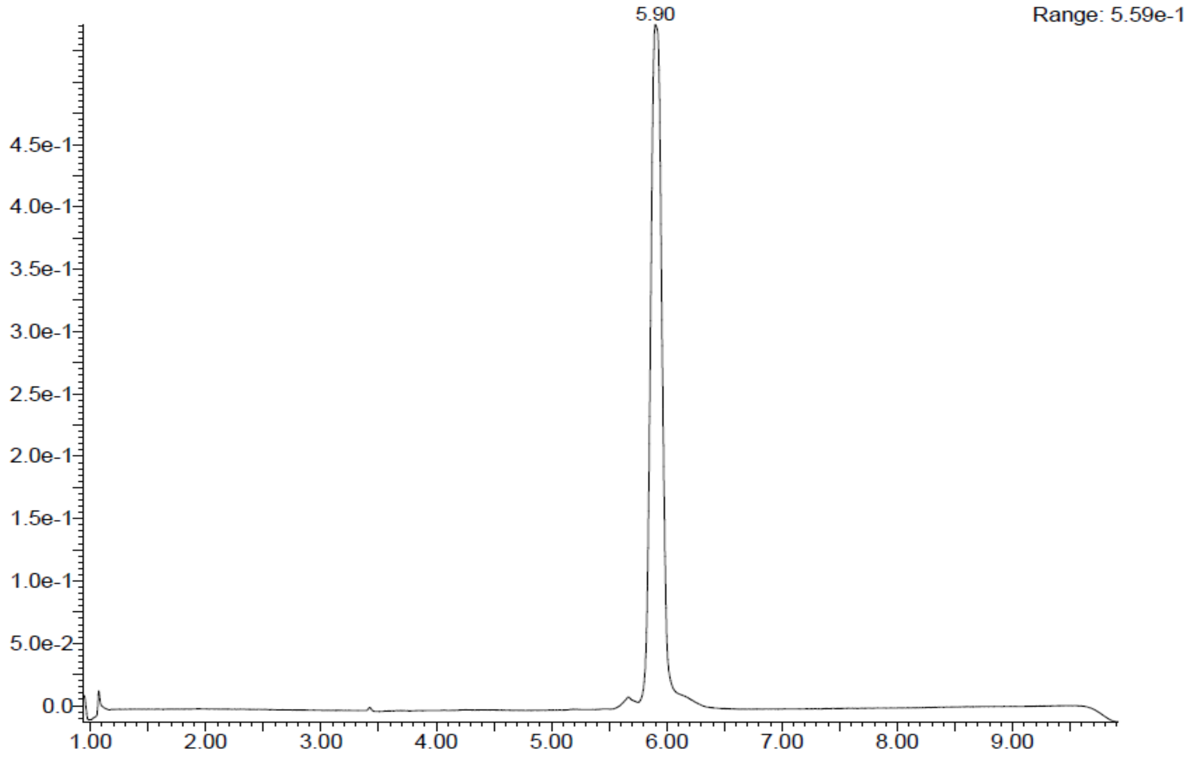

Supplementary Figure S123: Reverse-phase UPLC **ON41** after HPLC purification (UV absorbance at 260 nm vs time in min).

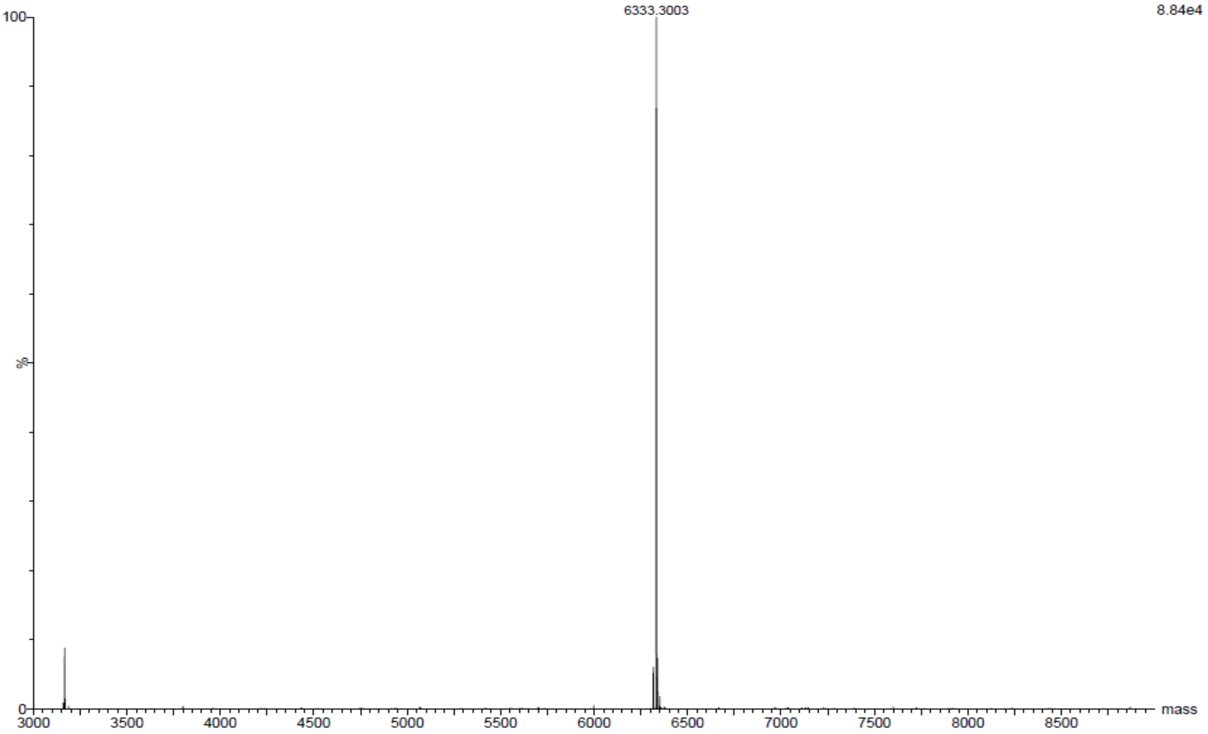

Supplementary Figure S124: Mass spectrum (ES-) of **ON41**. Required **6333.22** Da, found **6332.30** Da. y-axis = relative intensity (%), x-axis = mass in Da.

2.7 Phosphotriester (PTE) and phosphothiotriester (PTTE)

Supplementary Table T5: Deprotection of oligonucleotide (ONS5 and ONS6) containing PTE linkages.

Nucleotides in black have 2´-OMe ribose sugars and phosphorothioate internucleoside linkages. Nucleotides in red are locked nucleic acid isopropyl phosphotriester linkages.

Oligonucleotides	Sequence (5´→3´)	Deprotection condition	Alkyl group	Structure
ONOX1 iPr	CCUCUUACCUCAGUT$_o$ACA	EDA-THF, 2h, rt or aqueous NH_3, 55 °C, 5h	^{i}Pr	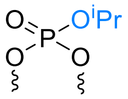
ONOX4 iPr	CCT$_o$CUT$_o$ACCT$_o$CAGUT$_o$ACA		^{i}Pr	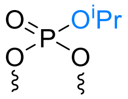

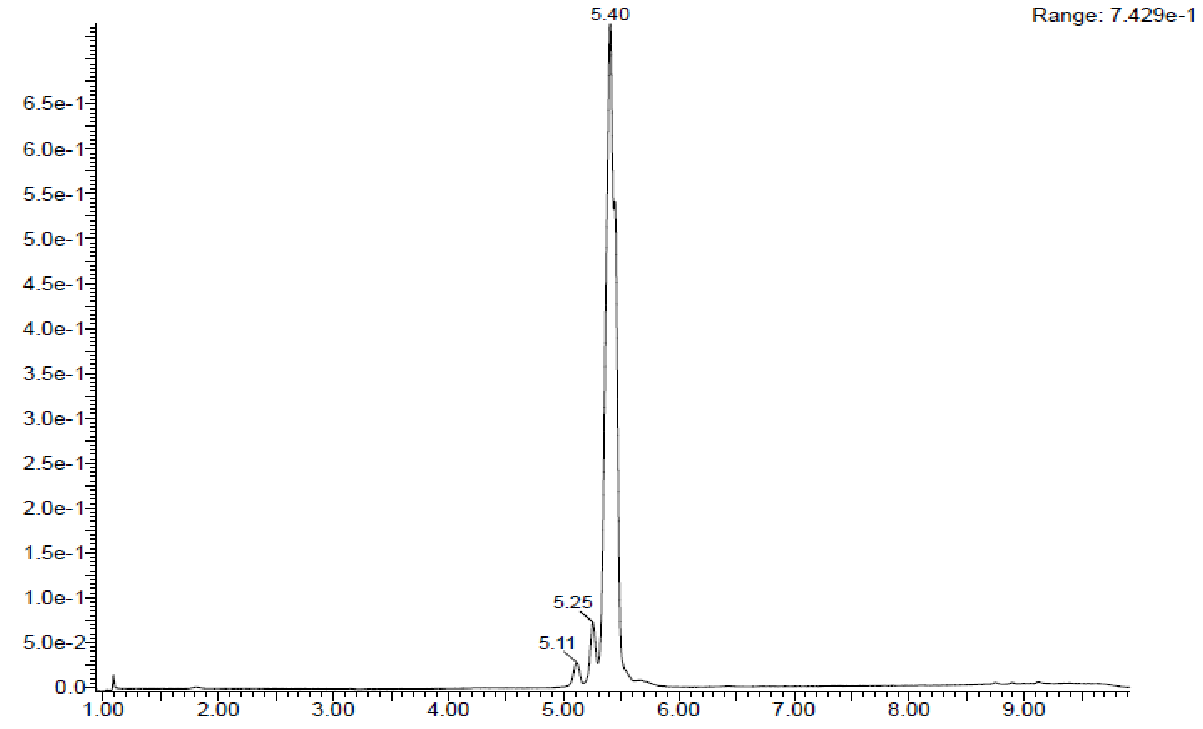

Supplementary Figure S125: Reverse-phase UPLC of **ONOX1** after HPLC purification (UV absorbance at 260 nm vs time in min).

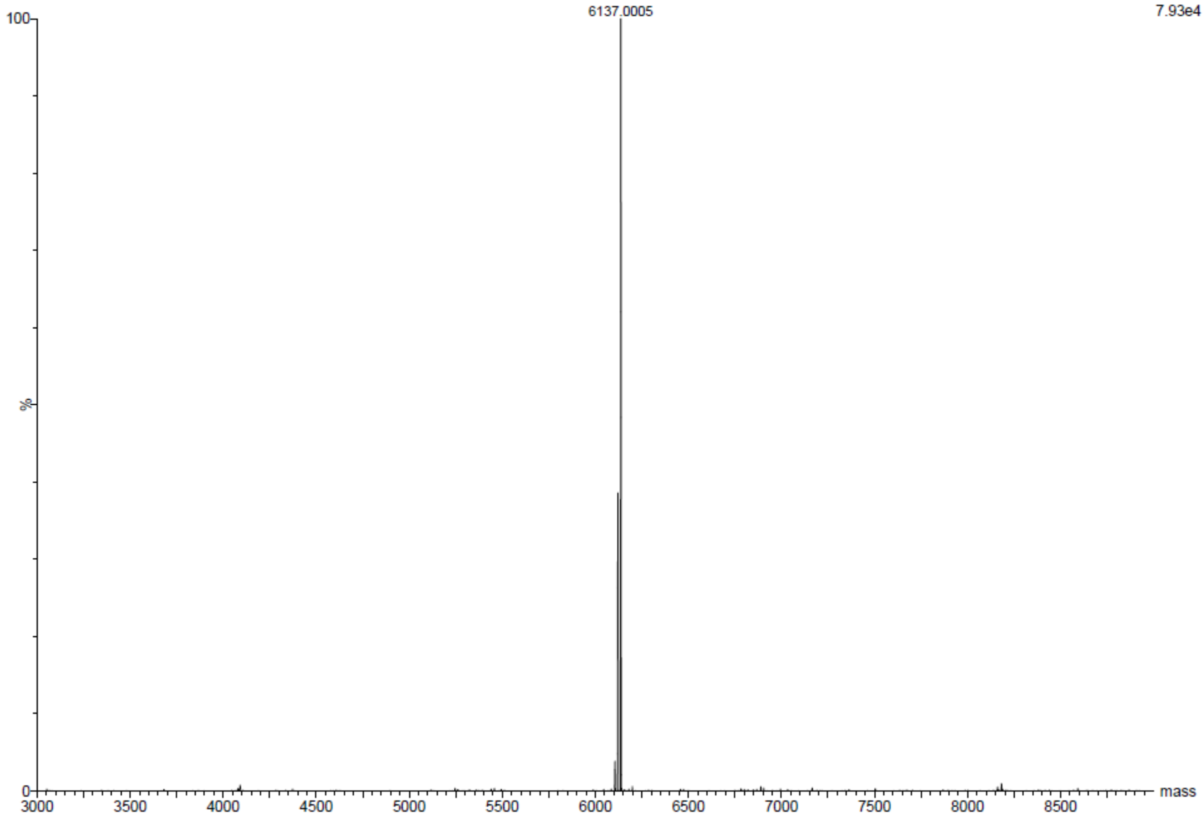

Supplementary Figure S126: Mass spectrum (ES-) of **ONOX1**. Required **6136.91** Da, found **6137.00** Da. y-axis = relative intensity (%), x-axis = mass in Da.

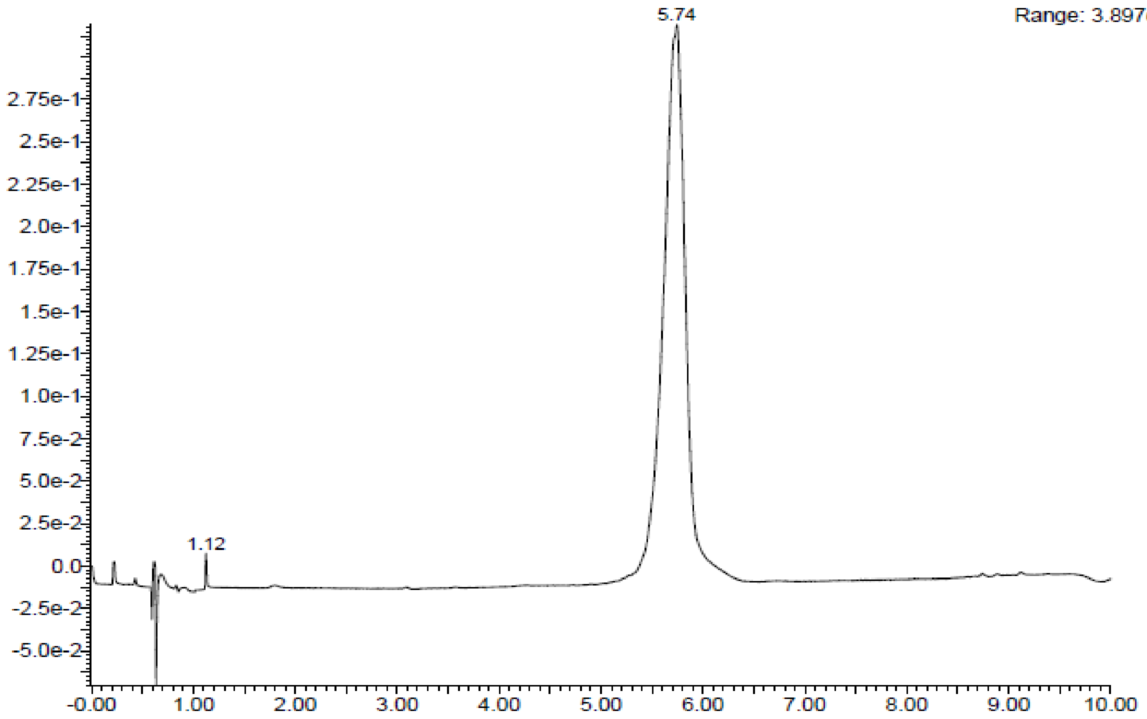

Supplementary Figure S127: Reverse-phase UPLC **ONOX4** after HPLC purification (UV absorbance at 260 nm vs time in min)

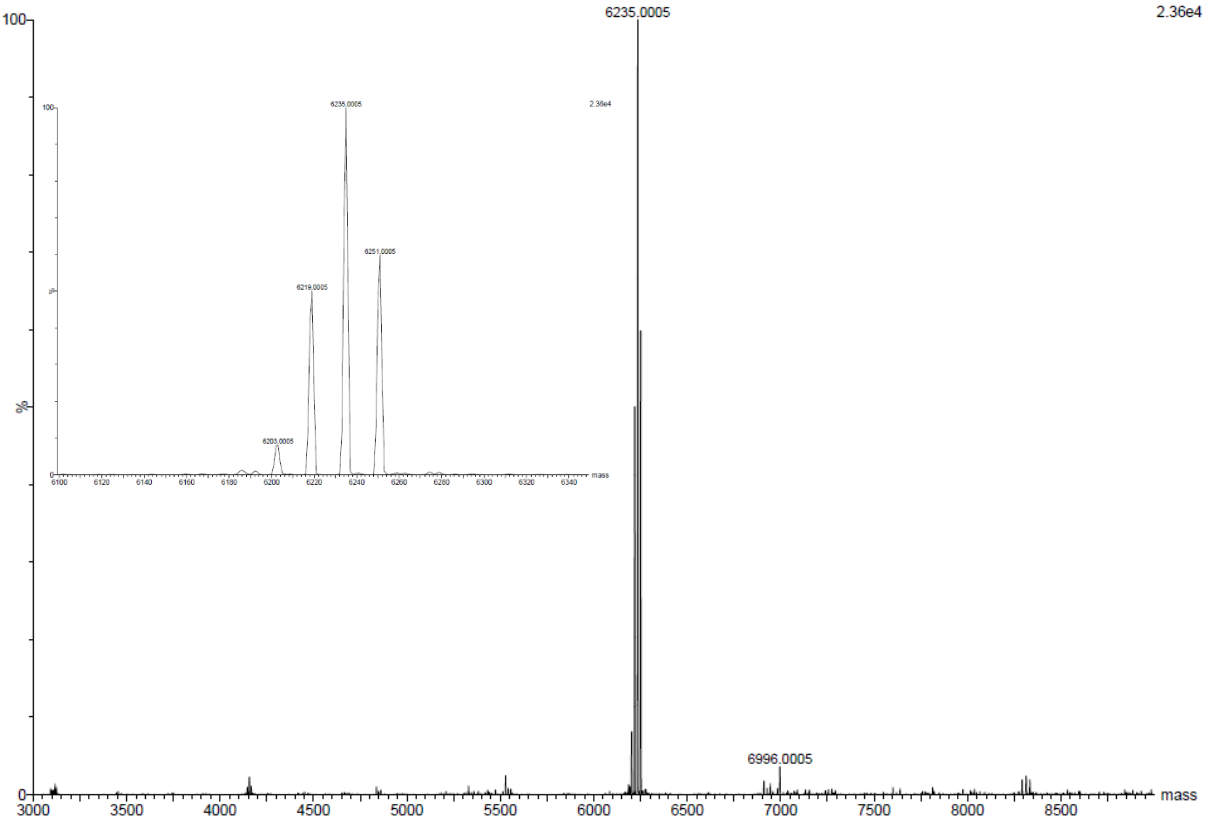

Supplementary Figure S128: Mass spectrum (ES-) of **ONOX4**. Required **6251.00** Da, found **6251.00** Da. y-axis = relative intensity (%), x-axis = mass in Da.

2.8 Complementary DNA and RNA and control oligonucleotide

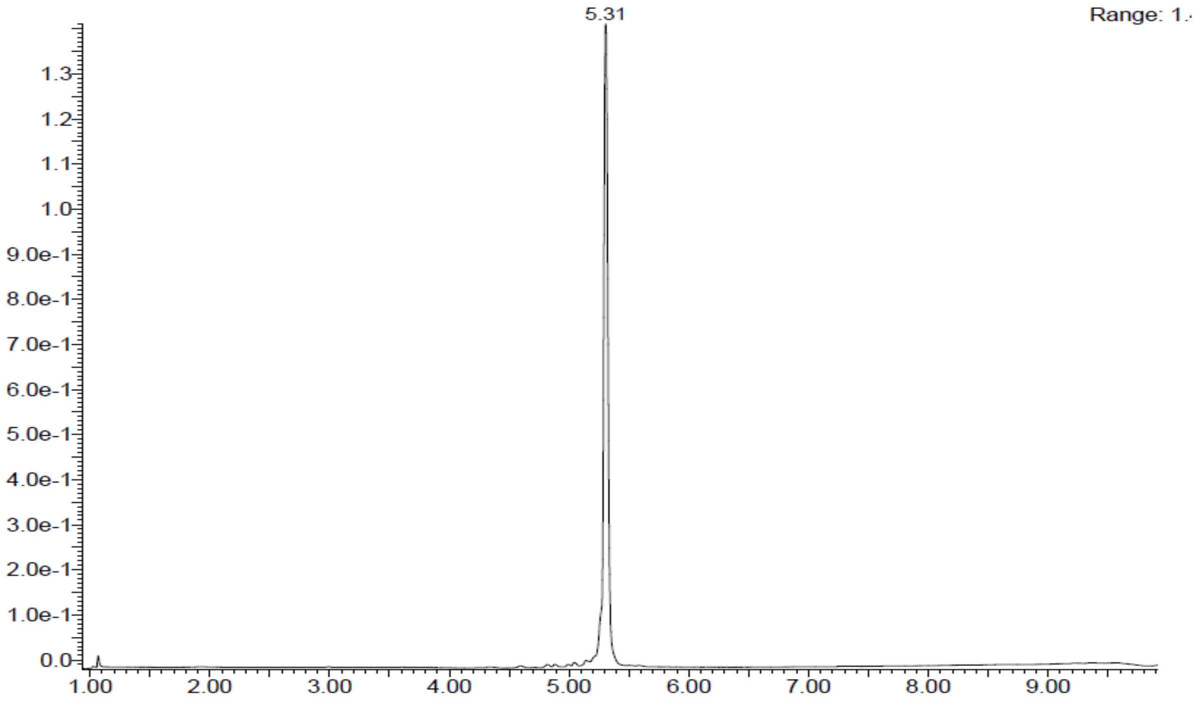

Supplementary Figure S129: Reverse-phase UPLC target DNA **ON42** after HPLC purification (UV absorbance at 260 nm vs time in min)

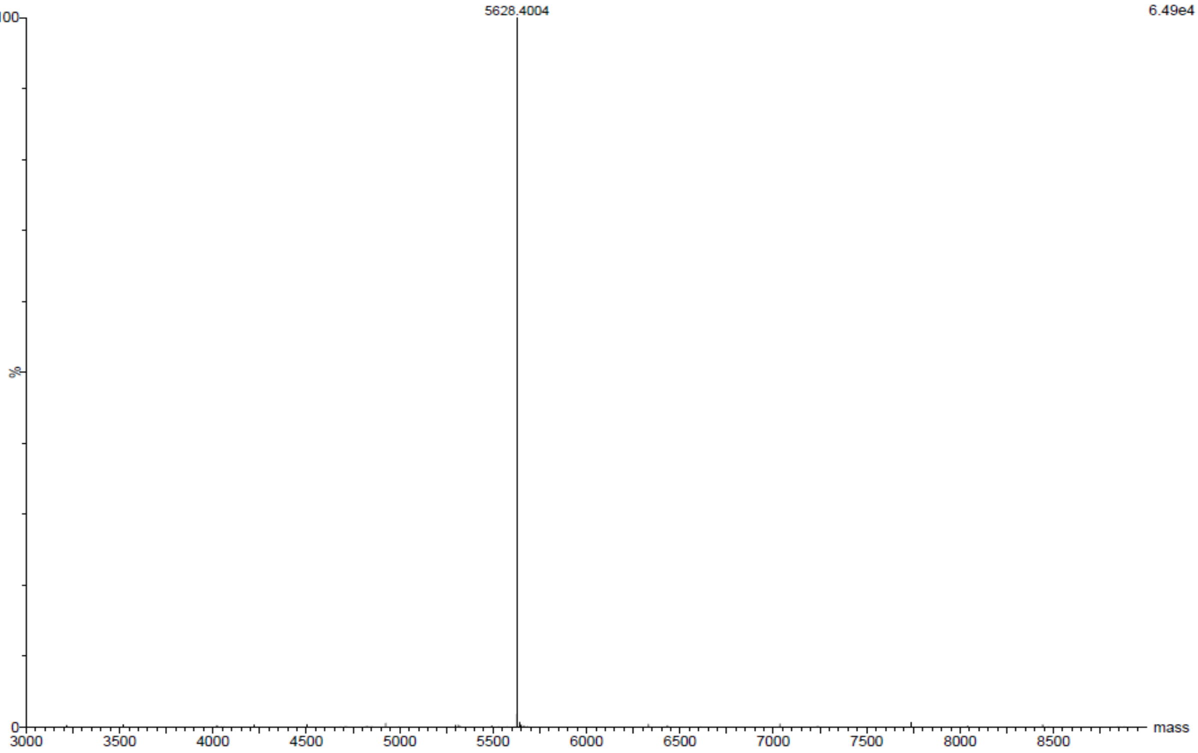

Supplementary Figure S130: Mass spectrum (ES-) of target DNA **ON42**. Required **5627.71** Da, found **5628.40** Da. y-axis = relative intensity (%), x-axis = mass in Da.

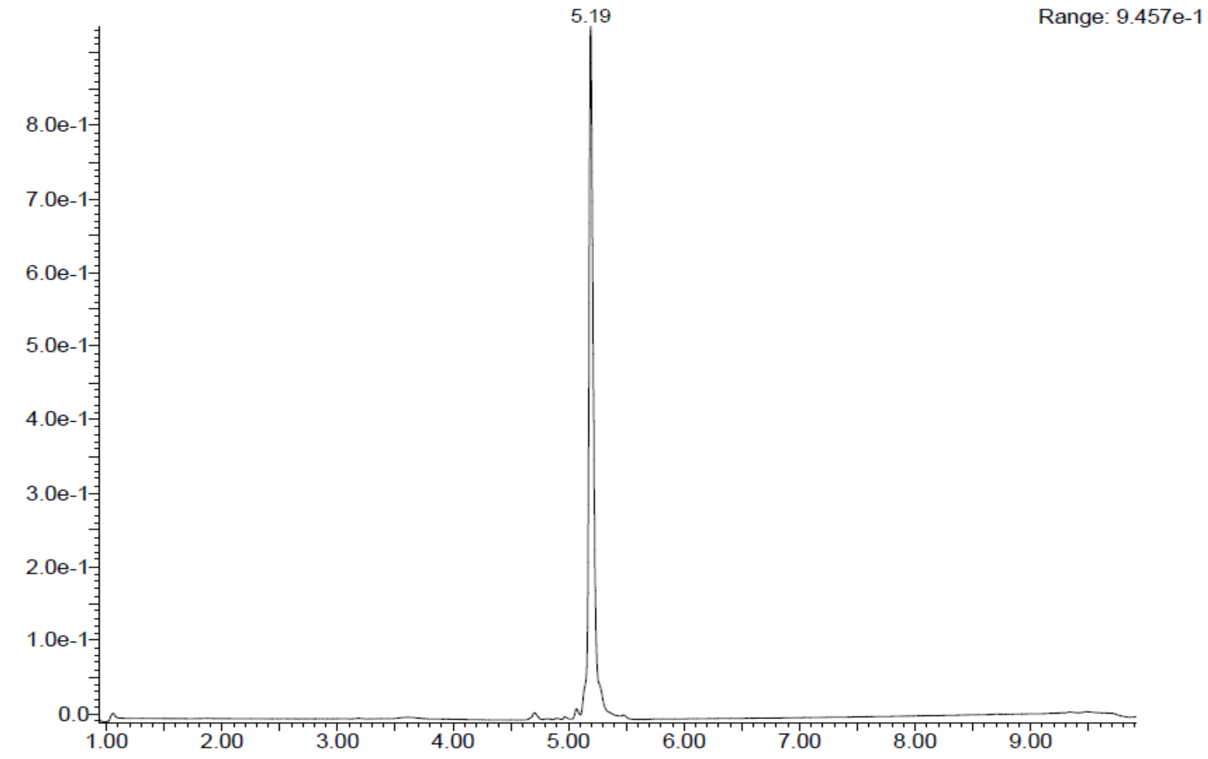

Supplementary Figure S131: Reverse-phase UPLC target RNA **ON43** after HPLC purification (UV absorbance at 260 nm vs time in min)

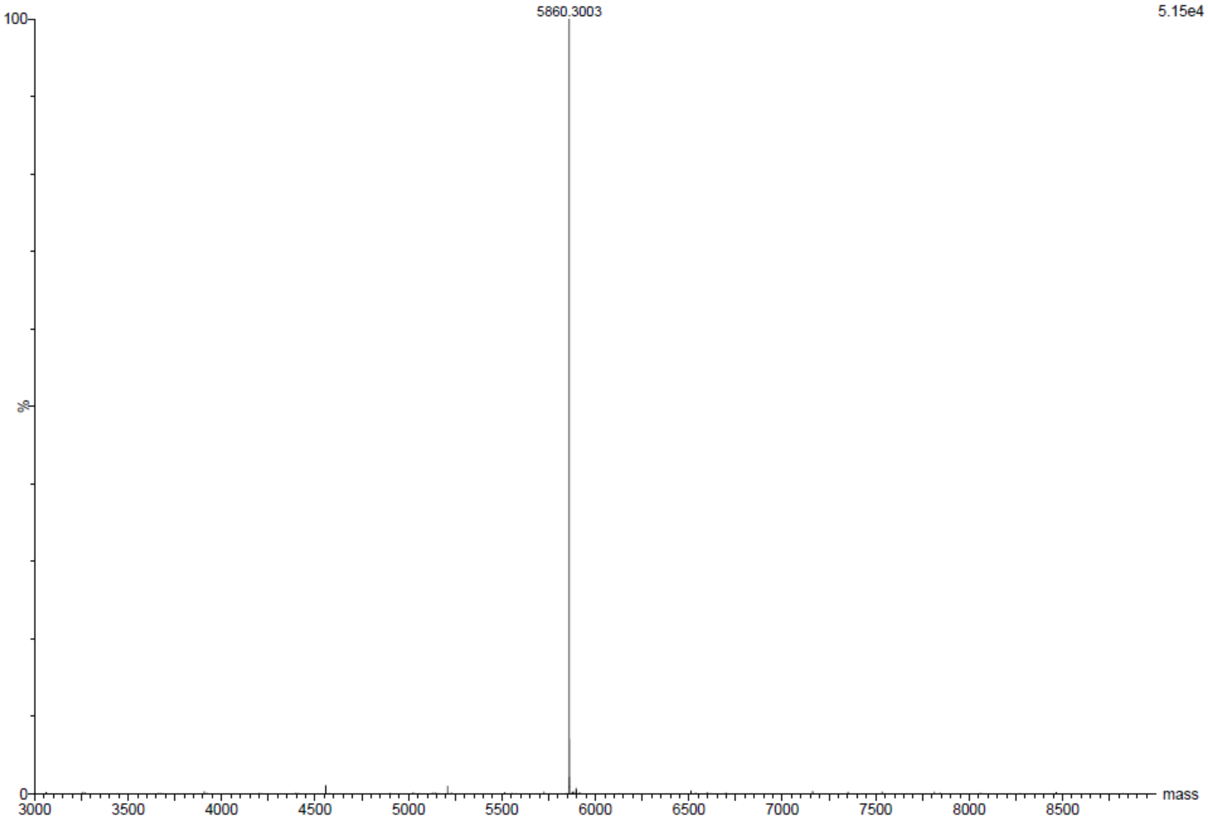

Supplementary Figure S132: Mass spectrum (ES-) of target RNA **ON43**. Required **5859.59** Da, found **5860.30** Da. y-axis = relative intensity (%), x-axis = mass in Da.

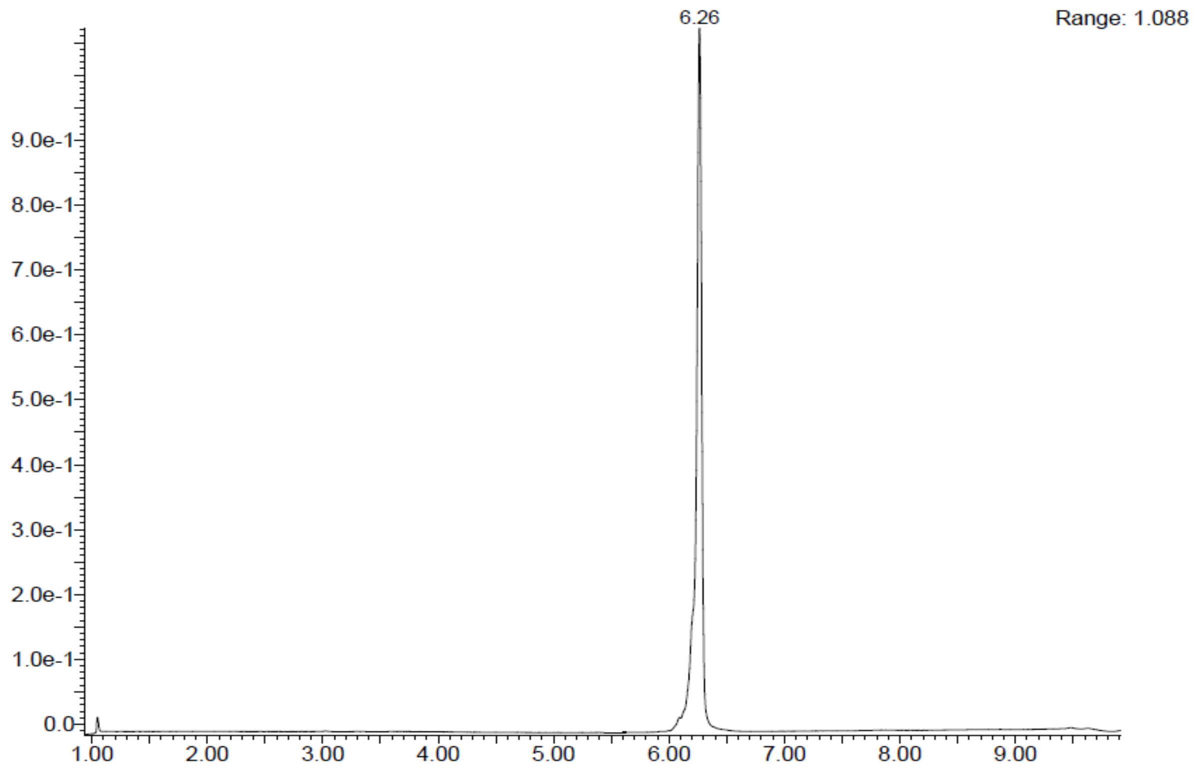

Supplementary Figure S133: Reverse-phase UPLC of control 2´-OMe-PS oligonucleotide **ON44** (UV absorbance at 260 nm vs time in min).

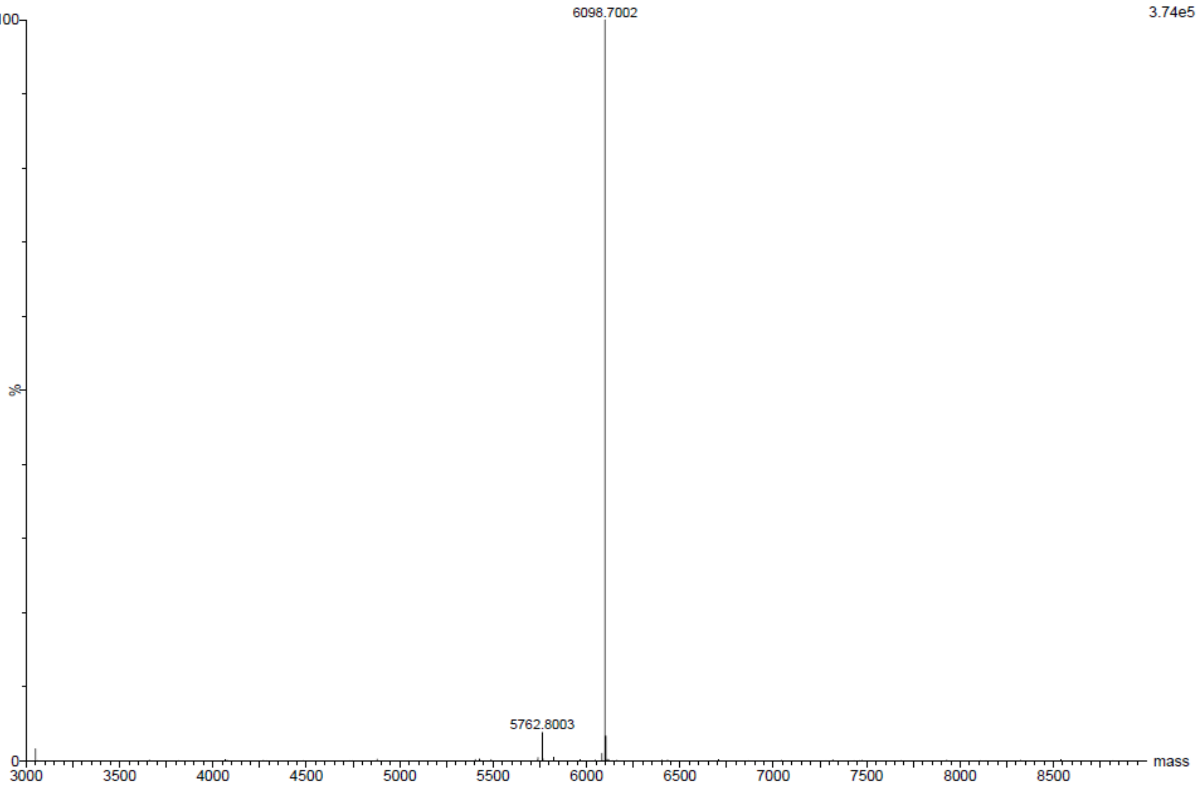

Supplementary Figure S134: Mass spectrum (ES-) of Control 2´-OMe-PS oligonucleotide **ON44**. Required **6098.88** Da, found **6098.70** Da. y-axis = relative intensity (%), x-axis = mass in Da.

3.0 Click reaction and UPLC-MS of oligonucleotide conjugates

Protocol for click reaction for the synthesis of ON46-ON48: A solution of modified oligonucleotide **ON39-ON41** (5 nM, 1 equiv.) in TEAA buffer (2 M, pH = 7.0; 20 µL), and stock of azide **25** (100 nmol in water, 20 equiv.) and sodium ascorbate (250 nM in water, 50 equiv.) were vortexed together. The mixture was degassed by bubbling argon gas for 5 min followed by addition of Cu (II): THPTA (tris(3-hydroxypropyltriazolylmethyl)amine) complex (50 nM in 55%/45% DMSO/H_2O, 10.0 equiv.). Reaction was shaken for 3-24 hours (3h for **ON39**, 12h for **ON40** and 24h for **ON41**) at 25 °C. The mixture was desalted by NAP 10 gel filtration, lyophilised. The crude oligonucleotide was purified by HPLC to obtain the desired oligonucleotide. The oligonucleotide was analysed by UPLC-MS analysis.

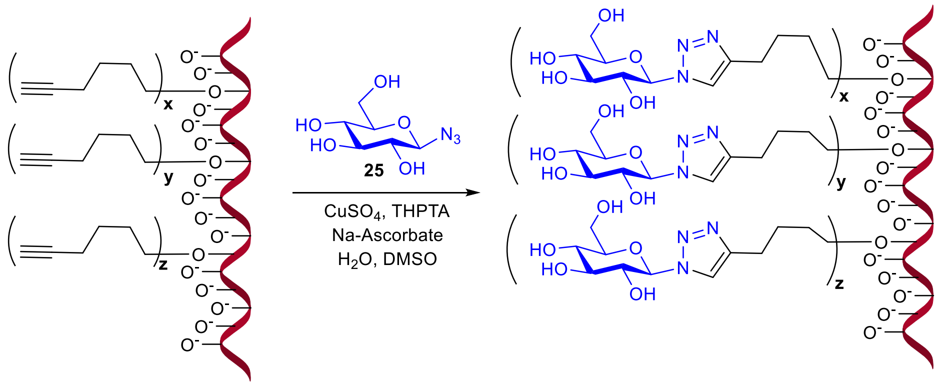

ON39; x = 1, y = 0, z = 0
ON40; x = 0, y = 1, z = 1
ON41; x = 1, y = 1, z = 1

ON47; x = 1, y = 0, ,z = 0
ON48; x = 0, y = 1, z = 1
ON49; x = 1, y = 1, z = 1

ON47 CCU CUU ACC UCA_{Glu} GUU ACA
ON48 CCU CUU A_{Glu}GluCC UCA_{Glu} GUU ACA
ON49 CCU CUUA_{Glu}CC UCA_{Glu} GUU A_{Glu}CA

Supplementary Figure S135: Synthesis of **ON47-ON49** by site-specific CuAAC multiple post-labelling of 18-mer oligonucleotide **ON39-ON41** with glucose ligands (THPTA = tris(3-hydroxypropyltriazolylmethyl)amine click catalyst).

**ON47**	CCU CUU ACC UCA$_{Glu}$ GUU ACA	**Hexynyl-GluN$_3$**	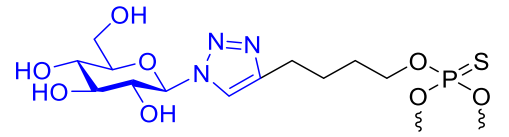

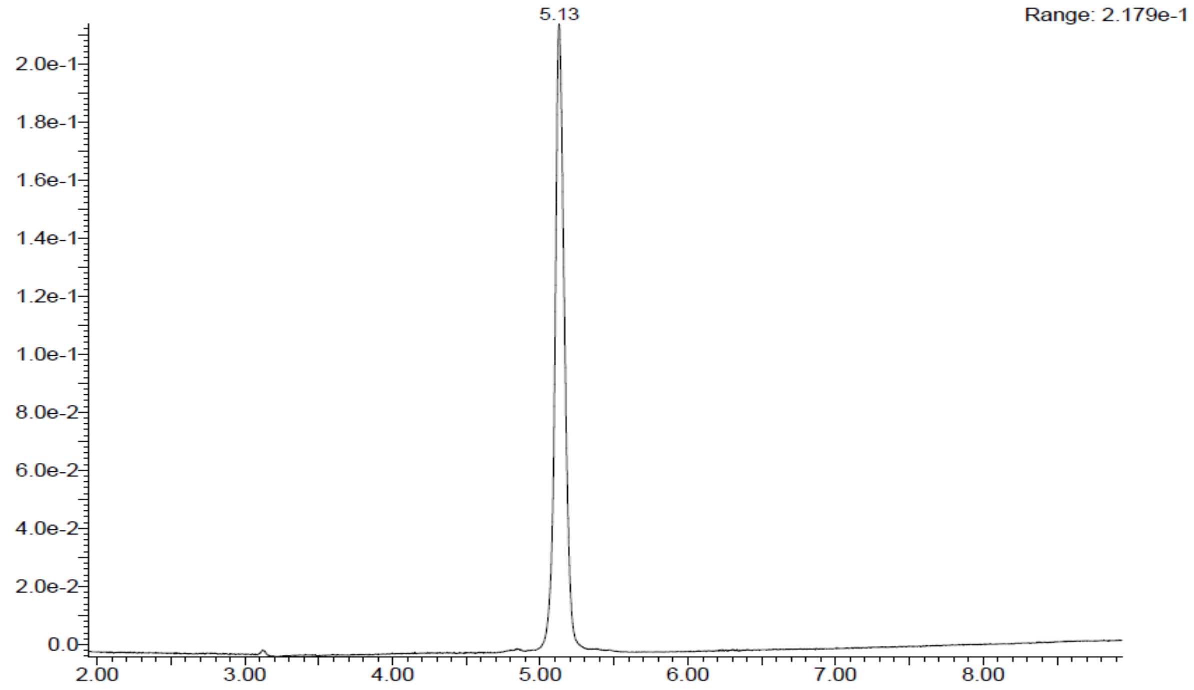

Supplementary Figure S136: Reverse-phase UPLC **ON47** (UV absorbance at 260 nm vs time in min)

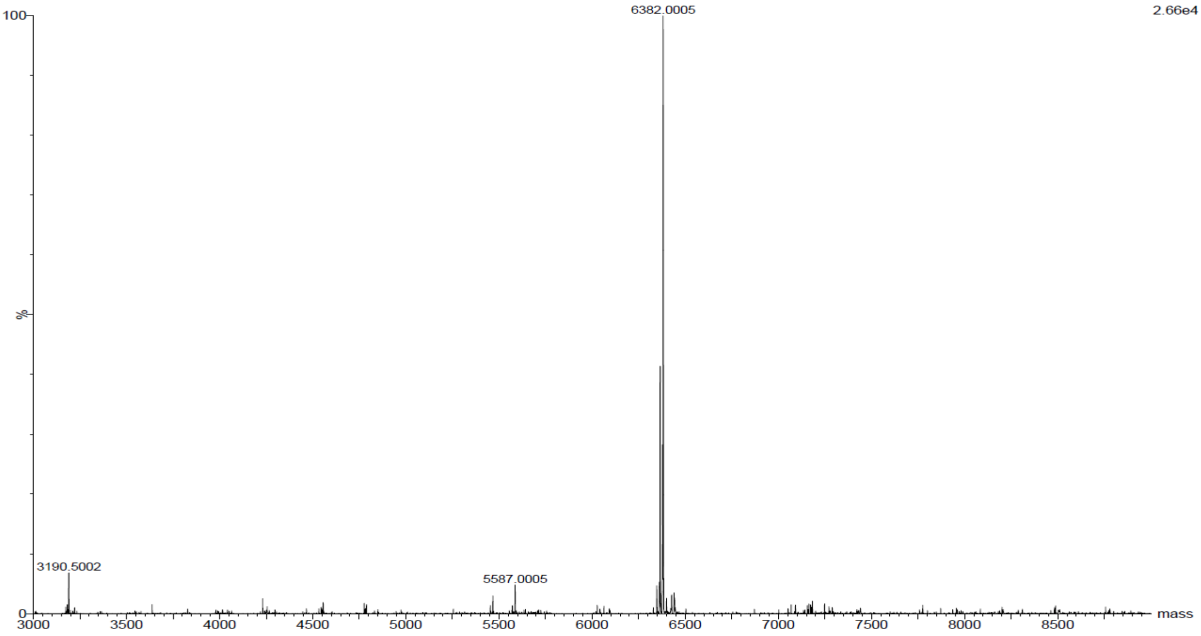

Supplementary Figure S137: Mass spectrum (ES-) of **ON47**. Required **6382.16** Da, found **6382.00** Da. y-axis = relative intensity (%), x-axis = mass in Da.

**ON48**	CCU CUU A$_{Glu}$CC UCA GUU A$_{Glu}$CA	**2.Hexyn-GluN$_3$**	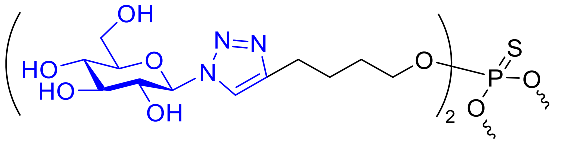

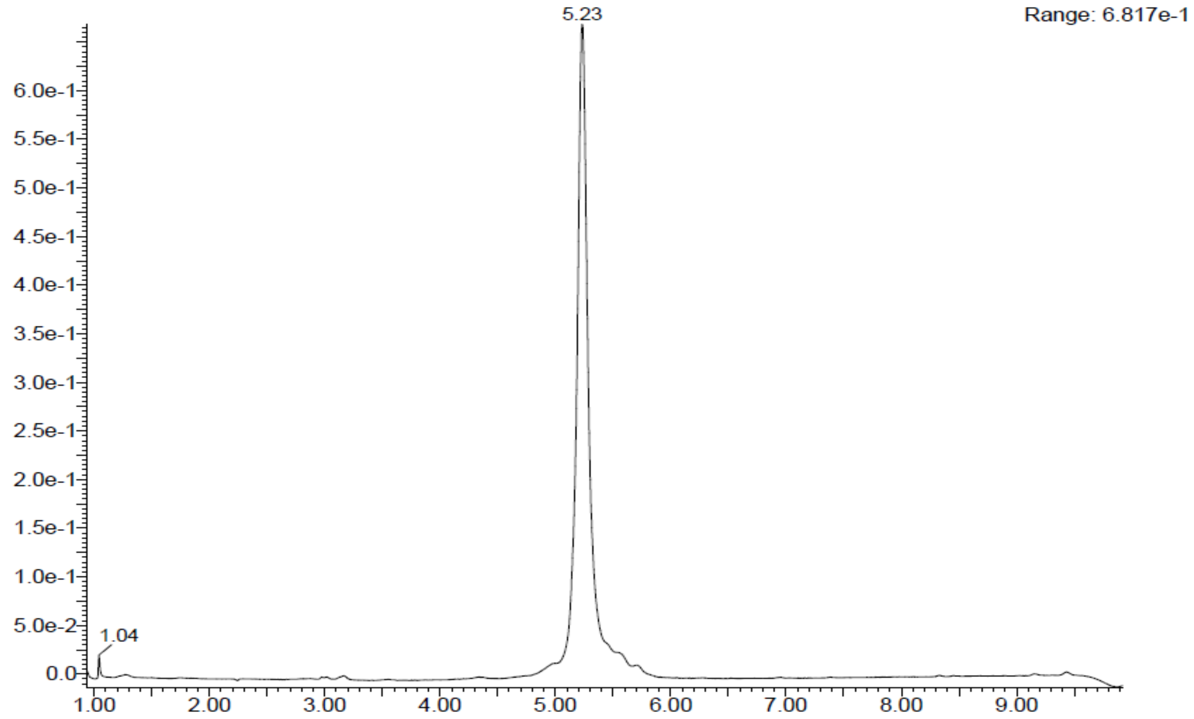

Supplementary Figure S138: Reverse-phase UPLC **ON48** (UV absorbance at 260 nm vs time in min)

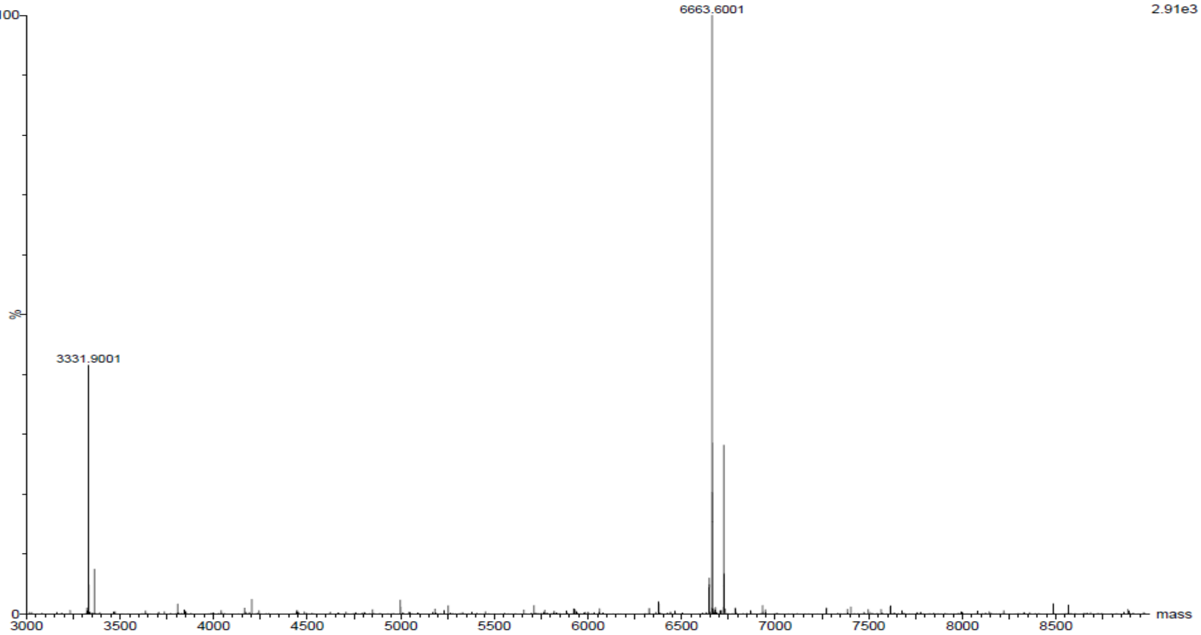

Supplementary Figure S139: Mass spectrum (ES-) of **ON48**. Required **6665.45** Da, found **6663.60** Da. y-axis = relative intensity (%), x-axis = mass in Da.

**ON49**	CCU CUU AGluCC UCAGlu GUU AGluCA	**3.Hexyn-GluN3**	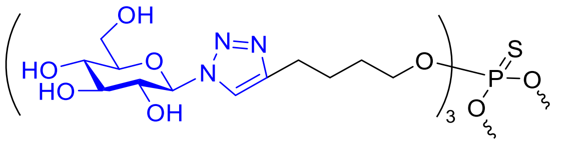

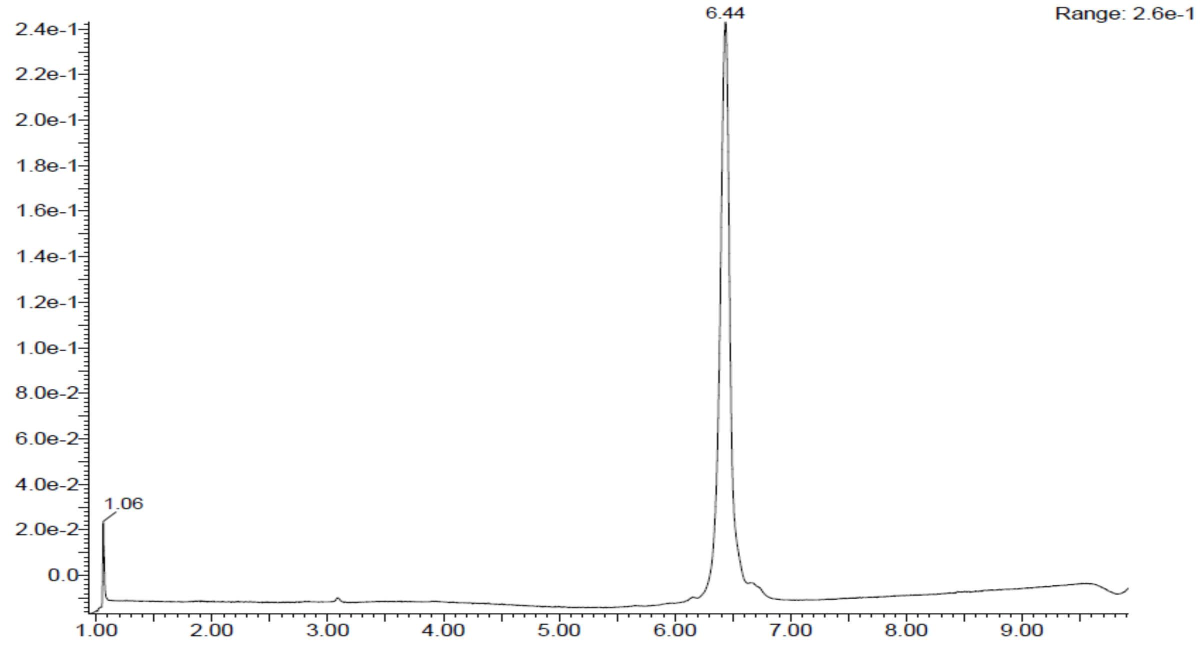

Supplementary Figure S140: Reverse-phase UPLC **ON49** (UV absorbance at 260 nm vs time in min)

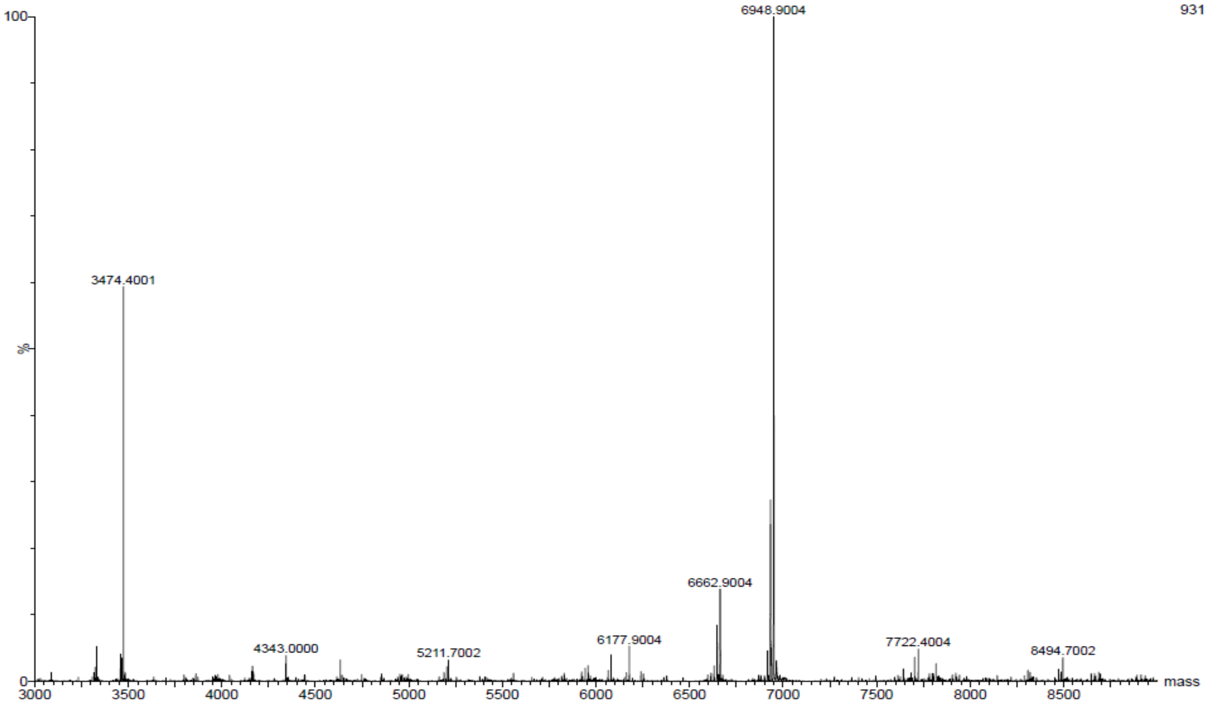

Supplementary Figure S141: Mass spectrum (ES-) of **ON49**. Required **6948.73** Da, found **6948.90** Da. y-axis = relative intensity (%), x-axis = mass in Da.

4.0 UV meting experiments

UV melting experiments were performed using a Cary 4000 scan UV-vis spectrophotometer. Unless mentioned elsewhere 2 nM of each oligonucleotide was dissolved in 1 mL of 10 mM phosphate buffer containing 100 mM NaCl (for DNA Target) and 25 mM NaCl (for RNA target) at pH 7.0. The samples were first denatured by heating to 85 °C (10 °C/min) and then annealed by slowly cooling to 20 °C. (1 °C/min). Six successive cycles of heating and cooling were performed at a gradient of 1 °C/min whilst recording the change in UV absorbance at 260 nm. The built-in Cary WinUV 3.0 software was then used to calculate the melting temperature from the first derivative of the melting curves. The curves shown are representative of three independent repeats, each consisting of at least two technical repeats. Tm values are an average of three experiments with an error of ±0.25 °C.

4.4 MeOPr PTTE oligonucleotides

Supplementary Table T6: Duplex melting temperatures (Tm) in °C of **ON1-ON4** against DNA and RNA. Nucleotides in black have 2´-OMe ribose sugars and phos-phorothioate internucleoside linkages. Nucleotides in red are locked nucleic acid phosphorothioate triesters.

ON	Sequence (5´→3´)	MeOPr group	DNA target (**ON42**)		RNA target (**ON43**)	
			Tm	(ΔTm)	Tm	(ΔTm)
ON1 MeOPr	CCU CUU ACC UCA GUU ACA	1	51.8	+3.3	64.1	+2.8
ON2 MeOPr	CCU CUU ACC UCA GUU ACA	2	53.4	+4.9	64.8	+3.5
ON3 MeOPr	CCU CUU ACC UCA GUU ACA	2	51.9	+3.4	64.1	+2.8
ON4 MeOPr	CCU CUU ACC UCA GUU ACA	3	54.5	+6.0	66.2	+4.9

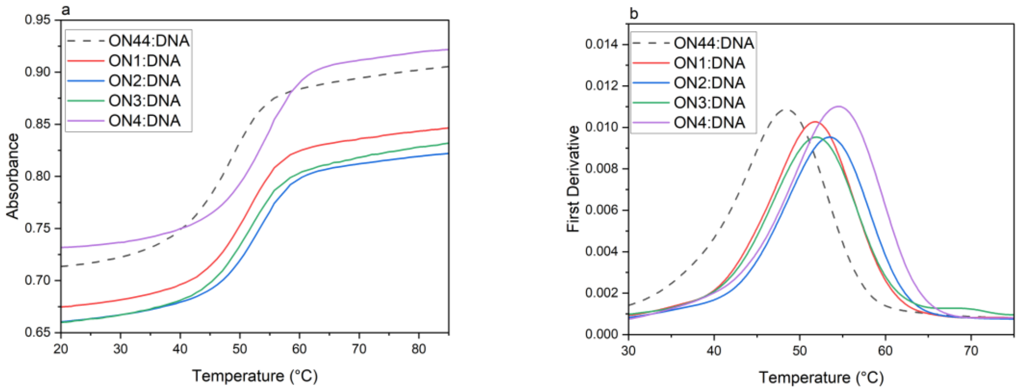

Supplementary Figure S142: UV melting studies for modified ONs **(ON1-ON4)** against complementary DNA (**ON42**). **a.** Representative UV melting curves measured using 2 μM of each ON in 10 mM Na-phosphate buffer, 100 mM NaCl, pH = 7.0; **b.** 1st derivative of melting curves.

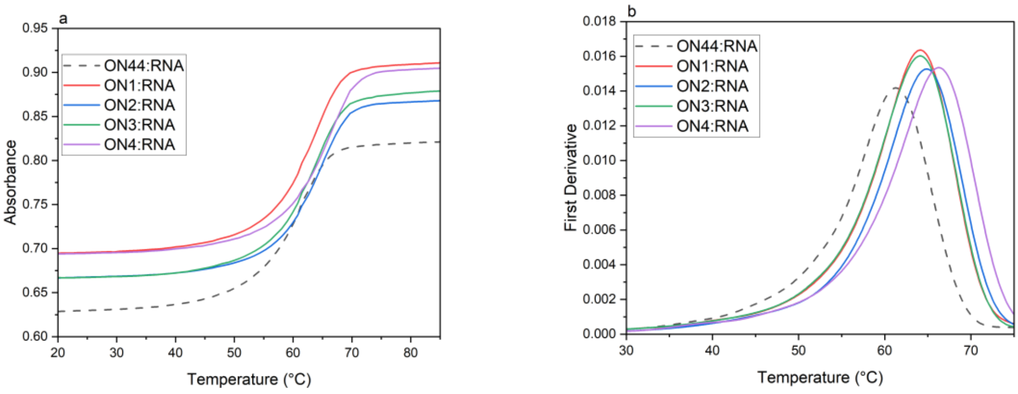

Supplementary Figure S143: UV melting studies for modified ONs **(ON1-ON4)** against complementary RNA (**ON43**). **a.** Representative UV melting curves measured using 2 μM of each ON in 10 mM Na-phosphate buffer, 25 mM NaCl, pH = 7.0; **b.** 1st derivative of melting curves.

Supplementary Table T7: Duplex melting temperatures (Tm) in °C of **ON5-ON8** against DNA and RNA. Nucleotides in black have 2´-OMe ribose sugars and phosphorothioate internucleoside linkages. Nucleotides in red are locked nucleic acid phosphorothioate triester.

**ON**	Sequence (5´→3´)	MeOPr group	DNA target (**ON42**)		RNA target (**ON43**)	
			Tm	(ΔTm)	Tm	(ΔTm)
ON5 MeOPr	CCU CUU ACC UCA GUT ACA	1	51.4	+2.9	64.3	+3.0
ON6 MeOPr	CCU CUT ACC UCA GUT ACA	2	54.4	+5.9	66.8	+5.5
ON7 MeOPr	CCT CUT ACC TCA GUT ACA	4	58.8	+10.3	70.7	+9.4
ON8 MeOPr	CCT CTT ACC TCA GTT ACA	6	64.2	+15.6	>75.0	Nd

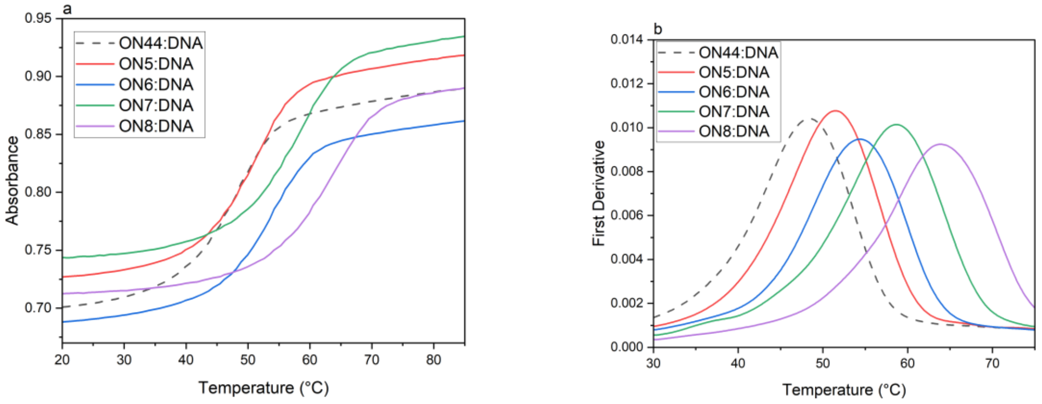

Supplementary Figure S144: UV melting studies for modified ONs **(ON5-ON8)** against complementary DNA (**ON42**). **a.** Representative UV melting curves measured using 2 µM of each ON in 10 mM Na-phosphate buffer, 100 mM NaCl, pH = 7.0; **b.** 1st derivative of melting curves.

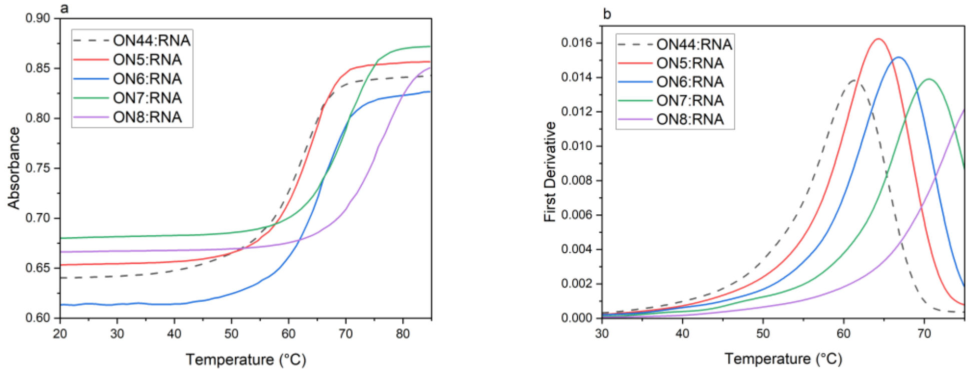

Supplementary Figure S145: UV melting studies for modified ONs **(ON5-ON8)** against complementary RNA (**ON43**). **a.** Representative UV melting curves measured using 2 µM of each ON in 10 mM Na-phosphate buffer, 25 mM NaCl, pH = 7.0; **b.** 1st derivative of melting curves.

4.2 iPr PTTE oligonucleotides

Supplementary Table T8: Duplex melting temperatures (Tm) in °C of **ON9-ON12** against DNA and RNA. Nucleotides in black have 2´-OMe ribose sugars and phosphorothioate internucleoside linkages. Nucleotides in red are locked nucleic acid phosphorothioate triester.

ON	Sequence (5´→3´)	^{i}Pr group	DNA target (**ON42**)		RNA target (**ON43**)	
			Tm	(ΔTm)	Tm	(ΔTm)
ON9 iPr	CCU CUU ACC UCA GUU ACA	1	52.5	+4.0	64.9	+3.6
ON10 iPr	CCU CUU ACC UCA GUU ACA	2	55.2	+6.7	66.5	+5.2
ON11 iPr	CCU CUU ACC UCA GUU ACA	2	53.7	+5.2	65.8	+4.5
ON12 iPr	CCU CUU ACC UCA GUU ACA	3	56.8	+8.3	68.3	+7.0

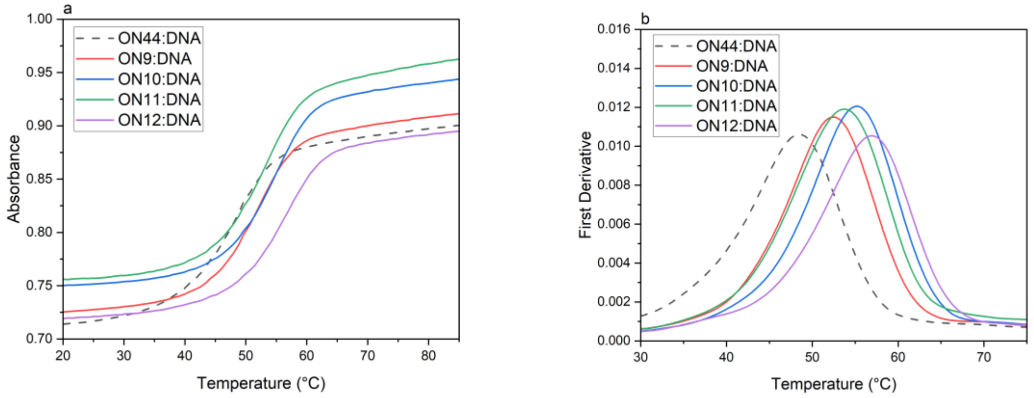

Supplementary Figure S146: UV melting studies for modified ONs **(ON9-ON12)** against complementary DNA (**ON42**). **a.** Representative UV melting curves measured using 2 μM of each ON in 10 mM Na-phosphate buffer, 100 mM NaCl, pH = 7.0; **b.** 1st derivative of melting curves.

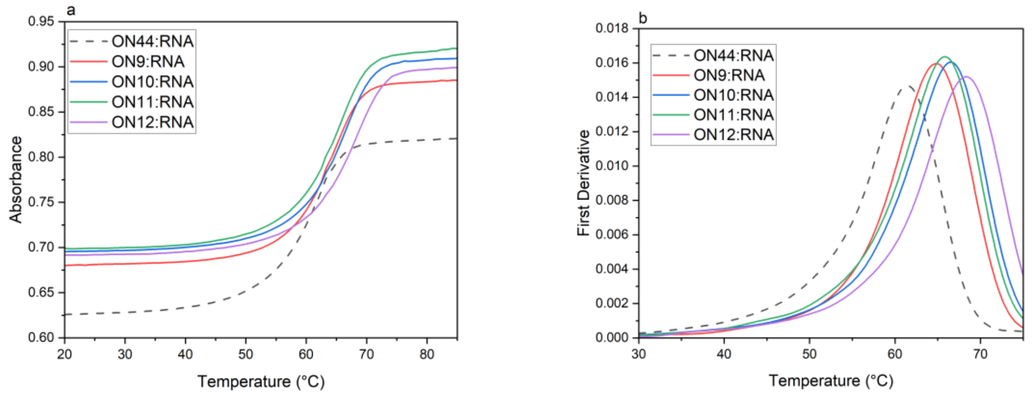

Supplementary Figure S147: UV melting studies for modified ONs **(ON9-ON12)** against complementary RNA (**ON43**). **a.** Representative UV melting curves measured using 2 μM of each ON in 10 mM Na-phosphate buffer, 25 mM NaCl, pH = 7.0; **b.** 1st derivative of melting curves.

Supplementary Table T9: Duplex melting temperatures (Tm) in °C of **ON13-ON16** against DNA and RNA. Nucleotides in black have 2´-OMe ribose sugars and phosphorothioate internucleoside linkages. Nucleotides in red are locked nucleic acid phosphorothioate triester.

ON	Sequence (5´→3´)	iPr group	DNA target (ON42)		RNA target (ON43)	
			Tm	(ΔTm)	Tm	(ΔTm)
ON13 iPr	CCU CUU ACC UCA GUT ACA	1	52.3	+3.8	64.9	+3.6
ON14 iPr	CCU CUT ACC UCA GUT ACA	2	56.4	+7.9	68.2	+6.9
ON15 iPr	CCT CUT ACC TCA GUT ACA	4	62.0	+13.5	74.3	+13.0
ON16 iPr	CCT CTT ACC TCA GTT ACA	6	66.7	+18.2	>75.0	Nd

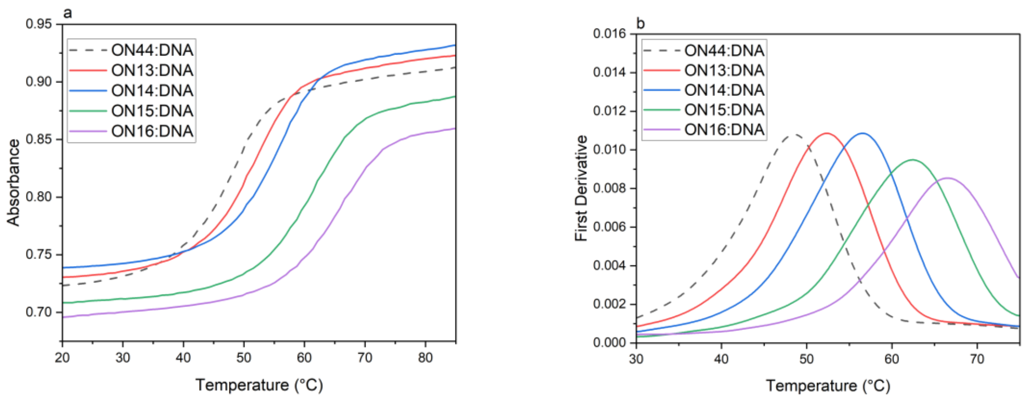

Supplementary Figure S148: UV melting studies for modified ONs **(ON13-ON16)** against complementary DNA (**ON42**). **a.** Representative UV melting curves measured using 2 µM of each ON in 10 mM Na-phosphate buffer, 100 mM NaCl, pH = 7.0; **b.** 1st derivative of melting curves.

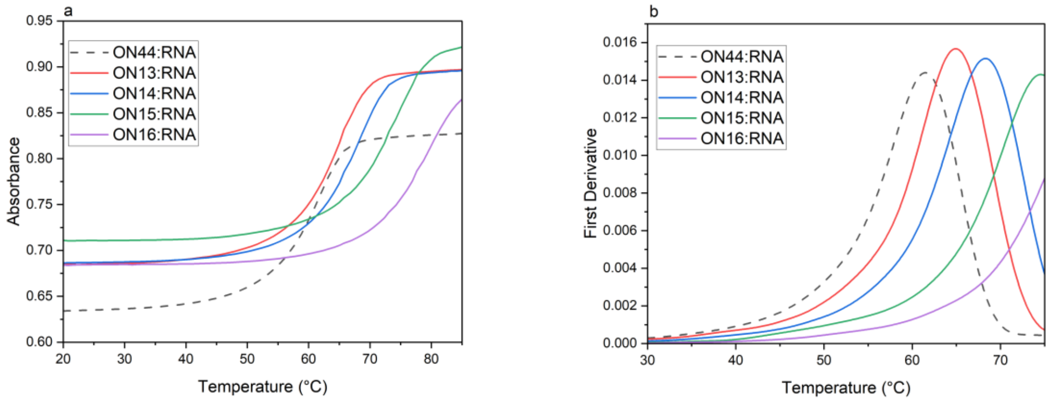

Supplementary Figure S149: UV melting studies for modified ONs **(ON13-ON16)** against complementary RNA (**ON43**). **a.** Representative UV melting curves measured using 2 µM of each ON in 10 mM Na-phosphate buffer, 25 mM NaCl, pH = 7.0; **b.** 1st derivative of melting curves.

4.3 LNA phosphorothiaote oligonucleotides

Supplementary Table T10: Duplex melting temperatures (Tm) in °C of **ON17-ON20** against DNA and RNA. Nucleotides in black have 2´-OMe ribose sugars and phosphorothioate internucleoside linkages. Nucleotides in red are locked nucleic acid phosphorothioate.

**ON**	Sequence (5´→3´)	DNA target (**ON42**)		RNA target (**ON43**)	
		Tm	(ΔTm)	Tm	(ΔTm)
ON17 LNA control	CCU CUU ACC UCA GUU ACA	53.4	+4.9	65.7	+4.4
ON18 LNA control	CCU CUU ACC UCA GUU ACA	56.4	+7.9	67.8	+6.5
ON19 LNA control	CCU CUU ACC UCA GUU ACA	55.6	+7.1	67.0	+5.7
ON20 LNA control	CCU CUU ACC UCA GUU ACA	59.2	+10.7	70.1	+8.9

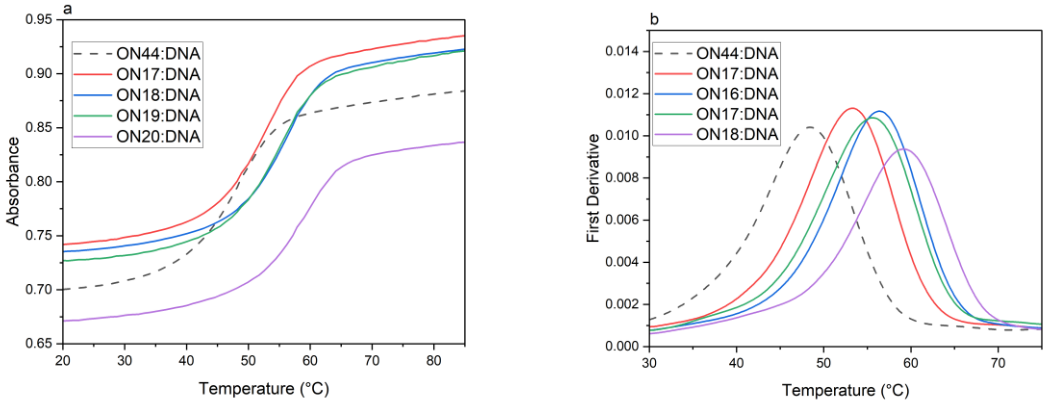

Supplementary Figure S150: UV melting studies for modified ONs **(ON17-ON20)** against complementary DNA (**ON42**). **a.** Representative UV melting curves measured using 2 µM of each ON in 10 mM Na-phosphate buffer, 100 mM NaCl, pH = 7.0; **b.** 1st derivative of melting curves.

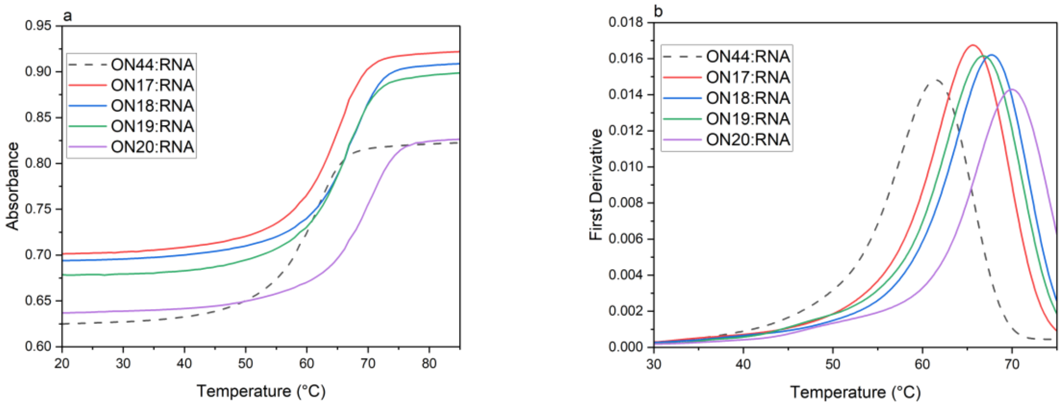

Supplementary Figure S151: UV melting studies for modified ONs **(ON17-ON20)** against complementary RNA (**ON43**). **a.** Representative UV melting curves measured using 2 µM of each ON in 10 mM Na-phosphate buffer, 25 mM NaCl, pH = 7.0; **b.** 1st derivative of melting curves.

Supplementary Table T11: Duplex melting temperatures (Tm) in °C of **ON21-ON24** against DNA and RNA. Nucleotides in black have 2´-OMe ribose sugars and phosphorothioate internucleoside linkages. Nucleotides in red are locked nucleic acid phosphorothioate.

ON	Sequence (5´→3´)	DNA target (**ON42**)		RNA target (**ON43**)	
		Tm	(ΔTm)	Tm	(ΔTm)
ON21 LNA control	CCU CUU ACC UCA GUT ACA	54.2	+5.7	66.6	+5.3
ON22 LNA control	CCU CUT ACC UCA GUT ACA	58.9	+10.4	70.4	+9.1
ON23 LNA control	CCT CUT ACC TCA GUT ACA	67.1	+18.6	>75.0	Nd
ON24 LNA control	CCT CTT ACC TCA GTT ACA	73.3	+24.8	>75.0	Nd

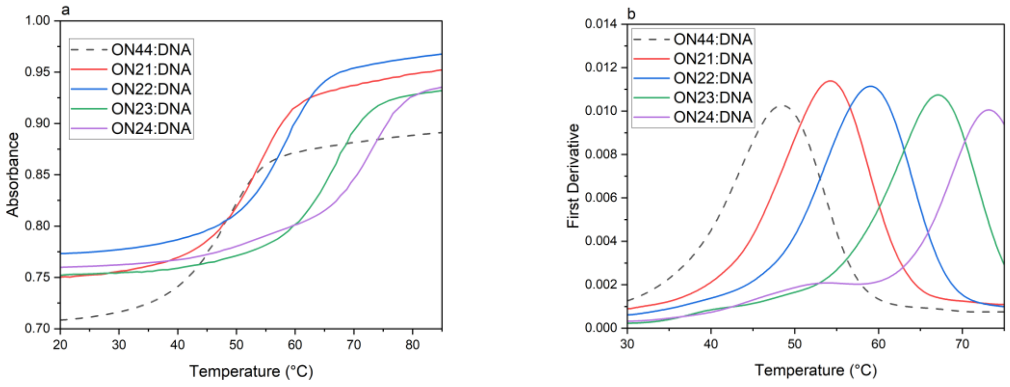

Supplementary Figure S152: UV melting studies for modified ONs **(ON21-ON24)** against complementary DNA (**ON42**). **a.** Representative UV melting curves measured using 2 µM of each ON in 10 mM Na-phosphate buffer, 100 mM NaCl, pH = 7.0; **b.** 1st derivative of melting curves.

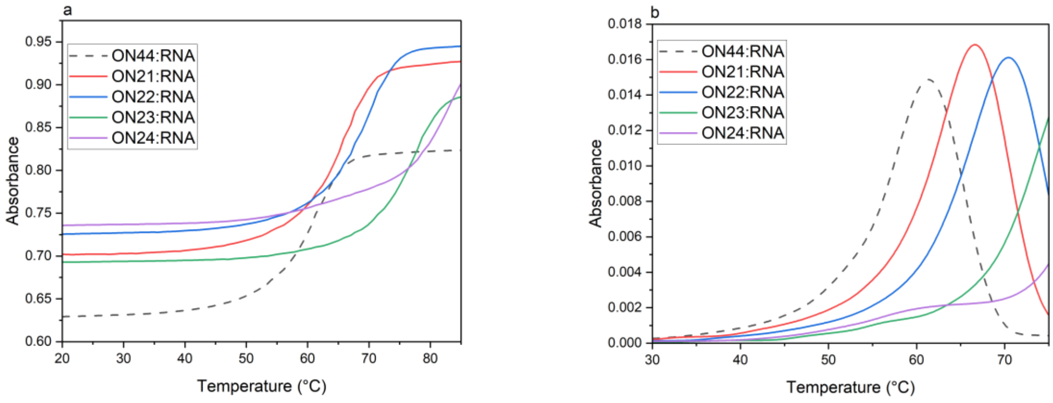

Supplementary Figure S153: UV melting studies for modified ONs **(ON21-ON24)** against complementary RNA (**ON43**). **a.** Representative UV melting curves measured using 2 µM of each ON in 10 mM Na-phosphate buffer, 25 mM NaCl, pH = 7.0; **b.** 1st derivative of melting curves.

4.4 THP PTTE oligonucleotides

Supplementary Table T12: Duplex melting temperatures (Tm) in °C of **ON25-ON28** against DNA and RNA. Nucleotides in black have 2´-OMe ribose sugars and phosphorothioate internucleoside linkages. Nucleotides in red are locked nucleic acid phosphorothioate triester.

ON	Sequence (5´→3´)	THP group	DNA target (**ON42**)		RNA target (**ON43**)	
			Tm	(ΔTm)	Tm	(ΔTm)
ON25 THP	CCU CUU ACC UCA GUU ACA	1	51.8	+3.3	64.4	+3.1
ON26 THP	CCU CUU ACC UCA GUU ACA	2	54.5	+5.0	66.4	+5.1
ON27 THP	CCU CUU ACC UCA GUU ACA	2	52.7	+4.2	64.7	+3.4
ON28 THP	CCU CUU ACC UCA GUU ACA	3	55.1	+6.6	66.6	+5.3

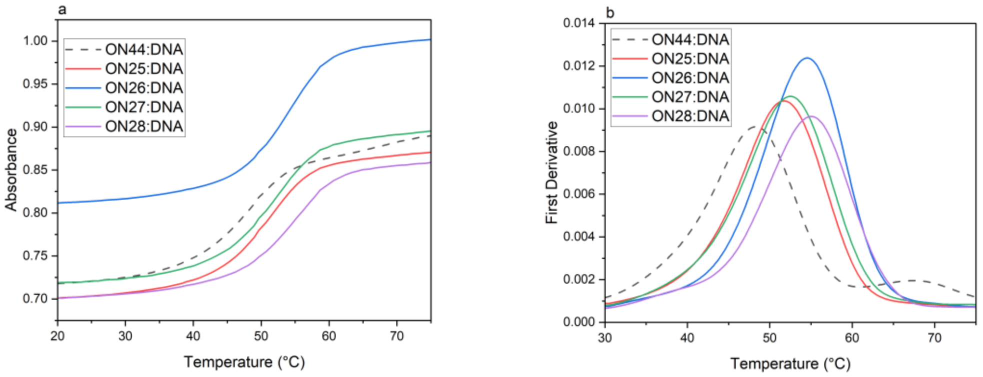

Supplementary Figure S154: UV melting studies for modified ONs **(ON25-ON28)** against complementary DNA (**ON42**). **a.** Representative UV melting curves measured using 2 µM of each ON in 10 mM Na-phosphate buffer, 100 mM NaCl, pH = 7.0; **b.** 1st derivative of melting curves.

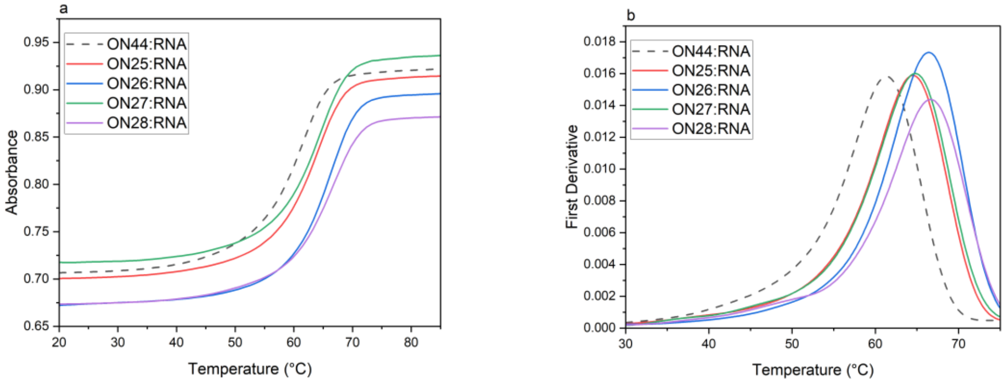

Supplementary Figure S155: UV melting studies for modified ONs **(ON25-ON28)** against complementary RNA (**ON43**). **a.** Representative UV melting curves measured using 2 µM of each ON in 10 mM Na-phosphate buffer, 25 mM NaCl, pH = 7.0; **b.** 1st derivative of melting curves.

Supplementary Table T13: Duplex melting temperatures (Tm) in °C of **ON29-ON34** against DNA and RNA. Nucleotides in black have 2´-OMe ribose sugars and phosphorothioate internucleoside linkages. Nucleotides in red are locked nucleic acid phosphorothioate triester.

ON	Sequence (5´→3´)	THP group	DNA target (**ON42**)		RNA target (**ON43**)	
			Tm	(ΔTm)	Tm	(ΔTm)
ON29 THP	CCU CUU ACC UCA GUT ACA	1	52.0	+3.5	64.8	+3.5
ON30 THP	CCU CUT ACC UCA GUT ACA	2	55.4	+6.9	67.6	+6.3
ON31 THP	CCT CUT ACC TCA GUT ACA	4	60.3	+11.8	72.7	+11.4
ON32 THP	CCT CTT ACC TCA GTT ACA	6	64.2	+15.7	>75.0	Nd
ON33 THP	CCT CUT ACC TCA GUT ACA	7	62.4	+13.9	>75.0[I]	Nd
ON34 THP	CCT CTT ACC TCA GTT ACA	9	67.8	+19.3	>75.0[I]	Nd

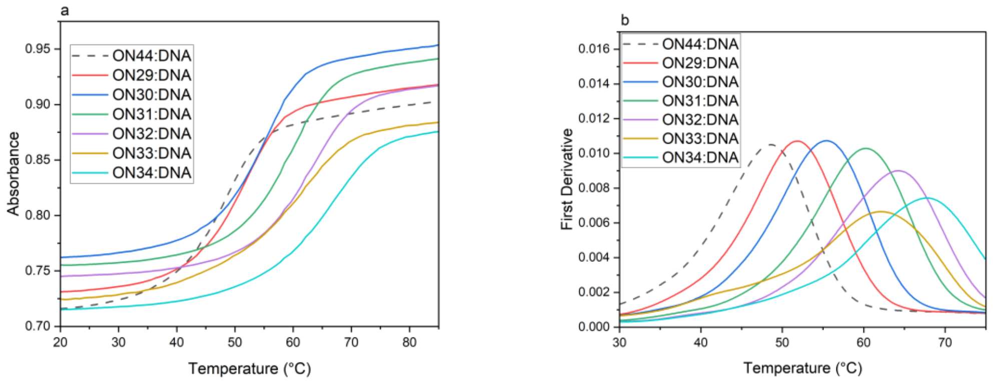

Supplementary Figure S156: UV melting studies for modified ONs **(ON29-ON34)** against complementary DNA (**ON42**). **a.** Representative UV melting curves measured using 2 µM of each ON in 10 mM Na-phosphate buffer, 100 mM NaCl, pH = 7.0; **b.** 1st derivative of melting curves.

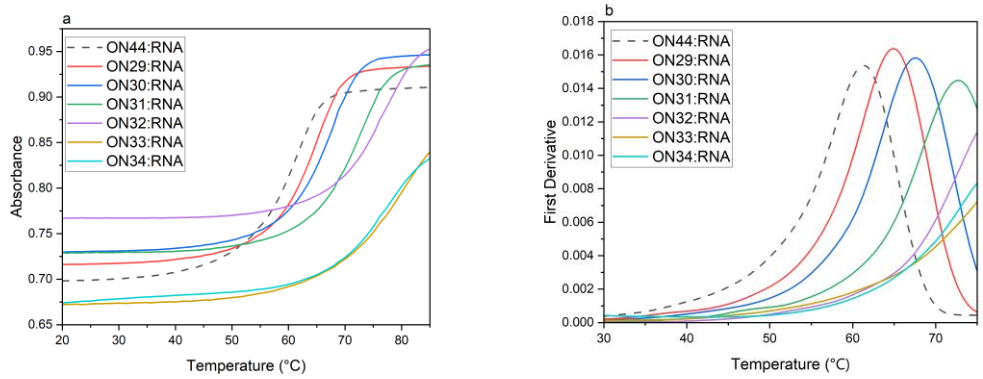

Supplementary Figure S157: UV melting studies for modified ONs **(ON29-ON34)** against complementary RNA (**ON43**). **a.** Representative UV melting curves measured using 2 µM of each ON in 10 mM Na-phosphate buffer, 25 mM NaCl, pH = 7.0; **b.** 1st derivative of melting curves.

4.5 C16 PTTE oligonucleotides

Supplementary Table T14: Duplex melting temperatures (Tm) in °C of **ON35-ON38** against DNA and RNA. Nucleotides in black have 2´-OMe ribose sugars and phosphorothioate internucleoside linkages. Nucleotides in red are locked nucleic acid phosphorothioate triester.

ON	Sequence (5´→3´)	$C_{16}H_{33}$ group	DNA target (**ON42**)		RNA target (**ON43**)	
			Tm	(ΔTm)	Tm	(ΔTm)
ON35 C16	CCU CUU ACC UCA GUU ACA	1	47.9	-0.6	61.0 (68.7)[I]	-0.3 (-4.8)[I]
ON36 C16	CCU CUU ACC UCA GUU ACA	2	40.6	-7.9	56.1 (63.3)[I]	-5.2 (-10.2)[I]
ON37 C16	CCU CUU ACC UCA GUU ACA	2	39.9	-8.6	55.6 (62.4)[I]	-5.7 (-11.1)[I]
ON38 C16	CCU CUU ACC UCA GUU ACA	3	Nf	Nd	47.2 (53.6)[I]	-14.1 (-19.9)[I]

[I]Tm value was measures at 200 mM NaCl concentration in 10 mM Na-Phosphate buffer pH 7.0. Tm value of the control oligo **ON44** is 73.5 °C at 200 mM NaCl concentration in 10 mM Na-Phosphate buffer pH 7.0. Nd = Not determined; Nf: Not found.

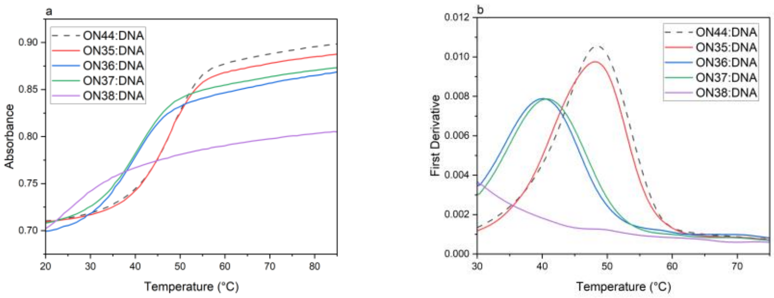

Supplementary Figure S158: UV melting studies for modified ONs **(ON35-ON38)** against complementary DNA (**ON42**). **a.** Representative UV melting curves measured using 2 µM of each ON in 10 mM Na-phosphate buffer, 100 mM NaCl, pH = 7.0; **b.** 1st derivative of melting curves.

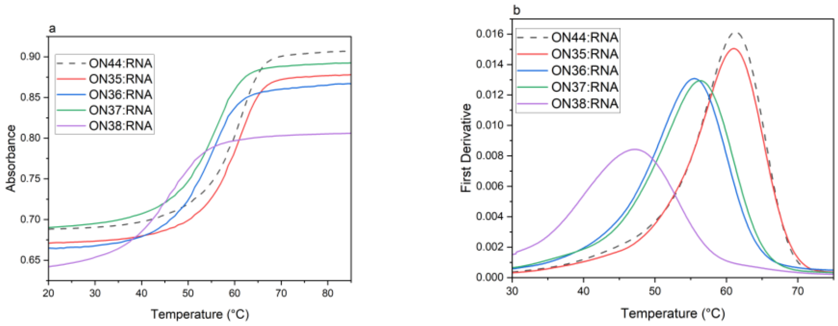

Supplementary Figure S159: UV melting studies for modified ONs **(ON35-ON38)** against complementary RNA (**ON43**). **a.** Representative UV melting curves measured using 2 µM of each ON in 10 mM Na-phosphate buffer, 25 mM NaCl, pH = 7.0; **b.** 1st derivative of melting curves.

4.6 Hexyn PTTE oligonucleotides

Supplementary Table T15: Duplex melting temperatures (Tm) in °C of **ON1-ON4** against DNA and RNA. Nucleotides in black have 2´-OMe ribose sugars and phosphorothioate internucleoside linkages. Nucleotides in red are locked nucleic acid phosphorothioate triester.

ON	Sequence (5´→3´)	Hexynyl group	DNA target (**ON42**)		RNA target (**ON43**)	
			Tm	(ΔTm)	Tm	(ΔTm)
ON39 Hexynyl	CCU CUU ACC UCA GUU ACA	1	51.1	+2.6	63.7	+2.4
ON40 Hexynyl	CCU CUU ACC UCA GUU ACA	2	51.5	+3.0	64.3	+3.0
ON41 Hexynyl	CCU CUU ACC UCA GUU ACA	3	53.0	+4.5	64.7	+3.4

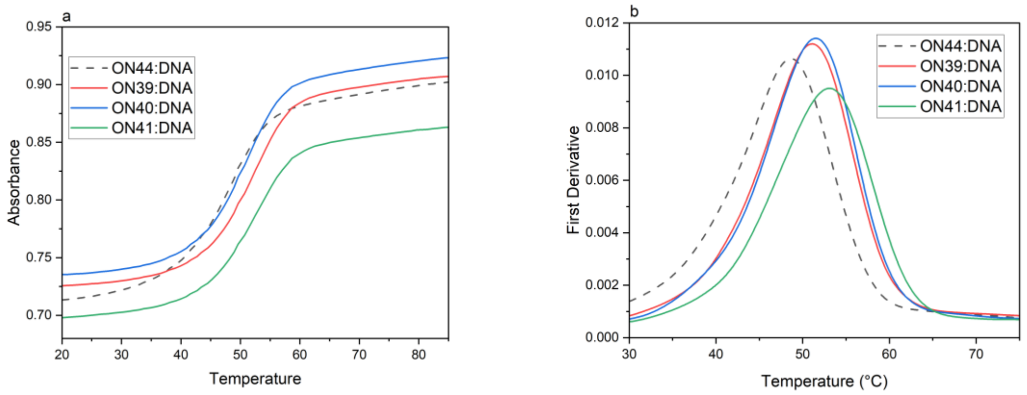

Supplementary Figure S160: UV melting studies for modified ONs **(ON39-ON41)** against complementary DNA (**ON42**). **a.** Representative UV melting curves measured using 2 µM of each ON in 10 mM Na-phosphate buffer, 100 mM NaCl, pH = 7.0; **b.** 1st derivative of melting curves.

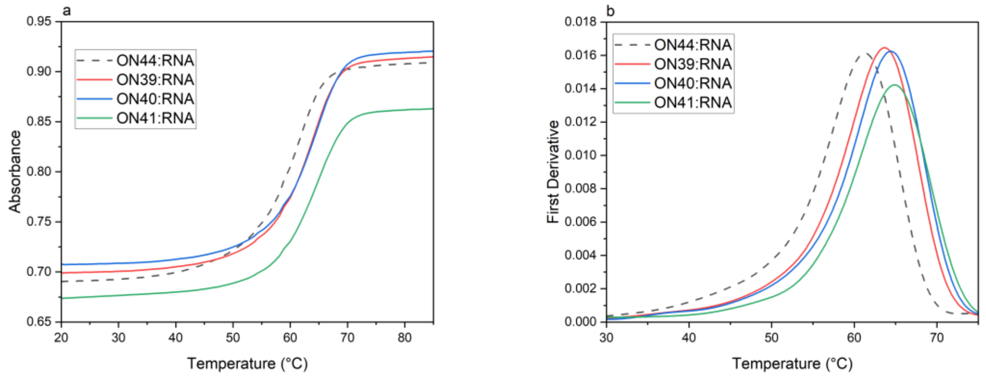

Supplementary Figure S161: UV melting studies for modified ONs **(ON39-ON41)** against complementary RNA (**ON43**). **a.** Representative UV melting curves measured using 2 µM of each ON in 10 mM Na-phosphate buffer, 25 mM NaCl, pH = 7.0; **b.** 1st derivative of melting curves.

4.7 UV melting study at different salt concentration

Supplementary Table T16: Tms were measured at 10 mM Na-phosphate buffer, pH = 7.0 at different NaCl concentration. **ΔTm ON34 THP** = (Tm of ON34 THP at different salt concentration - 24.3).

NaCl salt concentration (mM)	Tm DNA target		ΔTm ON34 THP
	ON44 control	ON34 THP	
10	33.8	58.1	33.8
25	38.8	61.4	37.1
50	43.6	64.4	40.0
100	48.5	67.8	43.4
150	51.9	69.3	45.0
200	53.6	70.0	45.7
250	55.1	71.1	46.7
300	56.4	71.8	47.5

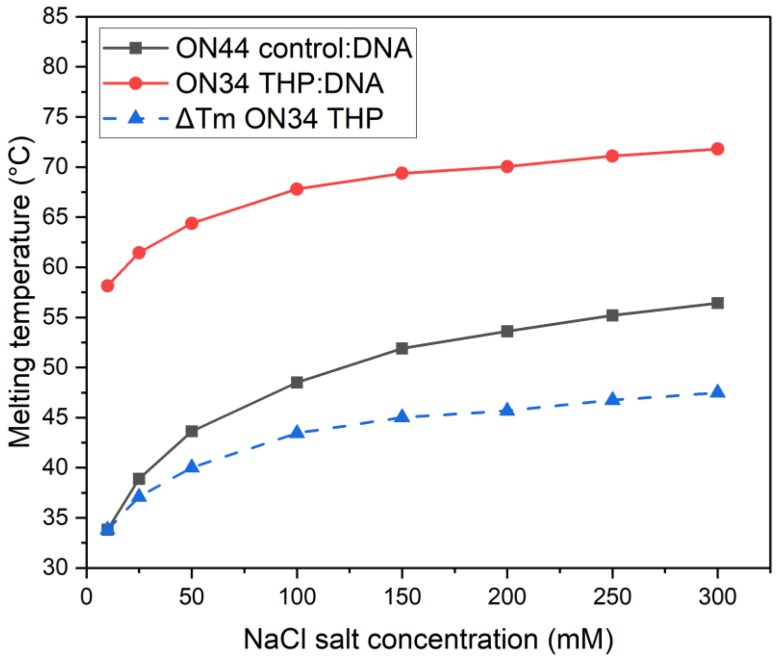

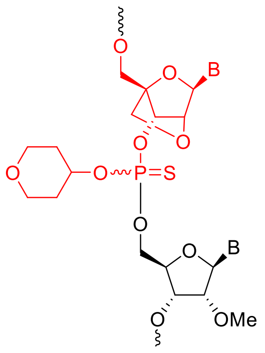

ON34 THP

Supplementary Figure S162: UV melting studies of ON44 THP and ON44 control at different salt concentration at different NaCl concentration against the complementary DNA (**ON42**).

4.8 UV melting experiments without NaCl salt

Supplementary Table T17: Duplex melting temperatures (Tm) in °C of **ON1-ON4** against RNA. Nucleotides in black have 2´-OMe ribose sugars and phosphorothioate internucleoside linkages. Nucleotides in red are locked nucleic acid phosphorothioate triester (except **ON23-ON24** those have phosphorothioate internucleoside).

**ON**	Sequence (5´→3´)	RNA target (**ON43**)	
		Tm	(ΔTm)
ON44 2'-O-methyl RNA control	CCU CUU ACC UCA GUU ACA	53.4	--
ON8 MeOPr	CCT CTT ACC TCA GTT ACA	69.3	+16.9
ON16 iPr	CCT CTT ACC TCA GTT ACA	72.5	+19.1
ON23 LNA control	CCT CUT ACC TCA GUT ACA	69.5	+16.1
ON24 LNA control	CCT CTT ACC TCA GTT ACA	75.4	+22.0
ON32 THP	CCT CTT ACC TCA GTT ACA	70.4	+17.0
ON33 THP	CCT CUT ACC TCA GUT ACA	67.8	+14.4
ON34 THP	CCT CTT ACC TCA GTT ACA	74.9	+21.5

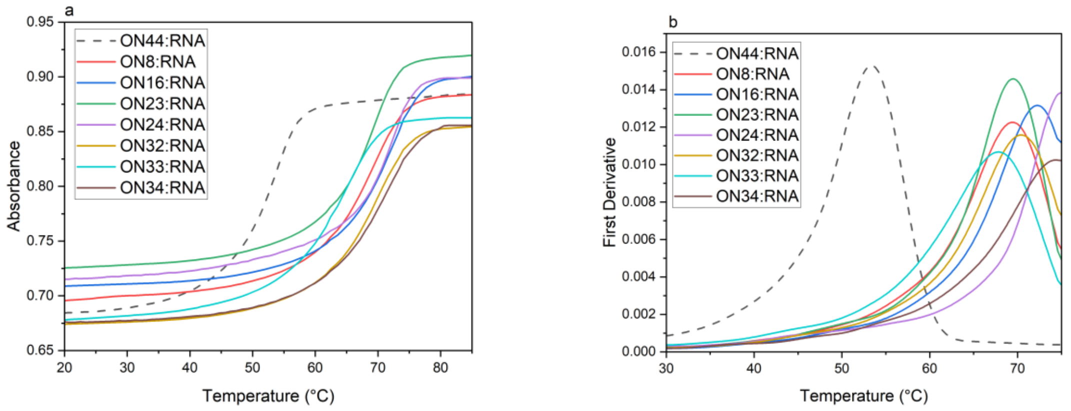

Supplementary Figure S163: UV melting studies for modified ONs **(ON39-ON41)** against complementary RNA (**ON43**). **a.** Representative UV melting curves measured using 2 µM of each ON in 10 mM Na-phosphate buffer, pH = 7.0; **b.** 1st derivative of melting curves.

5.0 Circular dichroism (CD)

CD spectra were acquired with a Chirascan CD spectrometer (Applied Photophysics Ltd). Duplex sequences (2 μM) were measured in 2 mm quartz cuvettes at 20 °C. Spectra were obtained by the accumulation of six scans at a speed 20nm/min over a range of 200–330 nm, a bandwidth of 1 nm.

5.1 CD spectra of oligonucleotide:RNA duplex

Nucleotides in black have 2´-OMe ribose sugars and phosphorothioate internucleoside linkages. Nucleotides in red are locked nucleic acid phosphorothioate triester.

Oligonucleotide	Sequence (5´→3´)	Alkyl group	PTTE
ON4 MeOPr	CCU CUUACCUCAGUUACA	**MeOPr**	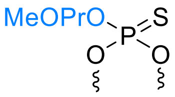
ON12 iPr	CCUCUUACCUCAGUUACA	**ⁱPr**	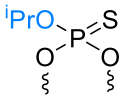
ON20 LNA control	CCUCUUACCUCAGUUACA	**No alkyl group**	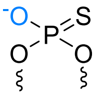
ON28 THP	CCUCUUACCUCAGUUACA	**THP**	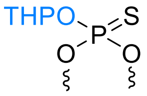
ON44 RNA control	CCUCUUACCUCAGUUACA (2´-OMe-PS)		

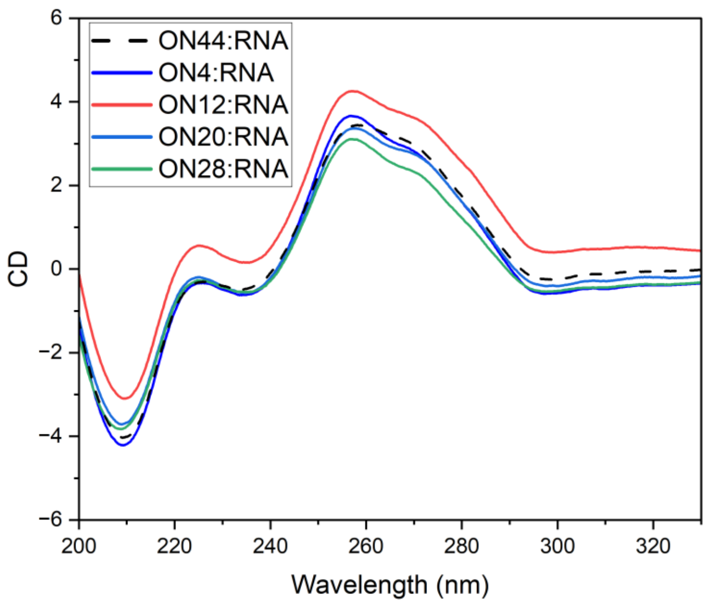

Supplementary Figure S164: CD spectra of oligonucleotides-RNA (**ON43**) duplex at in 10 mM Na-Phosphate buffer at 25 mM NaCl pH 7.0. Y-axis is ellipticity θ, (10^{-3} deg.cm^2/dmol).

Nucleotides in black have 2´-OMe ribose sugars and phosphorothioate internucleoside linkages. Nucleotides in red are locked nucleic acid phosphorothioate triester.

Oligonucleotide	Sequence (5´→3´)	Modification	PTTE
ON35 C16	CCUCUUACCUCAGUUACA	**C16**	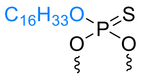
ON36 C16	CCUCUUACCUCAGUUACA		
ON37 C16	CCUCUUACCUCAGUUACA		
ON38 C16	CCUCUUACCUCAGUUACA		
ON44 RNA control	CCUCUUACCUCAGUUACA (2´-OMe-PS)		

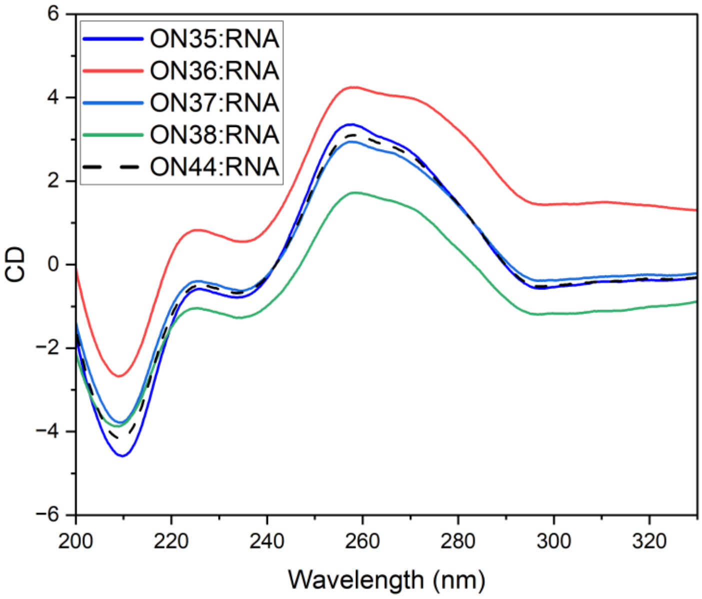

Supplementary Figure S165: CD spectra of oligonucleotides-RNA (**ON43**) duplex at in 10 mM Na-Phosphate buffer at 200 mM NaCl at pH 7.0. Y-axis is ellipticity θ, (10^{-3} deg.cm^2/dmol).

5.2 CD spectra of oligonucleotide:DNA duplex

Nucleotides in black have 2´-OMe ribose sugars and phosphorothioate internucleoside linkages. Nucleotides in red are locked nucleic acid phosphorothioate triester.

Oligonucleotide	Sequence (5´→3´)	Modification	PTTE
ON13 iPr	CCUCUUACCUCAGUTACA	^{i}Pr	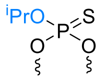
ON14 iPr	CCUCUTACCUCAGUTACA		
ON15 iPr	CCTCUTACCTCAGUTACA		
ON16 iPr	CCTCTTACCTCAGTTACA		
ON44 RNA control	CCUCUUACCUCAGUUACA (2´-OMe-PS**)**		

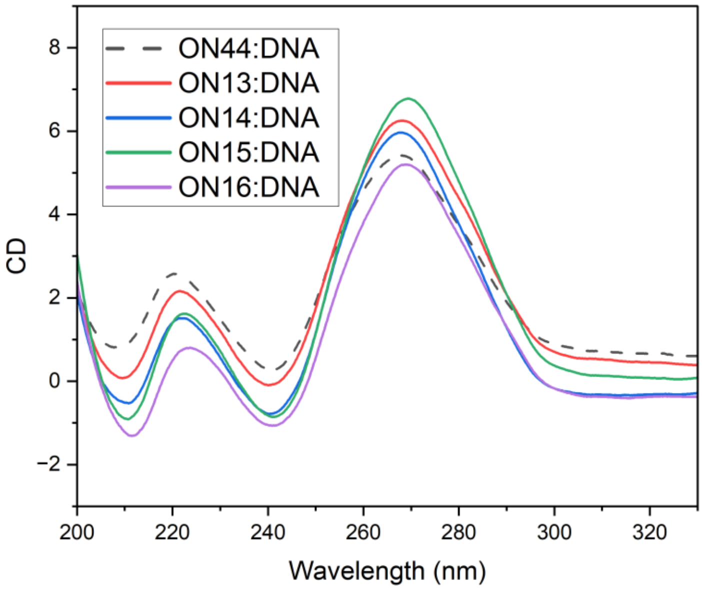

Supplementary Figure S166: CD spectra of oligonucleotides (**ON13-ON16**): DNA (**ON42**) duplex in 10 mM Na-Phosphate buffer pH 7.0 at 100 mM NaCl. Y-axis is ellipticity θ, (10^{-3} deg.cm^2/dmol).

Nucleotides in black have 2´-OMe ribose sugars and phosphorothioate internucleoside linkages. Nucleotides in red are locked nucleic acid phosphorothioate triester.

Oligonucleotide	Sequence (5´→3´)	Modification	Phosphorothioate
ON21 LNA control	CCUCUUACCUCAGUTACA	No alkyl group	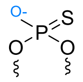
ON22 LNA control	CCUCUTACCUCAGUTACA		
ON23 LNA control	CCTCUTACCTCAGUTACA		
ON24 LNA control	CCTCTTACCTCAGTTACA		
ON44 RNA control	CCUCUUACCUCAGUUACA (2´-OMe-PS)		

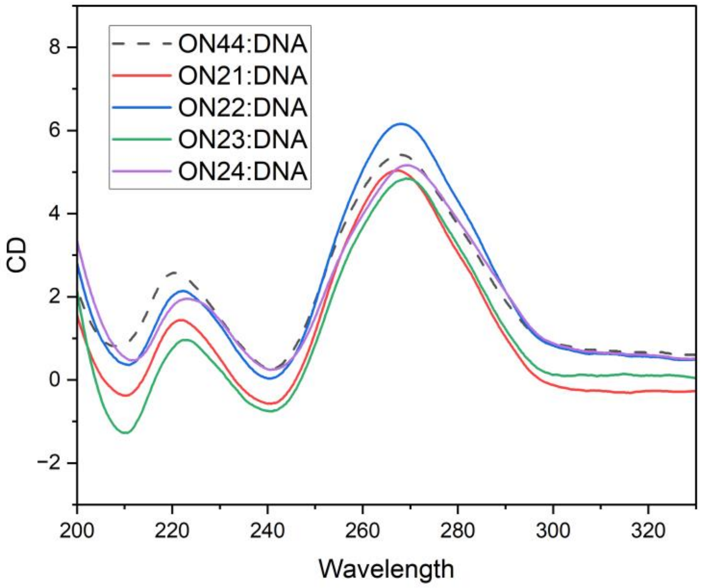

Supplementary Figure S167: CD spectra of oligonucleotides (**ON21-ON24**): DNA (**ON42**) duplex in 10 mM Na-Phosphate buffer pH 7.0 at 100 mM NaCl. Y-axis is ellipticity θ, (10^{-3} deg.cm^2/dmol).

Nucleotides in black have 2´-OMe ribose sugars and phosphorothioate internucleoside linkages. Nucleotides in red are locked nucleic acid phosphorothioate triester.

Oligonucleotide	Sequence (5´→3´)	Modification	PTTE
ON29 THP	CCUCUUACCUCAGUTACA	THP	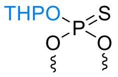
ON30 THP	CCUCUTACCUCAGUTACA		
ON31 THP	CCTCUTACCTCAGUTACA		
ON32 THP	CCTCTTACCTCAGTTACA		
ON33 THP	CCTCUTACCTCAGUTACA		
ON34 THP	CCTCTTACCTCAGTTACA		
ON44 RNA control	CCUCUUACCUCAGUUACA (2´-OMe-PS)		

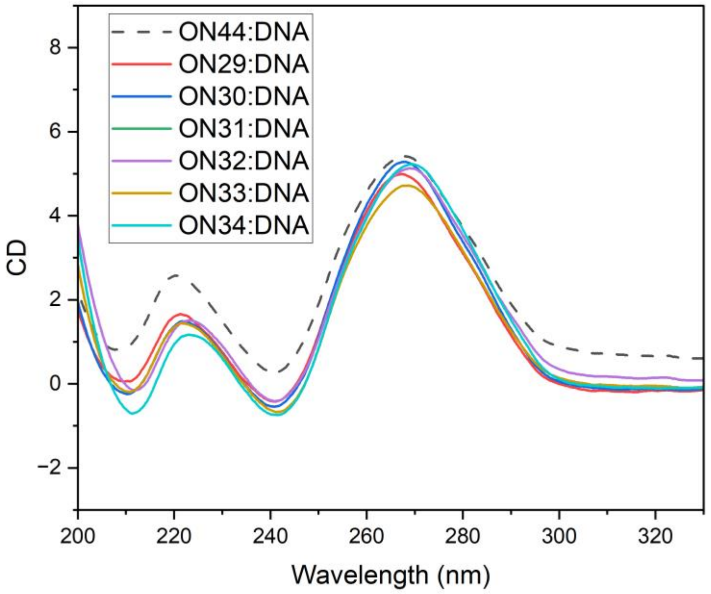

Supplementary Figure S168: CD spectra of oligonucleotides (**ON29-ON34**): DNA (**ON42**) duplex in 10 mM Na-Phosphate buffer pH 7.0 at 100 mM NaCl. Y-axis is ellipticity θ, (10^{-3} deg.cm^2/dmol).

Nucleotides in black have 2´-OMe ribose sugars and phosphorothioate internucleoside linkages. Nucleotides in red are locked nucleic acid phosphorothioate triester.

**Oligonucleotide**	**Sequence (5´→3´)**	**Modification**	**PTTE**
ON35 C16	CCUCUUACCUCAGUUACA	**C16**	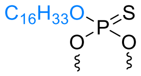
ON36 C16	CCUCUUACCUCAGUUACA		
ON37 C16	CCUCUUACCUCAGUUACA		
ON38 C16	CCUCUUACCUCAGUUACA		
ON44 RNA control	CCUCUUACCUCAGUUACA (2´-OMe-PS)		

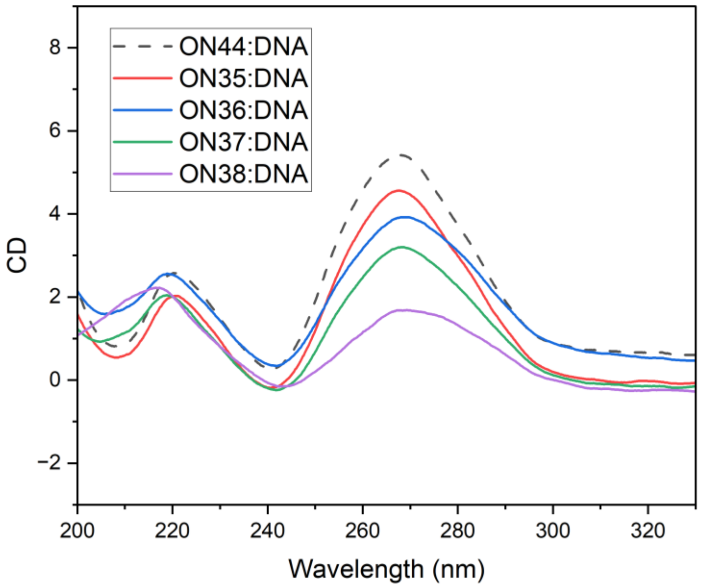

Supplementary Figure S169: CD spectra of oligonucleotides (**ON35-ON38**): DNA (**ON42**) duplex in 10 mM Na-Phosphate buffer pH 7.0 at 100 mM NaCl. Y-axis is ellipticity θ, (10^{-3} deg.cm^2/dmol).

Nucleotides in black have 2´-OMe ribose sugars and phosphorothioate internucleoside linkages. Nucleotides in red are locked nucleic acid phosphorothioate triester.

Oligonucleotide	Sequence (5´→3´)	Modification	PTTE
ON39 Hexynyl	CCUCUUACCUCAGUUACA	**Hexynyl**	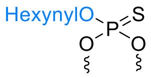
ON40 Hexynyl	CCUCUUACCUCAGUUACA		
ON41 Hexynyl	CCUCUUACCUCAGUUACA		
ON44 Hexynyl	CCUCUUACCUCAGUUACA (2´-OMe-PS**)**		

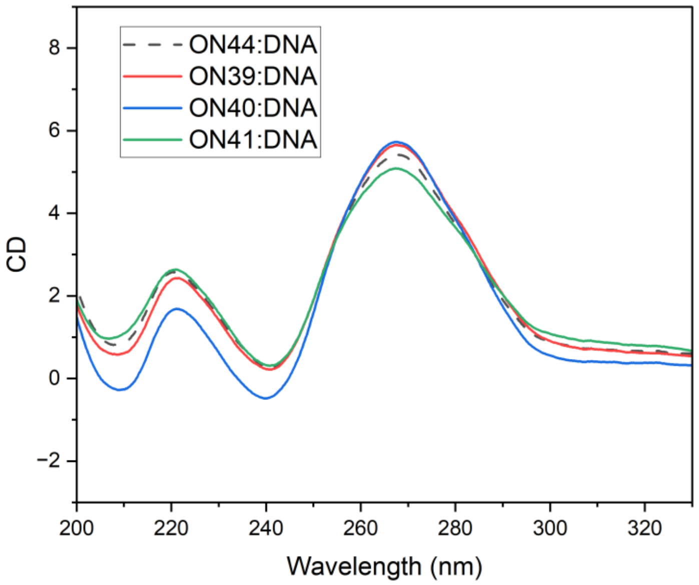

Supplementary Figure S170: CD spectra of oligonucleotides (**ON39-ON41**): DNA (**ON42**) duplex in 10 mM Na-Phosphate buffer pH 7.0 at 100 mM NaCl. Y-axis is ellipticity θ, (10^{-3} deg.cm^2/dmol).

6.0 Enzymatic stability of selected oligonucleotide

Nuclease S1 from Aspergillus oryzae and Gibco PBS buffer (pH 7.0) were mixed and the control DNA (unmodified PO backbone, **ON 46** = **5´-CCT CTT ACC TCA GTT ACA-3´**) or modified oligonucleotide (**ON4**, **ON15, ON31, ON35** and **ON41**) was added to reach final concentration 5 µM. The sample was mixed in an eppendorf tube. 10 µL of the reaction mixture was immediately removed (t =0), mixed with formamide (10 µL) and stored at −20 °C (0 h). The remaining reaction mixtures were incubated at 37 °C. Aliquots (10 µL) were taken at different time intervals, mixed with formamide (10 µL), and stored at −20 °C. 10 µL of each sample was taken, 10 µl loading buffer was added and the protein denatured at 95 °C for 2 minutes. The samples were then analysed by denaturing 20% polyacrylamide gel electrophoresis.

ON4 MeOPr	CCU CUU ACC UCA GUU ACA	**3.MeOPr**	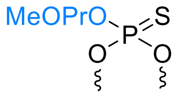

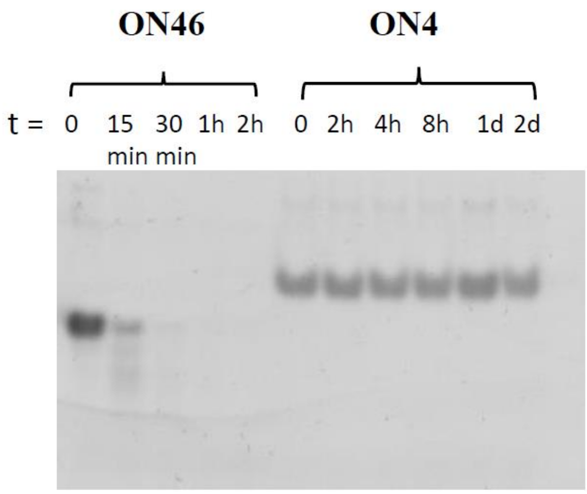

Supplementary Figure S171: Denaturing polyacrylamide gel electrophoresis (PAGE) analysis of modified **ON4** and unmodified **control ON46** after incubation in Nuclease S1 *Aspergillus oryzae* in PBS buffer. t = incubation time.

ON15 iPr	CCT CUT ACC TCA GUT ACA	**4.^{i}Pr**	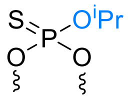

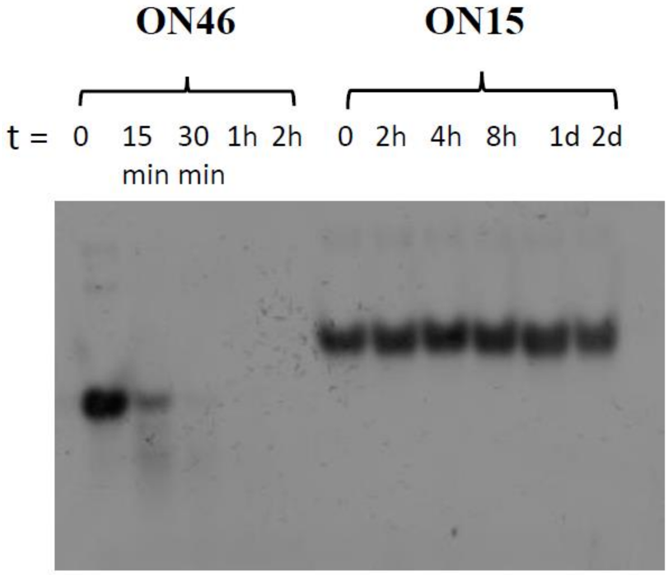

Supplementary Figure S172: Denaturing polyacrylamide gel electrophoresis (PAGE) analysis of modified **ON15** and unmodified **control ON46** after incubation in Nuclease S1 *Aspergillus oryzae* in PBS buffer. t = incubation time.

ON31 THP	CCT CUT ACC TCA GUT ACA	**4.THP**	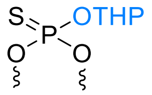

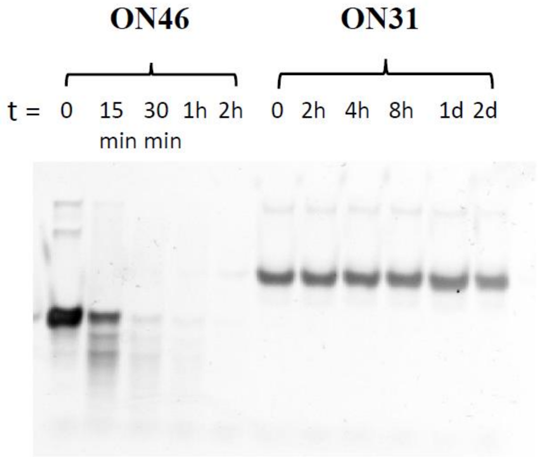

Supplementary Figure S173: Denaturing polyacrylamide gel electrophoresis (PAGE) analysis of modified **ON31** and unmodified **control ON46** after incubation in Nuclease S1 *Aspergillus oryzae* in PBS buffer. t = incubation time.

ON35 C16	CCU CUU ACC UCA GUU ACA	$C_{16}H_{33}$	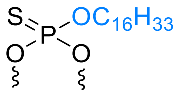

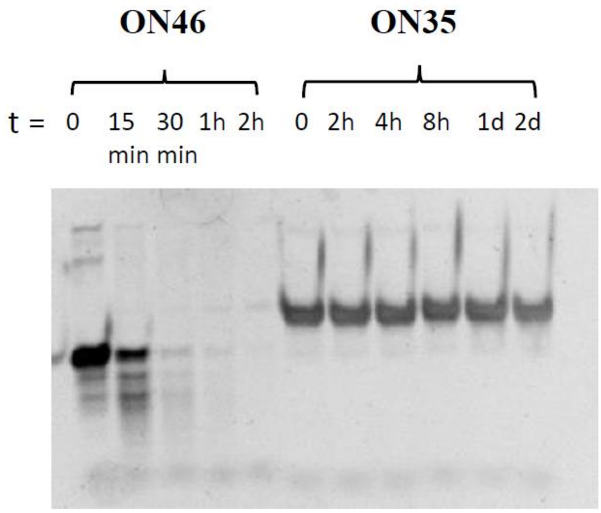

Supplementary Figure S174: Denaturing polyacrylamide gel electrophoresis (PAGE) analysis of modified **ON35** and unmodified **control ON46** after incubation in Nuclease S1 *Aspergillus oryzae* in PBS buffer. t = incubation time.

ON41 Hexynyl	CCU CUU ACC UCA GUU ACA	**3.Hexynyl**	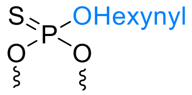

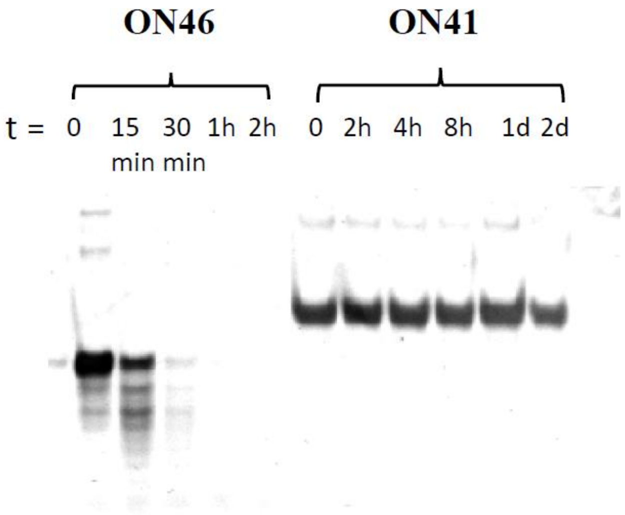

Supplementary Figure S175: Denaturing polyacrylamide gel electrophoresis (PAGE) analysis of modified **ON41** and unmodified **control ON46** after incubation in Nuclease S1 *Aspergillus oryzae* in PBS buffer. t = incubation time.

6.1 Enzymatic digestion study with model oligonucleotides

Supplementary Table T18: List of oligonucleotides made for enzymatic analysis containing PTE linkages.

Nucleotides in black have deoxy ribose sugars. Nucleotides in red are locked nucleic acids and THP triesters. All are phosphodiester linkages. Subscript ‘x’ is phosphorothioate linkage.

Oligonucleotides	Sequence (5′→3′)	Mass Expected	Mass Observed
ONS5 THP	TTT TTT TTA TTT	3709.51	3709.90
ONS6 THP	TTT TTT TTA$_X$ TTT	3725.57	3726.00
ONS7	TTT TTT TTT TTT	3588.37	3588.70
ONS8	TTT TTT TTT$_X$ TTT	3604.43	3604.40

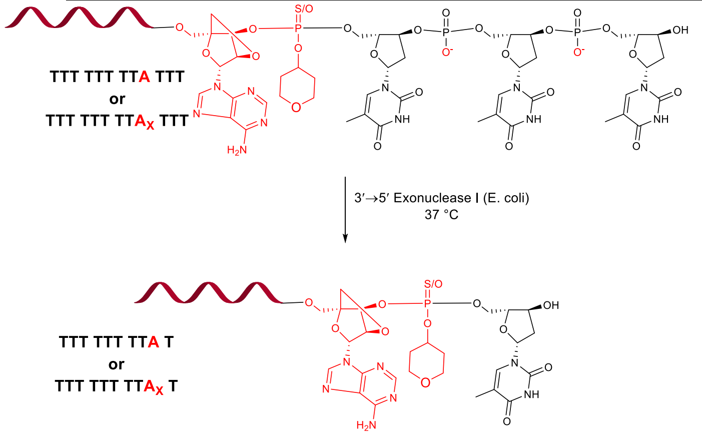

LNA triester (PO/PS) internucleotide linkage inhibits the digestion of oligonucleotide by the exonuclease.

After digestion, 12-mer nt ONS5 and ONS6 gave fully 10 mer nt.

Supplementary Figure S176: Enzymatic digestion of ONS5 and ONS6 with 3'→ 5' exonuclease I.

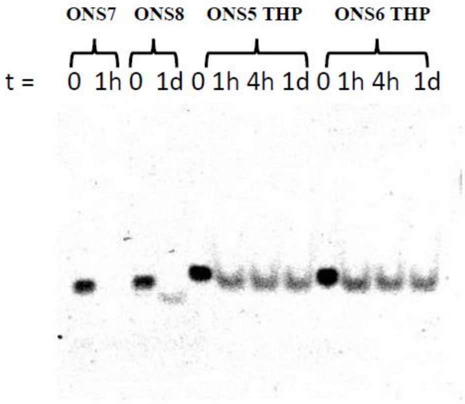

Supplementary Figure S177: Denaturing polyacrylamide gel electrophoresis (PAGE) analysis of modified **ONS5 and ONS6** and unmodified **control ONS7 and control ONS8** after incubation in Exonuclease I in NEBuffer™ 4 buffer.

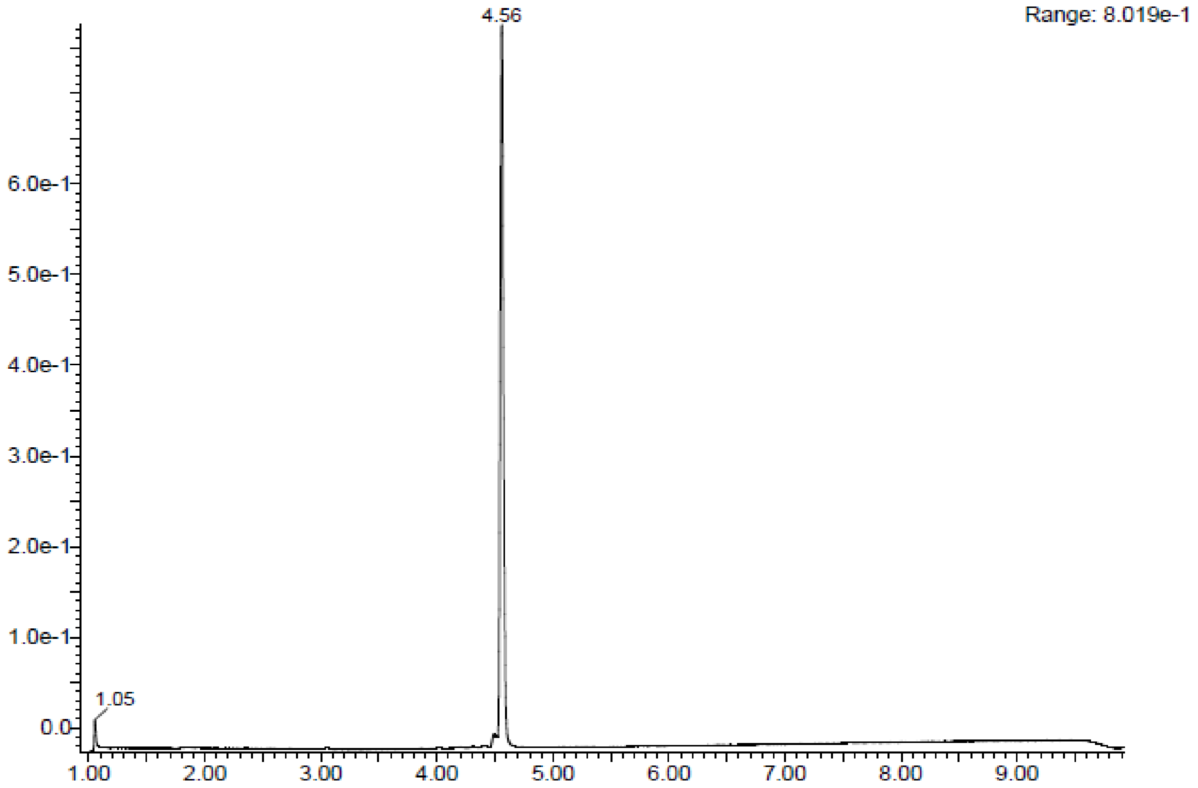

Supplementary Figure S178: Reverse-phase UPLC **ONS5** (UV absorbance at 260 nm vs time in min).

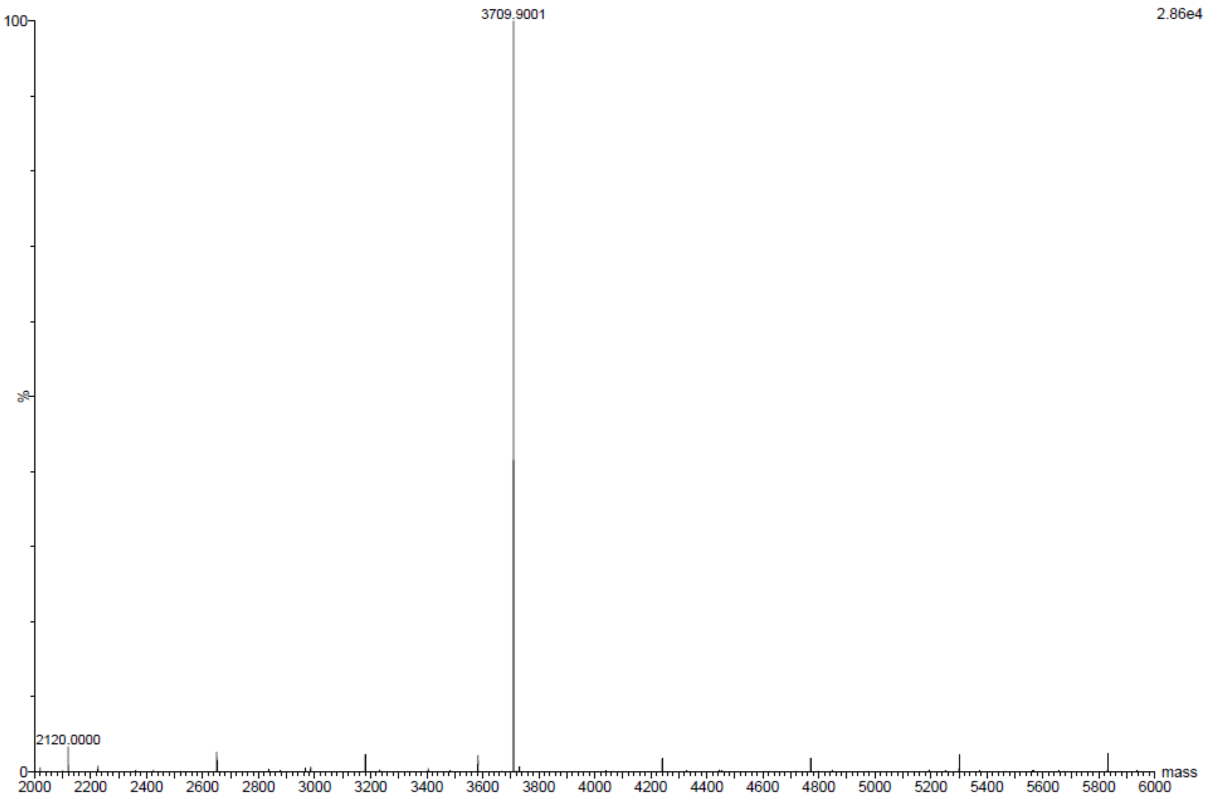

Supplementary Figure S179: Mass spectrum (ES-) of **ONS5**. Required **3725.57** Da, found **3726.00** Da. y-axis = relative intensity (%), x-axis = mass in Da.

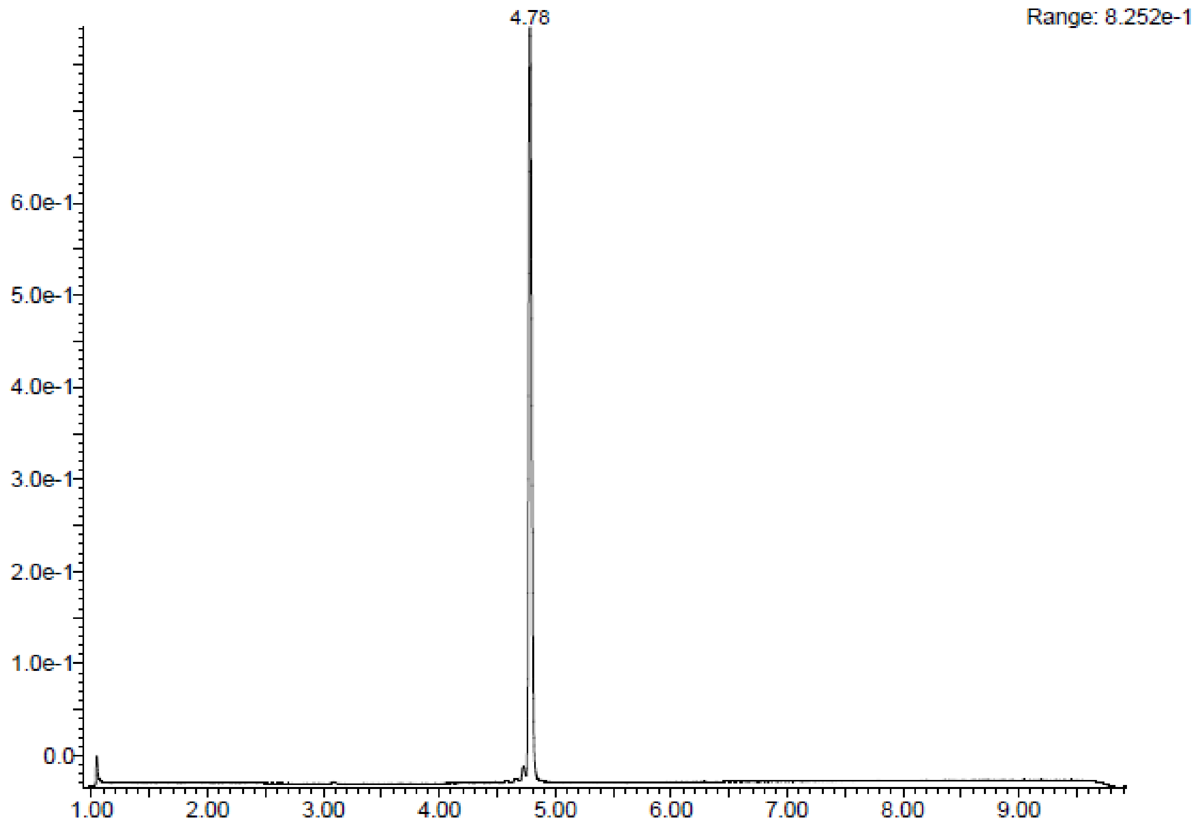

Supplementary Figure S180: Reverse-phase UPLC **ONS6** (UV absorbance at 260 nm vs time in min).

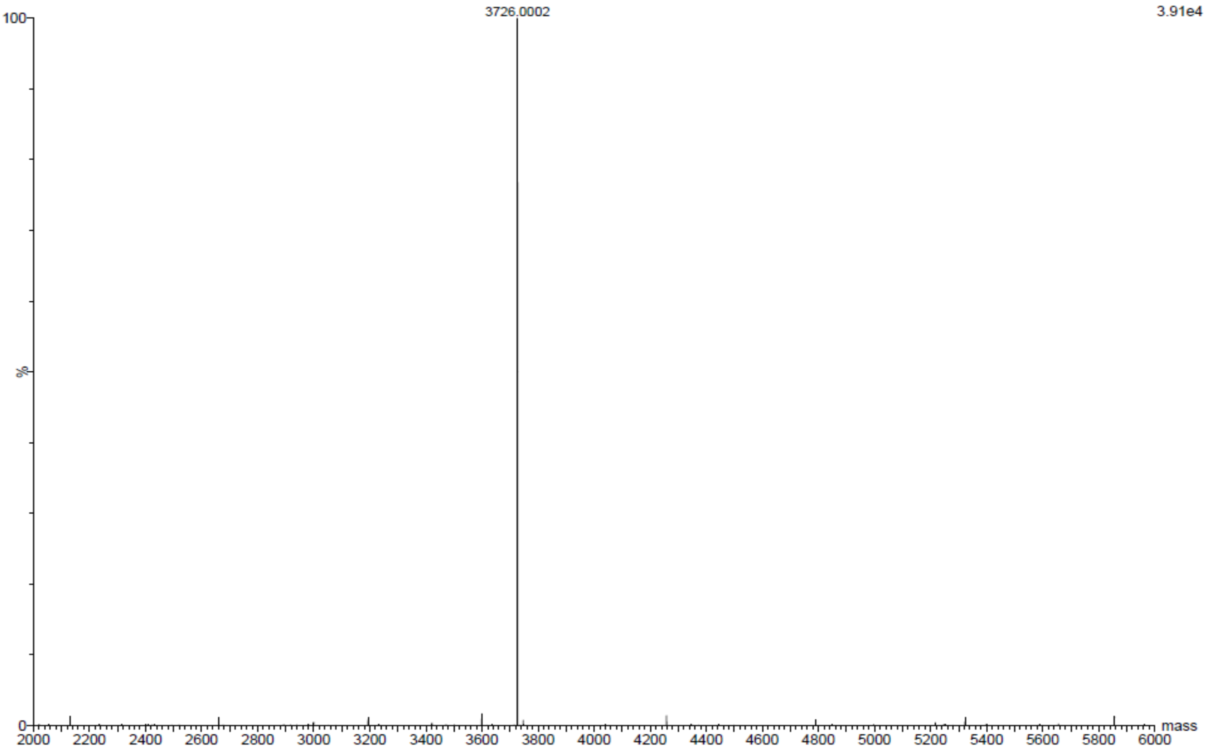

Supplementary Figure S181: Mass spectrum (ES-) of **ONS6**. Required **3709.51** Da, found **3709.90** Da. y-axis = relative intensity (%), x-axis = mass in Da.

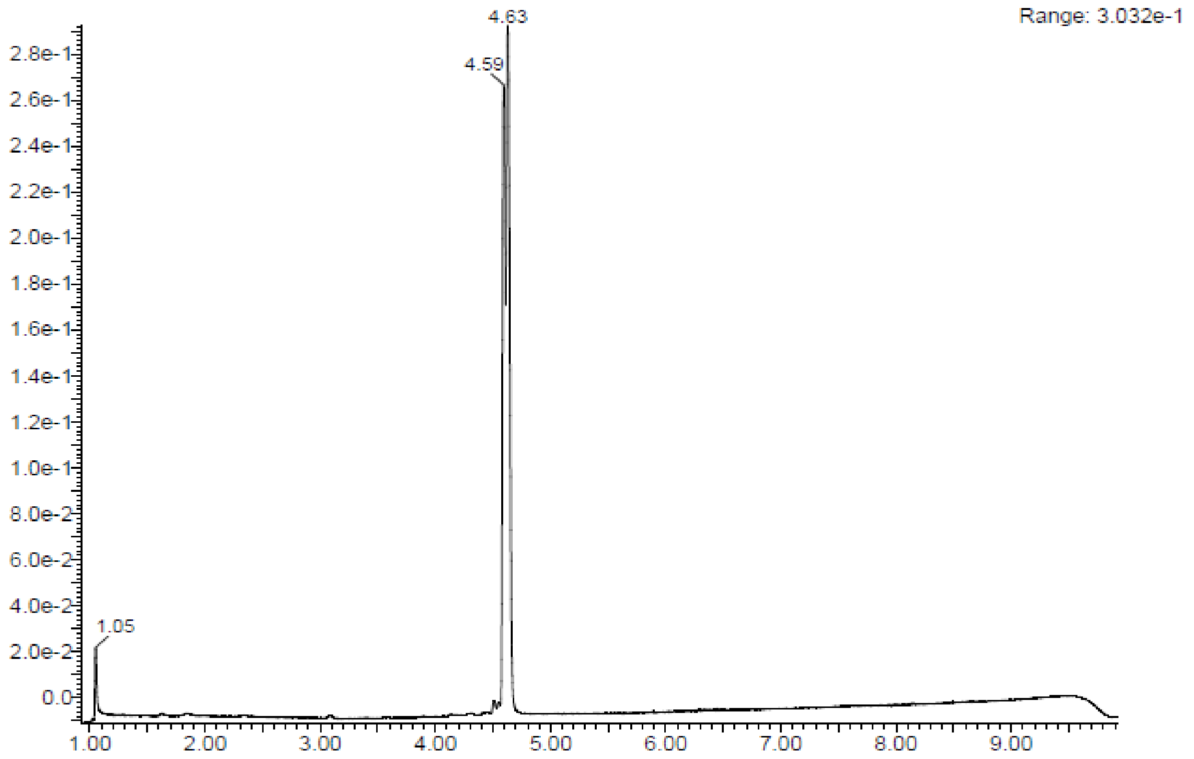

Supplementary Figure S182: Reverse-phase UPLC **ONS5** after digestion with exonuclease I (UV absorbance at 260 nm vs time in min).

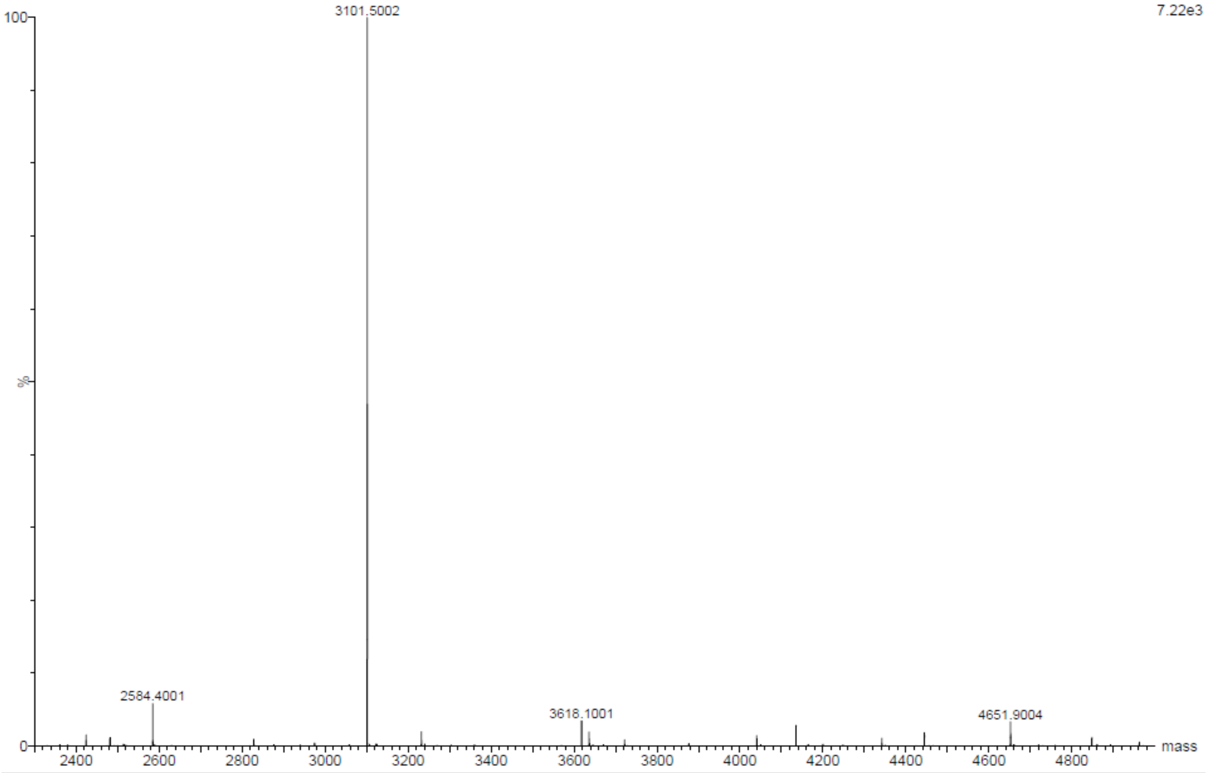

Supplementary Figure S183: Mass spectrum (ES-) of **ONS5** after digestion with exonuclease I. Required **3101.12** Da, found **3101.50** Da. y-axis = relative intensity (%), x-axis = mass in Da.

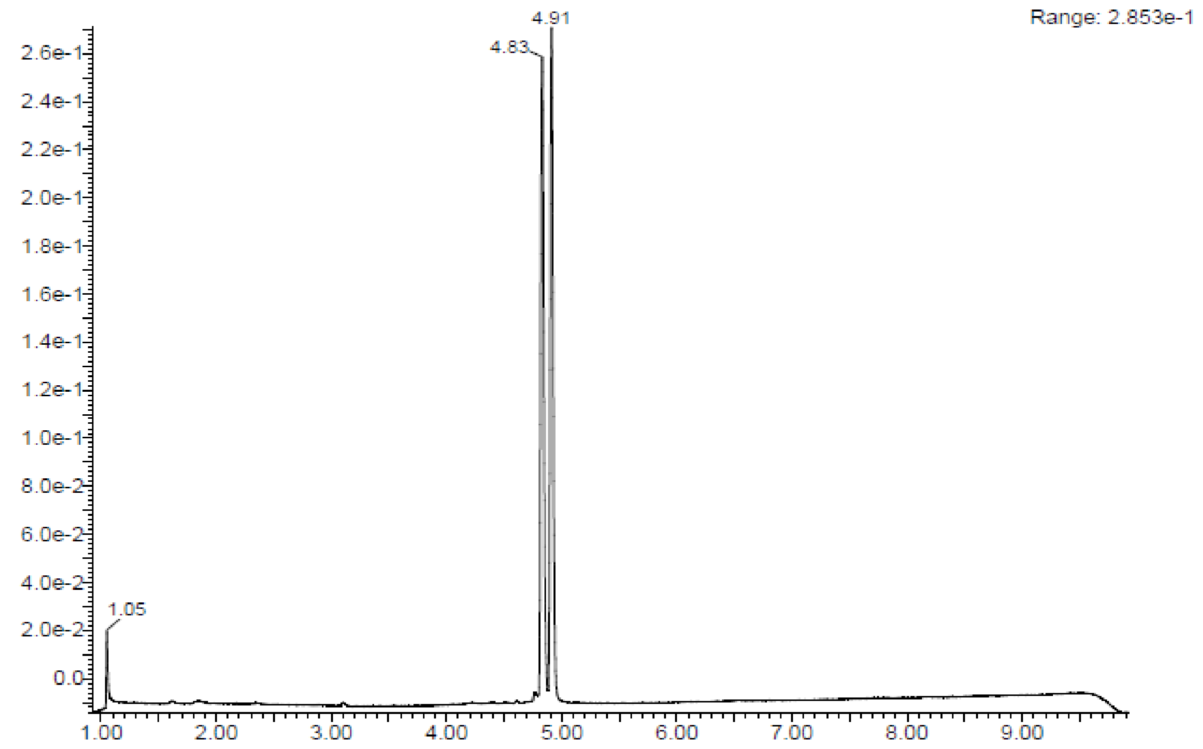

Supplementary Figure S184: Reverse-phase UPLC **ONS6** after digestion with exonuclease I (UV absorbance at 260 nm vs time in min).

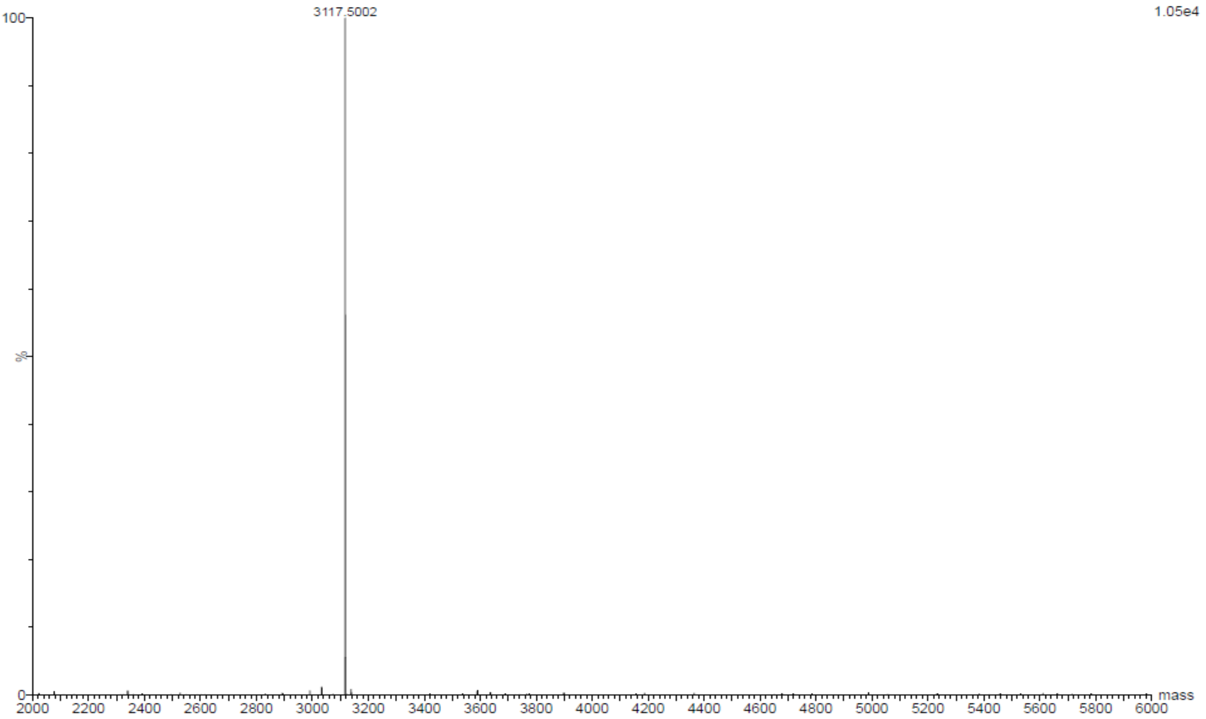

Supplementary Figure S185: Mass spectrum (ES-) of **ONS6** after digestion with exonuclease I. Required **3117.18** Da, found **3117.50** Da. y-axis = relative intensity (%), x-axis = mass in Da.

7.0 Trityl monitor readings

ON8 MeOPr	CCT CTT ACC TCA GTT ACA	**6.MeOPr**	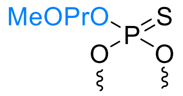

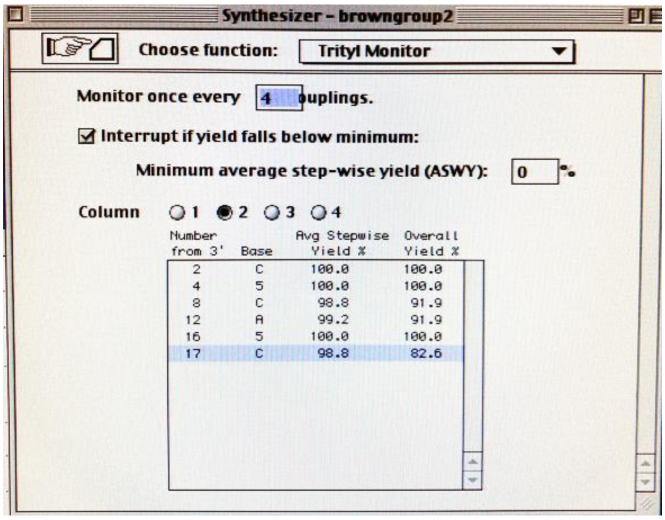

Supplementary Figure S186: Trityl Monitor Readings of **ON8** obtained from the Solid Phase Synthesizer using the of the monomer **14**.

ON15 iPr	CCT CUT ACC TCA GUT ACA	**4.^{i}Pr**	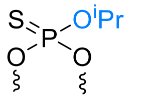

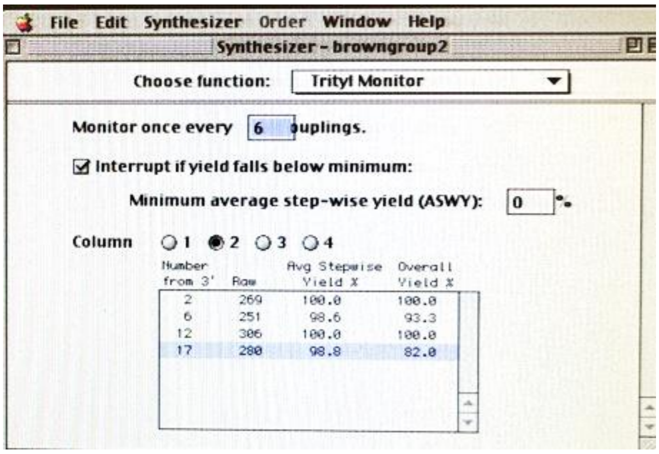

Supplementary Figure S187: Trityl Monitor Readings of **ON15** obtained from the Solid Phase Synthesizer using the of the monomer **15**.

ON16 iPr	CCT CTT ACC TCA GTT ACA	**6.^{i}Pr**	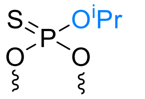

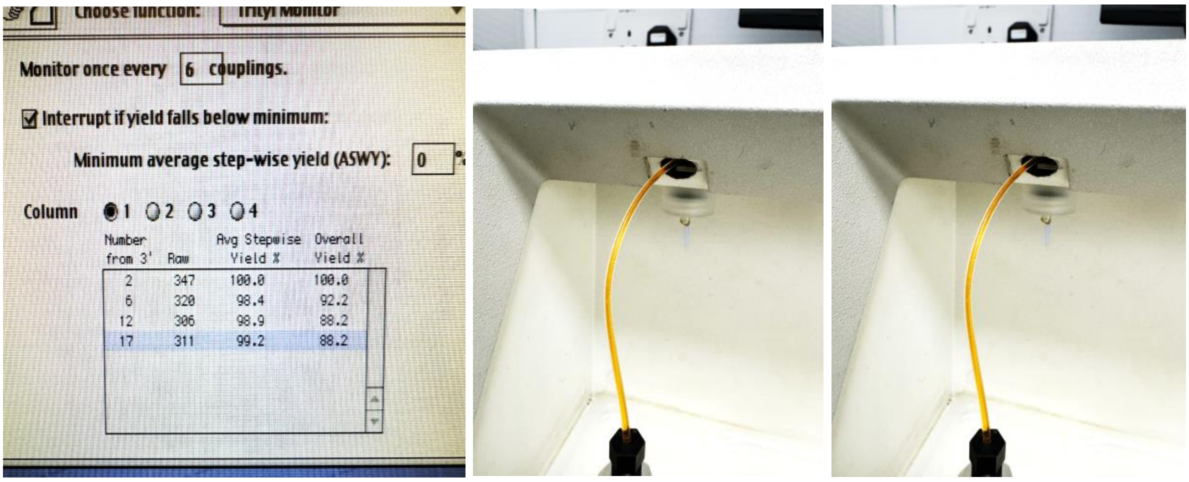

Supplementary Figure S188: *Left*: Trityl Monitor Readings of **ON16** during the Solid Phase Synthesis using the of the monomer **15**. *Middle*: colour of DMT removal after addition of first modified monomer **15**. Right: Colour of the DMT removal after addition of last modified monomer **15**.

ON24 LNA control	CCT CTT ACC TCA GTT ACA	**Phosphorothiate**	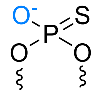

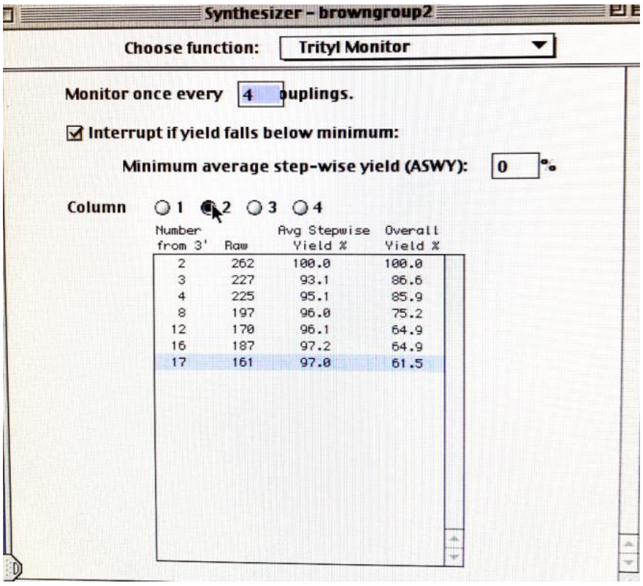

Supplementary Figure S189: Trityl Monitor Readings of **ON24** obtained from the Solid Phase Synthesizer using the of the monomer **16**.

ON33 THP	CCT CUT ACC TCA GUT ACA	**7.THP**	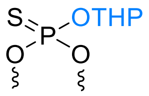

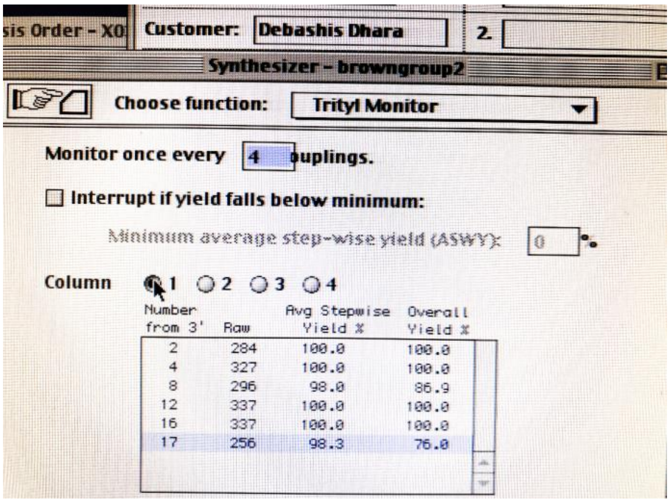

Supplementary Figure S190: Trityl Monitor Readings of **ON33** obtained from the Solid Phase Synthesizer using monomer **17** and **22**.

ON34 THP	CCT CTT ACC TCA GTT ACA	**9.THP**	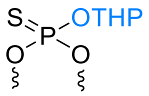

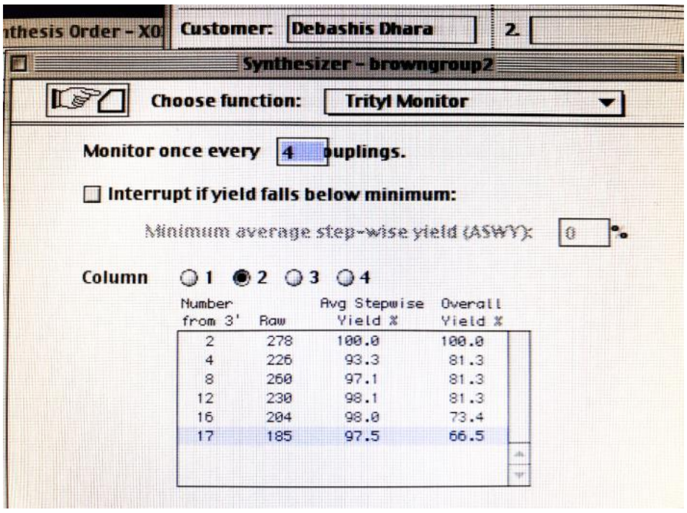

Supplementary Figure S191: Trityl Monitor Readings of **ON34** obtained from the Solid Phase Synthesizer using monomer **17** and **22**.

ON38 C16	CCU CUU ACC UCA GUU ACA	**3.$C_{16}H_{33}$**	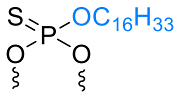

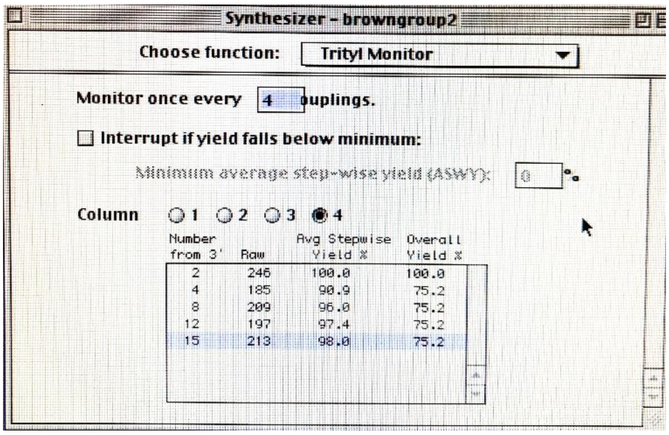

Supplementary Figure S192: Trityl Monitor Readings of **ON38** obtained from the Solid Phase Synthesizer using monomer **23**.

ON41 Hexynyl	CCU CUU ACC UCA GUU ACA	**3.Hexynyl**	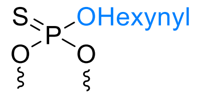

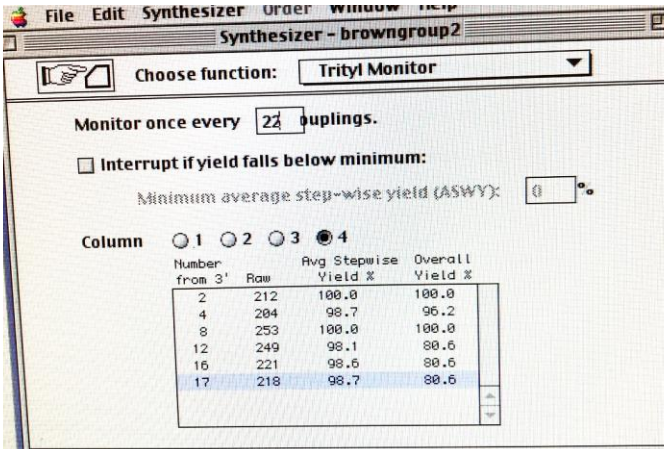

Supplementary Figure S193: Trityl Monitor Readings of **ON41** obtained from the Solid Phase Synthesizer using monomer **24**.

8.0 NMR spectra of novel compounds

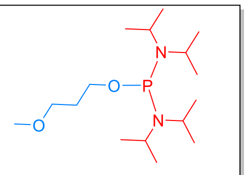

Chemical Formula: $C_{16}H_{37}N_2O_2P$
Exact Mass: 320.26
7

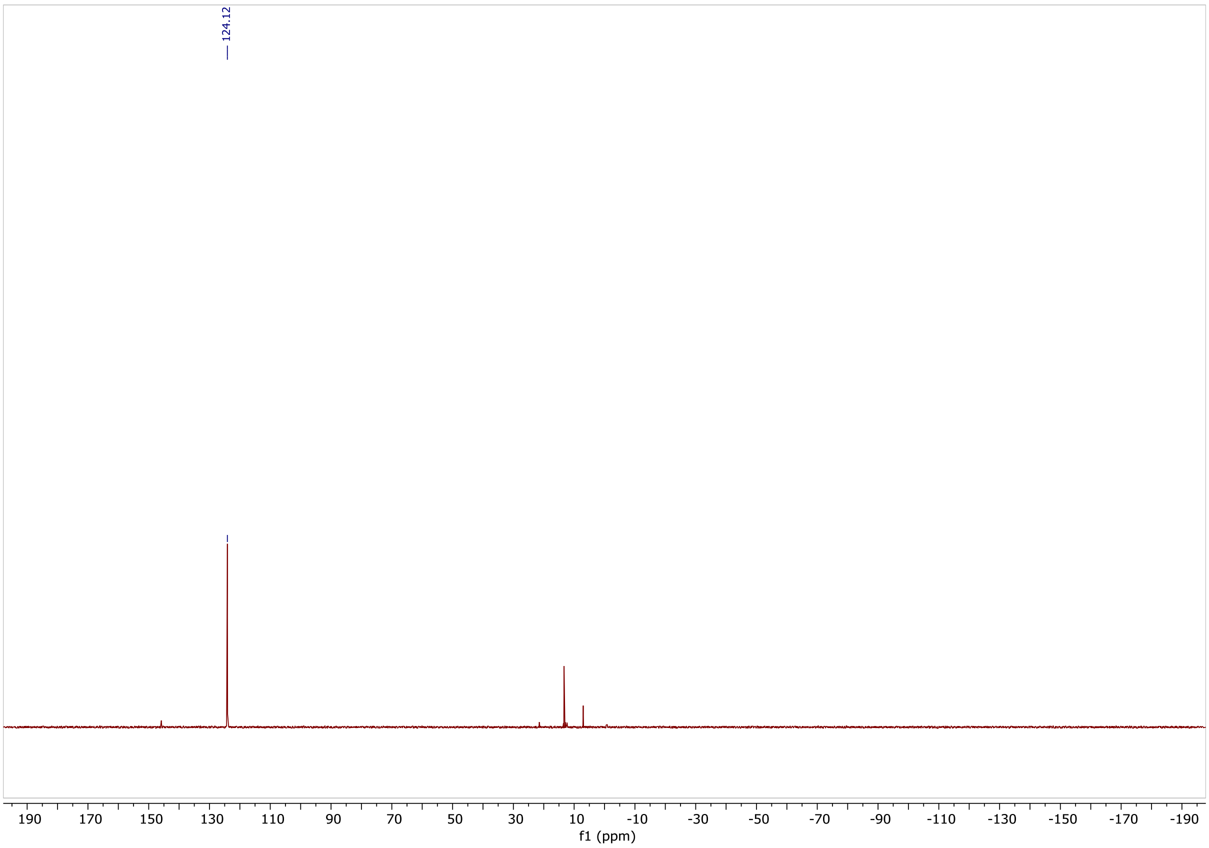

Supplementary Figure S194: ^{31}P NMR (162 MHz, $CDCl_3$) spectrum of **crude 7.**

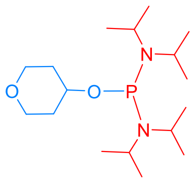

Chemical Formula: $C_{17}H_{37}N_2O_2P$
Exact Mass: 332.2593
10

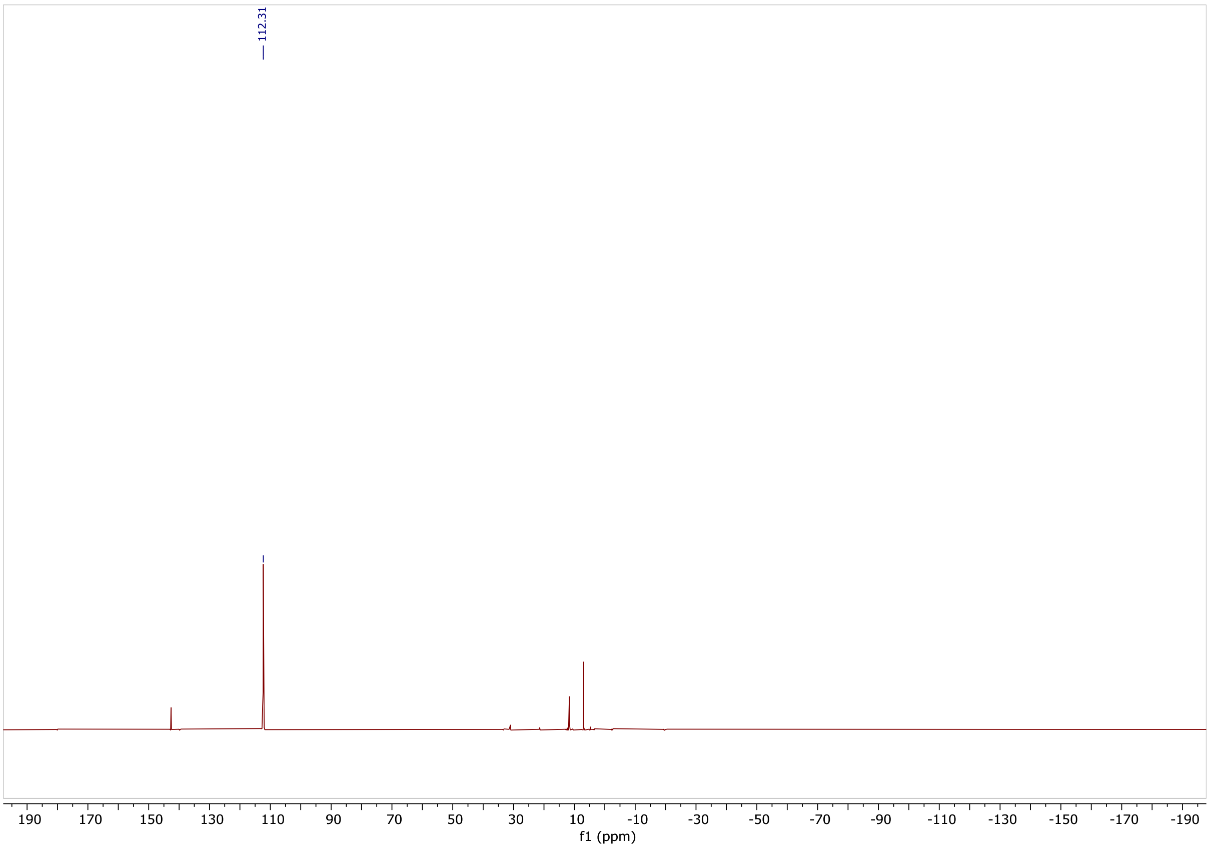

Supplementary Figure S195: ^{31}P NMR (162 MHz, $CDCl_3$) spectrum of **crude 10.**

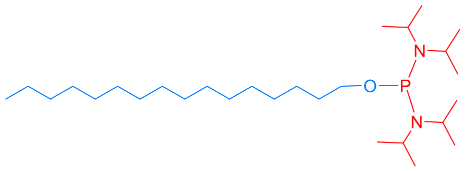

Chemical Formula: $C_{28}H_{61}N_2OP$
Exact Mass: 472.4522

11

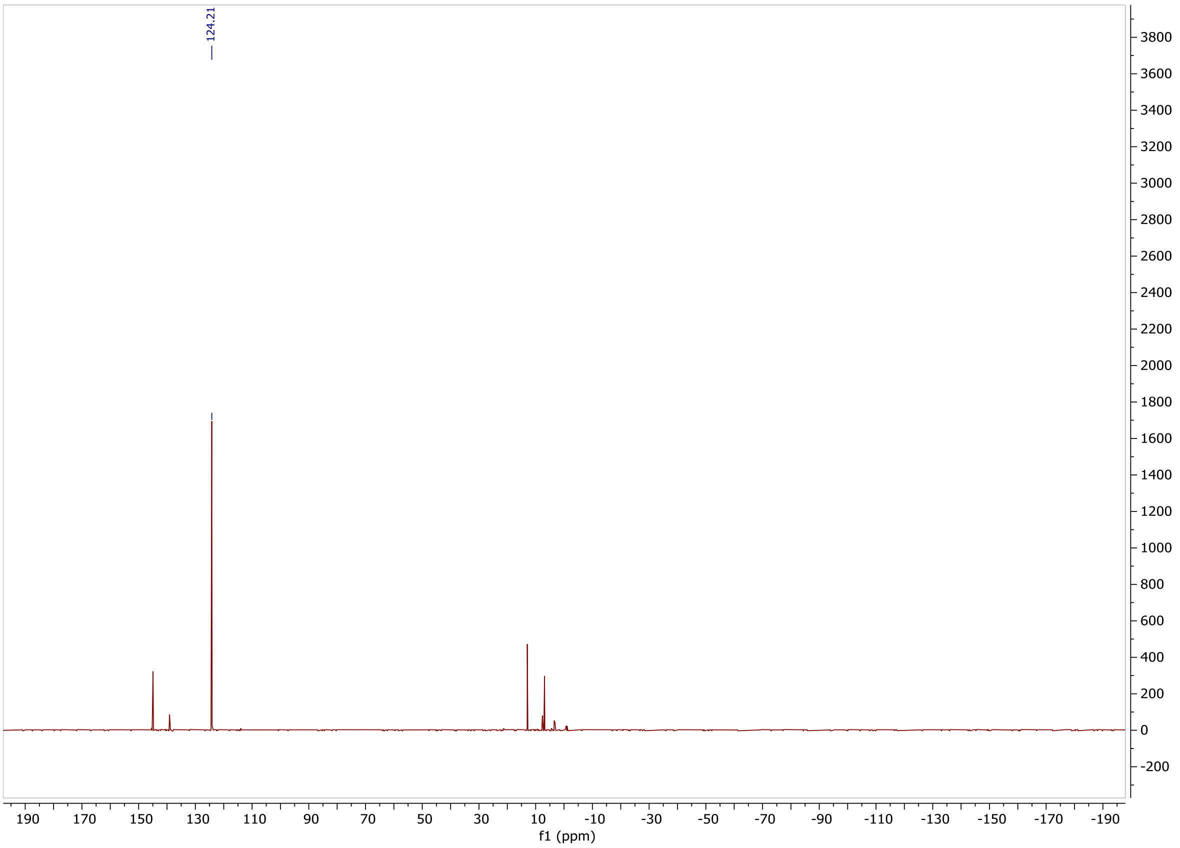

Supplementary Figure S196: ^{31}P NMR (162 MHz, $CDCl_3$) spectrum of **crude 11.**

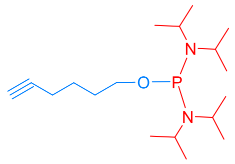

Chemical Formula: $C_{18}H_{37}N_2OP$
Exact Mass: 328.2644
12

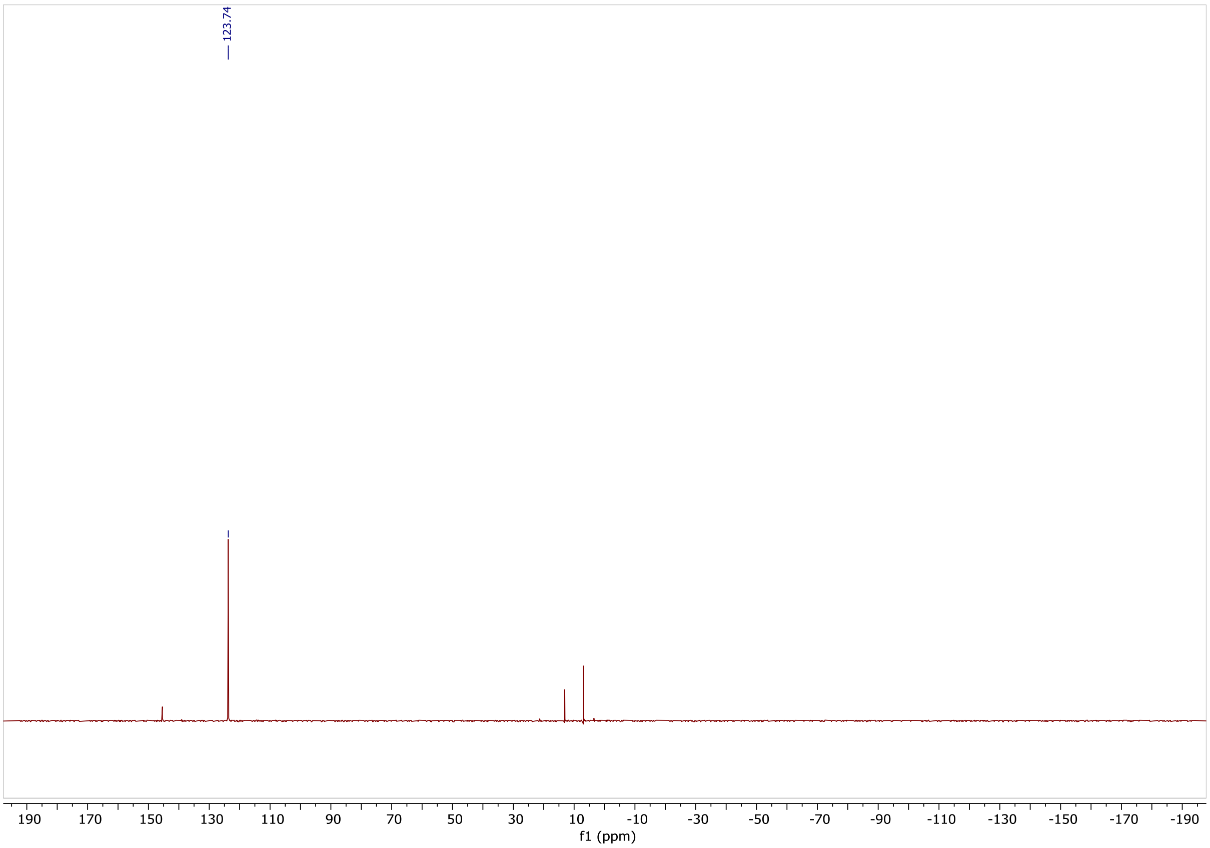

Supplementary Figure S197: ^{31}P NMR (162 MHz, $CDCl_3$) spectrum of **crude 12.**

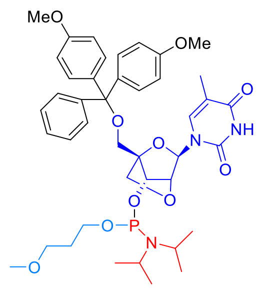

Chemical Formula: $C_{42}H_{54}N_3O_{10}P$
Exact Mass: 791.3547

14

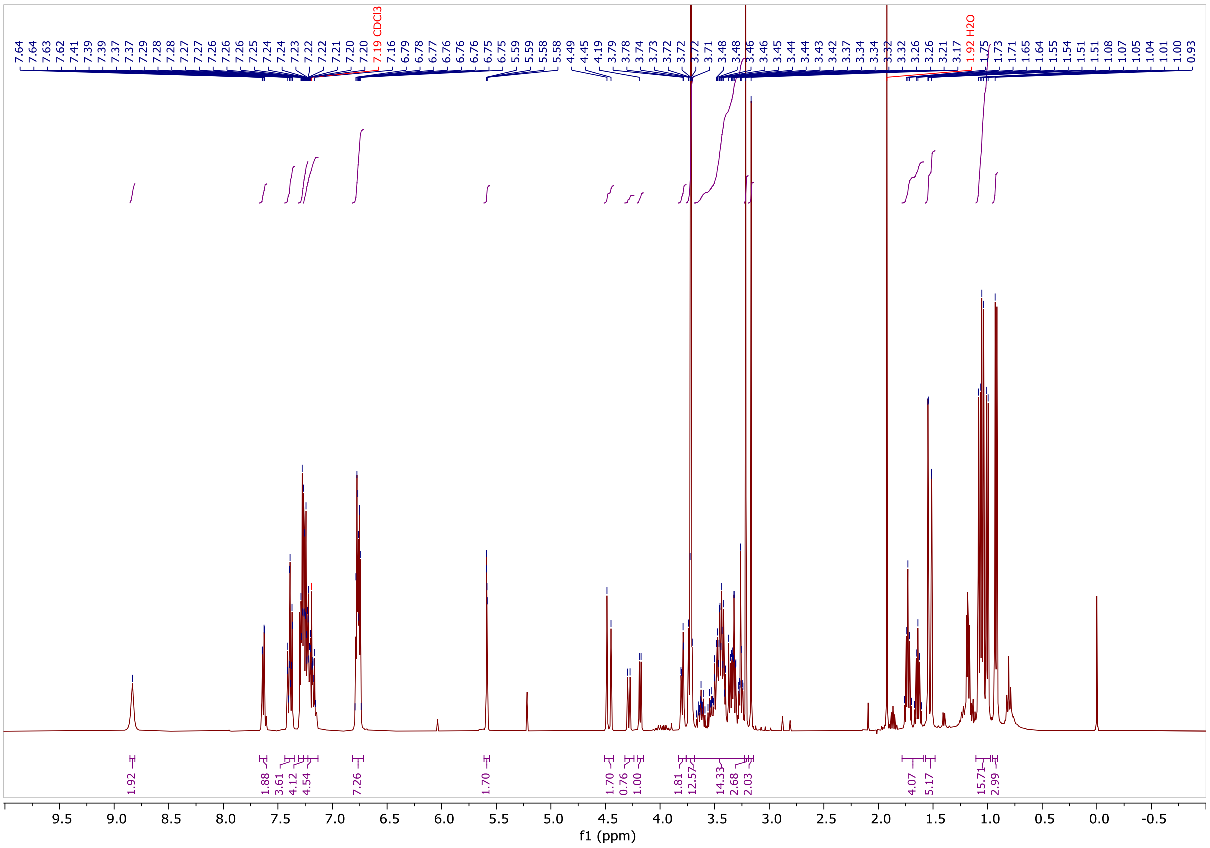

+

Supplementary Figure S198: ^{1}H NMR (400 MHz, $CDCl_3$) spectrum of **14**.

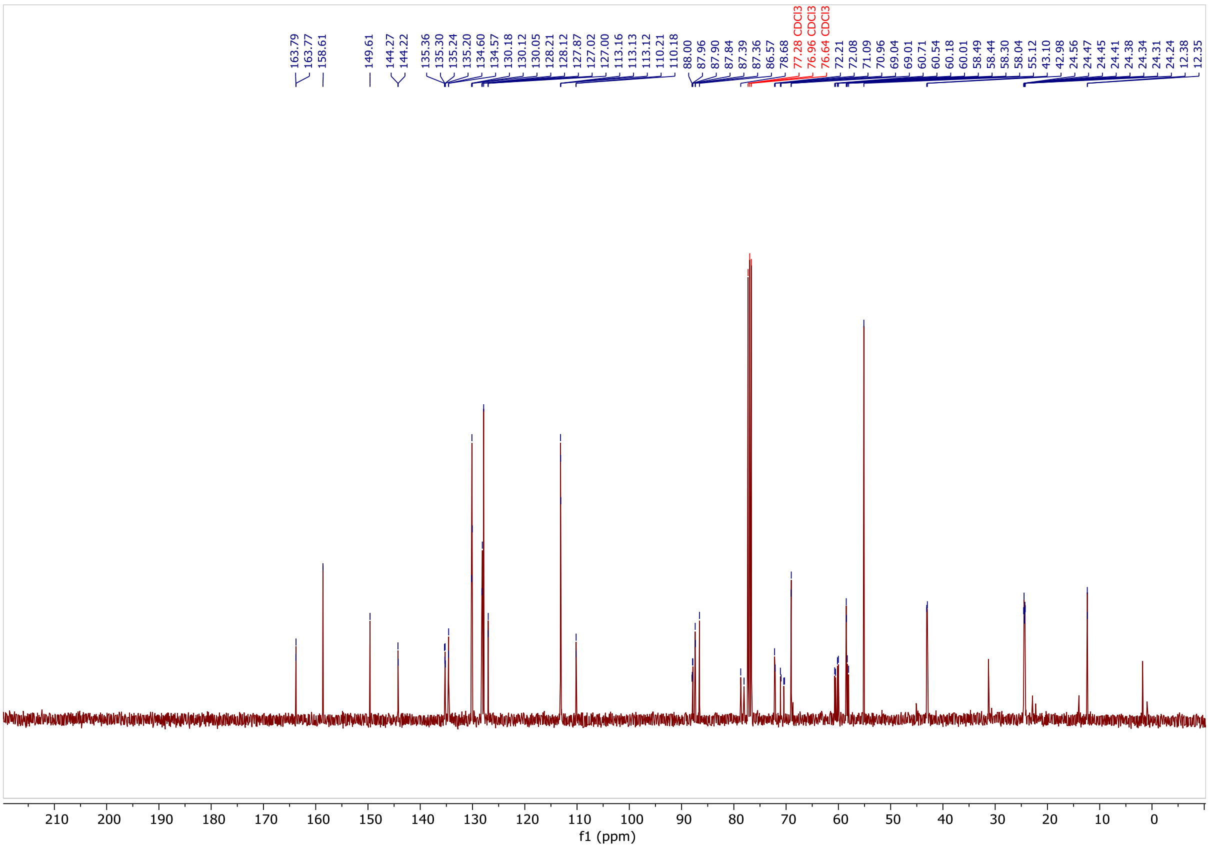

Supplementary Figure S199: $^{13}C\{^{1}H\}$ NMR (101 MHz, $CDCl_3$) spectrum of **14**.

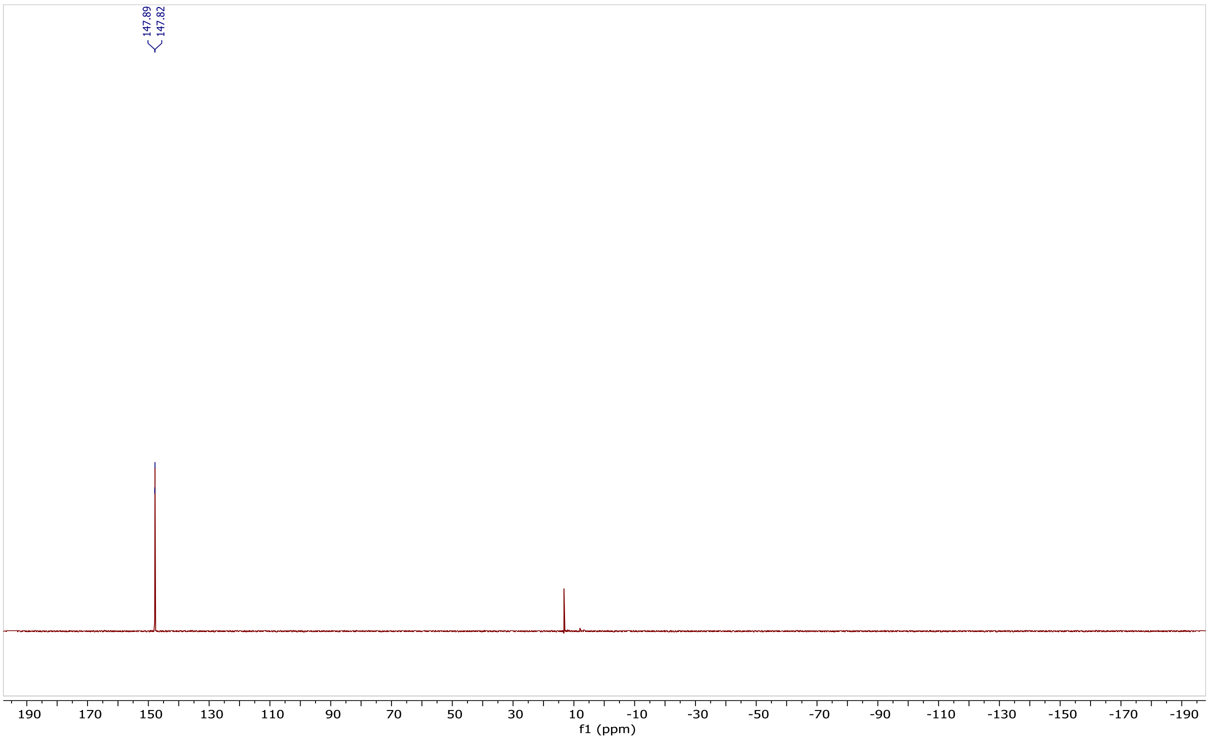

Supplementary Figure S200: ^{31}P NMR (162 MHz, $CDCl_3$) spectrum of **14.**

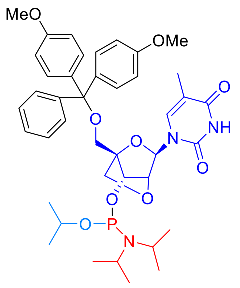

15

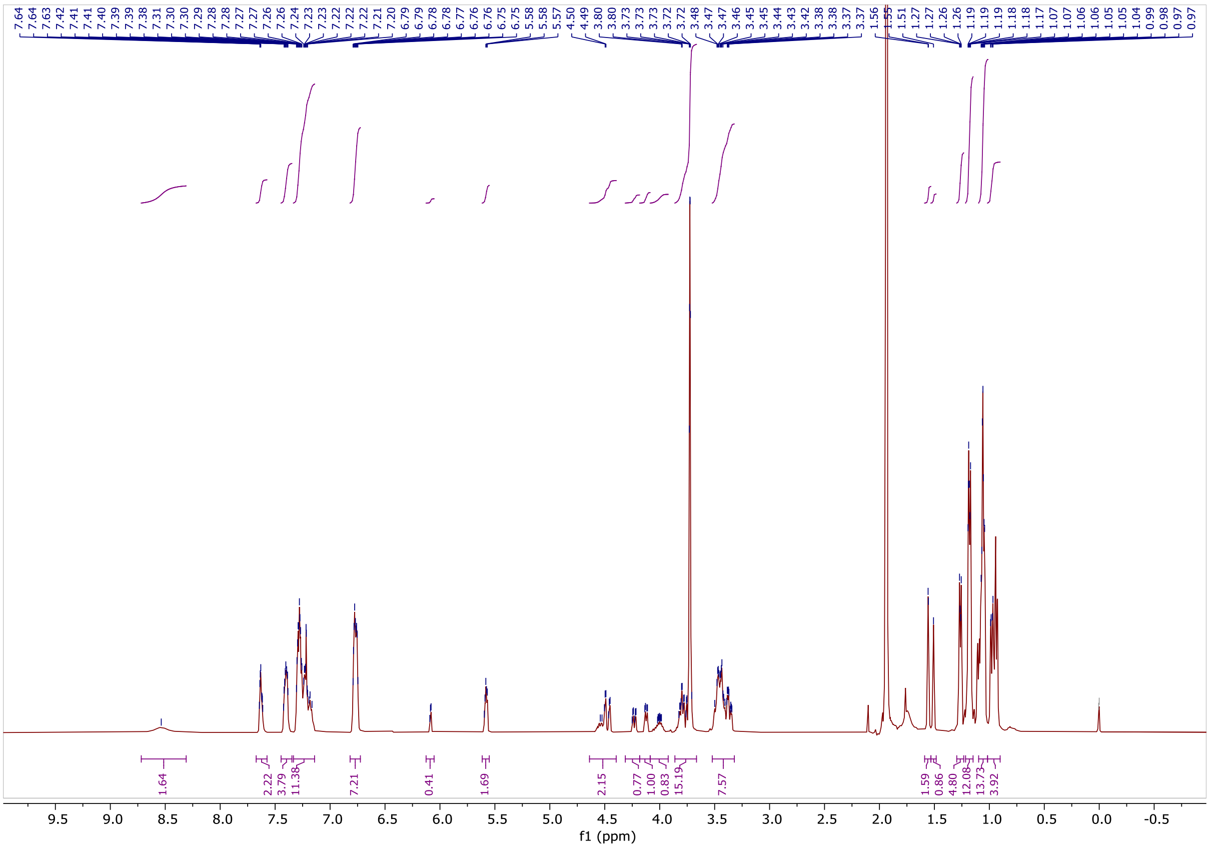

Supplementary Figure S201: ^{1}H NMR (400 MHz, $CDCl_3$) spectrum of **15**.

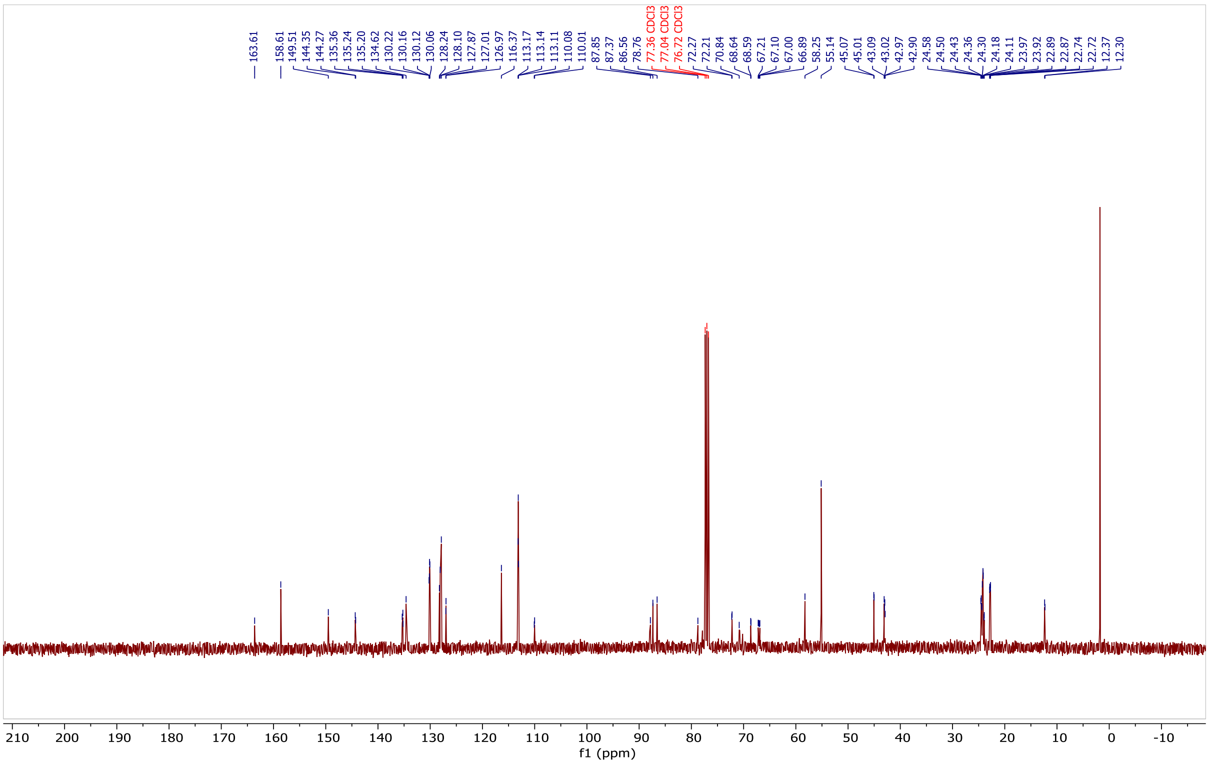

Supplementary Figure S202: $^{13}C\{^{1}H\}$ NMR (101 MHz, $CDCl_3$) spectrum of **15**.

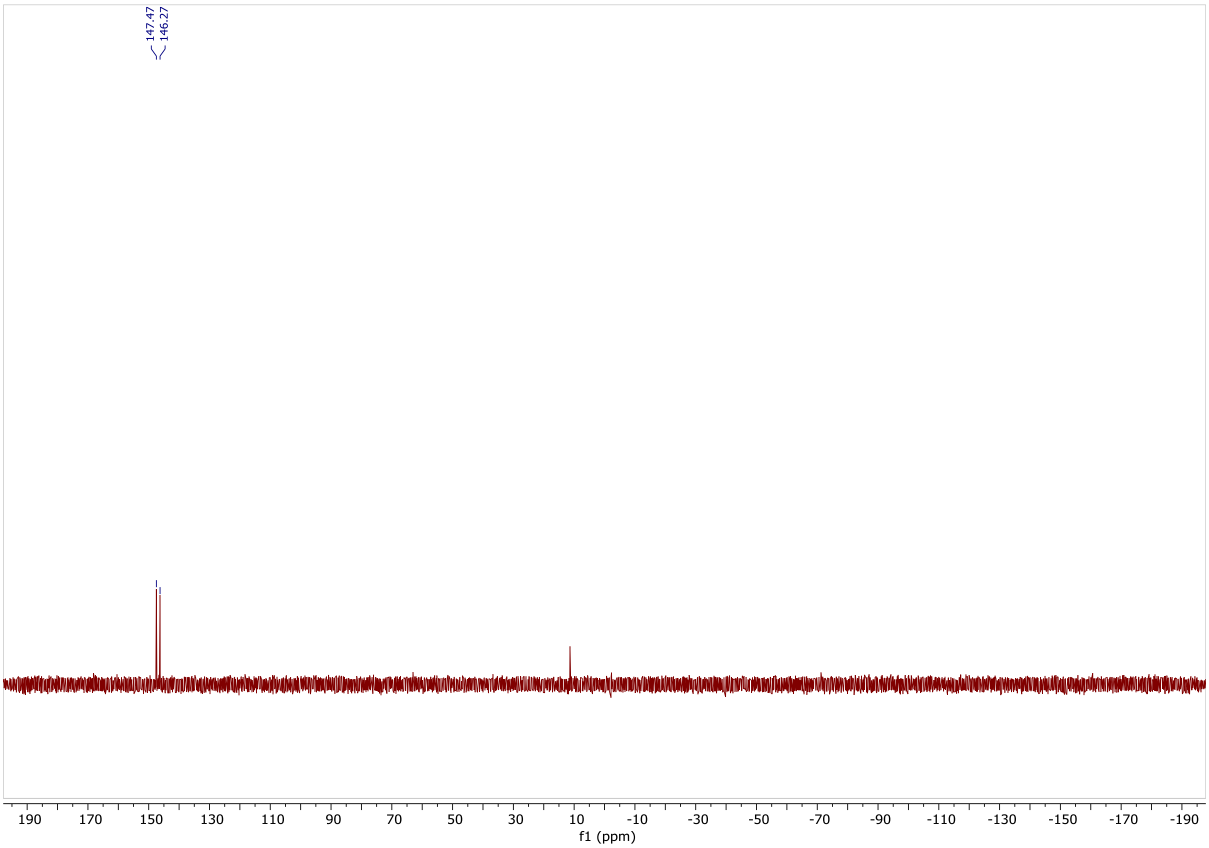

Supplementary Figure S203: ^{31}P NMR (162 MHz, $CDCl_3$) Spectrum of **15**.

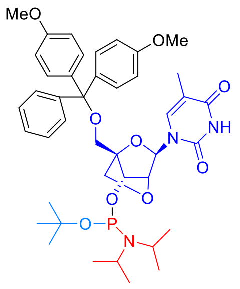

16

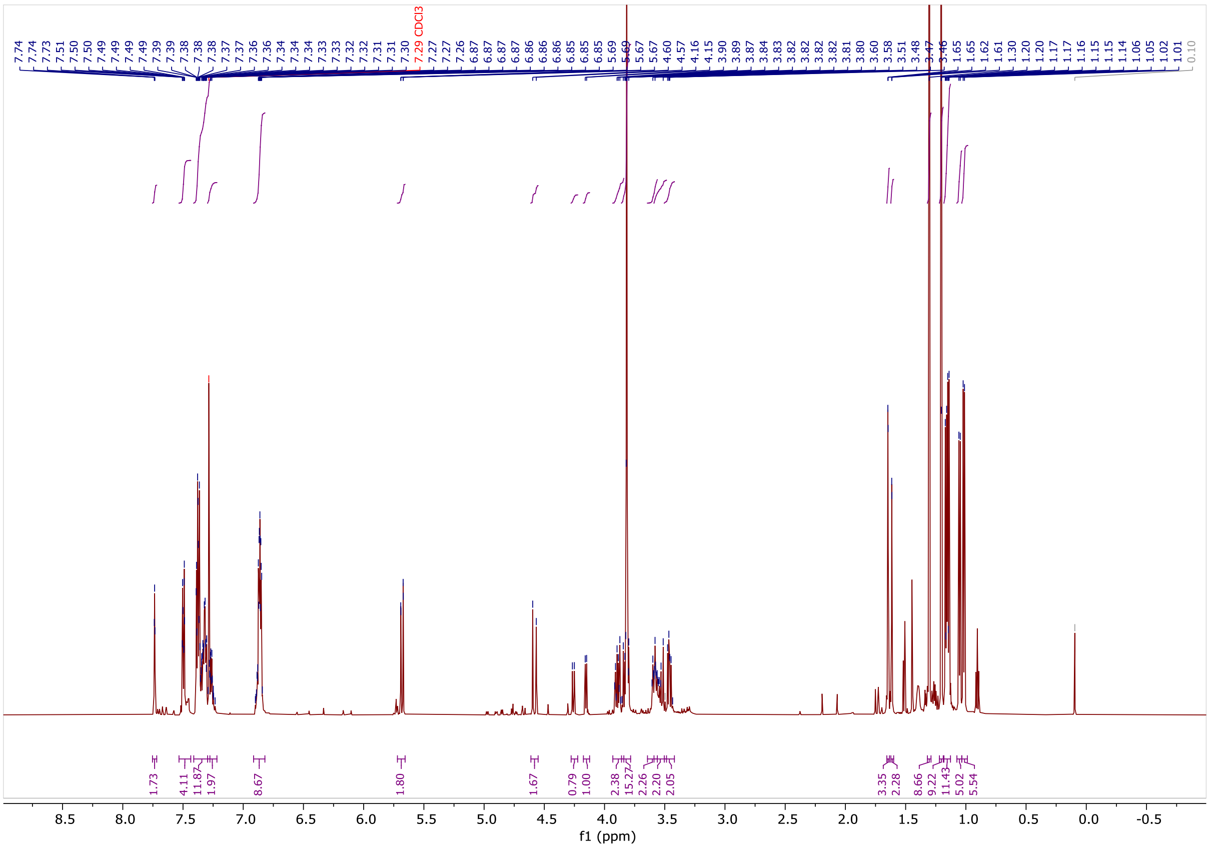

Supplementary Figure S204: ^{1}H NMR (600 MHz, $CDCl_3$) spectrum of **16**.

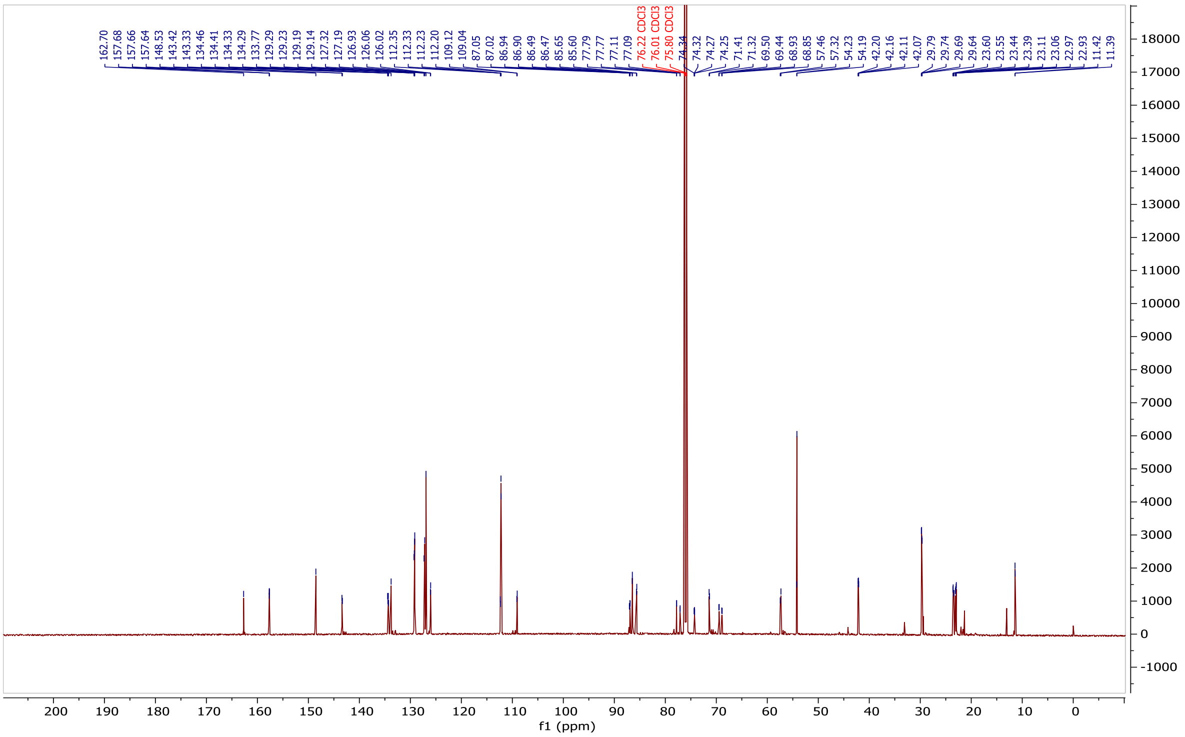

Supplementary Figure S205: $^{13}C\{^{1}H\}$ NMR (151 MHz, $CDCl_3$) spectrum of **16**.

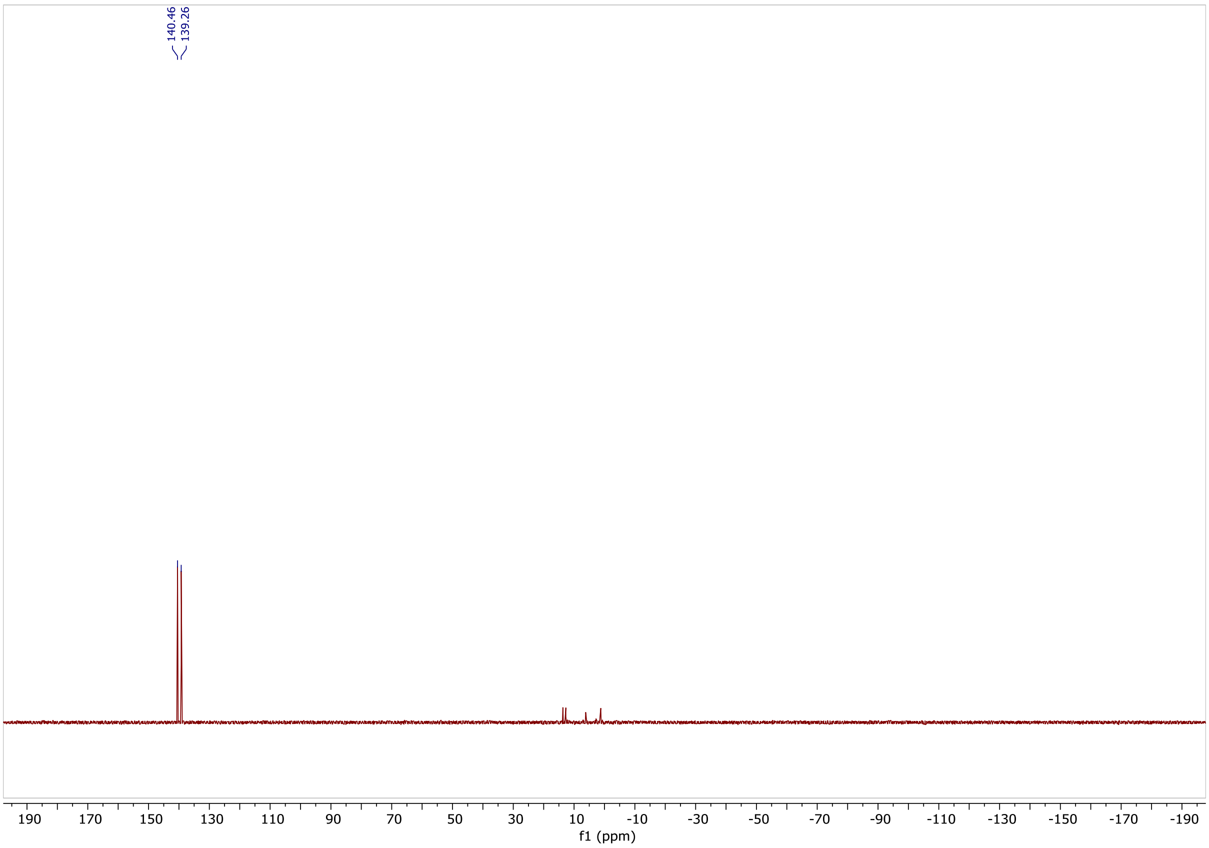

Supplementary Figure S206: ^{31}P NMR (162 MHz, $CDCl_3$) spectrum of **16.**

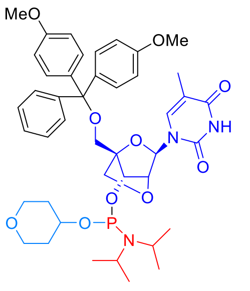

Chemical Formula: $C_{43}H_{54}N_3O_{10}P$
Exact Mass: 803.3547
17

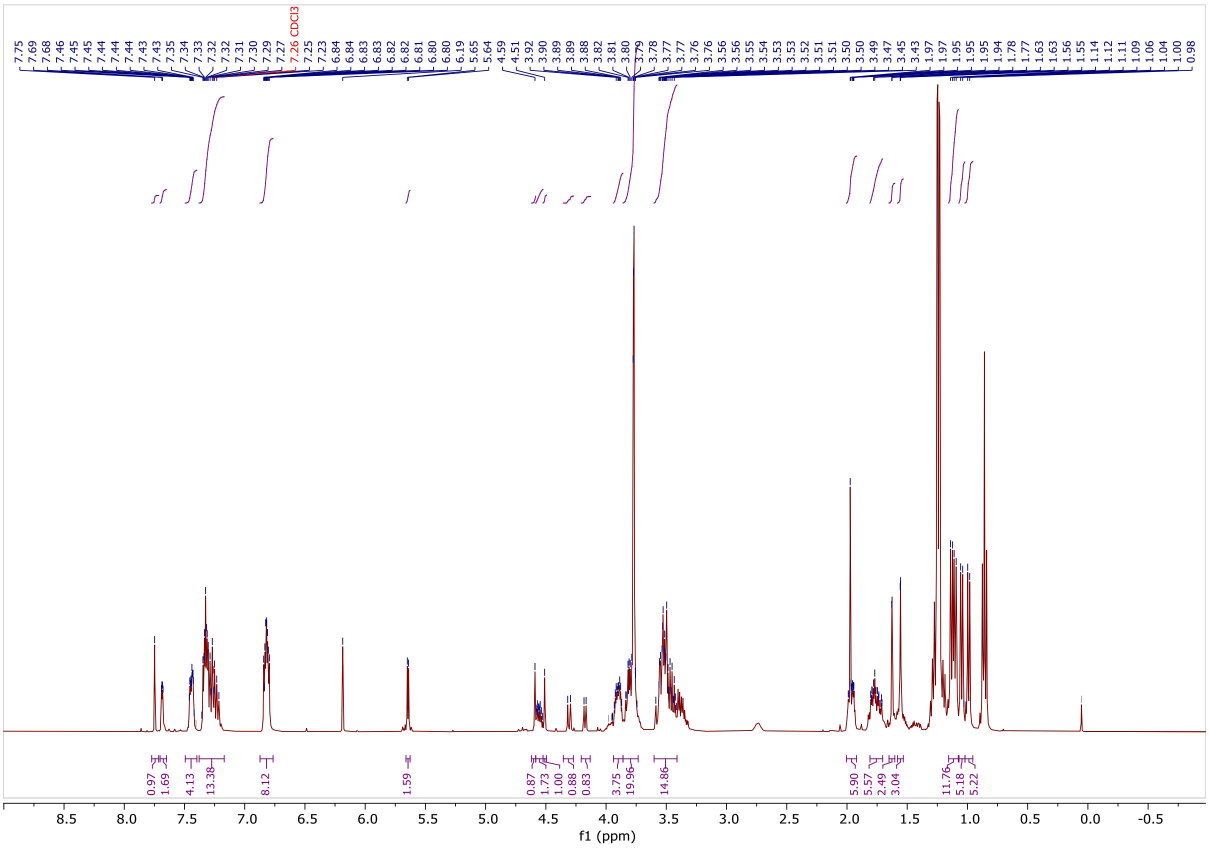

Supplementary Figure S207: ^{1}H NMR (400 MHz, $CDCl_3$) spectrum of **17**.

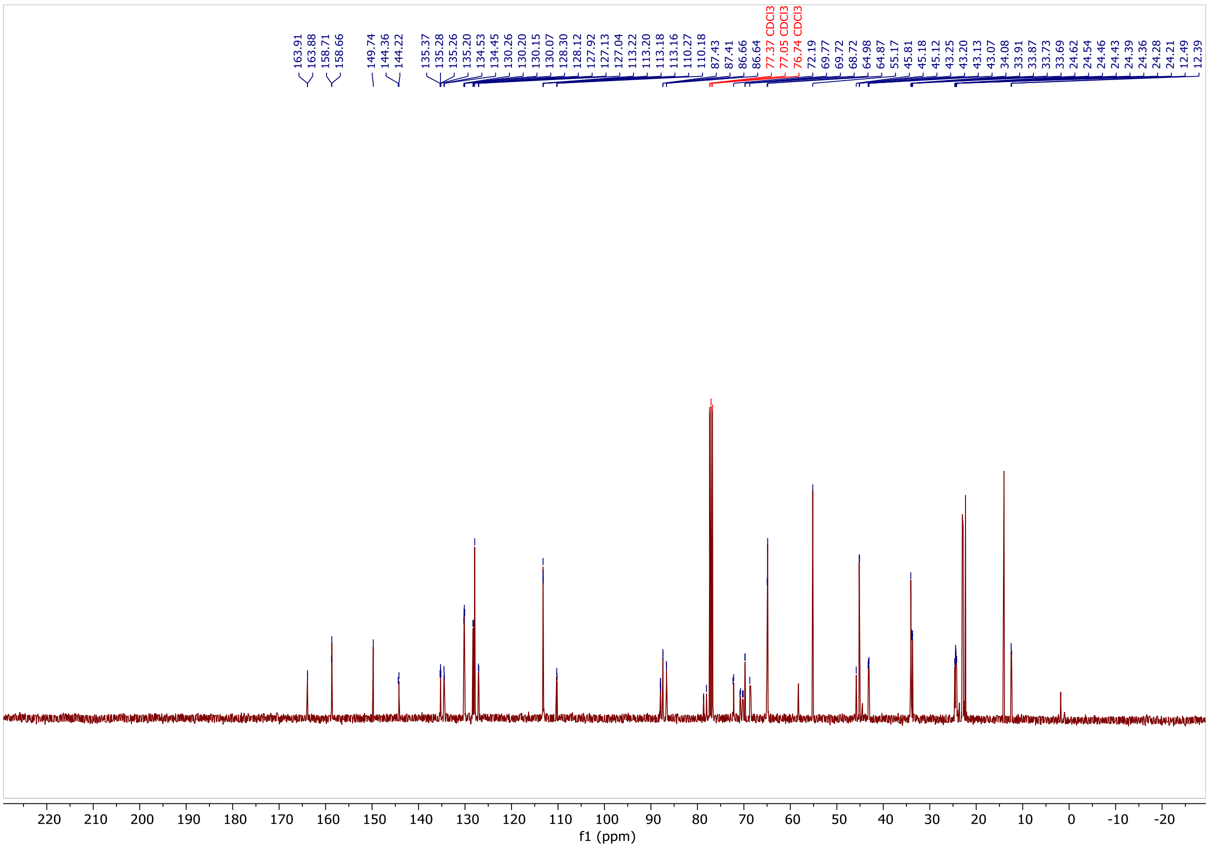

Supplementary Figure S208: $^{13}C\{^{1}H\}$ NMR (101 MHz, $CDCl_3$) spectrum of **17**.

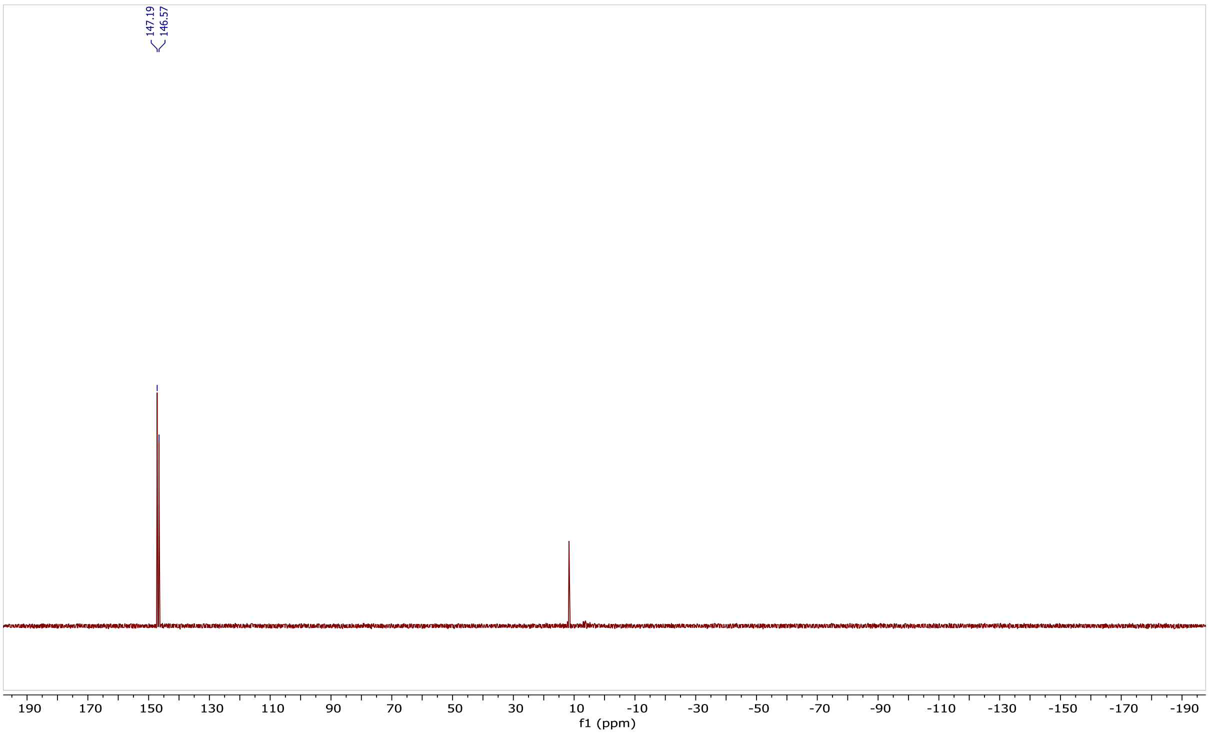

Supplementary Figure S209: ^{31}P NMR (162 MHz, $CDCl_3$) spectrum of **17.**

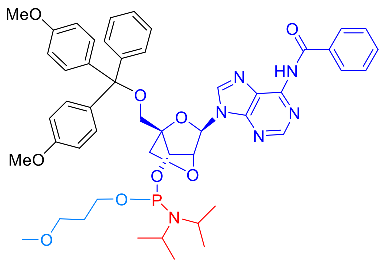

Chemical Formula: $C_{49}H_{57}N_6O_9P$
Exact Mass: 904.3925

19

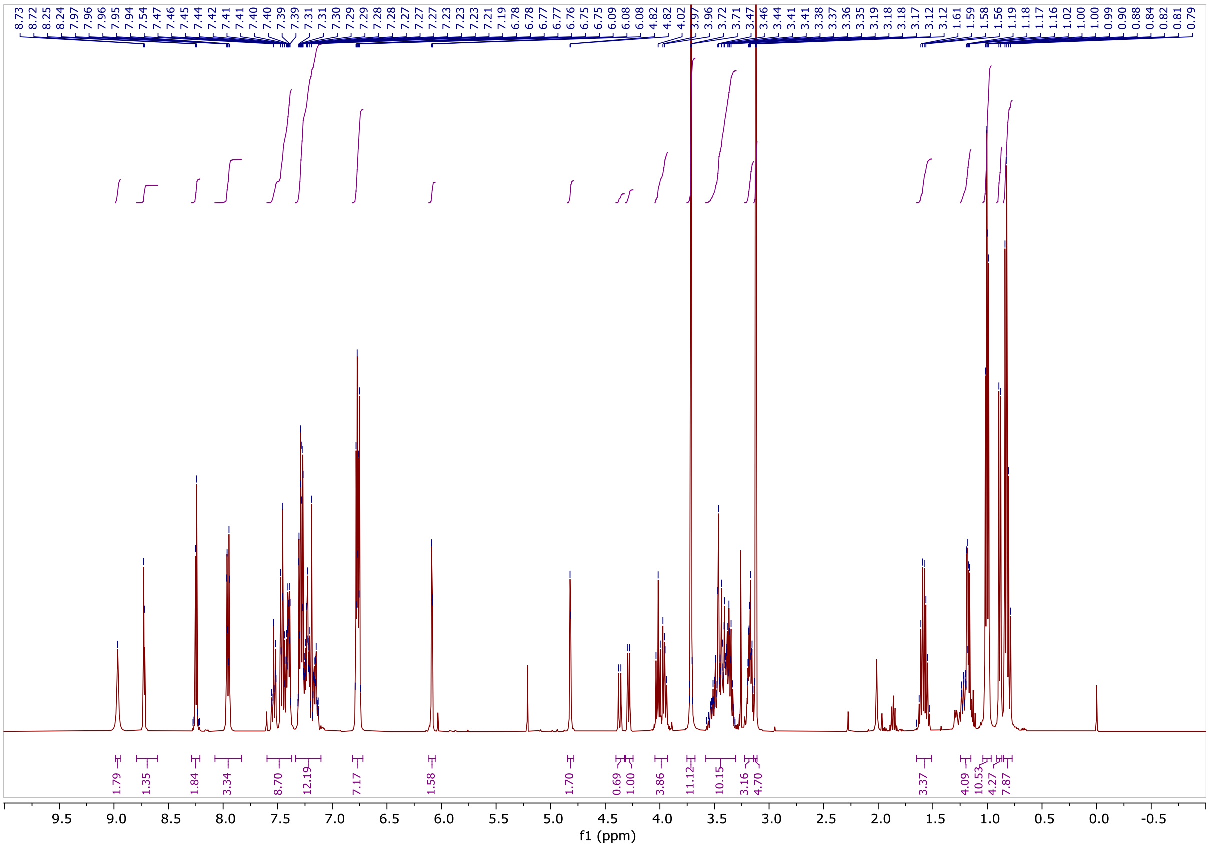

Supplementary Figure S210: ^{1}H NMR (400 MHz, $CDCl_3$) spectrum of **19**.

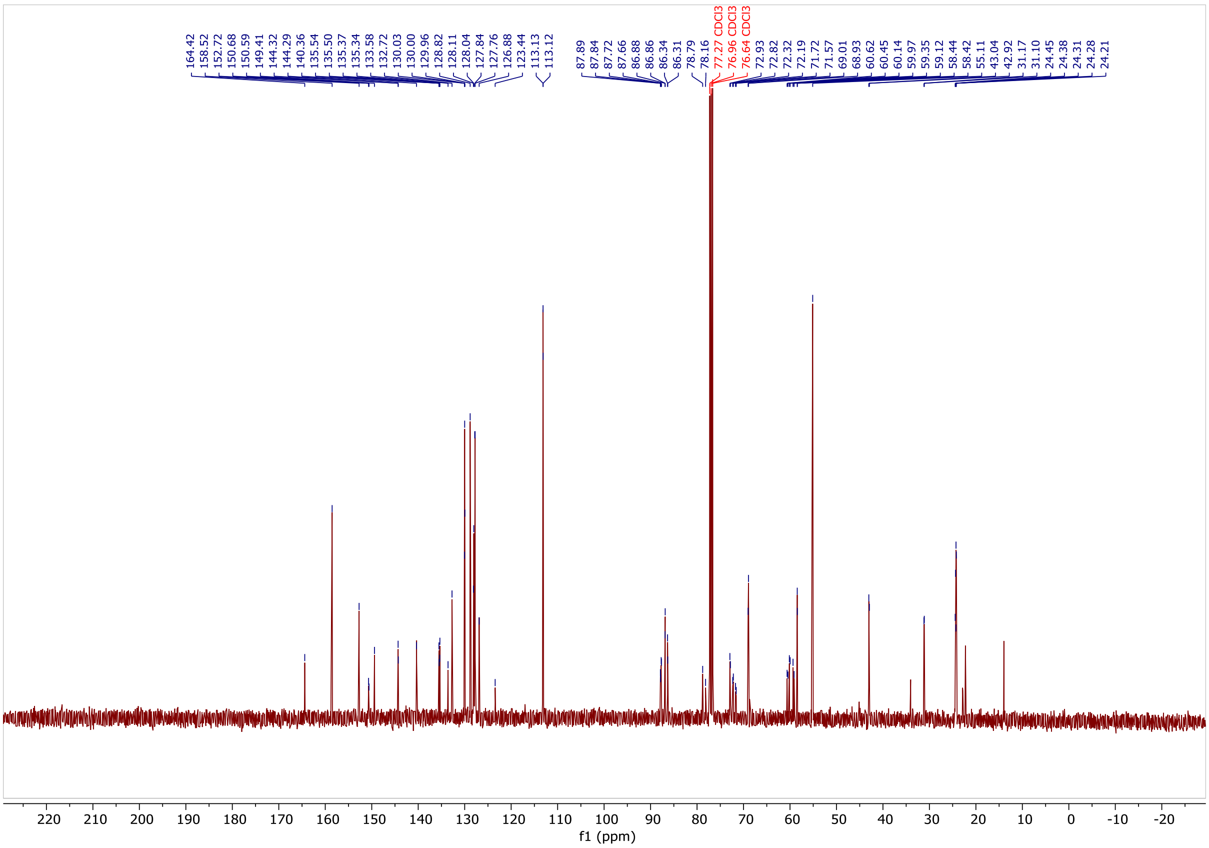

Supplementary Figure S211: $^{13}C\{^{1}H\}$ NMR (101 MHz, $CDCl_3$) spectrum of **19**.

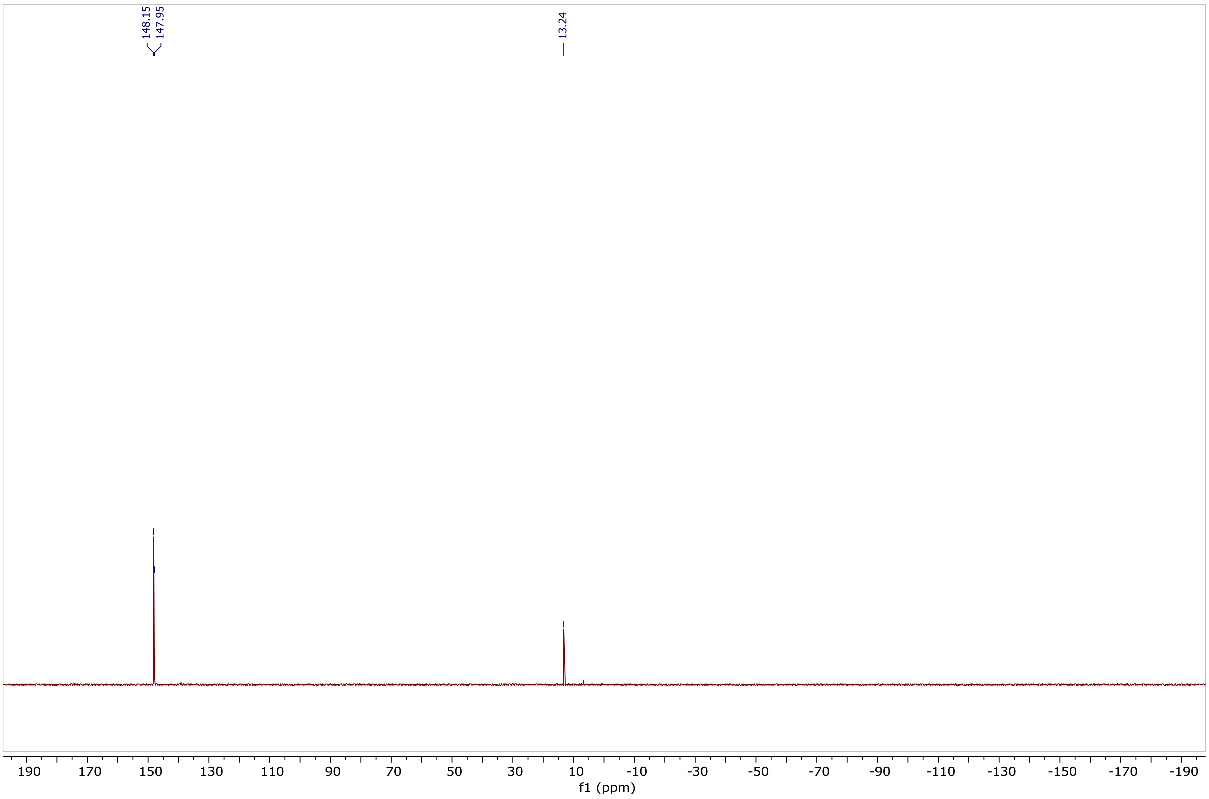

Supplementary Figure S212: ^{31}P NMR (162 MHz, $CDCl_3$) spectrum of **19.**

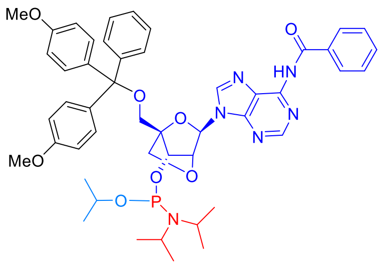

Chemical Formula: $C_{48}H_{55}N_6O_8P$
Exact Mass: 874.3819
20

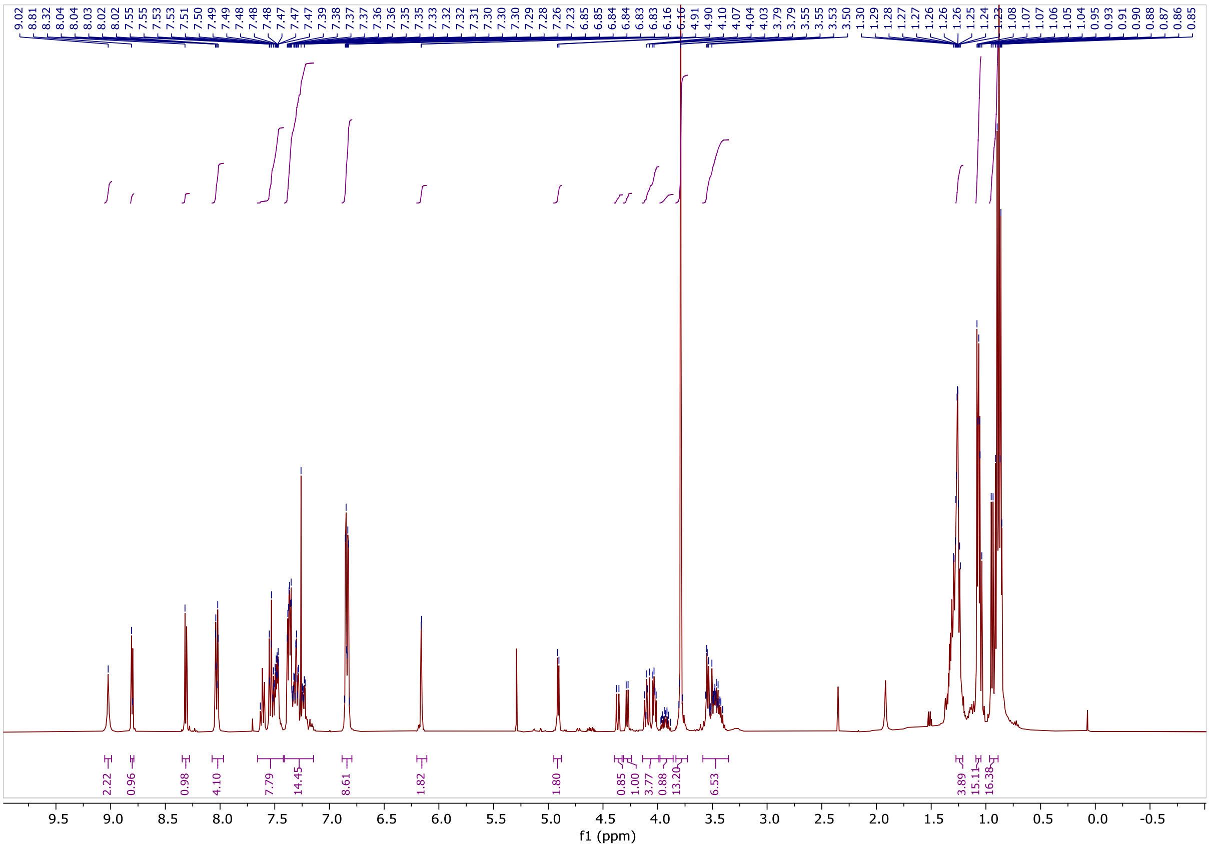

Supplementary Figure S213: ^{1}H NMR (400 MHz, $CDCl_3$) spectrum of **20**.

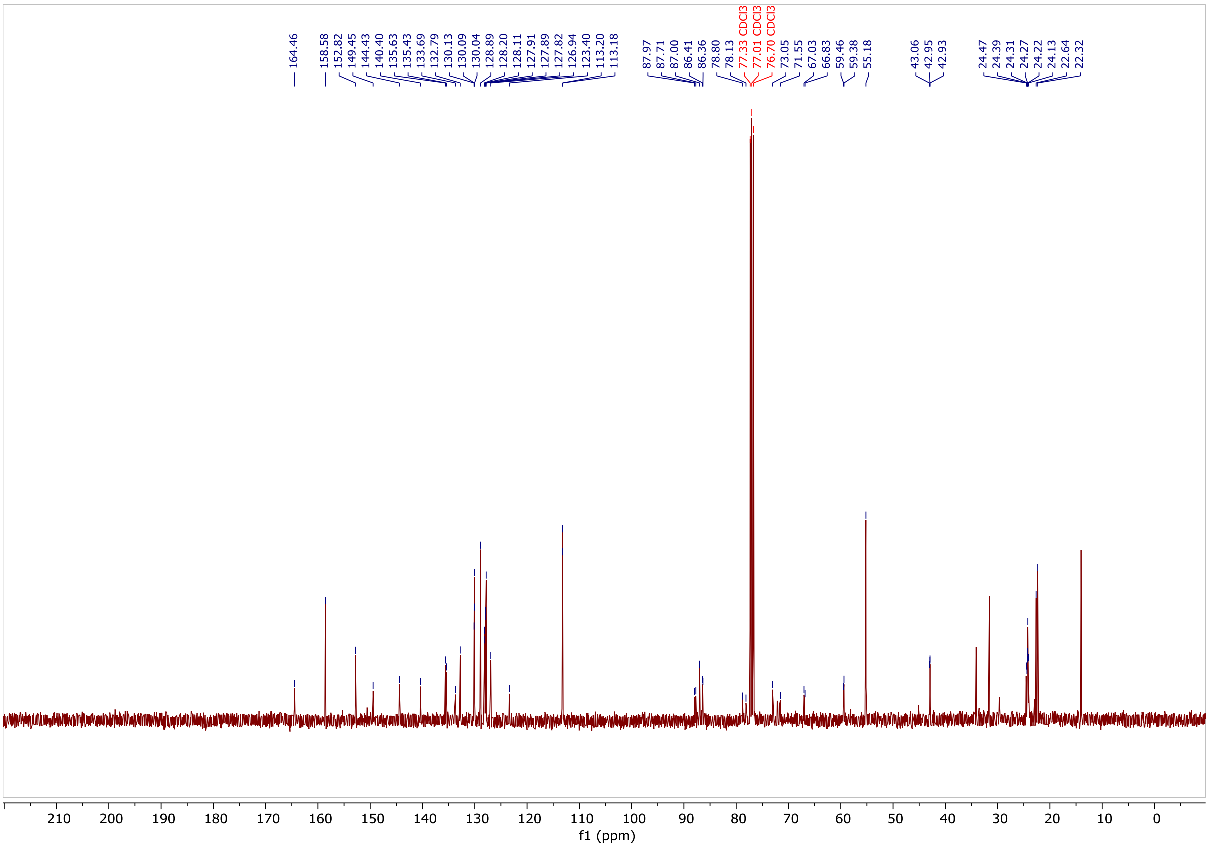

Supplementary Figure S214: $^{13}C\{^{1}H\}$ NMR (101 MHz, $CDCl_3$) spectrum of **20**.

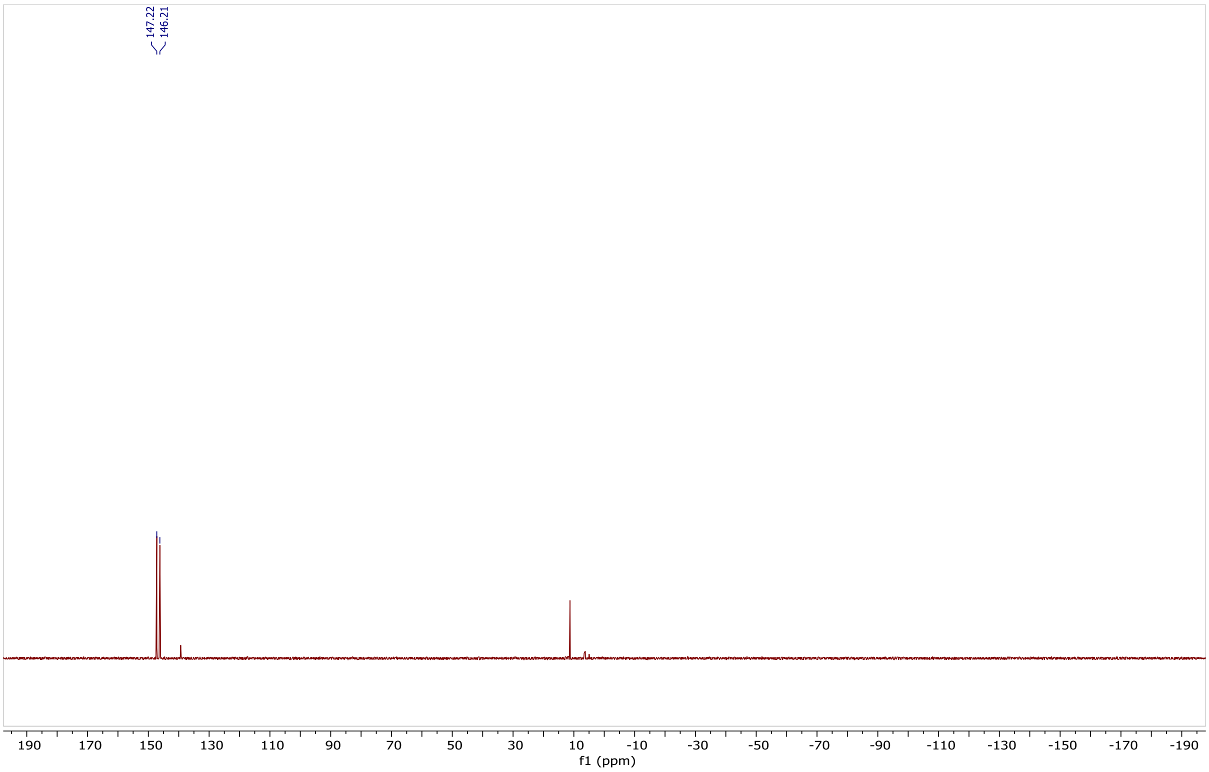

Supplementary Figure S215: ^{31}P NMR (162 MHz, $CDCl_3$) spectrum of **20.**

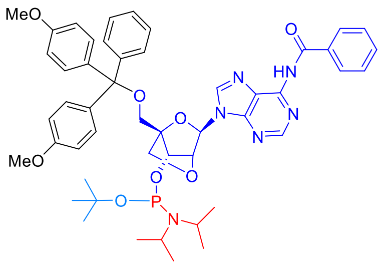

Chemical Formula: $C_{49}H_{57}N_6O_8P$
Exact Mass: 888.3975
21

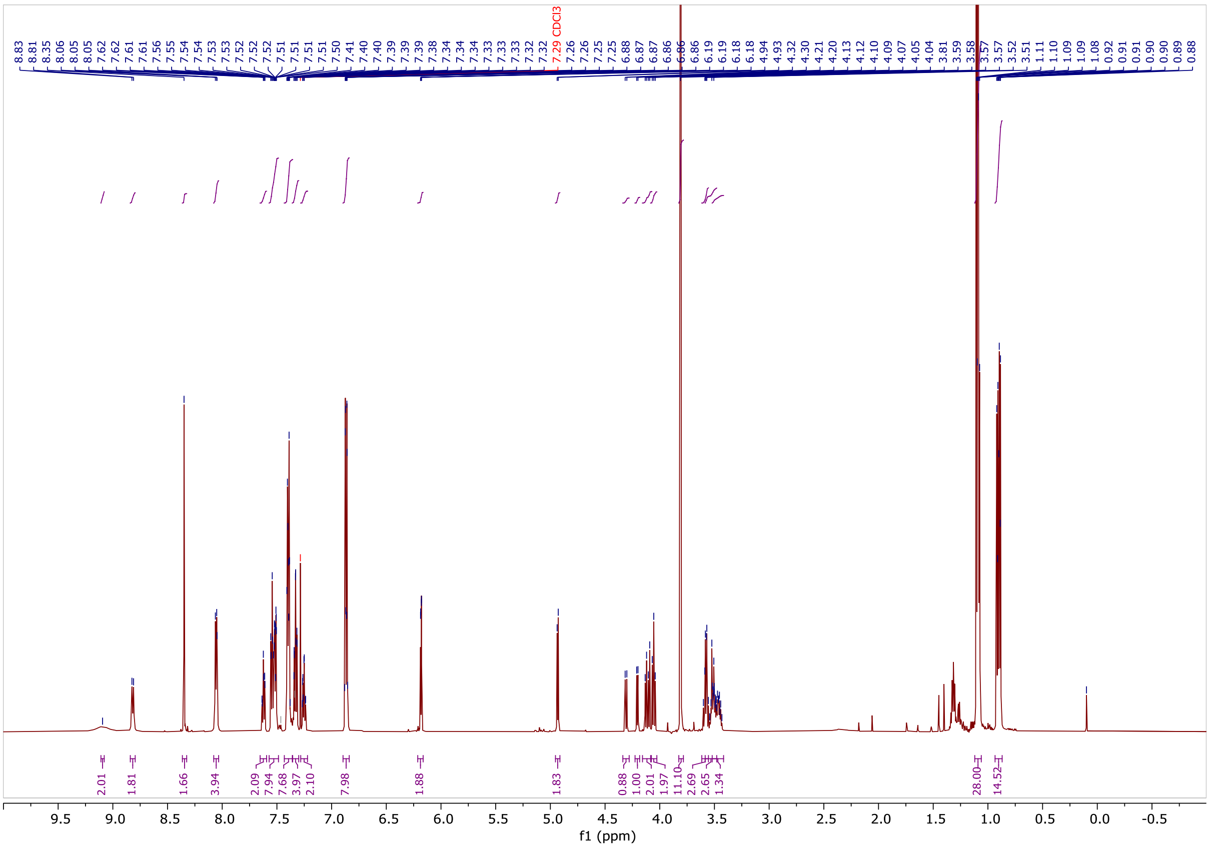

Supplementary Figure S216: ^{1}H NMR (600 MHz, $CDCl_3$) spectrum of **21**.

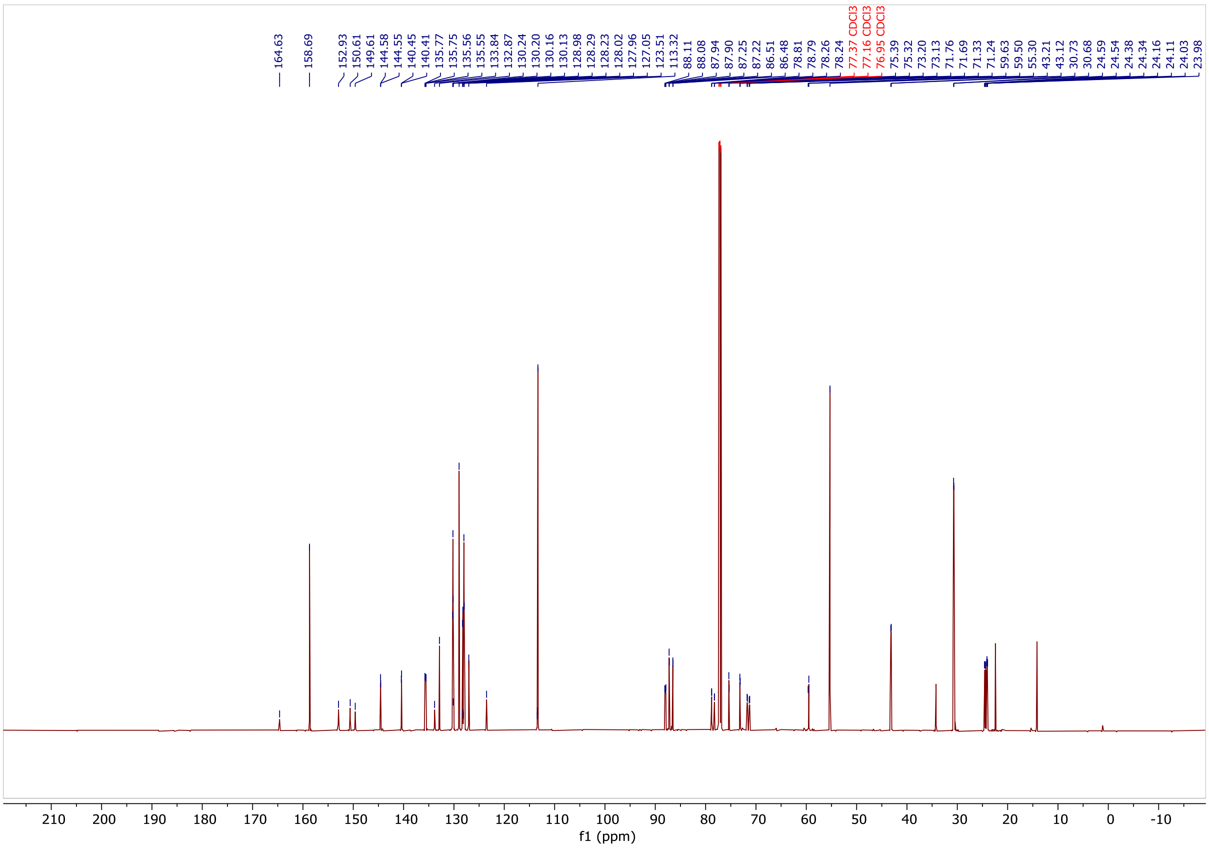

Supplementary Figure S217: $^{13}C\{^{1}H\}$ NMR (151 MHz, $CDCl_3$) spectrum of **21**.

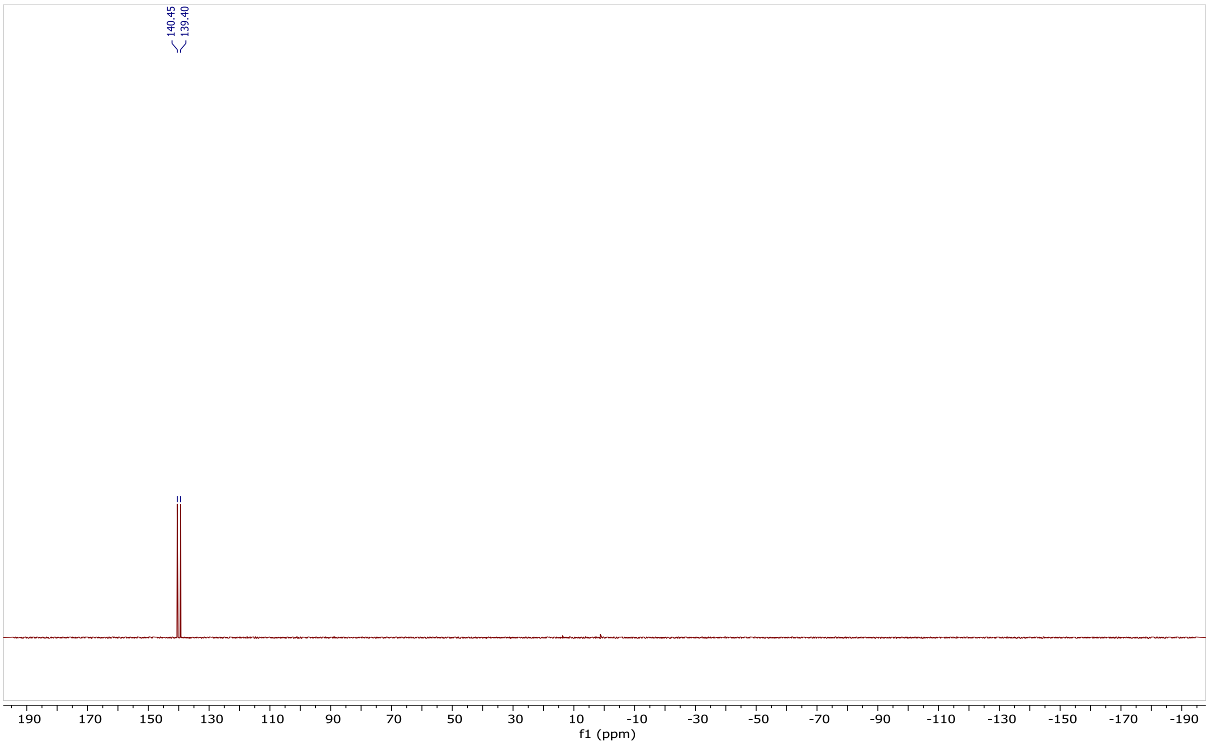

Supplementary Figure S218: ^{31}P NMR (162 MHz, $CDCl_3$) spectrum of **21.**

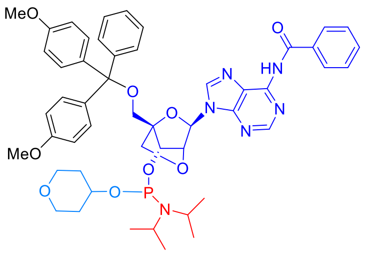

Chemical Formula: $C_{50}H_{57}N_6O_9P$
Exact Mass: 916.3925

22

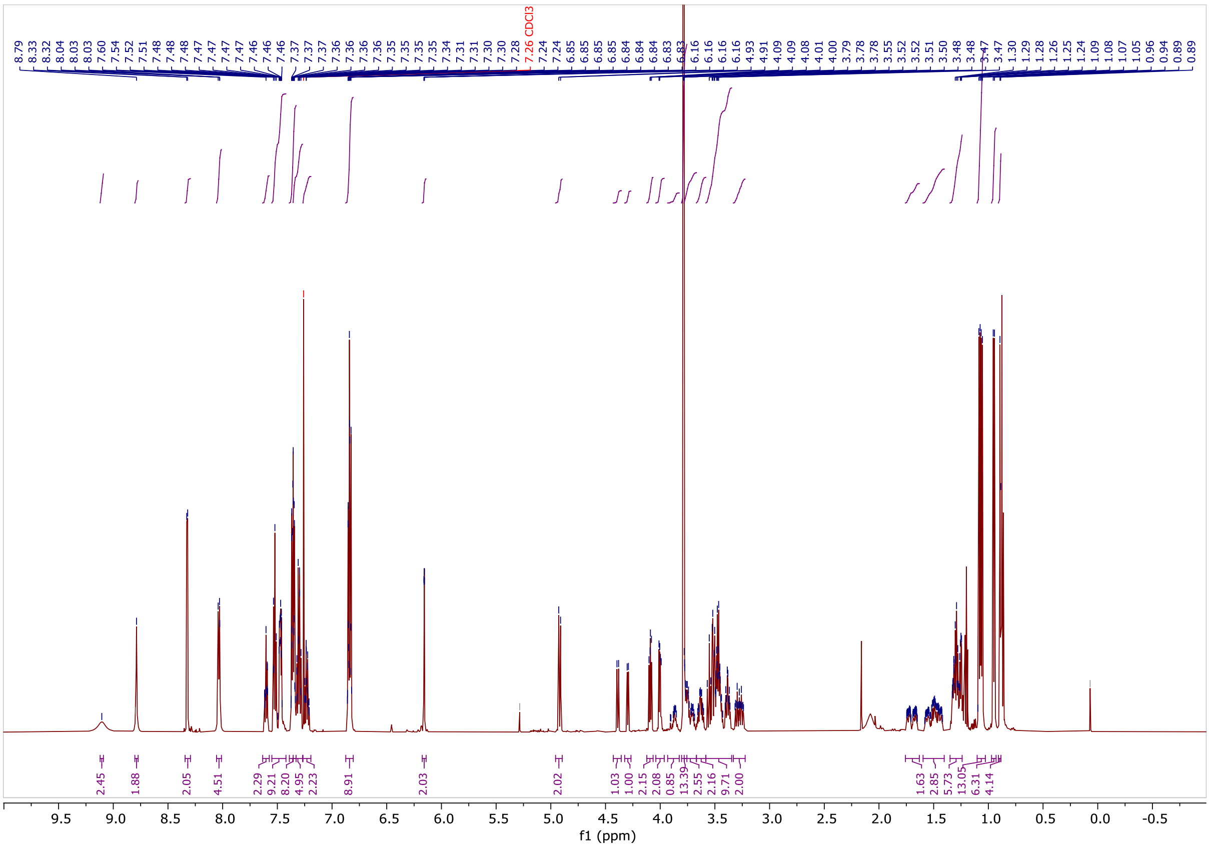

Supplementary Figure S219: ^{1}H NMR (600 MHz, $CDCl_3$) spectrum of **22**.

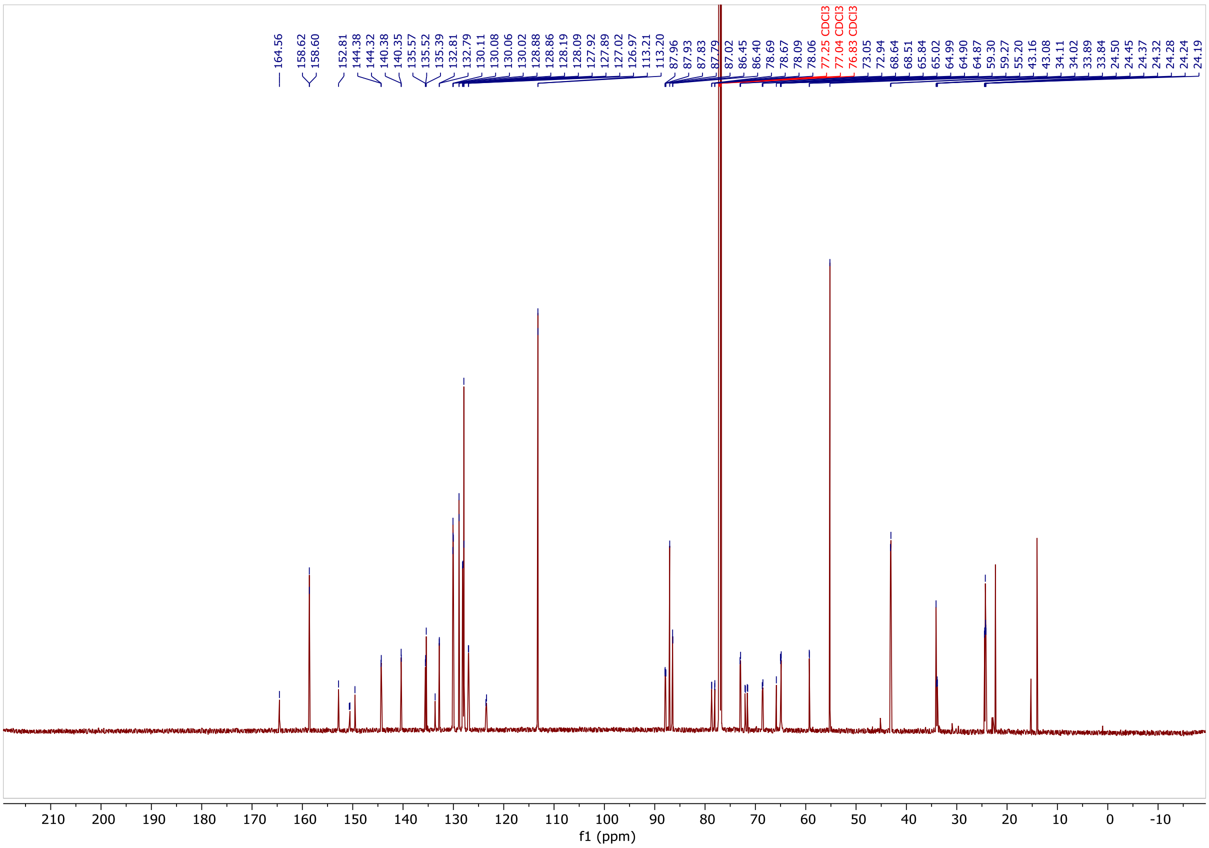

Supplementary Figure S220: $^{13}C\{^{1}H\}$ NMR (151 MHz, $CDCl_3$) spectrum of **22**.

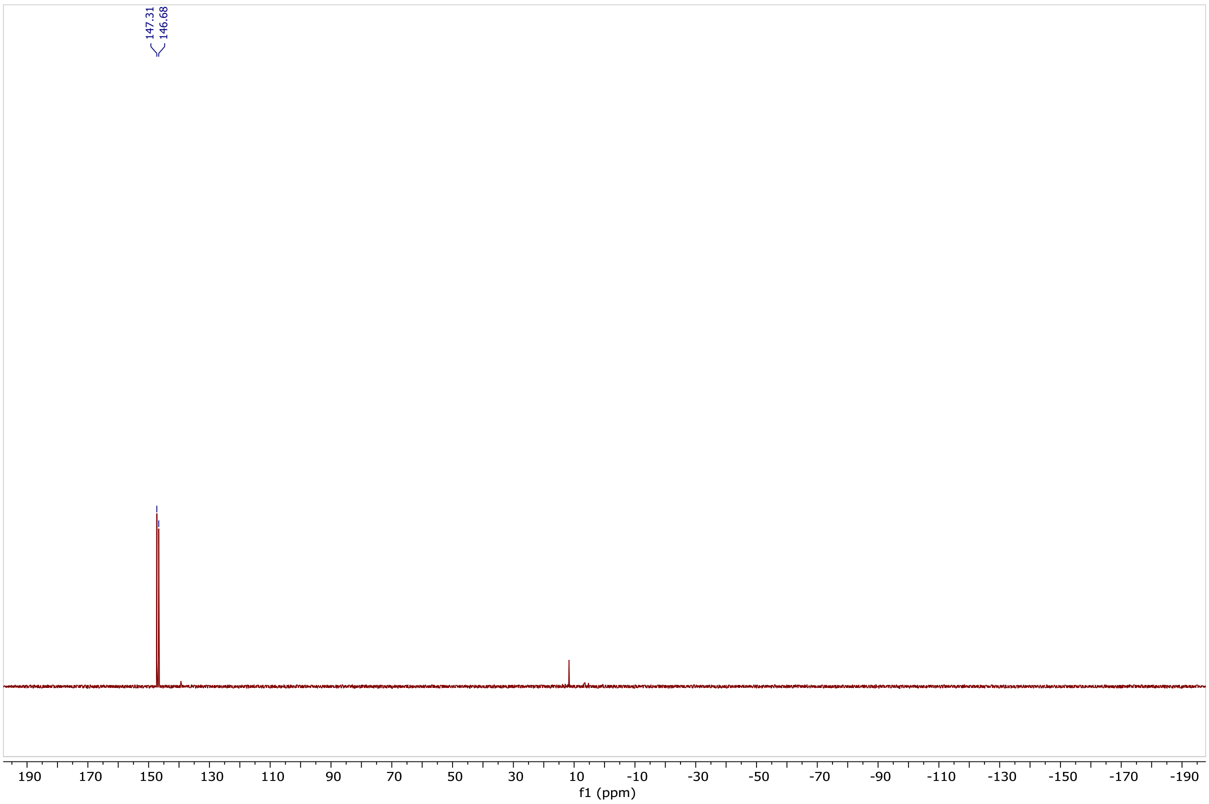

Supplementary Figure S221: ^{31}P NMR (162 MHz, $CDCl_3$) spectrum of **22.**

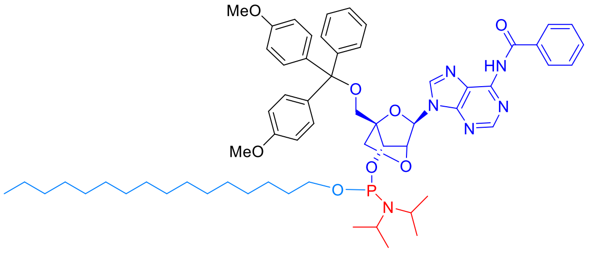

23

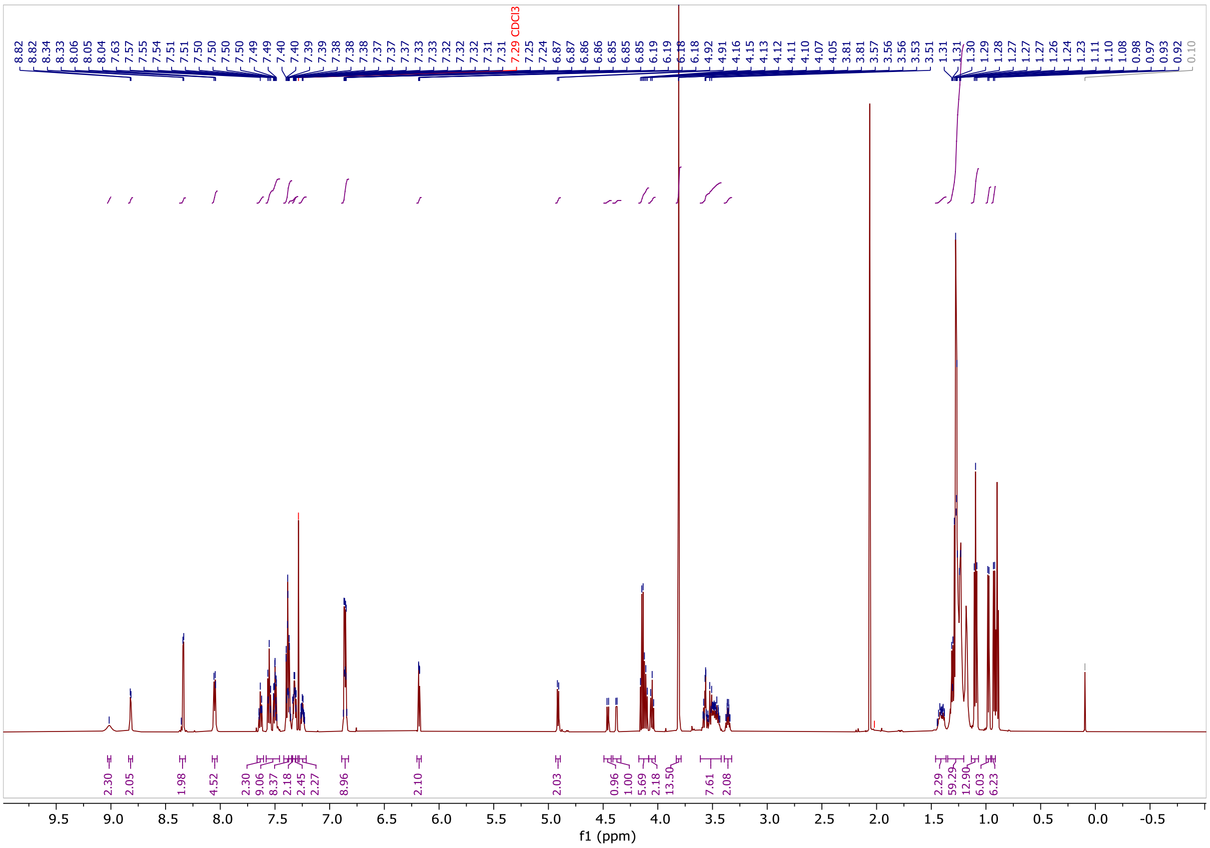

Supplementary Figure S222: ^{1}H NMR (600 MHz, $CDCl_3$) spectrum of **23**.

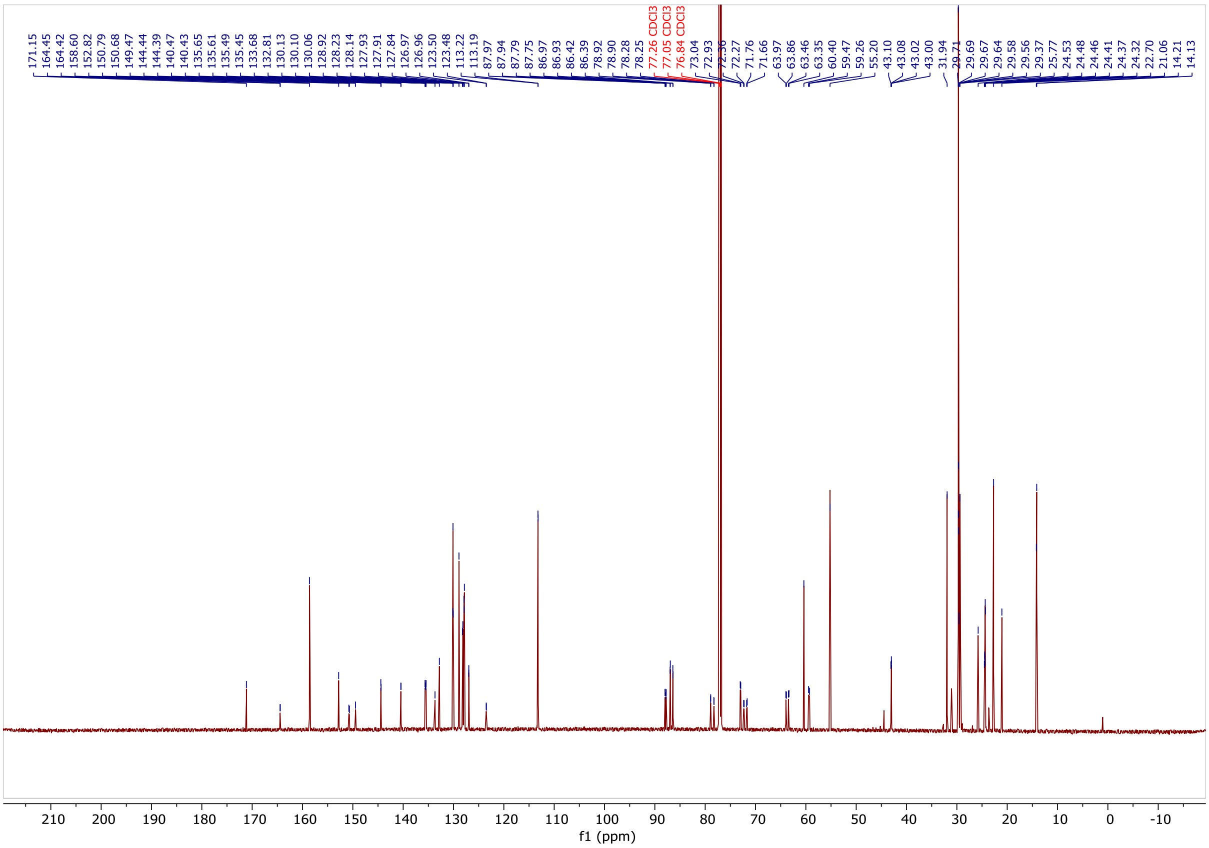

Supplementary Figure S223: $^{13}C\{^{1}H\}$ NMR (151 MHz, $CDCl_3$) spectrum of **23**.

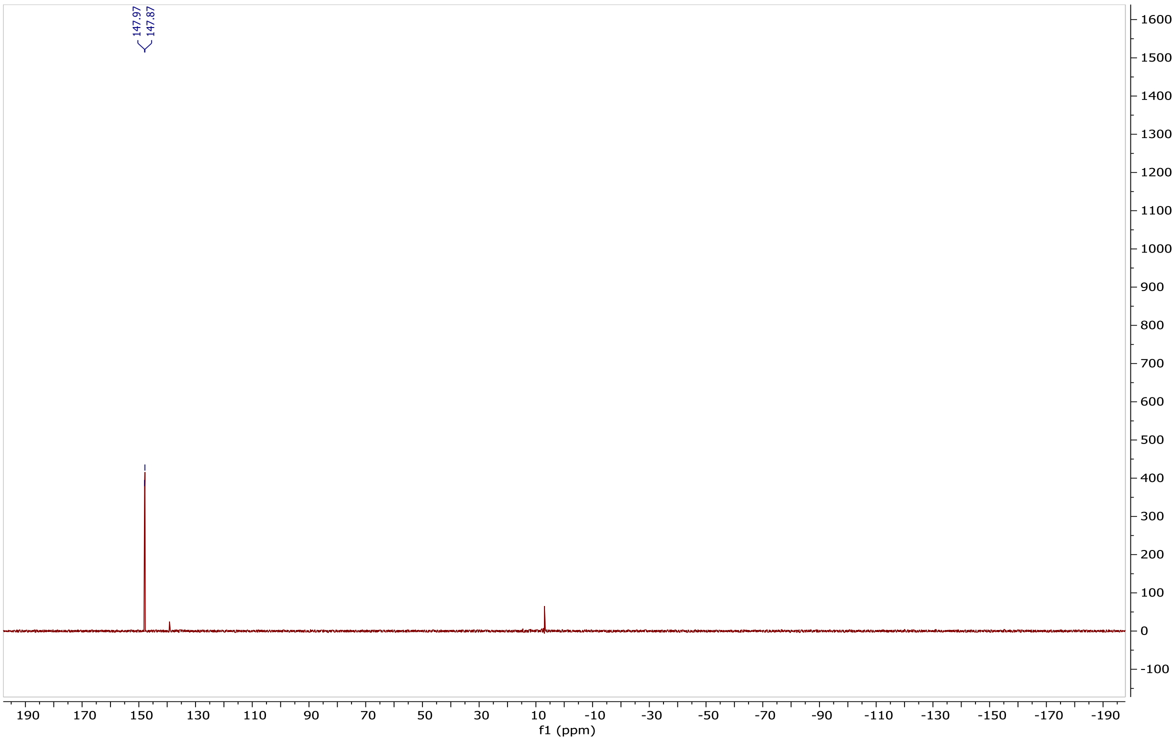

Supplementary Figure S224: ^{31}P NMR (162 MHz, $CDCl_3$) spectrum of **23.**

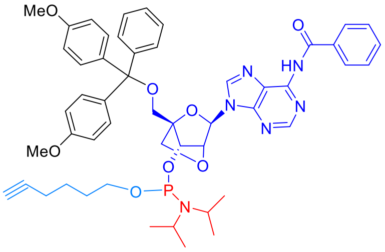

Chemical Formula: $C_{51}H_{57}N_6O_8P$
Exact Mass: 912.3975

24

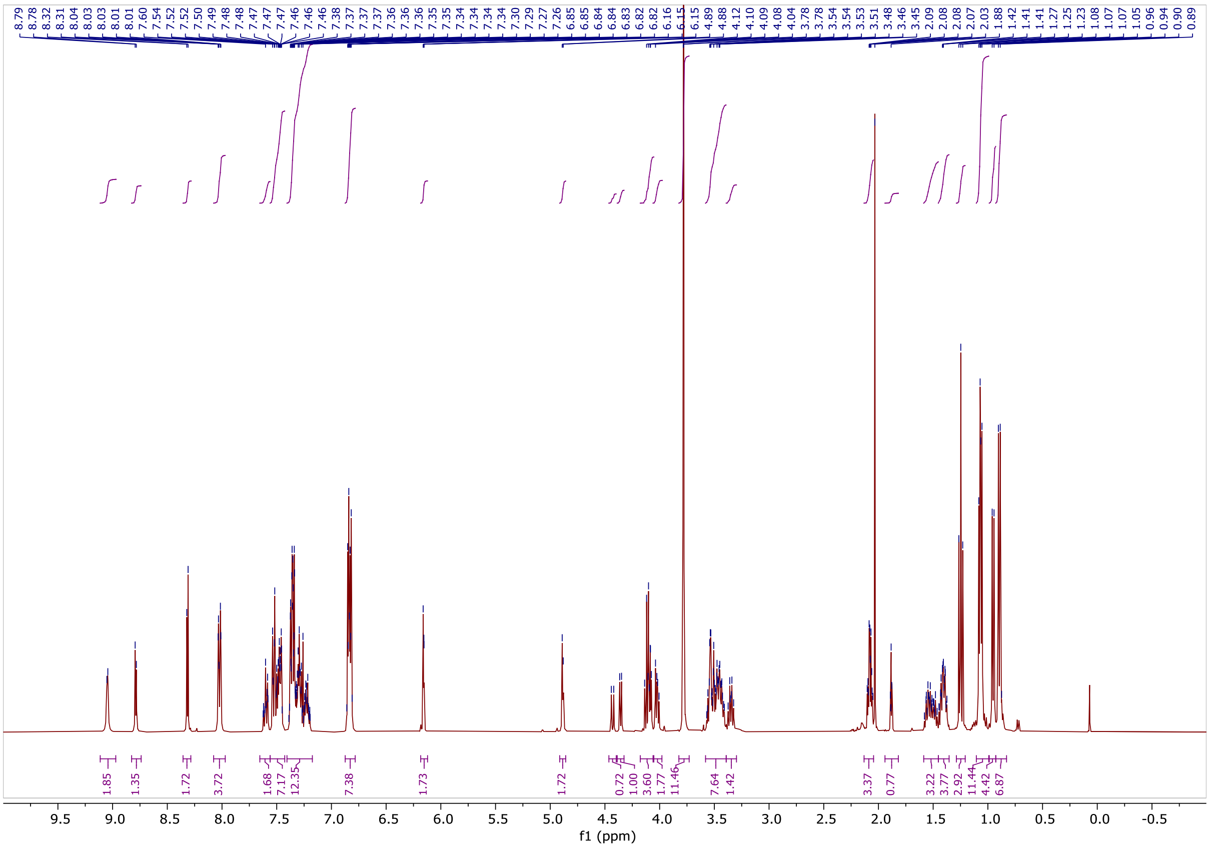

Supplementary Figure S225: ^{1}H NMR (400 MHz, $CDCl_3$) spectrum of **24**.

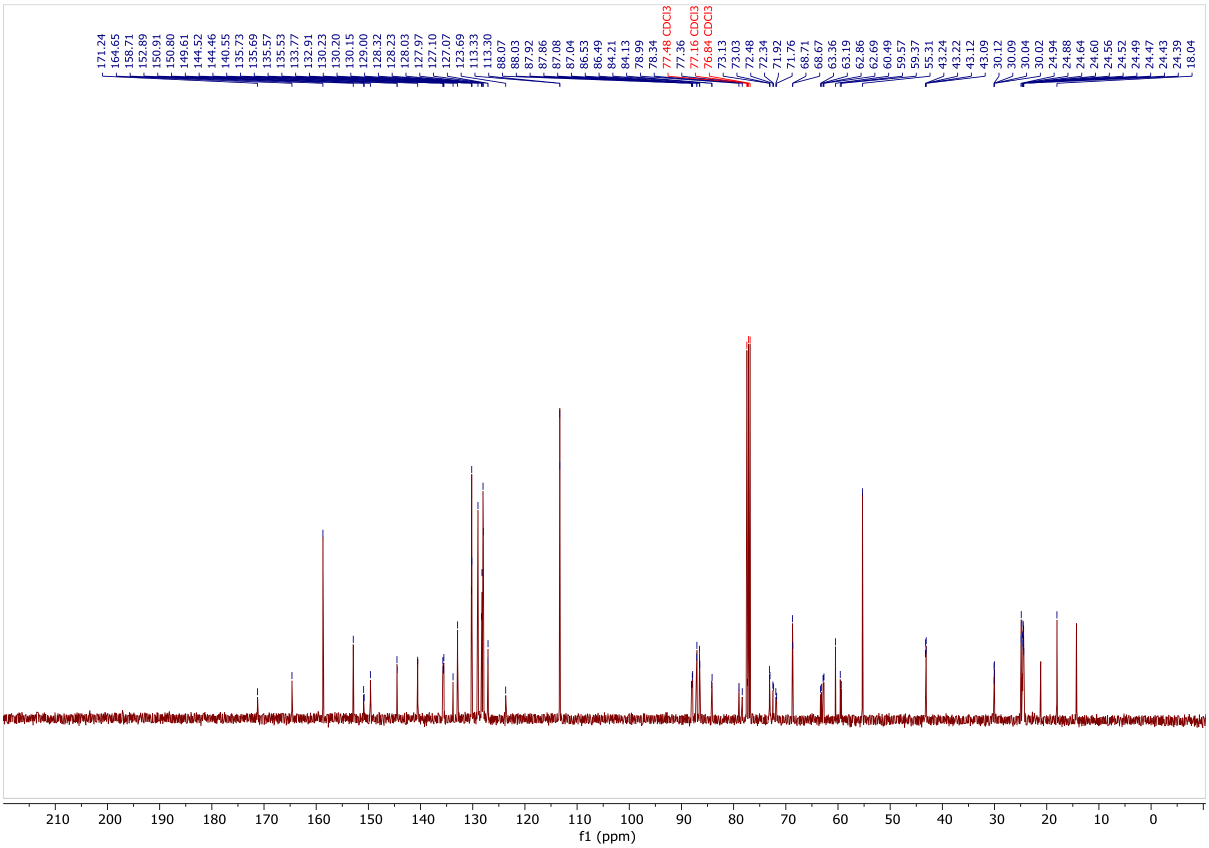

Supplementary Figure S226: $^{13}C\{^{1}H\}$ NMR (101 MHz, $CDCl_3$) spectrum of **24**.

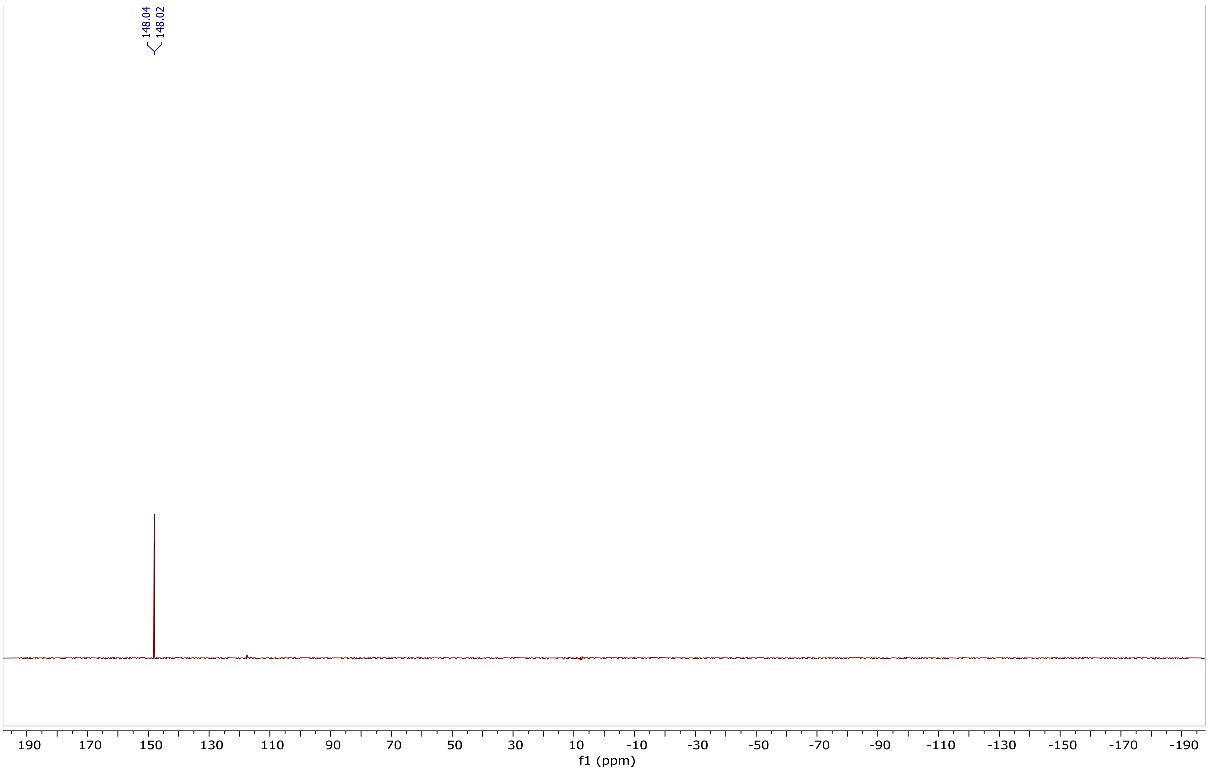

Supplementary Figure S227: ^{31}P NMR (162 MHz, $CDCl_3$) Spectrum of **24.**

9.0 Biological experiments, materials and methods

Cell culture

HeLa pLuc/705 cells[3] were maintained in Dulbecco's Modified Eagle Medium (DMEM; Gibco, 31966) supplemented with 10% fetal bovine serum (FBS) and 1X Antibiotic-Antimycotic (Gibco, 15240) at 37 °C and 5% CO_2 in a humidified incubator.

Transfection experiments

HeLa pLuc/705 cells were seeded at a density of 10,000 cells/well in 100 µL DMEM supplemented with 10% FBS and 1X Antibiotic-Antimycotic in a 96-well plate (Greiner, 655098) and incubated for 16 h at 37 °C and 5% CO_2 in a humidified incubator. Oligonucleotides in aqueous solution were diluted to 100 nM in 300 µL Opti-MEM (Gibco, 31985). Separately, 2.25 µL of Lipofectamine 2000 Transfection Reagent (Invitrogen, 11668) were diluted to a final volume of 300 µL in Opti-MEM and incubated for 5 min at room temperature. After 5 min, the diluted oligonucleotide and diluted transfection reagent were mixed and incubated for 20 min at room temperature to allow complex formation. After 20 min, the complexes were serially diluted in Opti-MEM to achieve final oligonucleotide concentrations of 50, 12.5, 3.125, and 0 nM. At 16 h post-seeding, the complete media was aspirated from the cells and replaced with the diluted complexes or Opti-MEM alone (i.e., the 0 nM treatment or untreated cells). The cells were incubated for 4 h at 37 °C and 5% CO_2 in a humidified incubator. At 4 h post-transfection, the complexes were aspirated from the cells and replaced with complete media, and the cells were incubated for a further 44 h at 37 °C and 5% CO_2 in a humidified incubator.

Gymnosis experiments

Oligonucleotides in aqueous solution were diluted to 40 µM in 400 µL Opti-MEM and then serially diluted in Opti-MEM to achieve oligonucleotide concentrations of 40, 20, 10, and 0 µM or 40, 20, 10, 5, 2.5, 1.25, 0.625, and 0 µM. Then, 50 µL of diluted oligonucleotide or Opti-MEM alone (i.e., the 0 µM treatment or untreated cells) and 50 µL HeLa pLuc/705 cells in DMEM supplemented with 6% FBS and 2X Antibiotic-Antimycotic were added to each well of a 96-well plate (Greiner, 655098) to achieve final oligonucleotide concentrations of 20, 10, 5, and 0 µM or 20, 10, 5, 2.5, 1.25, 0.625, 0.3125, and 0 µM and a seeding density of 10,000 cells/well in 100 µL DMEM supplemented with 3% FBS and 1X Antibiotic-Antimycotic. The cells were incubated for 72 h at 37 °C and 5% CO_2 in a humidified incubator.

Luciferase assays

At the end of the transfection or gymnosis experiments described above, the complete media was aspirated from the cells, and the cells were washed with phosphate buffered saline (PBS; Gibco, 10010) or PBS followed by 0.1 mg/mL heparin (Merck, H3149) in PBS followed by PBS, respectively. Lysis was performed in 100 µL of Glo Lysis Buffer (Promega, E2661) for 10 min at room temperature with shaking. Then, 50 µL of lysate and 50 µL of Bright-Glo Reagent (Promega, E2620) were added to each well of a 96-well white plate (Greiner, 655075), and after 2 min, luminescence was measured using a CLARIOstar microplate reader (BMG Labtech, software version 5.21.R2). Total protein quantification was carried out using a detergent compatible (DC) Protein Assay (Bio-Rad, 5000111) according to the manufacturer's

instructions. Briefly, a bovine serum albumin (BSA) standard (Thermo Scientific, 23209) was prepared at a concentration range of 20-20,000 µg/mL. Then, 5 µL of lysate or BSA standard were treated with 15 µL of Reagent A' and 120 µL of Reagent B and incubated for 20 min at room temperature. Absorbance at 750 nm was measured using a CLARIOstar microplate reader (BMG Labtech, software version 5.21.R2). Total protein quantities were calculated from the measured absorbances using the equation of the linear-fit standard curve in Microsoft Excel.

Both the transfection and gymnosis experiments described above were performed in biological duplicate or triplicate, where each biological replicate was performed in technical triplicate. To calculate the final fold increase over untreated value shown in the plots, the two or three biological replicates were averaged. Data in the plots are means ± standard deviations for two or three biological replicates (n=2 or 3). Statistical analyses were performed in GraphPad Prism 10 for macOS Version 10.2.2 (341).

Activity data under gymnosis conditions at 5, 10, and 20 µM were plotted against Δ Tm v RNA values (i.e., the change in Tm compared to ON44, a 2'-O-methyl RNA control, when paired with ON43, an unmodified RNA complement) and fit by nonlinear regression (Gaussian equation) in GraphPad Prism 10 for macOS Version 10.2.2 (341). The data in the plots are means ± standard deviations for three or more biological replicates ($n \geq 3$).

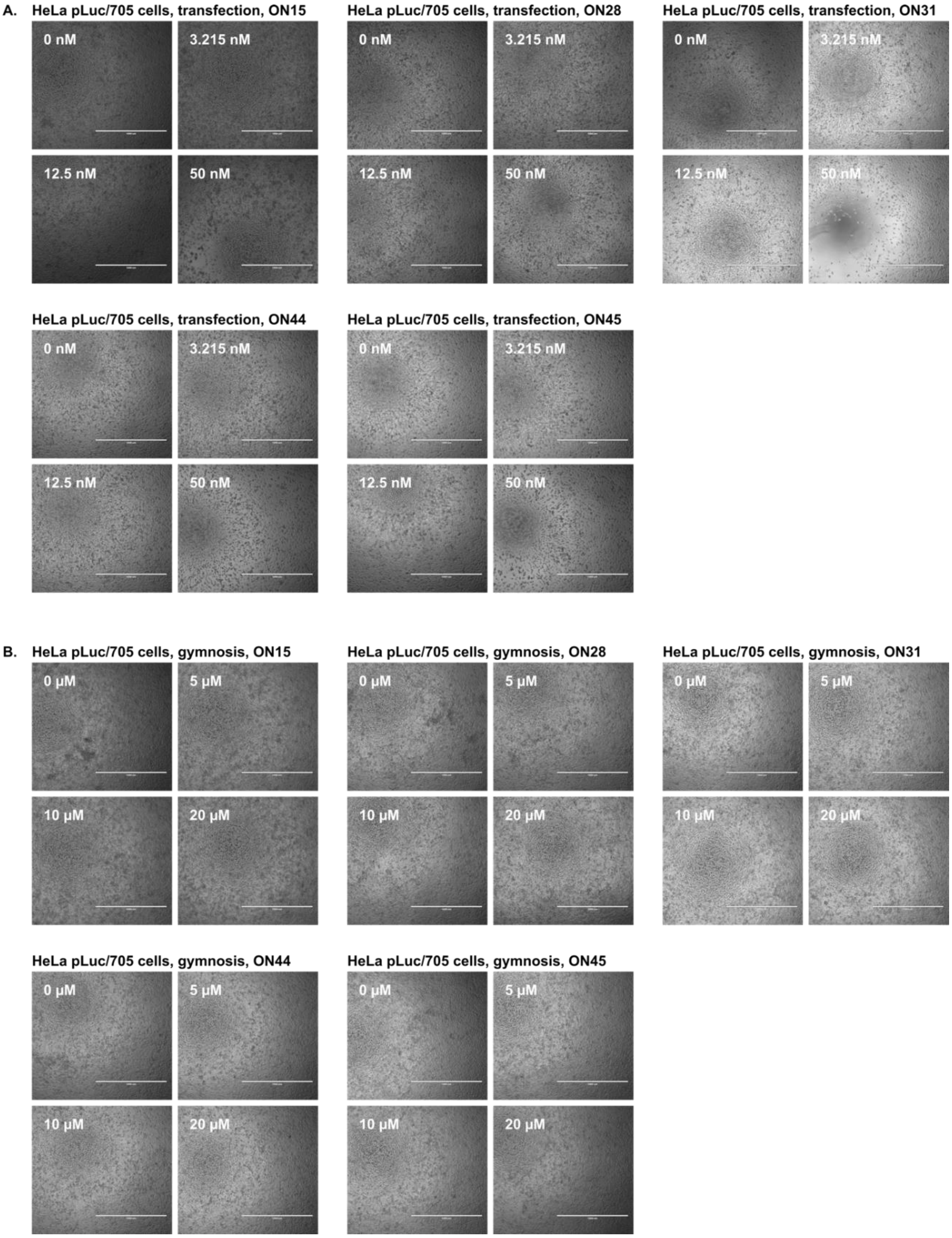

Supplementary Figure S228: Micrographs of HeLa pLuc/705 cells following treatment with selected ONs. A) ONs were transfected into cells at the indicated concentrations using Lipofectamine 2000, and images were captured at the assay endpoint 48 h later. B) ONs were applied to HeLa pLuc/705 cells at the indicated concentrations in the absence of a transfection reagent, and images were captured at the assay endpoint 72 h later. Scale bar = 1000 µm. Micrographs are representative of three images taken per condition.

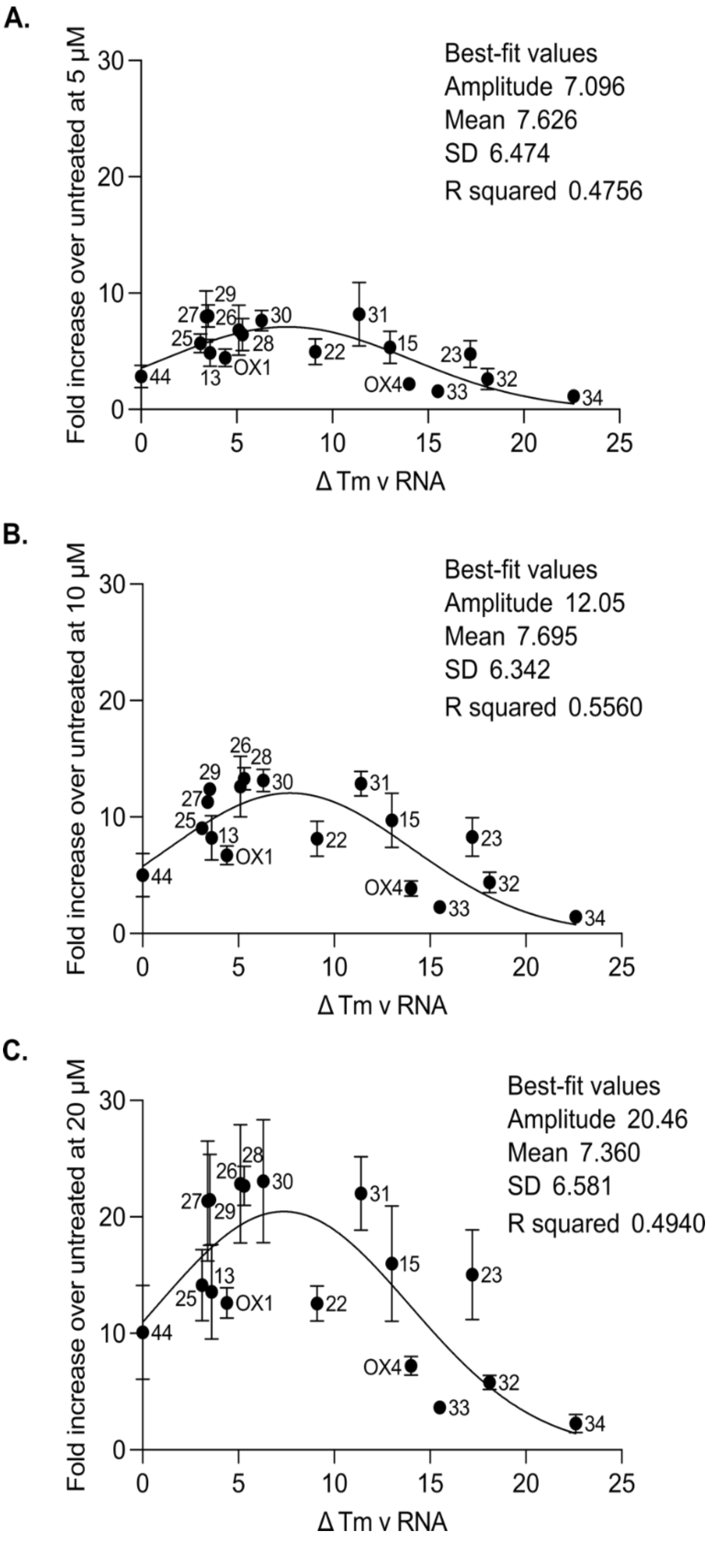

Supplementary Figure S229: Activity vs. ΔTm v RNA. The fold increase over untreated values for ONs applied to HeLa pLuc/705 cells in the absence of a transfection reagent at A) 5 µM, B) 10 µM, and C) 20 µM were plotted against the ΔTm v RNA values for the ONs, and the data were fit by nonlinear regression (Gaussian equation) in GraphPad Prism 10 for macOS Version 10.2.2 (341). Data are means ± standard deviations for three or more biological replicates ($n \geq 3$), where each biological replicate was performed in technical triplicate.

10.0 References

(1) Reddy, P. G.; Chun, B.-K.; Zhang, H.-R.; Rachakonda, S.; Ross, B. S.; Sofia, M. J. Stereoselective Synthesis of PSI-352938: A β-d-2′-Deoxy-2′-α-fluoro-2′-β-C-methyl-3′,5′-cyclic Phosphate Nucleotide Prodrug for the Treatment of HCV. *J. Org. Chem.* **2011**, *76* (10), 3782-3790.
(2) Roiban, G.-D.; Mehler, G.; Reetz, M. T. Palladium-Catalysed Amination of Aryl- and Heteroaryl Halides Using tert-Butyl Tetraisopropylphosphorodiamidite as an Easily Accessible and Air-Stable Ligand. *Eur. J. Org. Chem.* **2014**, *2014* (10), 2070-2076.
(3) Kang, S.-H.; Cho, M.-J.; Kole, R. Up-Regulation of Luciferase Gene Expression with Antisense Oligonucleotides: Implications and Applications in Functional Assay Development. *Biochemistry* **1998**, *37* (18), 6235-6239.
